# First-in-human study to evaluate safety, tolerability, and immunogenicity of heterologous regimens using the multivalent filovirus vaccines Ad26.Filo and MVA-BN-Filo administered in different sequences and schedules: A randomized, controlled study

**DOI:** 10.1371/journal.pone.0274906

**Published:** 2022-10-05

**Authors:** Viki Bockstal, Georgi Shukarev, Chelsea McLean, Neil Goldstein, Stephan Bart, Auguste Gaddah, Dickson Anumenden, Jeroen N. Stoop, Anne Marit de Groot, Maria G. Pau, Jenny Hendriks, Stephen C. De Rosa, Kristen W. Cohen, M. Juliana McElrath, Benoit Callendret, Kerstin Luhn, Macaya Douoguih, Cynthia Robinson

**Affiliations:** 1 Janssen Infectious Diseases and Vaccines, Leiden, The Netherlands; 2 Optimal Research, LLC, Rockville, Maryland, United States of America; 3 Janssen Infectious Diseases and Vaccines, Beerse, Belgium; 4 Vaccine and Infectious Disease Division, Fred Hutchinson Cancer Research Center, Seattle, Washington, United States of America; Public Health England, UNITED KINGDOM

## Abstract

**Background:**

Though clinically similar, Ebola virus disease and Marburg virus disease are caused by different viruses. Of the 30 documented outbreaks of these diseases in sub-Saharan Africa, eight were major outbreaks (≥200 cases; five caused by *Zaire ebolavirus* [EBOV], two by *Sudan ebolavirus* [SUDV], and one by *Marburg virus* [MARV]). Our purpose is to develop a multivalent vaccine regimen protecting against each of these filoviruses. This first-in-human study assessed the safety and immunogenicity of several multivalent two-dose vaccine regimens that contain Ad26.Filo and MVA-BN-Filo.

**Methods:**

Ad26.Filo combines three vaccines encoding the glycoprotein (GP) of EBOV, SUDV, and MARV. MVA-BN-Filo is a multivalent vector encoding EBOV, SUDV, and MARV GPs, and Taï Forest nucleoprotein. This Phase 1, randomized, double-blind, placebo-controlled study enrolled healthy adults (18–50 years) into four groups, randomized 5:1 (active:placebo), to assess different Ad26.Filo and MVA-BN-Filo vaccine directionality and administration intervals. The primary endpoint was safety; immune responses against EBOV, SUDV, and MARV GPs were also assessed.

**Results:**

Seventy-two participants were randomized, and 60 (83.3%) completed the study. All regimens were well tolerated with no deaths or vaccine-related serious adverse events (AEs). The most frequently reported solicited local AE was injection site pain/tenderness. Solicited systemic AEs most frequently reported were headache, fatigue, chills, and myalgia; most solicited AEs were Grade 1–2. Solicited/unsolicited AE profiles were similar between regimens. Twenty-one days post-dose 2, 100% of participants on active regimen responded to vaccination and exhibited binding antibodies against EBOV, SUDV, and MARV GPs; neutralizing antibody responses were robust against EBOV (85.7–100%), but lower against SUDV (35.7–100%) and MARV (0–57.1%) GPs. An Ad26.Filo booster induced a rapid further increase in humoral responses.

**Conclusion:**

This study demonstrates that heterologous two-dose vaccine regimens with Ad26.Filo and MVA-BN-Filo are well tolerated and immunogenic in healthy adults.

**ClinicalTrials.gov:**

NCT02860650.

## Introduction

Ebola virus disease (EVD) and Marburg virus disease (MVD) are caused by filoviruses that regularly and unpredictably cause deadly outbreaks in sub-Saharan Africa; EVD is often caused by *Zaire ebolavirus* (EBOV) or *Sudan ebolavirus* (SUDV), while MVD is caused by *Marburg virus* (MARV). Both diseases can cause severe illness in humans, with high case fatality rates of approximately 50.0% [1, 2].

Though caused by different viruses, EVD and MVD are clinically similar. There have been over 30 outbreaks of EVD and MVD since they were first identified in 1976 and 1967, respectively [1, 3, 4]. Of these, eight were major outbreaks (≥200 cases): five were caused by EBOV, two were caused by SUDV [1], and one was caused by MARV [2]. The 2014–2016 EVD outbreak in West Africa was caused by EBOV and was by far the largest outbreak to date, with over 28,000 cases and 11,000 deaths reported [5, 6]. More outbreaks of EBOV have occurred since then, including the 2018–2020 outbreak in the Democratic Republic of Congo, with over 3,400 cases and 2,200 deaths, which further highlights the devastating impact of EVD [7].

Two vaccines are licensed that provide protection against EVD caused by EBOV: recombinant vesicular stomatitis virus–Zaire Ebola virus (rVSV-ZEBOV-GP [Ervebo^®^]) [8, 9] and the heterologous two-dose Ad26.ZEBOV (Zabdeno), MVA-BN-Filo (MVA; Mvabea) vaccine regimen [10–12]. At present, there are no specific treatments or vaccines that have been licensed against MVD or against EVD caused by SUDV. The World Health Organization has emphasized the need for accelerated research to develop treatments and vaccines for SUDV and MARV due to a lack of medical countermeasures and the threat of future outbreaks [13, 14]. Heterologous two-dose Ad26.Filo and MVA regimens have previously been shown to provide protection from lethal infections with *Ebolavirus* caused by EBOV and SUDV and from MARV species in non-human primates; therefore, these are promising candidates for a broadly protective prophylactic filovirus vaccine [15]. While the heterologous two-dose Ad26.ZEBOV, MVA vaccine regimen is approved for use in a 56-day (56d) interval regimen, previous studies have found that the reverse vaccination order of MVA, Ad26.ZEBOV also elicits robust antibody and T cell responses [16, 17]. Furthermore, the reverse vaccination order with shorter intervals between doses may result in higher cellular responses compared to the Ad26.ZEBOV, MVA 56d interval regimen [16]. These results supported investigation of a 56d and a 14-day (14d) interval regimen, as well as the directionality of Ad26.Filo and MVA vaccine administration.

The objectives of this first-in-human (FIH) study were to evaluate the safety, tolerability, reactogenicity, and immunogenicity of several multivalent filovirus vaccine regimens, aimed to protect against EVD caused by EBOV or SUDV and against MVD caused by MARV.

## Methods

### Study design

**Fig 1:**
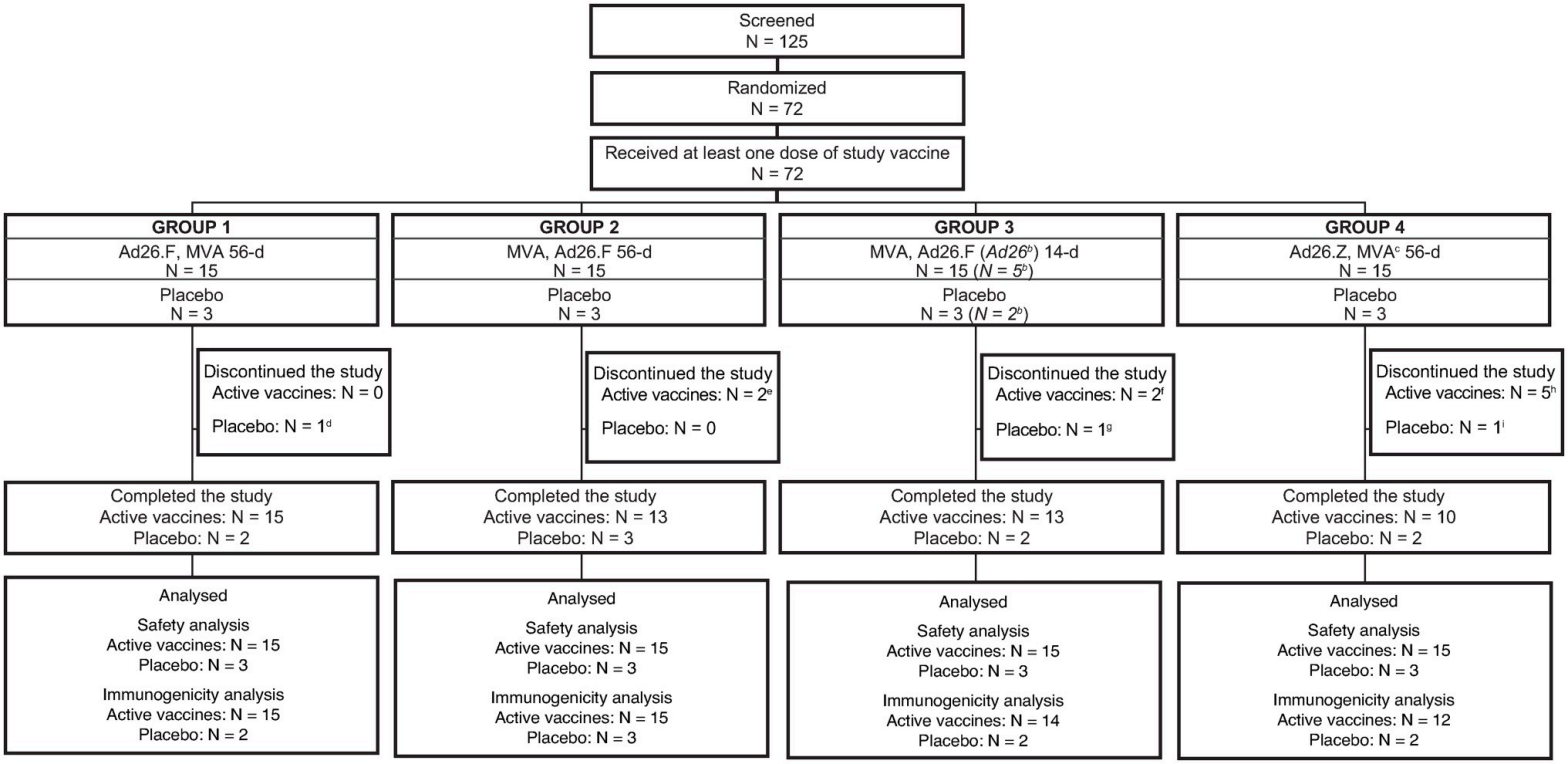
Participant disposition^a^. Ad26.F = Ad26.Filo; Ad26.Z = Ad26.ZEBOV; Inf U = infectious units; MVA = MVA-BN-Filo; N = number of participants. ^a^A total of four participants had a major protocol deviation. In Group 1, one participant received disallowed concomitant therapy (bleomycin and cisplatin) during the post-dose 2 follow-up period. In Group 2, one participant did not have a Day 8 visit. In Group 4, one participant did not have a Day 8 visit, and another participant received a disallowed concomitant therapy (prednisone) and, as a result, discontinued the study prior to receiving the second vaccination. Participants were considered lost to follow-up after multiple unsuccessful contact attempts. ^b^Only a subset of Group 3 participants received a booster vaccination. ^c^MVA-BN-Filo at a dose of 1x10^8^ Inf U. ^d^Participant was unable to attend visits due to a family emergency. This participant had only received dose 1 vaccination. ^e^Participants were lost to follow-up. One of two participants had only received dose 1 vaccination. ^f^One participant was lost to follow-up and one participant suddenly relocated. One of two participants had only received dose 1 vaccination. ^g^Withdrawal by the participant (no longer interested in participating). This participant had only received dose 1 vaccination. ^h^Withdrawal by the participant (one participant was out of town and no longer interested in participating, one participant did not want to receive the second vaccination, one participant relocated, and one participant moved out of state) and one participant was lost to follow-up. One additional participant was unable to receive the second vaccination due to taking disallowed medication (prednisone) to treat an adverse event of left hip pain; this participant did not discontinue study participation. Three of five participants had only received dose 1 vaccination. ^i^Participant was lost to follow-up. This participant had only received dose 1 vaccination.

This Phase 1, randomized, double-blind, placebo-controlled study (ClinicalTrials.gov: NCT02860650) was conducted at a single site in the United States, according to the Declaration of Helsinki and Good Clinical Practice guidelines. The study protocol and amendments were reviewed and approved by the Chesapeake IRB institutional review board. All participants provided written informed consent before screening. Following screening, participants were enrolled into four groups of 18 adults and then randomized 5:1 (active:placebo [0.9% saline]) within the group (Fig 1 in S1 File). This was based on a computer-generated randomization schedule prepared before the study by, or under supervision of, the sponsor and was balanced by using randomly permuted blocks. Participants, clinical staff, and site personnel remained blinded to the allocation of investigational products throughout the study, except the unblinded pharmacist. All vaccines and placebo were administered by intramuscular injection into the deltoid.

Ad26.Filo was administered at a dose of 9x10^10^ viral particles (vp), MVA at 5x10^8^ infectious units (Inf U; except for in the control group, Group 4, where MVA was administered at 1x10^8^ Inf U), and Ad26.ZEBOV at 5x10^10^ vp. Participants assigned to active regimens in Group 1 received Ad26.Filo followed by MVA 56 days later. Groups 2 and 3 received MVA followed by Ad26.Filo 56 days or 14 days later, respectively. A subset of seven participants in Group 3 also received a booster vaccination on Day 92 (n = 5, Ad26.Filo; n = 2, placebo) to assess reactivation of the immune responses (MVA, Ad26.Filo 14d plus Ad26.Filo booster). Group 4 served as a tolerability and immunogenicity study control group; participants received Ad26.ZEBOV followed by MVA 56 days later.

### Participants

Participants were eligible for study enrollment if they met the following key criteria: healthy adults aged 18–50 years old; no prior vaccination with a filovirus vaccine candidate; no known previous exposure to MARV, EBOV, SUDV, or *Taï Forest Ebolavirus* (TAFV). Full inclusion and exclusion criteria are provided in S1 Protocol.

### Vaccines

Ad26.Filo is a mixture of three Ad26-based recombinant, replication-incompetent vaccines in a 1:1:1 ratio, i.e., Ad26.ZEBOV (Zabdeno, Janssen-Cilag International N.V.), Ad26.SUDV, and Ad26.MARV encoding the glycoproteins (GPs) of EBOV Mayinga, SUDV Gulu, and MARV Angola, respectively. MVA-BN-Filo (MVA; Mvabea, Bavarian Nordic) is a recombinant, modified vaccinia Ankara-based, non-replicating vaccine, encoding the GPs of EBOV Mayinga, SUDV Gulu, MARV Musoke, and the nucleoprotein (NP) of TAFV.

### Endpoints and assessments

The primary study objective was to assess the safety and reactogenicity of the two-dose heterologous vaccine regimen. Participants were monitored for adverse events (AEs) for one hour immediately after vaccination; solicited local and systemic AEs were recorded in diary cards on the day of each study injection and for seven subsequent days. Unsolicited AEs were reported up to 28 days post-dose 2 (all groups), and up to 28 days after the booster dose (Group 3 booster subset only). Other safety observations included analysis of clinical laboratory tests, vital signs measurements, physical examinations, and electrocardiogram assessment. Serious AEs (SAEs) were reported throughout the study. AEs were graded according to the Food and Drug Administration (FDA) toxicity grading scale [18].

The secondary study objective was the assessment of EBOV, SUDV, and MARV GP-specific immunoglobulin G (IgG) binding antibody responses. Total IgG responses against EBOV Kikwit GP were assessed with an EBOV GP filovirus animal non-clinical group (FANG) enzyme-linked immunosorbent assay (ELISA) at Battelle Biomedical Research Center (summarized as geometric mean concentration [GMC]; units: ELISA units/mL [EU/mL]), as previously described [15, 17, 19–21]. An adaptation of this assay was used to assess IgG binding responses to the MARV Ci67 GP and SUDV Gulu GP. A FANG ELISA result was considered positive if the value was above the assay-specific lower limit of quantification (LLOQ), which was 66.96 EU/mL for EBOV, 14.86 EU/mL for SUDV, and 19.19 EU/mL for MARV. Values <LLOQ were imputed with LLOQ/2. For the calculation of fold increases, values <LLOQ were imputed with LLOQ. Immune responses to the study vaccine regimens were evaluated using serum samples collected immediately before each vaccination, at seven days and at 21 days after the second vaccination, and at 180, 240, and 360 days after the first vaccination. In the subset of participants receiving a booster vaccination, immune responses were also measured seven and 21 days post-booster. Immune responses to TAFV NP were not analyzed as the assays were not available at the time of the study.

The pre-defined exploratory objectives were the assessment of EBOV, SUDV, and MARV GP-specific neutralizing antibody responses and cellular immune responses. Serum samples were analyzed using EBOV Zaire-Makona GP, SUDV Gulu GP, and MARV Angola GP pseudovirion neutralization assays (psVNA; summarized as geometric mean titer [GMT]; unit: 50% inhibitory concentration [IC_50_] titer) at Monogram Biosciences, San Francisco, CA, as previously described [19, 20]. A psVNA result (IC_50_ titer) was considered positive if the specific IC_50_ titer was more than three times amphotropic murine leukemia virus (aMLV) titer (specificity control) and above the assay-specific LLOQ (120 for EBOV, 40 for SUDV and MARV), meaning that values that were less than or equal to three times aMLV or below the assay-specific LLOQ were considered negative and imputed with LLOQ/2. For the calculation of fold increases, values below the LLOQ were imputed with LLOQ. For both the ELISA and the psVNA, a participant was considered a responder if the sample was negative at baseline and positive post-baseline, or if the sample was positive at baseline and there was a greater than 3-fold increase from baseline.

Frozen peripheral blood mononuclear cells were analyzed by intracellular cytokine staining (ICS) at Fred Hutchinson Cancer Research Center, Seattle, Washington, United States of America (unit: total cytokine responses [% of subset]) [22]. T cell responses are expressed as the median percentage of responding T cells, with responder rates. Methodology for the ICS assay are included in the Methods and Table 1 in S1 File.

### Statistical analyses

Sample size determination was not based on formal hypothesis testing considerations but did comply with the recommended sample size (range, 20 to 80) by the Code of Federal Regulations 312.21 for FIH products (Ad26.Filo in this study) [23]. The full analysis set was defined as all participants who were randomized and received at least one dose of study vaccine, regardless of the occurrence of protocol deviations. Summaries and analysis of AEs, and of other safety data, were based on the full analysis set. The immunogenicity analysis set was defined as all participants who were randomized, were vaccinated, and had data from baseline and at least one post-vaccination immunogenicity blood draw. Analysis of immune responses was performed on the immunogenicity analysis set. Safety summaries were compiled and safety and immune response data were analyzed using SAS version 9.2. No formal statistical testing of safety data or immune response data was planned or performed.

## Results

### Participant characteristics/demographics

The first participant was enrolled on 14 September 2016 and the date of last participant last visit was 08 January 2018. Of the 125 participants screened, 72 participants were randomized (5:1, active:placebo) and received at least one dose of study vaccine. In total, 60/72 participants (83.3%) completed the study (Fig 1). Participant demographics were similar between treatment groups (Table 1). The majority of participants were White (36/72, 50.0%) or Black or African American (26/72, 36.1%), were female (40/72; 55.6%), and the median age was 28.5 years.

**Table 1:** Participant demographics and characteristics; full analysis set.

	All participants	56-day interval				14-day interval	
		Group 1	Group 2	Group 4	Pooled placebo (Groups 1, 2, and 4)	Group 3	
		Ad26.F, MVA	MVA, Ad26.F	Ad26.Z, MVA^a^	Placebo, Placebo	MVA, Ad26.F	Placebo, Placebo
**N**	72	15	15	15	9	15	3
**Male, n (%)**	32 (44.4)	5 (33.3)	8 (53.3)	5 (33.3)	5 (55.6)	7 (46.7)	2 (66.7)
**Median age, years**	28.5	29.0	27.0	28.0	28.0	28.0	35.0
Range	19;50	20;50	19;50	19;43	25;39	21;47	35;48
**Median BMI, kg/m²**	25.4	25.8	23.3	25.1	26.0	25.80	24.80
**Race, n (%)**							
White	36 (50.0)	7 (46.7)	5 (33.3)	8 (53.3)	5 (55.6)	8 (53.3)	3 (100)
Black or African American	26 (36.1)	5 (33.3)	6 (40.0)	6 (40.0)	4 (44.4)	5 (33.3)	0
Asian	6 (8.3)	2 (13.3)	3 (20.0)	0	0	1 (6.7)	0
Native Hawaiian or other Pacific Islander	1 (1.4)	1 (6.7)	0	0	0	0	0
Other	1 (1.4)	0	1 (6.7)	0	0	0	0
Multiple	2 (2.8)	0	0	1 (6.7)	0	1 (6.7)	0

Ad26.F = Ad26.Filo; Ad26.Z = Ad26.ZEBOV; BMI = body mass index; Inf U = infectious units; MVA = MVA-BN-Filo.

^a^ MVA-BN-Filo at a dose of 1x10^8^ Inf U.

### Safety

Solicited local and systemic AEs were mostly Grade 1 or 2 (Table 2), were transient in nature, and were generally reported more frequently in participants who received an active vaccine than in those who received placebo. There were no Grade 4 AEs, no deaths, and no AEs leading to permanent discontinuation of study vaccination.

**Table 2:** Solicited local and systemic AEs by worst severity grade by dose; full analysis set.

	Grade	Ad26.F	MVA	MVA^a^	Ad26.Z	Placebo
**N**	-	48	45	11	15	23
**Local AEs**						
Any event, n (%)	Any	38 (79.2)	40 (88.9)	5 (45.5)	13 (86.7)	4 (17.4)
	Grade 3	2 (4.2)	0	0	2 (13.3)	0
Erythema/ Redness	Any	5 (10.4)	0	0	0	0
	Grade 3	1 (2.1)	0	0	0	0
Itching	Any	8 (16.7)	4 (8.9)	1 (9.1)	1 (6.7)	0
	Grade 3	0	0	0	0	0
Pain/Tenderness	Any	38 (79.2)	40 (88.9)	4 (36.4)	13 (86.7)	4 (17.4)
	Grade 3	2 (4.2)	0	0	2 (13.3)	0
Swelling/ Induration	Any	11 (22.9)	12 (26.7)	0	3 (20)	1 (4.3)
	Grade 3	0	0	0	1 (6.7)	0
Warmth	Any	14 (29.2)	10 (22.2)	1 (9.1)	4 (26.7)	1 (4.3)
	Grade 3	0	0	0	1 (6.7)	0
**Systemic AEs**						
Any event, n (%)	Any	43 (89.6)	28 (62.2)	4 (36.4)	12 (80)	13 (56.5)
	Grade 3	11 (22.9)	0	1 (9.1)	3 (20)	1 (4.3)
Arthralgia	Any	22 (45.8)	2 (4.4)	1 (9.1)	4 (26.7)	1 (4.3)
	Grade 3	2 (4.2)	0	0	1 (6.7)	0
Chills	Any	31 (64.6)	4 (8.9)	0	7 (46.7)	0
	Grade 3	6 (12.5)	0	0	2 (13.3)	0
Fatigue	Any	36 (75)	20 (44.4)	3 (27.3)	9 (60)	6 (26.1)
	Grade 3	3 (6.3)	0	1 (9.1)	2 (13.3)	0
General itching	Any	5 (10.4)	3 (6.7)	1 (9.1)	1 (6.7)	1 (4.3)
	Grade 3	0	0	0	0	0
Headache	Any	38 (79.2)	14 (31.1)	1 (9.1)	9 (60)	7 (30.4)
	Grade 3	6 (12.5)	0	0	3 (20)	0
Myalgia	Any	28 (58.3)	6 (13.3)	2 (18.2)	6 (40)	1 (4.3)
	Grade 3	5 (10.4)	0	0	2 (13.3)	0
Nausea	Any	20 (41.7)	4 (8.9)	0	1 (6.7)	4 (17.4)
	Grade 3	1 (2.1)	0	0	1 (6.7)	1 (4.3)
Pyrexia	Any	10 (20.8)	1 (2.2)	0	2 (13.3)	0
	Grade 3	1 (2.1)	0	0	0	0
Rash	Any	0	1 (2.2)	1 (9.1)	0	0
	Grade 3	0	0	0	0	0
Vomiting	Any	5 (10.4)	1 (2.2)	0	0	2 (8.7)
	Grade 3	1 (2.1)	0	0	0	0

Ad26.F = Ad26.Filo; Ad26.Z = Ad26.ZEBOV; AEs = adverse events; Inf U = infectious units; MVA = MVA-BN-Filo; n (%) = number (percentage) of doses with one or more events.

^a^ MVA-BN-Filo at a dose of 1x10^8^ Inf U.

The most frequently reported solicited local AE was injection site pain/tenderness (Table 2), which was reported more frequently following active vaccines than following placebo (post-Ad26.Filo [38/48, 79.2%]; post-MVA 5x10^8^ Inf U [40/45, 88.9%]; post-MVA 1x10^8^ Inf U [4/11, 36.4%]; post-Ad26.ZEBOV [13/15, 86.7%]; and post-placebo [4/23, 17.4%]). In total, four participants experienced Grade 3 solicited local AEs: two of 48 (4.2%) participants who received Ad26.Filo (injection site pain/tenderness, n = 2; erythema/redness, n = 1) and two of 15 (13.3%) participants who received Ad26.ZEBOV (injection site pain/tenderness, n = 2; swelling/induration and warmth, n = 1).

The most frequent solicited systemic AEs were headache, fatigue, chills, and myalgia (Table 2). In total, 16 participants (11.3%) experienced Grade 3 solicited systemic AEs; the majority of these (11/16) were reported following vaccination with Ad26.Filo. The most frequent Grade 3 solicited systemic AEs were headache and chills after vaccination with Ad26.Filo (6/48, 12.5% for both), and headache after Ad26.ZEBOV dosing (3/15, 20.0%).

The most frequent unsolicited AE across all doses was a decrease in hemoglobin (Table 3). The majority of unsolicited AEs were Grade 1 or 2 in severity and considered unrelated to a study vaccine by the investigator. Two Grade 3 unsolicited AEs were reported, both of which were laboratory abnormalities. Grade 3 decreased hemoglobin from baseline was observed in one participant (1/45, 2.2%) seven days post-dose 2 vaccination with MVA 5x10^8^ Inf U. The abnormality was judged to be clinically insignificant and reported as a Grade 3 unsolicited AE unrelated to the study vaccine. A repeat test was done on Day 240 and assessed as Grade 1. Grade 3 decreased segmented neutrophils were observed in one participant (1/48, 2.1%) seven days post-dose 2 vaccination with Ad26.Filo. The abnormality was judged to be clinically insignificant and reported as a Grade 3 unsolicited AE unrelated to the study vaccine. A repeat test was done on the Day 78 visit; the AE was assessed as Grade 2. A total of two SAEs (germ cell neoplasm and appendicitis) were reported in the study; these occurred more than 28 days post-vaccination and were not considered related to the study vaccine by the investigator.

**Table 3:** Unsolicited AEs most frequently reported, by system organ class and dictionary-derived term (reported by ≥10% participants in any regimen) by dose; full analysis set.

	Grade	Ad26.F	MVA	MVA^a^	Ad26.Z	Placebo
**N**		48	45	11	15	23
**Any unsolicited AE, n (%)**	Any	34 (70.8)	26 (57.8)	5 (45.5)	9 (60)	13 (56.5)
	Grade 3	1 (2.1)	1 (2.2)	0	0	0
**MedDRA System Organ Class**						
**Investigations**	Any	16 (33.3)	17 (37.8)	1 (9.1)	2 (13.3)	8 (34.8)
	Grade 3	1 (2.1)	1 (2.2)	0	0	0
Hemoglobin decreased	Any	9 (18.8)	13 (28.9)	1 (9.1)	1 (6.7)	3 (13)
	Grade 3	0	1 (2.2)	0	0	0
Red blood cells urine	Any	3 (6.3)	5 (11.1)	0	0	4 (17.4)
	Grade 3	0	0	0	0	0
**Renal and urinary disorders**	Any	5 (10.4)	7 (15.6)	1 (9.1)	1 (6.7)	4 (17.4)
	Grade 3	0	0	0	0	0
Proteinuria	Any	5 (10.4)	7 (15.6)	1 (9.1)	1 (6.7)	4 (17.4)
	Grade 3	0	0	0	0	0
**Infections and infestations**	Any	7 (14.6)	5 (11.1)	0	2 (13.3)	2 (8.7)
	Grade 3	0	0	0	0	0
Upper respiratory tract infection	Any	5 (10.4)	3 (6.7)	0	1 (6.7)	1 (4.3)
	Grade 3	0	0	0	0	0
**Skin and subcutaneous tissue disorders**	Any	6 (12.5)	2 (4.4)	2 (18.2)	1 (6.7)	1 (4.3)
	Grade 3	0	0	0	0	0
**Respiratory, thoracic, and mediastinal disorders**	Any	2 (4.2)	4 (8.9)	2 (18.2)	1 (6.7)	0
	Grade 3	0	0	0	0	0
**Gastrointestinal disorders**	Any	6 (12.5)	0	0	0	2 (8.7)
	Grade 3	0	0	0	0	0
**General disorders and administration site conditions**	Any	5 (10.4)	1 (2.2)	0	0	0
	Grade 3	0	0	0	0	0
**Injury, poisoning and procedural complications**	Any	1 (2.1)	1 (2.2)	0	2 (13.3)	0
	Grade 3	0	0	0	0	0

Ad26.F = Ad26.Filo; Ad26.Z = Ad26.ZEBOV; AEs = adverse events; Inf U = infectious units; MVA = MVA-BN-Filo; n (%) = number (percentage) of doses with one or more events; N = number of doses.

^a^ MVA-BN-Filo at a dose of 1x10^8^ Inf U.

No notable trend in clinical laboratory abnormalities was observed. Most participants (76.1%) meeting the FDA toxicity grading criteria for decreased hemoglobin from baseline had hemoglobin values within the laboratory normal ranges. Any decrease in hemoglobin level from baseline was considered to be a graded laboratory abnormality. The most frequently reported graded laboratory abnormalities were a decreased hemoglobin level from baseline (59.1–73.3% across all participants, including placebo) and protein in urine (9.1–30.4% across all participants, including placebo; Table 2 in S1 File). Most cases of protein in urine were Grade 1 (i.e., trace protein). The majority of abnormal vital signs measurements were Grade 1 or 2 in severity. Grade 3 hypertension (systolic) was reported in two participants following vaccination with Ad26.Filo (n = 1 on Day 1 [Group 1 –Ad26.Filo, MVA 56d]; n = 1 on Day 15 [Group 3 –MVA, Ad26.Filo 14d]), but neither abnormality was deemed to be clinically significant or reported as an AE.

### Immunogenicity

#### Binding antibody responses

**Fig 2:**
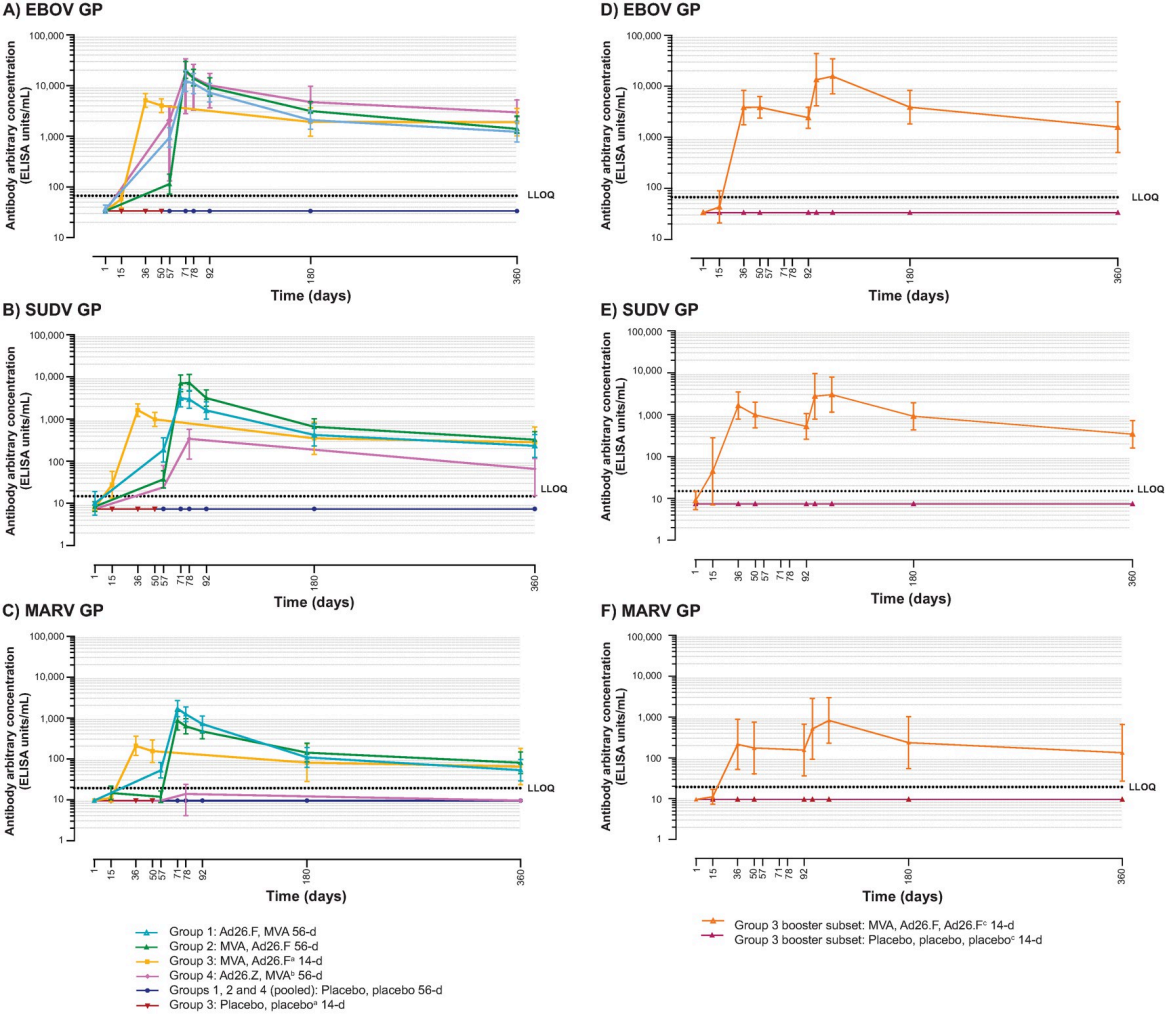
GP-specific binding antibody responses (ELISA units/mL): Regimen profiles (geometric means with 95% CIs); immunogenicity analysis set. Ad26.F = Ad26.Filo; Ad26.Z = Ad26.ZEBOV; CIs = confidence intervals; EBOV = *Zaire ebolavirus*; ELISA = enzyme-linked immunosorbent assay; GP = glycoprotein; Inf U = infectious units; LLOQ = lower limit of quantification; MARV = *Marburg virus*; MVA = MVA-BN-Filo; SUDV = *Sudan ebolavirus*. ^a^Includes all Group 3 participants up to and including the Day 50 time point, after which point only Group 3 participants who did not receive a third dose of vaccine/placebo on Day 92 are included. ^b^MVA-BN-Filo at a dose 1x10^8^ Inf U. ^c^All time points include only Group 3 participants who received three doses of vaccine/placebo.

EBOV, SUDV, and MARV GP-specific binding antibody concentrations are summarized in Tables 3–8 in S1 File, respectively, and depicted in Fig 2. In placebo recipients, binding antibody responses were not observed (<LLOQ) at any time point.

#### Binding antibody responses—EBOV GP

At 21 days post-dose 2, 100.0% of participants in the filovirus regimen groups (42/42) and in the control group (Ad26.ZEBOV, MVA 56d; 11/11) exhibited binding antibody responses against EBOV. The highest GMC at 21 days post-dose 2 was observed after MVA, Ad26.Filo 56d (13,867 EU/mL) and was comparable to the response observed in the control group (11,295 EU/mL). At Day 360, the magnitude of the binding antibody responses had decreased, yet responses persisted in 100.0% of participants in filovirus regimen groups (35/35) and in the control group (9/9), with similar GMCs across the groups (filovirus regimen groups: 1,229–1,914 EU/mL; control group: 2,025 EU/mL).

For the MVA, Ad26.Filo 14d plus Ad26.Filo booster regimen, 100.0% (5/5) of participants exhibited a strong increase in binding antibody responses seven days post-booster vaccination, with a ~5.5-fold increase in GMC (Day 92 pre-booster GMC: 2,425 EU/mL; Day 99 GMC: 13,445 EU/mL). At Day 360, binding antibody responses were observed in 100.0% (4/4; GMC: 1,592 EU/mL) of participants, similar to that of participants who did not receive a booster vaccination.

#### Binding antibody responses—SUDV GP

At 21 days post-dose 2, 100.0% of participants in filovirus regimen groups (42/42) and in the control group (Ad26.ZEBOV, MVA 56d; 11/11) exhibited binding antibody responses against SUDV. The highest 21 days post-dose 2 GMC of 7,273 EU/mL was observed after MVA, Ad26.Filo 56d, while the GMC in the control group was 259 EU/mL. At Day 360, similar to the EBOV GP–specific responses, the magnitude of the binding antibody responses to SUDV GP had decreased, yet responses persisted in 92.9–100.0% of participants in filovirus regimen groups, with similar GMCs across the groups (231–324 EU/mL). Responses in the control group at Day 360 persisted in 77.8% of participants (7/9, GMC: 43 EU/mL).

For the MVA, Ad26.Filo 14d plus Ad26.Filo booster regimen, 100.0% (5/5) of participants exhibited a strong increase in binding antibody responses against SUDV GP within seven days after booster vaccination, with a ~5.2-fold increase in GMC (Day 92: 524 EU/mL; Day 99: 2,748 EU/mL). At Day 360, responses against SUDV GP were seen in 100.0% (4/4) of participants with a GMC of 343 EU/mL, similar to that observed in participants who did not receive a booster vaccination.

#### Binding antibody responses—MARV GP

At 21 days post-dose 2, 100.0% of participants (42/42) in filovirus regimen groups exhibited binding antibody responses against MARV. The highest GMC at 21 days post-dose 2 was observed after Ad26.Filo, MVA 56d (1,240 EU/mL). In the control group (Ad26.ZEBOV, MVA 56d) at this time point, responses were observed in 18.2% of participants (2/11; GMC: <LLOQ). At Day 360, similar to the EBOV and SUDV GP–specific responses, the magnitude of the binding antibody responses to MARV GP had decreased, yet responses persisted in 78.6–92.3% of participants in filovirus regimen groups, with similar GMCs across the groups (53–81 EU/mL). Responses did not persist at Day 360 in the control group.

For the MVA, Ad26.Filo 14d plus Ad26.Filo booster regimen, 100.0% (5/5) of participants exhibited a strong increase in binding antibody responses seven days post-booster vaccination, with a ~3.3-fold increase in GMC (Day 92: 155 EU/mL; Day 99: 510). At day 360, binding antibody responses were observed in 100.0% (4/4) of participants with a GMC of 133 EU/mL, similar to participants who did not receive a booster vaccination.

#### Neutralizing antibody responses

**Fig 3:**
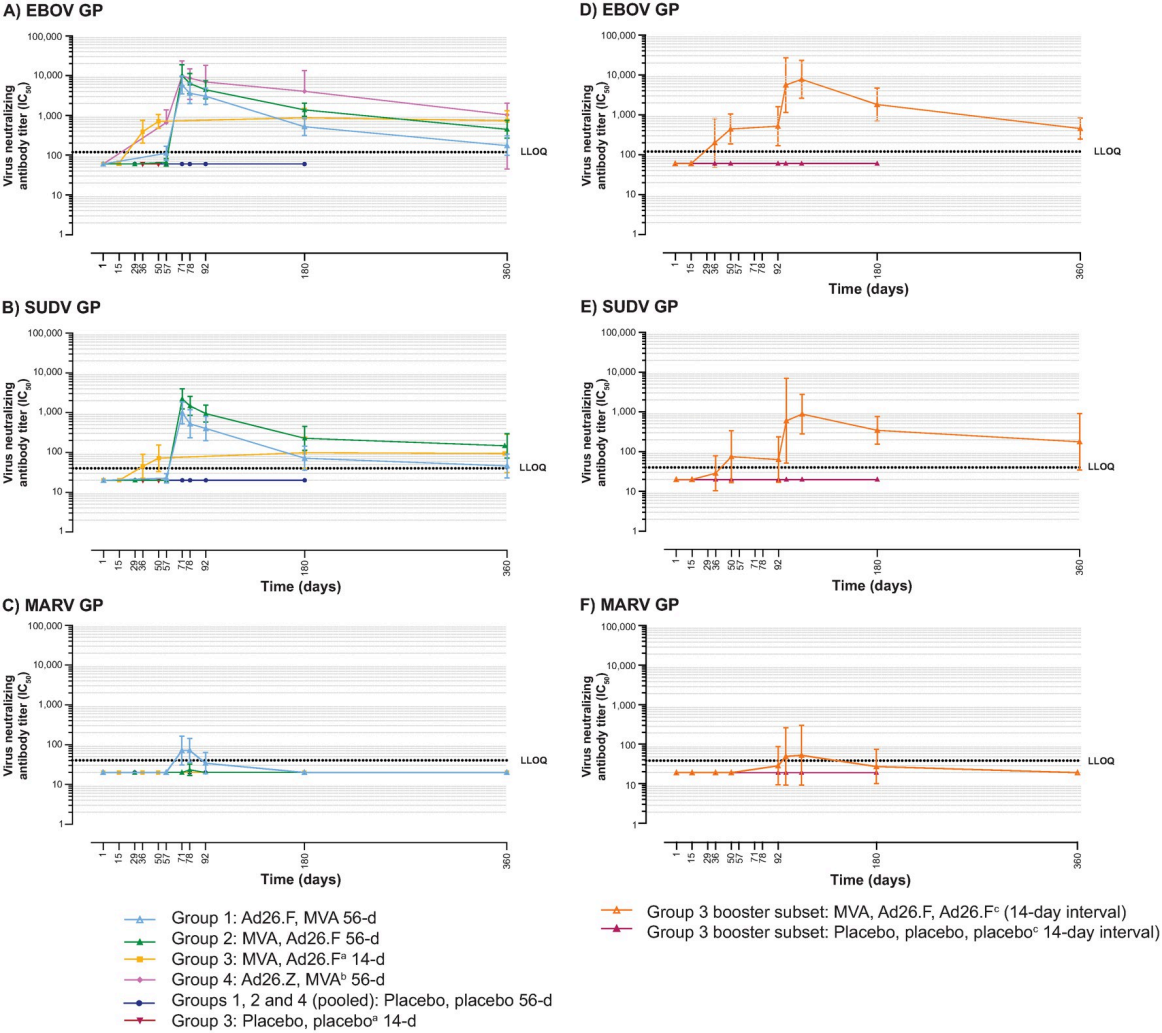
GP-specific neutralizing antibody responses (psVNA); immunogenicity analysis set. Ad26.F = Ad26.Filo; Ad26.Z = Ad26.ZEBOV; EBOV = *Zaire ebolavirus*; GP = glycoprotein; IC_50_ = 50% inhibitory concentration; Inf U = infectious units; LLOQ = lower limit of quantification; MARV = *Marburg virus*; MVA = MVA-BN-Filo; psVNA = pseudovirion neutralization assay; SUDV = *Sudan ebolavirus*. ^a^Includes all Group 3 participants up to and including the Day 50 time point, after which point only Group 3 participants who did not receive a third dose of vaccine/placebo on Day 92 are included. ^b^MVA-BN-Filo at a dose 1x10^8^ Inf U. ^c^All time points include only Group 3 participants who received three doses of vaccine/placebo.

EBOV, SUDV, and MARV GP-specific neutralizing antibody concentrations are summarized in Tables 9–14 in S1 File, respectively, and depicted in Fig 3. In placebo recipients, neutralizing antibody responses were not observed (<LLOQ) at any time point.

#### Neutralizing antibody responses—EBOV GP

At 21 days post-dose 2, 100.0% of participants in both the 56d interval filovirus regimen groups (28/28) and the control group (Ad26.ZEBOV, MVA 56d; 11/11), as well as 85.7% of participants (12/14) in the MVA, Ad26.Filo 14d group, exhibited responses against EBOV GP. The highest GMT at 21 days post-dose 2 was observed after MVA, Ad26.Filo 56d (6,343 IC_50_ titer) and was comparable to the response observed in the control group (6,192 IC_50_ titer). At Day 360, the magnitude of the neutralizing antibody responses had decreased in the 56d interval filovirus regimen groups compared to the 21 days post-dose 2 time point, yet responses persisted in the majority of participants: 64.3% in the Ad26.Filo, MVA 56d group (9/14, 175 IC_50_ titer) and 92.3% in the MVA, Ad26.Filo 56d group (12/13, 449 IC_50_ titer). In the MVA, Ad26.Filo 14d group, responses at Day 360 persisted in 100.0% of participants (8/8, GMT: 734 IC_50_ titer) at a magnitude similar to that observed at 21 days post-dose 2. Responses persisted at Day 360 in 88.9% of participants (8/9, GMT: 656 IC_50_ titer) in the control group.

For the MVA, Ad26.Filo 14d plus Ad26.Filo booster regimen, a strong increase in neutralizing antibody responses against EBOV GP was observed in 100.0% of participants (5/5) seven days post-booster vaccination, with a ~10.7-fold increase in GMT (Day 92: 520 IC_50_ titer; Day 99: 5,589 IC_50_ titer). Responses against EBOV persisted in 100.0% of participants (5/5, GMT: 453 IC_50_ titer) at Day 360.

#### Neutralizing antibody responses—SUDV GP

At 21 days post-dose 2, responses were induced in 92.9–100% of participants in the 56d interval filovirus regimen groups (Ad26.Filo, MVA 56d: 13/14, GMT: 526 IC_50_ titer; MVA, Ad26.Filo 56d: 14/14, GMT: 1,483 IC_50_ titer) and 35.7% of participants in the MVA, Ad26.Filo 14d group (5/14, GMT: 45 IC_50_ titer). At Day 360, the magnitude of the neutralizing antibody responses against SUDV GP had decreased in the 56d interval groups and increased in the 14d interval group compared to the 21 days post-dose 2 time point. Day 360 responder rates were 35.7% (5/14: 46 IC_50_ titer) in the Ad26.Filo, MVA 56d group and 76.9% (10/13: 147 IC_50_ titer) in the MVA, Ad26.Filo 56d group, versus 62.5% (5/8, GMT: 95 IC_50_ titer) in the MVA, Ad26.Filo 14d group. SUDV GP-specific neutralizing antibody responses were not assessed at any time point in the control group (Ad26.ZEBOV, MVA 56d).

For the MVA, Ad26.Filo 14d plus Ad26.Filo booster regimen, a strong increase in neutralizing antibody responses against SUDV GP was observed in 80.0% of participants (4/5) seven days post-booster, with a ~9.5-fold increase in GMT (Day 92: 63 IC_50_ titer; Day 99: 601 IC_50_ titer). On Day 360, responses against SUDV GP persisted in 80.0% of participants (4/5, GMT: 178 IC_50_ titer).

#### Neutralizing antibody responses—MARV GP

MARV GP-specific neutralizing antibody responses were only observed 21-days post-dose 2. These responses were induced in 57.1% (8/14, GMT: 72 IC_50_ titer) of participants following Ad26.Filo, MVA 56d and in 7.1% of participants (1/14, GMT: <LLOQ) following MVA, Ad26.Filo 56d. These responses did not persist until Day 360 and were not observed at any other time point or in any other vaccination regimen.

For the MVA, Ad26.Filo 14d plus Ad26.Filo booster regimen, the MARV GP neutralizing antibody responder rate (40.0%, 2/5) and GMT (51 IC_50_ titer) had increased at seven days post-booster as compared to the pre-booster time point (20.0% [1/5] and <LLOQ, respectively). However, these responses did not persist at Day 360.

#### Cellular immune responses

EBOV, SUDV, and MARV GP-specific CD4+ and CD8+ T cell responses were observed in the majority of participants vaccinated with each of the three filovirus regimens (Figs 2–7 in S1 File). Seven days post-dose 2, CD4+ T cell response rates for these three regimens were generally similar against EBOV (21.4–42.9%) and SUDV (21.4–78.6%) GPs, but tended to be lower against MARV GP (7.1–14.3%). At the same time point, CD8+ T cell response rates were 7.1–53.3% against EBOV, 21.4–64.3% against SUDV, and 21.4–66.7% against MARV GPs. In general, CD8+ T cell responses were more durable than CD4+ T cell responses. A full description of T cell responses can be found in the Results in S1 File.

## Discussion

In this Phase 1, randomized, double-blind, placebo-controlled, FIH study, several multivalent two-dose vaccine regimens comprising Ad26.Filo and MVA were assessed. Ad26.Filo is a trivalent combination (1:1:1) of three vaccines, Ad26.ZEBOV, Ad26.SUDV, and Ad26.MARVA, encoding the GPs of EBOV, SUDV, and MARV, respectively. MVA is a multivalent vaccine encoding the EBOV, SUDV, and MARV GPs and the TAFV NP. The results of this study support that an Ad26.Filo, MVA vaccine regimen in a 56d interval, and an MVA, Ad26.Filo vaccine regimen in either a 56d or 14d interval, are well tolerated and immunogenic in healthy adults. There were no deaths or discontinuations from the study vaccination due to an AE, and no SAEs were considered related to a study vaccine. Overall, solicited AEs were more frequent in active vaccine recipients than in placebo recipients, and with higher doses of MVA (5x10^8^ Inf U compared with 1x10^8^ Inf U), while most Grade 3 solicited systemic AEs were reported following vaccination with Ad26.Filo.

All filovirus vaccine regimens induced humoral immune responses to EBOV, SUDV, and MARV GPs. In general, MVA, Ad26.Filo 56d induced higher binding antibody responses against the three different filovirus GPs than the 14d interval. When measured after administration of dose 1 but prior to administration of dose 2, vaccination with Ad26.Filo as dose 1 generally induced higher antibody responses against EBOV, SUDV, and MARV GPs as compared to a first vaccination with MVA. The magnitude of the antibody responses increased after the second vaccination and 100.0% of participants in all three filovirus regimen groups had a binding antibody response against EBOV, SUDV, and MARV GPs. At the 21 days post-dose 2 time point, the binding antibody response rate against EBOV GP was similar between the vaccine regimens with a 56d interval, irrespective of the vaccination order. However, the magnitude of response was higher for MVA, Ad26.Filo 56d than for Ad26.Filo, MVA, 56d. At the same time point, binding antibody concentrations within the Ad26.Filo, MVA 56d interval group tended to be lower against SUDV GP and numerically higher against MARV GP in comparison to the MVA, Ad26.Filo 56d interval. Further clinical evaluation is needed to elucidate whether this numerical difference in antibody response magnitude is likely linked to the vaccination order, or rather a result of the small group sizes in this Phase 1 study. The responses against EBOV, SUDV, and MARV GPs persisted at least one year post-dose 1 vaccination, the last time point assessed in this study, with similar antibody concentrations observed on Day 360 between the different filovirus regimens, independent of the vaccine order and interval.

As with the binding antibody responses, the neutralizing antibody responses against EBOV and SUDV GPs were higher after vaccination with the MVA, Ad26.Filo regimen in a 56d interval compared to the 14d interval. Twenty-one days post-dose 2, EBOV and SUDV GP neutralizing antibody levels tended to be higher after vaccination with the MVA, Ad26.Filo regimen than with the Ad26.Filo, MVA regimen in a 56d interval. It is currently unclear why all participants vaccinated with each of the three filovirus vaccine regimens exhibited binding antibody responses against the MARV Ci67 GP, but neutralizing antibody responses against the MARV Angola GP were only observed in one of 29 participants vaccinated with MVA, Ad26.Filo in either the 14d or 56d interval, and eight of 14 participants vaccinated with the Ad26.Filo, MVA 56d regimen. It should be noted that the MARV GP and SUDV GP-specific neutralization assays were developed and qualified using non-human primate serum, and these assays may need further development with human serum to optimize the assay specificity and/or detection range.

Cellular immune responses were also evaluated after vaccination with the filovirus regimens, and EBOV, SUDV, and MARV GP-specific CD4+ and CD8+ T cell responses were observed in a substantial percentage of participants vaccinated with each of the three filovirus regimens. When measured after dose 1 but before dose 2, cellular responses were detectable in all vaccine regimens, but the frequency of responses was lower after receipt of MVA as dose 1. The sequence of Ad26.Filo, MVA appeared to generate more CD4+ and CD8+ T cell responses than the MVA, Ad26.Filo sequence of vaccine administration.

The Ad26.ZEBOV, MVA Ebola vaccine regimen was included here as a study control, and the humoral and cellular immune responses induced by this regimen against EBOV GP were consistent with those previously reported in monovalent Ebola vaccine Phase 1 clinical studies [17, 19, 20, 24–26]. The SUDV and MARV GP-specific humoral and cellular immune responses were also analyzed after vaccination with Ad26.ZEBOV, MVA to evaluate whether any immune responses to SUDV and MARV were induced by the Ad26.ZEBOV vaccine through cross-reactivity of EBOV GP-specific responses and/or by the multivalent MVA vaccine. When assessed after receipt of Ad26.ZEBOV as dose 1, but prior to receipt of MVA as dose 2, SUDV GP-specific binding antibodies (but not MARV GP-specific binding antibodies) were induced in a small percentage of participants. The observation of cross-reactivity against SUDV GP, but not MARV GP, after administration of Ad26.ZEBOV is not unexpected due to the higher degree of evolutionary relatedness between the two Ebola species strains (EBOV and SUDV) versus MARV. SUDV GP and MARV GP-specific neutralizing antibody responses and cellular immune responses were not observed after first vaccination with Ad26.ZEBOV. After receipt of the Ad26.ZEBOV, MVA vaccine regimen, SUDV GP-specific binding antibodies were observed in all participants, while MARV GP-specific binding antibodies were only observed in a minor percentage. SUDV GP and MARV GP-specific neutralizing antibody responses were not observed after Ad26.ZEBOV, MVA vaccination. SUDV GP-specific CD4+ and CD8+ T cells were observed in a substantial percentage of participants vaccinated with Ad26.ZEBOV, MVA. Vaccination with Ad26.ZEBOV, MVA did not induce MARV GP-specific CD4+ T cell responses at the evaluated time points, and MARV GP-specific CD8+ T cell responses were observed in one participant. This small Phase 1 study only evaluated whether any SUDV and MARV GP cross-reactive immune responses were induced by vaccination with the Ebola vaccine regimen Ad26.ZEBOV, MVA. Therefore, no inference can be assumed as to possible protection against SUDV and MARV.

Previous studies with the monovalent Ebola vaccine regimen Ad26.ZEBOV, MVA demonstrated that a booster vaccination one or two years after dose 1 vaccination induces a strong and rapid anamnestic response [16, 25]. Here, we included the MVA, Ad26.Filo 14d plus Ad26.Filo booster regimen to evaluate whether a booster vaccination with Ad26.Filo administered at approximately three months post-dose 1 (2.5 months post-dose 2) would be capable of inducing a similar strong and rapid increase in immune responses. A robust increase in humoral immune responses to EBOV, SUDV, and MARV GPs was observed within seven days, with approximate 5.5-, 3.3- and 5.2-fold increases in binding antibody GMC and 10.7-, 1.3-, and 9.5-fold increases in neutralizing antibody GMT against EBOV, SUDV, and MARV GPs, respectively, compared to the pre-booster time point. These responses were, in general, slightly further increased at 21 days post-booster and decreased thereafter. At Day 360, approximately nine months post-booster, the level of binding and neutralizing antibody responses was similar to the level observed pre-booster and also similar to the level observed at Day 360 in participants vaccinated with the same MVA, Ad26.Filo primary regimen in a 14d interval. These data, although limited to five participants, indicate that a booster vaccination given as early as 2.5 months after the last dose of the regimen is able to substantially increase the humoral immune responses to EBOV, SUDV, and MARV GPs within seven days. Although this short time period of seven days is indicative of an anamnestic response, it is most likely that the increase in antibody concentrations observed post-booster vaccination is the product of a combination of memory responses stimulated by the booster vaccination and ongoing germinal center reactions that originated after the second vaccination, benefiting from a new influx of antigen. In the clinical studies that evaluated a booster vaccination with Ad26.ZEBOV approximately one or two years after the primary Ebola vaccine regimen Ad26.ZEBOV, MVA, the level of persisting antibodies observed at one year post-booster was higher compared to the pre-booster time point, an observation that was not made here at nine months post-booster. It is possible that the timing of the booster vaccination in this study, only 2.5 months after the second dose, allowed for a rapid and strong increase in circulating antibody levels produced by short-lived plasma cells, but was not optimal for inducing an increase in the level of persisting circulating antibodies, generally produced by long-lived plasma cells.

The majority of EBOV, SUDV, and MARV candidate vaccines in the advanced stages of clinical development are monovalent, although two bivalent vaccines (rAd5 and cAd3-EBO) encoding the GP from EBOV and SUDV have completed Phase 1 clinical trials [27, 28]. The first human trial with an rAd5-based Ebola vaccine was a randomized, double-blind, placebo-controlled, dose-escalating, Phase 1 trial [28]. The bivalent vaccine, encoding the GPs of EBOV and SUDV, was demonstrated to be safe and well tolerated at both a low and a high dose, with the most common AEs being mild headaches of short duration. Additionally, both humoral and cellular immune responses were elicited in a dose-dependent manner, with humoral immune responses increasing in both frequency and magnitude and cellular immune responses increasing in frequency at the higher dose level. However, preexisting immunity to human Ad5 resulted in decreased antibody responses to the EBOV and SUDV GPs. Therefore, the known high degree of baseline seropositivity against human Ad5 was a critical concern for this Ad5 vector-based vaccine, as preexisting immunity could decrease the magnitude and/or frequency of immune responses against the EBOV and SUDV GPs. To address this issue, a bivalent recombinant chimpanzee adenovirus type 3-vectored vaccine expressing the GPs of EBOV and SUDV (cAd3-EBO) was developed and evaluated in a Phase 1, dose-escalation, open-label trial to evaluate safety and immunogenicity [27]. As with the rAd5-based bivalent Ebola vaccine, the bivalent cAd3-EBO was also found to be safe and well tolerated at both a low and a high dose, with fever as the most common AE (reported only in the high dose group). A dose-dependent effect was observed in regard to both reactogenicity and immunogenicity. In the high-dose group, participants reported increased use of medication for symptom relief, as well as lowered body temperature. Humoral immune responses increased in magnitude and cellular immune responses increased in frequency at the higher dose level.

The results from this FIH study support that multivalent two-dose vaccine regimens consisting of Ad26.Filo and MVA are immunogenic against all three tested antigens (EBOV, SUDV, and MARV). Each of these filoviruses is known to cause severe hemorrhagic fever in humans [29]. Compared with monovalent vaccine regimens, a multivalent vaccine regimen could reduce the number of vaccinations required to provide protection against more than one filovirus, which could in turn reduce health service costs [30, 31]. Given that EBOV, SUDV, and MARV are each responsible for at least one major outbreak (>200 cases) since 1976 [1, 2], a prophylactic multivalent filovirus vaccine regimen could offer a lifesaving preventative tool for communities in Africa.

### Limitations

This study was not intended to provide statistically confirmed conclusions regarding safety and immunogenicity of the various two-dose regimens; the study was conducted to provide a preliminary insight into the safety and immunogenicity of regimens involving Ad26.Filo and MVA. No inference as to possible protection against EBOV, SUDV, and MARV can be made. Furthermore, due to the nature of this Phase 1 study, small group sizes warrant caution when interpreting trends, particularly for the small subset of participants who received Ad26.Filo booster vaccination in Group 3.

## Conclusions

The multivalent two-dose heterologous vaccine regimens of Ad26.Filo and MVA investigated in this study were found to be well tolerated and immunogenic in healthy adults, irrespective of vaccine order or interval; immune responses against all three tested filovirus antigens were induced. Administration of Ad26.Filo as dose 1 in the regimen generally induced higher binding antibody responses against EBOV, SUDV, and MARV GPs, and higher neutralizing antibody responses against both EBOV and SUDV GPs, as compared to a first vaccination with MVA. A booster vaccination with Ad26.Filo administered on Day 92 induced a strong increase in humoral immune responses to EBOV, SUDV, and MARV GPs within seven days.

## Supporting information

S1 File (DOCX)

S1 Protocol (PDF)

S1 Checklist CONSORT 2010 checklist of information to include when reporting a randomised trial*. (DOC)

## References

[1] World Health Organization. Ebola virus disease. [Cited 15 July 2021]. https://www.who.int/news-room/fact-sheets/detail/ebola-virus-disease.

[2] World Health Organization. Marburg virus disease. [Cited 15 July 2021]. https://www.who.int/news-room/fact-sheets/detail/marburg-virus-disease.

[3] Report of an International Commission. Ebola haemorrhagic fever in Zaire, 1976. Bull World Health Organ. 1978;56: 271–293. 307456 PMC2395567

[4] Martini GA, Siegert R, editors. Marburg virus disease. Springer, Berlin, Heidelberg; 1971.

[5] World Health Organization. Ebola virus disease. [Cited 15 July 2021]. https://www.who.int/csr/disease/ebola/en.

[6] Centers for Disease Control and Prevention. 2014–2016 Ebola outbreak in West Africa. [Cited 15 July 2021]. https://www.cdc.gov/vhf/ebola/history/2014-2016-outbreak/index.html.

[7] World Health Organization. Ebola outbreak–Democratic Republic of the Congo: North Kivu, Ituri, 2018–2020. [Cited 24 August 2021]. https://www.who.int/emergencies/situations/Ebola-2019-drc-.

[8] US Food and Drug Administration. First FDA-approved vaccine for the prevention of Ebola virus disease, marking a critical milestone in public health preparedness and response. [Cited 15 July 2021]. https://www.fda.gov/news-events/press-announcements/first-fda-approved-vaccine-prevention-ebola-virus-disease-marking-critical-milestone-public-health.

[9] European Medicines Agency. First vaccine to protect against Ebola. [Cited 15 July 2021]. https://www.ema.europa.eu/en/news/first-vaccine-protect-against-ebola.

[10] European Commission. Vaccine against Ebola: commission grants new market authorisations. [Cited 15 July 2021]. https://ec.europa.eu/commission/presscorner/detail/en/IP_20_1248.

[11] European Medicines Agency. European public assessment report: Zabdeno. [Cited 15 July 2021]. https://www.ema.europa.eu/en/medicines/human/EPAR/zabdeno.

[12] European Medicines Agency. European public assessment report: Mvabea. [Cited 15 July 2021]. https://www.ema.europa.eu/en/medicines/human/EPAR/mvabea.

[13] World Health Organization. Ebola/Marburg research and development (R&D) roadmap. 2018. [Cited 15 July 2021]. https://www.who.int/blueprint/priority-diseases/key-action/Ebola-Marburg_Draft_Roadmap_publiccomment_MAY2018.pdf?ua=1.

[14] World Health Organization. Prioritizing diseases for research and development in emergency contexts. [Cited 15 July 2021]. https://www.who.int/blueprint/priority-diseases/en.

[15] Callendret B, Vellinga J, Wunderlich K, Rodriguez A, Steigerwald R, Dirmeier U, et al. A prophylactic multivalent vaccine against different filovirus species is immunogenic and provides protection from lethal infections with Ebolavirus and Marburgvirus species in non-human primates. PLoS One. 2018;13: e0192312. doi: 10.1371/journal.pone.0192312 29462200 PMC5819775

[16] Goldstein N, Bockstal V, Bart S, Luhn K, Robinson C, Gaddah A, et al. Safety and immunogenicity of heterologous and homologous 2-dose regimens of adenovirus serotype 26- and modified vaccinia Ankara-vectored Ebola vaccines: a randomized, controlled phase 1 study. J Infect Dis. 2022;226: 595–607. doi: 10.1093/infdis/jiaa586 32939546 PMC9441209

[17] Milligan ID, Gibani MM, Sewell R, Clutterbuck EA, Campbell D, Plested E, et al. Safety and immunogenicity of novel adenovirus type 26– and modified vaccinia Ankara–vectored Ebola vaccines: a randomized clinical trial. JAMA. 2016;315: 1610–1623. doi: 10.1001/jama.2016.4218 27092831

[18] US Food and Drug Administration. Guidance for industry: toxicity grading scale for healthy adult and adolescent volunteers enrolled in preventive vaccine clinical trials. September 2007. https://www.fda.gov/media/73679/download.

[19] Mutua G, Anzala O, Luhn K, Robinson C, Bockstal V, Anumendem D, et al. Safety and immunogenicity of a 2-dose heterologous vaccine regimen with Ad26.ZEBOV and MVA-BN-Filo Ebola vaccines: 12-month data from a phase 1 randomized clinical trial in Nairobi, Kenya. J Infect Dis. 2019;220: 57–67. doi: 10.1093/infdis/jiz071 30796816 PMC6548899

[20] Anywaine Z, Whitworth H, Kaleebu P, Praygod G, Shukarev G, Manno D, et al. Safety and immunogenicity of a 2-dose heterologous vaccination regimen with Ad26.ZEBOV and MVA-BN-Filo Ebola vaccines: 12-month data from a phase 1 randomized clinical trial in Uganda and Tanzania. J Infect Dis. 2019;220: 46–56. doi: 10.1093/infdis/jiz070 30796818 PMC6548900

[21] Logue J, Tuznik K, Follmann D, Grandits G, Marchand J, Reilly C, et al. Use of the Filovirus Animal Non-Clinical Group (FANG) Ebola virus immuno-assay requires fewer study participants to power a study than the Alpha Diagnostic International assay. J Virol Methods. 2018;255: 84–90. doi: 10.1016/j.jviromet.2018.02.018 29481881 PMC5942582

[22] Frahm N, DeCamp AC, Friedrich DP, Carter DK, Defawe OD, Kublin JG, et al. Human adenovirus-specific T cells modulate HIV-specific T cell responses to an Ad5-vectored HIV-1 vaccine. J Clin Invest. 2012;122: 359–367. doi: 10.1172/JCI60202 22201684 PMC3248307

[23] US Food and Drug Administration. Code of Federal Regulations Title 21. 21CFR312.21.

[24] Winslow RL, Milligan ID, Voysey M, Luhn K, Shukarev G, Douoguih M, et al. Immune responses to novel adenovirus type 26 and modified vaccinia virus Ankara–vectored Ebola vaccines at 1 year. JAMA. 2017;317: 1075–1077. doi: 10.1001/jama.2016.20644 28291882

[25] Ishola D, Manno D, Afolabi MO, Keshinro B, Bockstal V, Rogers B, et al. Safety and long-term immunogenicity of the two-dose Ad26.ZEBOV and MVA-BN-Filo Ebola vaccine regimen in adults in Sierra Leone: a combined open-label, non-randomised stage 1, and a randomised, double-blind, controlled stage 2 trial. Lancet Infect Dis. 2022;22: 97–109. doi: 10.1016/S1473-3099(21)00125-0 34529963 PMC7613326

[26] Afolabi M, Ishola D, Manno D, Keshinro B, Bockstal V, Rogers B, et al. Safety and immunogenicity of the two-dose heterologous Ad26.ZEBOV and MVA-BN-Filo Ebola vaccine regimen in children in Sierra Leone: a randomised, double-blind, controlled trial. Lancet Infect Dis. 2022;22: 110–122. doi: 10.1016/S1473-3099(21)00128-6 34529962 PMC7613317

[27] Ledgerwood JE, DeZure AD, Stanley DA, Coates EE, Novik L, Enama ME, et al. Chimpanzee adenovirus vector Ebola vaccine. N Engl J Med. 2017;376: 928–938. doi: 10.1056/NEJMoa1410863 25426834

[28] Ledgerwood JE, Costner P, Desai N, Holman L, Enama ME, Yamshchikov G, et al. A replication defective recombinant Ad5 vaccine expressing Ebola virus GP is safe and immunogenic in healthy adults. Vaccine. 2010;29: 304–313. doi: 10.1016/j.vaccine.2010.10.037 21034824

[29] Changula K, Kajihara M, Mweene AS, Takada A. Ebola and Marburg virus diseases in Africa: increased risk of outbreaks in previously unaffected areas? Microbiol Immunol. 2014;58: 483–491. doi: 10.1111/1348-0421.12181 25040642

[30] Halsey NA. Safety of combination vaccines: perception versus reality. Pediatr Infect Dis J. 2001;20: S40–S44. doi: 10.1097/00006454-200111001-00007 11704723

[31] Lauer KB, Borrow R, Blanchard TJ. Multivalent and multipathogen viral vector vaccines. Clin Vaccine Immunol. 2017;24: e00298–16. doi: 10.1128/CVI.00298-16 27535837 PMC5216423

