# Supplementary material for: First-in-human study to evaluate safety, tolerability, and immunogenicity of heterologous regimens using the multivalent filovirus vaccines Ad26.Filo and MVA-BN-Filo administered in different sequences and schedules: A randomized, controlled study
Source: PLoS One. 2022 Oct 5;17(10):e0274906. doi: 10.1371/journal.pone.0274906 (PMC9534391; doi:10.1371/journal.pone.0274906)
Supplement: S1 File — (DOCX) [file pone.0274906.s001.docx]

# Supporting information

**Methods. Intracellular Cytokine Staining (ICS) Assay for detection of IL-2, IFN-γ, and TNF-α.**

**Results. T cell responses measured by ICS detection of IL-2, IFN-γ, and TNF-α.**

**Table 1. Intracellular cytokine staining (ICS) staining panel.**

**Table 2. Clinical laboratory abnormalities following study vaccination by dose; full analysis set.**

**Table 3. EBOV GP-specific binding antibody responses (ELISA): descriptive statistics and responder rates; immunogenicity analysis set.**

**Table 4. EBOV GP-specific binding antibody responses (ELISA): descriptive statistics and responder rates; immunogenicity analysis set; Group 3 booster subset.**

**Table 5. SUDV GP-specific binding antibody responses (ELISA): descriptive statistics and responder rates; immunogenicity analysis set.**

**Table 6. SUDV GP-specific binding antibody responses (ELISA): descriptive statistics and responder rates; immunogenicity analysis set; Group 3 booster subset.**

**Table 7. MARV GP-specific binding antibody responses (ELISA): descriptive statistics and responder rates; immunogenicity analysis set.**

**Table 8. MARV GP-specific binding antibody responses (ELISA): descriptive statistics and responder rates; immunogenicity analysis set; Group 3 booster subset.**

**Table 9. EBOV GP-specific neutralizing antibody responses (psVNA): descriptive statistics and responder rates; immunogenicity analysis set.**

**Table 10. EBOV GP-specific neutralizing antibody responses (psVNA): descriptive statistics and responder rates; immunogenicity analysis set; Group 3 booster subset.**

**Table 11. SUDV GP-specific neutralizing antibody responses (psVNA): descriptive statistics and responder rates; immunogenicity analysis set.**

**Table 12. SUDV GP-specific neutralizing antibody responses (psVNA): descriptive statistics and responder rates; immunogenicity analysis set; Group 3 booster subset.**

**Table 13. MARV GP-specific neutralizing antibody responses (psVNA): descriptive statistics and responder rates; immunogenicity analysis set.**

**Table 14. MARV GP-specific neutralizing antibody responses (psVNA): descriptive statistics and responder rates; immunogenicity analysis set; Group 3 booster subset.**

**Fig 1. Study design.**

**Fig 2. CD4+ T cell cytokine responses (ICS): EBOV GP.**

**Fig 3. CD8+ T cell cytokine responses (ICS): EBOV GP.**

**Fig 4. CD4+ T cell cytokine responses (ICS): SUDV GP.**

**Fig 5. CD8+ T cell cytokine responses (ICS): SUDV GP.**

**Fig 6. CD4+ T cell cytokine responses (ICS): MARV GP.**

**Fig 7. CD8+ T cell cytokine responses (ICS): MARV GP.**

## Methods: Intracellular Cytokine Staining (ICS) Assay for detection of IL-2, IFN-γ, and TNF-α

Peripheral blood mononuclear cells (PBMCs) for cellular assays were isolated and cryopreserved from whole blood collected at baseline and multiple time points following vaccination. Flow cytometry was used to examine antigen-specific CD4+ and CD8+ T cell responses using a validated ICS assay for detection of IL-2, IFN-γ, and TNF-α. The assay was similar to a published report, and the details of the staining panel are included in **Table** **1 in S1 Supporting information** [1].

Previously cryopreserved PBMCs were stimulated for six hours with peptide pools covering the glycoproteins (GPs) encoded in the vaccines. These pools consisted of 15-mer peptides overlapping by 11 amino acids. There was a total of six peptide pools, two each for the three viruses encoded in the vaccines: Ebola virus Mayinga variant (EBOV GP1 and GP2), Sudan virus Gulu variant (SEBOV GP1 and GP2), and Marburg virus Angola variant (MARV GP1 and GP2. The EBOV peptides were synthesized by Think Peptides (Oxford, UK), and the other peptides were synthesized by JPT Technologies.

As a negative control, cells were not stimulated. As a positive control, cells were stimulated with a polyclonal stimulant, staphylococcal enterotoxin B. Two replicates were tested for the negative control. CD4+ and CD8+ T cells were the primary immunogenicity readouts. Since there are two pools for each of the three viral GPs, the total response for each GP was the sum of the response to the GP1 and GP2 pools for each virus.

Several criteria were used to determine if assay data were acceptable and could be statistically analyzed. The blood draw date must have been within the allowable visit window as determined by the protocol. On the second day after sample thawing, the viability must have been 66% or greater. If not, the sample for that specimen at that time point was retested. If upon retesting the viability remained below this threshold, the ICS assay was not performed and no data were reported to the statistical center for the time point. For the negative control acceptance criteria, if the average cytokine response for the negative control wells was above 0.1% for either the CD4+ or CD8+ T cells, then the sample was retested and the response for the retested sample was analyzed. Furthermore, the total number of CD4+ and CD8+ T cells must have exceeded certain thresholds. If the number of CD4+ or CD8+ T cells was less than 5,000 for any of the peptide pools or one of the negative control replicates for a particular sample, data for that stimulation were filtered. If both negative control replicates were <5,000 cells, the sample was retested. If upon retesting, one negative control replicate was <5,000, the negative control replicate with >5,000 cells was used. If both negative control replicates from the retest for a T cell subset were <5,000, then data for the T cell subset were not included in the analysis.

Sample positivity (interpretation) was determined using a one-sided Fisher's exact test [2]. A multiplicity adjustment was made across all tested peptide pools using the discrete Bonferroni adjustment method. If the adjusted p-value for an antigen was ≤0.00001, the response to the peptide for the T cell subset was considered positive. A participant was a responder at a considered time point if sample interpretation was negative at baseline but positive post-baseline and the post-baseline value was greater than 2x Sponsor-defined assay-specific threshold (0.04%), or sample interpretation was positive at both baseline and post-baseline and there was a greater than two-fold increase from baseline in background adjusted total cytokine response. Values below the assay-specific threshold were imputed with threshold/2 (0.02%). For the calculation of fold increases, values below the assay-specific threshold were imputed with the threshold.

**Supporting information references**

1. Moncunill G, Dobaño C, McElrath MJ, and De Rosa SC. OMIP-025: evaluation of human T- and NK-cell responses including memory and follicular helper phenotype by intracellular cytokine staining. Cytometry A*.* 2015;87: 289–292. doi: 10.1002/cyto.a.22590.

Horton H, Thomas EP, Stucky JA, Frank I, Moodie Z, Huang Y, et al. Optimization and validation of an 8-color intracellular cytokine staining (ICS) assay to quantify antigen-specific T cells induced by vaccination. J Immunol Methods*.* 2007;323: 39–54. doi: 10.1016/j.jim.2007.03.002.

## Results: T cell responses measured by ICS detection of IL-2, IFN-γ, and TNF-α

EBOV, SUDV, and MARV GP-specific CD4+ and CD8+ T cell responses are summarized in **Figs 2–7 in S1 Supporting Information.** In placebo recipients, CD4+ and CD8+ T cell responses were not observed (<sponsor-defined assay-specific threshold) at any time point (panels **E, F,** and **H** in **Figs 2–7 in S1 Supporting Information**).

*EBOV GP*

After a first vaccination with MVA-BN-Filo (MVA), CD4+ T cell responses against EBOV GP were observed in 7.1% (1/14; median: <threshold) of participants on Day 15, but not on Day 57. Vaccination with Ad26.Filo induced CD4+ T cell responses in 7.7% (1/13; median: <threshold) of participants on Day 57. In the control group, Ad26.ZEBOV vaccination induced CD4+ T cell responses in 30.0% (3/10; median: <threshold) of participants on Day 57. Seven days post-dose 2, the highest CD4+ T cell responder rate in Groups 1–3 was observed for the MVA, Ad26.Filo 14d interval group (42.9% [6/14]), and the lowest was observed for the MVA, Ad26.Filo 56d interval group (21.4% [3/14]), with median response levels ranging from <threshold–0.19% (**Fig 2 in S1 Supporting Information**). At Day 360, responses persisted in 14.3–25.0% of participants, with median response levels of <threshold–0.05%. In the control group (Ad26.ZEBOV, MVA 56d), CD4+ T cell responses were observed in 55.6% (5/9; median: 0.06%) of participants seven days post-dose 2, which had decreased to 28.6% (2/7; median: 0.05%) on Day 360. In the MVA, Ad26.Filo 14d plus Ad26.Filo booster group, on Day 99 (seven days post-booster vaccination), CD4+ T cell responses against EBOV GP were not observed, but had increased to 20% (1/5; median: 0.07%) by Day 360.

Seven days post-dose 2, the highest CD8+ T cell responder rate in Groups 1–3 was observed following Ad26.Filo, MVA 56d (53.3%, 8/15), and the lowest was observed following MVA, Ad26.Filo 56d (7.1%, 1/14) (**Fig 3 in S1 Supporting Information**), with median response levels of <threshold–0.07%. At Day 360, responses persisted in 38.5–75.0% of participants, with median response levels of 0.05-0.10%. In the control group, 62.5% (5/8; median: 0.12%) of participants exhibited CD8+ T cell responses at seven days post-dose 2, which had increased to 85.7% (6/7; median: 0.34%) on Day 360. In the MVA, Ad26.Filo 14d plus Ad26.Filo booster subset, on Day 99 (seven days post-booster vaccination), CD8+ T cell responses against EBOV GP were observed in 80.0% of participants (4/5; median: 0.51%), as compared to 75.0% (3/4; median: 0.26%) at the pre-booster time point. On Day 360, responder levels persisted at 80% (4/5), but the median response level had decreased (0.40%).

*SUDV GP*

Seven days post-dose 2, the highest CD4+ T cell responder rate in Groups 1–3 was observed for the MVA, Ad26.Filo 14d group (78.6% [11/14]) and the lowest was observed for MVA, Ad26.Filo 56d (21.4% [3/14]), with median response levels of 0.07–0.23% (**Fig 4 in S1 Supporting Information**). At Day 360, responses persisted in 21.4–75.0% of participants, with median response levels of <threshold–0.11%. In the control group (Ad26.ZEBOV, MVA 56d), CD4+ T cell responses were observed in 44.4% of participants (4/9; median: 0.04%) at 21 days post-dose 2; this response did not persist at Day 360. In the MVA, Ad26.Filo 14d plus Ad26.Filo booster group, on Day 99 (seven days post-booster vaccination), CD4+ T cell responses were observed in 60% (3/5; median: 0.29%) of participants, as compared to 50% (2/4; median: 0.09%) at the pre-booster time point. On Day 360, responder rates had remained constant as compared to seven days post-booster, but the median response had declined to 0.16%.

Seven days post-dose 2, the highest CD8+ T cell responder rate in Groups 1–3 was observed for MVA, Ad26 14d (64.3% [9/14]) and the lowest was observed for MVA, Ad26.Filo 56d (21.4% [3/14]), with median response levels ranging from 0.04–0.15% (**Fig 5 in S1 Supporting Information**). At Day 360, responses persisted in 50.0–75.0% of participants, with median response levels of 0.05–0.13%. In the control group (Ad26.ZEBOV, MVA 56d), CD8+ T cell responses were observed in 50.0% of participants (4/8; median: <threshold) 21 days post-dose 2, and 42.9% of participants (3/7; median: <threshold) on Day 360.

In the MVA, Ad26.Filo 14d plus Ad26.Filo booster group, on Day 99 (seven days post-booster vaccination), CD8+ T cell responses were observed in 100.0% (5/5; median: 0.38%) of participants, similar to the pre-booster time point (100.0% [4/4]; median: 0.35%); **Fig 5G in S1 Supporting Information**). On Day 360, the responder rates had persisted (100.0% [5/5]), but the median response had declined to 0.26%.

*MARV GP*

Seven days post-dose 2, the highest CD4+ T cell responder rate in Groups 1–3 was observed for both the 56d interval regimens (14.3% [2/14]), while the lowest was observed for MVA, Ad26.Filo 14d (7.1% [1/14]); median response levels were all <threshold (**Fig 6 in S1 Supporting Information**). At Day 360, responses persisted in 15.4% (2/13; median: <threshold) and 7.1% (1/14; median: <threshold) of participants who received the MVA, Ad26.Filo and Ad26.Filo, MVA regimens in a 56d interval, respectively, but responses were not observed in participants who received MVA, Ad26.Filo 14d. MARV GP-specific CD4+ T cell responses were not observed in the control group (Ad26.ZEBOV, MVA 56d). In the MVA, Ad26.Filo 14d plus Ad26.Filo booster subset, CD4+ T cell responses against MARV GP were not observed on Day 92 or on Day 99, but had increased to 20% (1/5; median: 0.06%) by Day 360.

Seven days post-dose 2, the highest CD8+ T cell responder rate in Groups 1–3 was observed following Ad26.Filo, MVA 56d (66.7% [10/15]) and the lowest was observed following MVA, Ad26.Filo 56d (21.4% [3/14]), with median response levels of <threshold–0.14% (**Fig 7 in S1 Supporting Information**). At Day 360, responses persisted in 50.0–76.9% of participants, with median response levels of 0.10–0.36%. In the control group (Ad26.ZEBOV, MVA 56d), MARV GP-specific CD8+ T cell responses were only observed at 21 days post-dose 2 (12.5% of participants [1/8]). In the MVA, Ad26.Filo 14d plus Ad26.Filo booster group, on Day 99 (seven days post-booster vaccination), CD8+ T cell responses against MARV GP were observed in 40.0% of participants (2/5; median: <threshold) as compared to 25.0% (1/4; median: <threshold) at the pre-booster time point. On Day 360, responder rates remained at 40.0% of participants (2/5; median: <threshold).

**Table 1. Intracellular cytokine staining (ICS) staining panel.**

| **Antibody** | **Antibody clone** | **Manufacturer** | **Catalog number** |
| --- | --- | --- | --- |
| AViD^a^ | NA | Molecular Probes/Invitrogen | L34957 |
| CD14 BV510^a^ | M5E2 | BioLegend | 301842 |
| CD56 BV570 | HCD56 | BioLegend | 318330 |
| CD3 BUV737 | UCHT1 | BD Biosciences | 564307 |
| CD4 BUV395 | SK3 | BD Biosciences | 563550 |
| CD8 BV650 | RPA-T8 | BD Biosciences | 563821 |
| CD45RA APC H7 | HI100 | BD Biosciences | 560674 |
| CCR7 BV786 | G043H7 | BioLegend | 353229 |
| CXCR5 PE-Dazzle594 | J252D4 | BioLegend | 356928 |
| PD-1 BV605 | EH12.2H7 | BioLegend | 329924 |
| ICOS BV711 | DX29 | BD Biosciences | 563833 |
| IFNγ V450 | B27 | Becton Dickinson | 560371 |
| IL2 PE | MQ1-17H12 | BD Biosciences | 559334 |
| IL4 PerCP-Cy5.5 | MP4-25D2 | BioLegend | 500822 |
| IL-17a PE-Cy7 | BL168 | BioLegend | 512315 |
| TNFα FITC | Mab11 | eBioscience | 11-7349-82 |
| CD40L APC | TRAP1 | BD Biosciences | 555702 |
| Granzyme B Alx700 | GB11 | BD Biosciences | 560213 |

^a^AVID and CD14 are detected in the same channel.

**Table 2. Clinical laboratory abnormalities following study vaccination by dose; full analysis set.**

|  | **Grade** | **Ad26.Filo** | **MVA** | **MVA^a^** | **Ad26.ZEBOV** | **Placebo** |
| --- | --- | --- | --- | --- | --- | --- |
| **N** |  | 48 | 45 | 11 | 15 | 23 |
| Chemistry, n (%) |  |  |  |  |  |  |
| Alanine aminotransferase | Grade 1 | 0 | 2 (4.4) | 0 | 0 | 2 (8.7) |
| Aspartate aminotransferase | Grade 1 | 0 | 1 (2.2) | 0 | 0 | 0 |
| Urea nitrogen | Grade 1 | 1 (2.1) | 0 | 0 | 0 | 0 |
|  | Grade 2 | 1 (2.1) | 0 | 0 | 0 | 0 |
| Hematology, n (%) |  |  |  |  |  |  |
| Hemoglobin | Grade 1 | 3 (6.3) | 5 (11.1) | 1 (9.1) | 0 | 0 |
|  | Grade 2 | 2 (4.2) | 1 (2.2) | 1 (9.1) | 1 (6.7) | 0 |
| Hemoglobin change from baseline | Grade 1 | 28 (58.3) | 32 (71.1) | 7 (63.6) | 11 (73.3) | 12 (54.5) |
|  | Grade 2 | 3 (6.3) | 0 | 1 (9.1) | 0 | 1 (4.5) |
|  | Grade 3 | 0 | 1 (2.2) | 0 | 0 | 0 |
| Lymphocytes | Grade 1 | 0 | 1 (2.2) | 0 | 0 | 0 |
| Neutrophils, segmented | Grade 1 | 0 | 0 | 1 (9.1) | 2 (13.3) | 0 |
|  | Grade 2 | 2 (4.2) | 1 (2.2) | 1 (9.1) | 1 (6.7) | 0 |
|  | Grade 3 | 1 (2.1) | 0 | 0 | 0 | 0 |
| Platelets | Grade 2 | 0 | 0 | 0 | 1 (6.7) | 0 |
| Prothrombin time | Grade 1 | 0 | 0 | 1 (9.1) | 0 | 0 |
| WBC – decrease | Grade 1 | 0 | 0 | 1 (9.1) | 0 | 0 |
| WBC – increase | Grade 1 | 0 | 1 (2.2) | 0 | 1 (6.7) | 1 (4.3) |
| Urinalysis, n (%) |  |  |  |  |  |  |
| Urine erythrocytes | Grade 1 | 1 (12.5)^b^ | 0^c^ | 0^d^ | 0^e^ | 0^f^ |
|  | Grade 2 | 1 (12.5)^b^ | 0^c^ | 0^d^ | 0^e^ | 0^f^ |
|  | Grade 3 | 1 (12.5)^b^ | 0^c^ | 0^d^ | 0^e^ | 0^f^ |
| Urine protein | Grade 1 | 11 (22.9) | 7 (15.6) | 1 (9.1) | 1 (6.7) | 4 (17.4) |
|  | Grade 2 | 1 (2.1) | 1 (2.2) | 0 | 0 | 3 (13) |

MVA = MVA-BN-Filo; n (%) = number (percentage) of doses with one or more events; N = number of doses; WBC = white blood cell.

^a^MVA-BN-Filo at a dose of 1x10^8^ Inf U; ^b^N = 8; ^c^N = 7; ^d^N = 1; ^e^N = 3; ^f^N = 7.

In addition to the two Grade 3 laboratory abnormalities reported as adverse events (AEs), Grade 3 erythrocytes in urine was reported by one participant post-dose 2 vaccination with Ad26.Filo, but this was not reported as an AE as the participant was menstruating at the time of urinalysis.

**Table 3. EBOV GP-specific binding antibody responses (ELISA): descriptive statistics and responder rates; immunogenicity analysis set.**

|  | **56-day interval** | | | | **14-day interval** | |
| --- | --- | --- | --- | --- | --- | --- |
|  | Group 1 | Group 2 | Group 4 | Pooled placebo (Groups 1, 2, and 4) | Group 3 | |
|  | Ad26.Filo, MVA | MVA, Ad26.Filo | Ad26.ZEBOV, MVA^a^ | Placebo, placebo | MVA, Ad26.Filo^b^ | Placebo, placebo^b^ |
| **Baseline** |  |  |  |  |  |  |
| N | 15 | 15 | 12 | 7 | 14 | 2 |
| GMC, EU/mL (95% CI) | <LLOQ (<LLOQ; <LLOQ) | <LLOQ (<LLOQ; <LLOQ) | <LLOQ (<LLOQ; <LLOQ) | <LLOQ (<LLOQ; <LLOQ) | <LLOQ (<LLOQ; <LLOQ) | <LLOQ (<LLOQ; <LLOQ) |
| **Day 15** |  |  |  |  |  |  |
| N | 0 | 0 | 0 | 0 | 14 | 2 |
| GMC, EU/mL (95% CI) | - | - | - | - | <LLOQ (<LLOQ; 87) | <LLOQ (<LLOQ; <LLOQ) |
| Responder, n/N^c^ (%) | - | - | - | - | 6/14 (42.9) | 0/2 (0.0) |
| (95% CI) | - | - | - | - | (17.7; 71.1) | (0.0; 84.2) |
| **Day 29** |  |  |  |  |  |  |
| N | 0 | 15 | 0 | 3 | 0 | 0 |
| GMC, EU/mL (95% CI) | - | 106 (<LLOQ; 182) | - | <LLOQ (<LLOQ; <LLOQ) | - | - |
| Responder, n/N^c^ (%) | - | 10/15 (66.7) | - | 0/3 (0.0) | - | - |
| (95% CI) | - | (38.4; 88.2) | - | (0.0; 70.8) | - | - |
| **Day 36** |  |  |  |  |  |  |
| N | 0 | 0 | 0 | 0 | 14 | 2 |
| GMC, EU/mL (95% CI) | - | - | - | - | 5121 (3740; 7012) | <LLOQ (<LLOQ; <LLOQ) |
| Responder, n/N^c^ (%) | - | - | - | - | 14/14 (100.0) | 0/2 (0.0) |
| (95% CI) | - | - | - | - | (76.8; 100.0) | (0.0; 84.2) |
| **Day 50** |  |  |  |  |  |  |
| N | 0 | 0 | 0 | 0 | 13 | 2 |
| GMC, EU/mL (95% CI) | - | - | - | - | 4022 (2949; 5486) | <LLOQ (<LLOQ; <LLOQ) |
| Responder, n/N^c^ (%) | - | - | - | - | 13/13 (100.0) | 0/2 (0.0) |
| (95% CI) | - | - | - | - | (75.3; 100.0) | (0.0; 84.2) |
| **Day 57** |  |  |  |  |  |  |
| N | 15 | 14 | 12 | 7 | 0 | 0 |
| GMC, EU/mL (95% CI) | 946 (605; 1480) | 114 (73; 180) | 1553 (949; 2542) | <LLOQ (<LLOQ; <LLOQ) | - | - |
| Responder, n/N^c^ (%) | 15/15 (100.0) | 11/14 (78.6) | 12/12 (100.0) | 0/7 (0.0) | - | - |
| (95% CI) | (78.2; 100.0) | (49.2; 95.3) | (73.5; 100.0) | (0.0; 41.0) | - | - |
| **Day 71** |  |  |  |  |  |  |
| N | 15 | 14 | 11 | 7 | 0 | 0 |
| GMC, EU/mL (95% CI) | 12369 (7672; 19942) | 20102 (13516; 29898) | 13991 (8549; 22897) | <LLOQ (<LLOQ; <LLOQ) | - | - |
| Responder, n/N^c^ (%) | 15/15 (100.0) | 14/14 (100.0) | 11/11 (100.0) | 0/7 (0.0) | - | - |
| (95% CI) | (78.2; 100.0) | (76.8; 100.0) | (71.5; 100.0) | (0.0; 41.0) | - | - |
| **Day 78** |  |  |  |  |  |  |
| N | 14 | 14 | 11 | 7 | 0 | 0 |
| GMC, EU/mL (95% CI) | 11002 (6980; 17341) | 13867 (9478; 20289) | 11295 (6897; 18499) | <LLOQ (<LLOQ; <LLOQ) | - | - |
| Responder, n/N^c^ (%) | 14/14 (100.0) | 14/14 (100.0) | 11/11 (100.0) | 0/7 (0.0) | - | - |
| (95% CI) | (76.8; 100.0) | (76.8; 100.0) | (71.5; 100.0) | (0.0; 41.0) | - | - |
| **Day 92** |  |  |  |  |  |  |
| N | 15 | 14 | 11 | 7 | 0 | 0 |
| GMC, EU/mL (95% CI) | 7246 (4733; 11093) | 9232 (6092; 13990) | 8227 (5151; 13140) | <LLOQ (<LLOQ; <LLOQ) | - | - |
| Responder, n/N^c^ (%) | 15/15 (100.0) | 14/14 (100.0) | 11/11 (100.0) | 0/7 (0.0) | - | - |
| (95% CI) | (78.2; 100.0) | (76.8; 100.0) | (71.5; 100.0) | (0.0; 41.0) | - | - |
| **Day 180** |  |  |  |  |  |  |
| N | 14 | 14 | 10 | 7 | 8 | 0 |
| GMC, EU/mL (95% CI) | 2094 (1372; 3194) | 3163 (2020; 4952) | 3108 (1556; 6208) | <LLOQ (<LLOQ; <LLOQ) | 1916 (1010; 3636) | - |
| Responder, n/N^c^ (%) | 14/14 (100.0) | 14/14 (100.0) | 10/10 (100.0) | 0/7 (0.0) | 8/8 (100.0) | - |
| (95% CI) | (76.8; 100.0) | (76.8; 100.0) | (69.2; 100.0) | (0.0; 41.0) | (63.1; 100.0) | - |
| **Day 360** |  |  |  |  |  |  |
| N | 14 | 13 | 9 | 7 | 8 | 0 |
| GMC, EU/mL (95% CI) | 1229 (774; 1950) | 1410 (800; 2484) | 2025 (842; 4869) | <LLOQ (<LLOQ; <LLOQ) | 1914 (1024; 3575) | - |
| Responder, n/N^c^ (%) | 14/14 (100.0) | 13/13 (100.0) | 9/9 (100.0) | 0/7 (0.0) | 8/8 (100.0) | - |
| (95% CI) | (76.8; 100.0) | (75.3; 100.0) | (66.4; 100.0) | (0.0; 41.0) | (63.1; 100.0) | - |

ELISA = enzyme-linked immunosorbent assay; EBOV = *Zaire ebolavirus*; GP = glycoprotein; Inf U = infectious units; LLOQ = lower limit of quantification (66.96 EU/mL); MVA = MVA-BN-Filo; N = number of participants with data at that time point.

^a^MVA-BN-Filo at a dose of 1x10^8^ Inf U; ^b^Includes all Group 3 participants up to and including the Day 50 time point, after which only Group 3 participants who did not receive a booster vaccination are included; ^c^Number of participants with data at baseline and at that time point.

Geometric mean concentrations (GMCs) with 95% confidence intervals (CIs) are shown on the reported scale (ELISA units/mL).

Responder rates are shown with corresponding Exact Clopper-Pearson 95% CIs.

**Table 4. EBOV GP-specific binding antibody responses (ELISA): descriptive statistics and responder rates; immunogenicity analysis set; Group 3 booster subset.**

|  |  | **14-day interval** | | |
| --- | --- | --- | --- | --- |
|  |  | MVA, Ad26.Filo, Ad26.Filo |  | Placebo, placebo, placebo |
| **Baseline** |  |  |  |  |
| N |  | 5 |  | 2 |
| GMC, EU/mL (95% CI) |  | <LLOQ (<LLOQ; <LLOQ) |  | <LLOQ (<LLOQ; <LLOQ) |
| **Day 15** |  |  |  |  |
| N |  | 5 |  | 2 |
| GMC, EU/mL (95% CI) |  | <LLOQ (<LLOQ; 89) |  | <LLOQ (<LLOQ; <LLOQ) |
| Responder, n/N^a^ (%) |  | 1/5 (20.0) |  | 0/2 (0.0) |
| 95% CI |  | (0.5; 71.6) |  | (0.0; 84.2) |
| **Day 36** |  |  |  |  |
| N |  | 5 |  | 2 |
| GMC, EU/mL (95% CI) |  | 3843 (1761; 8388) |  | <LLOQ (<LLOQ; <LLOQ) |
| Responder, n/N^a^ (%) |  | 5/5 (100.0) |  | 0/2 (0.0) |
| 95% CI |  | (47.8; 100.0) |  | (0.0; 84.2) |
| **Day 50** |  |  |  |  |
| N |  | 5 |  | 2 |
| GMC, EU/mL (95% CI) |  | 3883 (2394; 6299) |  | <LLOQ (<LLOQ; <LLOQ) |
| Responder, n/N^a^ (%) |  | 5/5 (100.0) |  | 0/2 (0.0) |
| 95% CI |  | (47.8; 100.0) |  | (0.0; 84.2) |
| **Day 92** |  |  |  |  |
| N |  | 5 |  | 2 |
| GMC, EU/mL (95% CI) |  | 2425 (1503; 3911) |  | <LLOQ (<LLOQ; <LLOQ) |
| Responder, n/N^a^ (%) |  | 5/5 (100.0) |  | 0/2 (0.0) |
| 95% CI |  | (47.8; 100.0) |  | (0.0; 84.2) |
| **Day 99** |  |  |  |  |
| N |  | 5 |  | 2 |
| GMC, EU/mL (95% CI) |  | 13445 (4125; 43829) |  | <LLOQ (<LLOQ; <LLOQ) |
| Responder, n/N^a^ (%) |  | 5/5 (100.0) |  | 0/2 (0.0) |
| 95% CI |  | (47.8; 100.0) |  | (0.0; 84.2) |
| **Day 113** |  |  |  |  |
| N |  | 5 |  | 2 |
| GMC, EU/mL (95% CI) |  | 15715 (7146; 34558) |  | <LLOQ (<LLOQ; <LLOQ) |
| Responder, n/N^a^ (%) |  | 5/5 (100.0) |  | 0/2 (0.0) |
| 95% CI |  | (47.8; 100.0) |  | (0.0; 84.2) |
| **Day 180** |  |  |  |  |
| N |  | 5 |  | 2 |
| GMC, EU/mL (95% CI) |  | 3918 (1834; 8370) |  | <LLOQ (<LLOQ; <LLOQ) |
| Responder, n/N^a^ (%) |  | 5/5 (100.0) |  | 0/2 (0.0) |
| 95% CI |  | (47.8; 100.0) |  | (0.0; 84.2) |
| **Day 360** |  |  |  |  |
| N |  | 4 |  | 1 |
| GMC, EU/mL (95% CI) |  | 1592 (506; 5010) |  | <LLOQ |
| Responder, n/N^a^ (%) |  | 4/4 (100.0) |  | 0/1 (0.0) |
| 95% CI |  | (39.8; 100.0) |  | (0.0; 97.5) |

CIs = confidence intervals; EBOV = *Zaire ebolavirus*; ELISA = enzyme-linked immunosorbent assay; GMC = geometric mean concentration; GP = glycoprotein; LLOQ = lower limit of quantification (66.96 EU/mL); MVA = MVA-BN-Filo; N = number of participants with data at that time point.

^a^Number of participants with data at baseline and at that time point.

All time points include only Group 3 participants who receive a booster vaccination on Day 92.

**Table 5. SUDV GP-specific binding antibody responses (ELISA): descriptive statistics and responder rates; immunogenicity analysis set.**

|  | **56-day interval** | | | | **14-day interval** | |
| --- | --- | --- | --- | --- | --- | --- |
|  | Group 1 | Group 2 | Group 4 | Pooled placebo (Groups 1, 2, and 4) | Group 3 | |
|  | Ad26.Filo, MVA | MVA, Ad26.Filo | Ad26.ZEBOV, MVA^a^ | Placebo, placebo | MVA, Ad26.Filo^b^ | Placebo, placebo^b^ |
| **Baseline** |  |  |  |  |  |  |
| N | 15 | 15 | 12 | 5 | 14 | 2 |
| GMC, EU/mL (95% CI) | <LLOQ (<LLOQ; <LLOQ) | <LLOQ (<LLOQ; <LLOQ) | <LLOQ (<LLOQ; <LLOQ) | <LLOQ (<LLOQ; <LLOQ) | <LLOQ (<LLOQ; <LLOQ) | <LLOQ (<LLOQ; <LLOQ) |
| **Day 15** |  |  |  |  |  |  |
| N | 0 | 0 | 0 | 0 | 14 | 2 |
| GMC, EU/mL (95% CI) | - | - | - | - | 28 (<LLOQ; 57) | <LLOQ (<LLOQ; <LLOQ) |
| Responder, n/N^c^ (%) | - | - | - | - | 10/14 (71.4) | 0/2 (0.0) |
| (95% CI) | - | - | - | - | (41.9; 91.6) | (0.0; 84.2) |
| **Day 29** |  |  |  |  |  |  |
| N | 0 | 15 | 0 | 3 | 0 | 0 |
| GMC, EU/mL (95% CI) | - | 40 (22; 72) | - | <LLOQ (<LLOQ; <LLOQ) | - | - |
| Responder, n/N^c^ (%) | - | 11/15 (73.3) | - | 0/3 (0.0) | - | - |
| (95% CI) | - | (44.9, 92.2) | - | (0.0, 70.8) | - | - |
| **Day 36** |  |  |  |  |  |  |
| N | 0 | 0 | 0 | 0 | 14 | 2 |
| GMC, EU/mL (95% CI) | - | - | - | - | 1627 (1159; 2283) | <LLOQ (<LLOQ; <LLOQ) |
| Responder, n/N^c^ (%) | - | - | - | - | 14/14 (100) | 0/2 (0.0) |
| (95% CI) | - | - | - | - | (76.8; 100.0) | (0.0; 84.2) |
| **Day 50** |  |  |  |  |  |  |
| N | 0 | 0 | 0 | 0 | 13 | 2 |
| GMC, EU/mL (95% CI) | - | - | - | - | 982 (673; 1432) | <LLOQ (<LLOQ; <LLOQ) |
| Responder, n/N^c^ (%) | - | - | - | - | 13/13 (100) | 0/2 (0.0) |
| (95% CI) | - | - | - | - | (75.3; 100.0) | (0.0; 84.2) |
| **Day 57** |  |  |  |  |  |  |
| N | 15 | 14 | 12 | 5 | 0 | 0 |
| GMC, EU/mL (95% CI) | 186 (96; 362) | 37 (23; 60) | <LLOQ  (<LLOQ; 19) | <LLOQ (<LLOQ; <LLOQ) | - | - |
| Responder, n/N^c^ (%) | 14/15 (93.3) | 11/14 (78.6) | 2/12 (16.7) | 0/5 (0.0) | - | - |
| (95% CI) | (68.1; 99.8) | (49.2; 95.3) | (2.1; 48.4) | (0.0; 52.2) | - | - |
| **Day 71** |  |  |  |  |  |  |
| N | 15 | 14 | 0 | 5 | 0 | 0 |
| GMC, EU/mL (95% CI) | 3145 (1945; 5084) | 7004 (4443; 11,042) | - | <LLOQ (<LLOQ; <LLOQ) | - | - |
| Responder, n/N^c^ (%) | 15/15 (100) | 14/14 (100) | - | 0/5 (0) | - | - |
| (95% CI) | (78.2; 100.0) | (76.8; 100.0) | - | (0.0; 52.2) | - | - |
| **Day 78** |  |  |  |  |  |  |
| N | 14 | 14 | 11 | 5 | 0 | 0 |
| GMC, EU/mL (95% CI) | 2942 (1804; 4797) | 7273 (4613; 11,466) | 259 (145; 464) | <LLOQ (<LLOQ; <LLOQ) | - | - |
| Responder, n/N^c^ (%) | 14/14 (100) | 14/14 (100) | 11/11 (100.0) | 0/5 (0.0) | - | - |
| (95% CI) | (76.8; 100.0) | (76.8; 100.0) | (71.5; 100.0) | (0.0; 52.2) | - | - |
| **Day 92** |  |  |  |  |  |  |
| N | 15 | 14 | 0 | 5 | 0 | 0 |
| GMC, EU/mL (95% CI) | 1601 (1002; 2558) | 3147 (2023; 4896) | - | <LLOQ (<LLOQ; <LLOQ) | - | - |
| Responder, n/N^c^ (%) | 15/15 (100) | 14/14 (100) | - | 0/5 (0) | - | - |
| (95% CI) | (78.2; 100.0) | (76.8; 100.0) | - | (0.0; 52.2) | - | - |
| **Day 180** |  |  |  |  |  |  |
| N | 14 | 14 | 0 | 5 | 8 | 0 |
| GMC, EU/mL (95% CI) | 421 (299; 776) | 652 (422; 1010) | - | <LLOQ (<LLOQ; <LLOQ) | 347 (145; 834) | - |
| Responder, n/N^c^ (%) | 13/14 (92.9) | 14/14 (100.0) | - | 0/5 (0.0) | 8/8 (100.0) | - |
| (95% CI) | (66.1; 99.8) | (76.8; 100.0) | - | (0.0; 52.2) | (63.1; 100.0) | - |
| **Day 360** |  |  |  |  |  |  |
| N | 14 | 13 | 9 | 5 | 8 | 0 |
| GMC, EU/mL (95% CI) | 231 (124; 431) | 324 (209; 502) | 43 (18; 102) | <LLOQ (<LLOQ; <LLOQ) | 283 (123; 651) | - |
| Responder, n/N^c^ (%) | 13/14 (92.9) | 13/13 (100.0) | 7/9 (77.8) | 0/5 (0.0) | 8/8 (100.0) | - |
| (95% CI) | (66.1; 99.8) | (75.3; 100.0) | (40.0; 97.2) | (0.0; 52.2) | (63.1; 100.0) | - |

ELISA = enzyme-linked immunosorbent assay; GP = glycoprotein; Inf U = infectious units; LLOQ = lower limit of quantification (14.86 EU/mL); MVA = MVA-BN-Filo; N = number of participants with data at that time point; SUDV = *Sudan ebolavirus*.

^a^MVA-BN-Filo at a dose of 1x10^8^ Inf U; ^b^Includes all Group 3 participants up to and including the Day 50 time point, after which point only Group 3 participants who did not receive a booster vaccination with vaccine/placebo on Day 92 are included; ^c^Number of participants with data at baseline and at that time point.

Geometric mean concentrations (GMCs) with 95% confidence intervals (CIs) are shown on the reported scale (ELISA units/mL).

Responder rates are shown with corresponding Exact Clopper-Pearson 95% CIs.

**Table 6. SUDV GP-specific binding antibody responses (ELISA): descriptive statistics and responder rates; immunogenicity analysis set; Group 3 booster subset.**

|  |  | **14-day Interval** | | |
| --- | --- | --- | --- | --- |
|  |  | MVA, Ad26.Filo, Ad26.Filo |  | Placebo, placebo, placebo |
| **Baseline** |  |  |  |  |
| N |  | 5 |  | 2 |
| GMC, EU/mL (95% CI) |  | <LLOQ (<LLOQ; 15) |  | <LLOQ (<LLOQ; <LLOQ) |
| **Day 15** |  |  |  |  |
| N |  | 5 |  | 2 |
| GMC, EU/mL (95% CI) |  | 44 (<LLOQ; 282) |  | <LLOQ (<LLOQ; <LLOQ) |
| Responder, n/N^a^ (%) |  | 4/5 (80.0) |  | 0/2 (0.0) |
| 95% CI |  | (28.4; 99.5) |  | (0.0; 84.2) |
| **Day 36** |  |  |  |  |
| N |  | 5 |  | 2 |
| GMC, EU/mL (95% CI) |  | 1650 (780; 3491) |  | <LLOQ (<LLOQ; <LLOQ) |
| Responder, n/N^a^ (%) |  | 5/5 (100.0) |  | 0/2 (0.0) |
| 95% CI |  | (47.8; 100.0) |  | (0.0; 84.2) |
| **Day 50** |  |  |  |  |
| N |  | 5 |  | 2 |
| GMC, EU/mL (95% CI) |  | 985 (487; 1995) |  | <LLOQ (<LLOQ; <LLOQ) |
| Responder, n/N^a^ (%) |  | 5/5 (100.0) |  | 0/2 (0.0) |
| 95% CI |  | (47.8; 100.0) |  | (0.0; 84.2) |
| **Day 92** |  |  |  |  |
| N |  | 5 |  | 2 |
| GMC, EU/mL (95% CI) |  | 524 (258; 1064) |  | <LLOQ (<LLOQ; <LLOQ) |
| Responder, n/N^a^ (%) |  | 5/5 (100.0) |  | 0/2 (0.0) |
| 95% CI |  | (47.8; 100.0) |  | (0.0; 84.2) |
| **Day 99** |  |  |  |  |
| N |  | 5 |  | 2 |
| GMC, EU/mL (95% CI) |  | 2748 (791; 9549) |  | <LLOQ (<LLOQ; <LLOQ) |
| Responder, n/N^a^ (%) |  | 5/5 (100.0) |  | 0/2 (0.0) |
| 95% CI |  | (47.8; 100.0) |  | (0.0; 84.2) |
| **Day 113** |  |  |  |  |
| N |  | 5 |  | 2 |
| GMC, EU/mL (95% CI) |  | 3012 (1142; 7941) |  | <LLOQ (<LLOQ; <LLOQ) |
| Responder, n/N^a^ (%) |  | 5/5 (100.0) |  | 0/2 (0.0) |
| 95% CI |  | (47.8; 100.0) |  | (0.0; 84.2) |
| **Day 180** |  |  |  |  |
| N |  | 5 |  | 2 |
| GMC, EU/mL (95% CI) |  | 915 (435; 1925) |  | <LLOQ (<LLOQ; <LLOQ) |
| Responder, n/N^a^ (%) |  | 5/5 (100.0) |  | 0/2 (0.0) |
| 95% CI |  | (47.8; 100.0) |  | (0.0; 84.2) |
| **Day 360** |  |  |  |  |
| N |  | 4 |  | 1 |
| GMC, EU/mL (95% CI) |  | 343 (161; 727) |  | <LLOQ |
| Responder, n/N^a^ (%) |  | 4/4 (100.0) |  | 0/1 (0.0) |
| 95% CI |  | (39.8; 100.0) |  | (0.0; 97.5) |

CI = confidence interval; ELISA = enzyme-linked immunosorbent assay; GMC = geometric mean concentration; GP = glycoprotein; LLOQ = lower limit of quantification (14.86 EU/mL); MVA = MVA-BN-Filo; N = number of participants with data at that time point; SUDV = *Sudan ebolavirus*.

^a^Number of participants with data at baseline and at that time point.

All time points include only Group 3 participants who receive a booster vaccination on Day 92.

**Table 7. MARV GP-specific binding antibody responses (ELISA): descriptive statistics and responder rates; immunogenicity analysis set.**

|  | **56-day interval** | | | | **14-day interval** | |
| --- | --- | --- | --- | --- | --- | --- |
|  | Group 1 | Group 2 | Group 4 | Pooled placebo (Groups 1, 2, and 4) | Group 3 | |
|  | Ad26.Filo, MVA | MVA, Ad26.Filo | Ad26.ZEBOV, MVA^a^ | Placebo, placebo | MVA, Ad26.Filo^b^ | Placebo, placebo^b^ |
| **Baseline** |  |  |  |  |  |  |
| N | 15 | 15 | 12 | 5 | 14 | 2 |
| GMC, EU/mL (95% CI) | <LLOQ (<LLOQ; <LLOQ) | <LLOQ (<LLOQ; <LLOQ) | <LLOQ  (<LLOQ;  <LLOQ) | <LLOQ (<LLOQ; <LLOQ) | <LLOQ (<LLOQ; <LLOQ) | <LLOQ (<LLOQ; <LLOQ) |
| **Day 15** |  |  |  |  |  |  |
| N | 0 | 0 | 0 | 0 | 14 | 2 |
| GMC, EU/mL (95% CI) | - | - | - | - | <LLOQ (<LLOQ; <LLOQ) | <LLOQ (<LLOQ; <LLOQ) |
| Responder, n/N^c^ (%) | - | - | - | - | 2/14 (14.3) | 0/2 (0.0) |
| (95% CI) | - | - | - | - | (1.8; 42.8) | (0.0; 84.2) |
| **Day 29** |  |  |  |  |  |  |
| N | 0 | 15 | 0 | 3 | 0 | 0 |
| GMC, EU/mL (95% CI) | - | <LLOQ (<LLOQ; 21) | - | <LLOQ (<LLOQ; <LLOQ) | - | - |
| Responder, n/N^c^ (%) | - | 5/15 (33.3) | - | 0/3 (0.0) | - | - |
| (95% CI) | - | (11.8; 61.6) | - | (0.0; 70.8) | - | - |
| **Day 36** |  |  |  |  |  |  |
| N | 0 | 0 | 0 | 0 | 14 | 2 |
| GMC, EU/mL (95% CI) | - | - | - | - | 208 (121; 357) | <LLOQ (<LLOQ; <LLOQ) |
| Responder, n/N^c^ (%) | - | - | - | - | 14/14 (100.0) | 0/2 (0.0) |
| (95% CI) | - | - | - | - | (76.8; 100.0) | (0.0; 84.2) |
| **Day 50** |  |  |  |  |  |  |
| N | 0 | 0 | 0 | 0 | 13 | 2 |
| GMC, EU/mL (95% CI) | - | - | - | - | 155 (82; 292) | <LLOQ (<LLOQ; <LLOQ) |
| Responder, n/N^c^ (%) | - | - | - | - | 13/13 (100.0) | 0/2 (0.0) |
| (95% CI) | - | - | - | - | (75.3; 100.0) | (0.0; 84.2) |
| **Day 57** |  |  |  |  |  |  |
| N | 15 | 14 | 12 | 5 | 0 | 0 |
| GMC, EU/mL (95% CI) | 52 (34; 81) | <LLOQ (<LLOQ; <LLOQ) | <LLOQ (<LLOQ; <LLOQ) | <LLOQ (<LLOQ; <LLOQ) | - | - |
| Responder, n/N^c^ (%) | 14/15 (93.3) | 2/14 (14.3) | 0/12 (0.0) | 0/5 (0.0) | - | - |
| (95% CI) | (68.1; 99.8) | (1.8; 42.8) | (0.0; 26.5) | (0.0; 52.2) | - | - |
| **Day 71** |  |  |  |  |  |  |
| N | 15 | 14 | 0 | 5 | 0 | 0 |
| GMC, EU/mL (95% CI) | 1694 (1075; 2671) | 861 (510; 1455) | - | <LLOQ (<LLOQ; <LLOQ) | - | - |
| Responder, n/N^c^ (%) | 15/15 (100.0) | 14/14 (100.0) | - | 0/5 (0.0) | - | - |
| (95% CI) | (78.2; 100.0) | (76.8; 100.0) | - | (0.0; 52.2) | - | - |
| **Day 78** |  |  |  |  |  |  |
| N | 14 | 14 | 11 | 5 | 0 | 0 |
| GMC, EU/mL (95% CI) | 1240 (825; 1862) | 631 (410; 970) | <LLOQ (<LLOQ; <LLOQ) | <LLOQ (<LLOQ; <LLOQ) | - | - |
| Responder, n/N^c^ (%) | 14/14 (100.0) | 14/14 (100.0) | 2/11 (18.2) | 0/5 (0.0) | - | - |
| (95% CI) | (76.8; 100.0) | (76.8; 100.0) | (2.3; 51.8) | (0.0; 52.2) | - | - |
| **Day 92** |  |  |  |  |  |  |
| N | 15 | 14 | 0 | 5 | 0 | 0 |
| GMC, EU/mL (95% CI) | 715 (452; 1130) | 472 (310; 720) | - | <LLOQ (<LLOQ; <LLOQ) | - | - |
| Responder, n/N^c^ (%) | 15/15 (100.0) | 14/14 (100.0) | - | 0/5 (0.0) | - | - |
| (95% CI) | (78.2; 100.0) | (76.8; 100.0) | - | (0.0; 52.2) | - | - |
| **Day 180** |  |  |  |  |  |  |
| N | 14 | 14 | 0 | 5 | 8 | 0 |
| GMC, EU/mL (95% CI) | 109 (62; 191) | 143 (84; 241) | - | <LLOQ (<LLOQ; <LLOQ) | 81 (28; 233) | - |
| Responder, n/N^c^ (%) | 13/14 (92.9) | 14/14 (100.0) | - | 0/5 (0.0) | 8/8 (100.0) | - |
| (95% CI) | (66.1; 99.8) | (76.8; 100.0) | - | (0.0; 52.2) | (63.1; 100.0) | - |
| **Day 360** |  |  |  |  |  |  |
| N | 14 | 13 | 9 | 5 | 8 | 0 |
| GMC, EU/mL (95% CI) | 53 (29; 97) | 81 (44; 147) | <LLOQ  (<LLOQ;  <LLOQ) | <LLOQ (<LLOQ; <LLOQ) | 65 (23; 181) | - |
| Responder, n/N^c^ (%) | 11/14 (78.6) | 12/13 (92.3) | 0/9 (0.0) | 0/5 (0.0) | 7/8 (87.5) | - |
| (95% CI) | (49.2; 95.3) | (64.0; 99.8) | (0.0; 33.6) | (0.0; 52.2) | (47.3; 99.7) | - |

ELISA = enzyme-linked immunosorbent assay; GP = glycoprotein; Inf U = infectious units; LLOQ = lower limit of quantification (19.19 EU/mL); MARV = *Marburg virus*; MVA = MVA-BN-Filo; N = number of participants with data at that time point.

^a^MVA-BN-Filo at a dose of 1x10^8^ Inf U; ^b^Includes all Group 3 participants up to and including the Day 50 time point, after which point only Group 3 participants who did not receive a booster vaccination with vaccine/placebo on Day 92 are included; ^c^Number of participants with data at baseline and at that time point.

Geometric mean concentrations (GMCs) with 95% confidence intervals (CIs) are shown on the reported scale (ELISA units/mL).

Responder rates are shown with corresponding Exact Clopper-Pearson 95% CIs.

**Table 8.** MARV GP-specific binding antibody responses (ELISA): descriptive statistics and responder rates; immunogenicity analysis set; Group 3 booster subset.

|  | | **14-day interval** | | |
| --- | --- | --- | --- | --- |
|  | | MVA, Ad26.Filo, Ad26.Filo |  | Placebo, placebo, placebo |
| **Baseline** |  |  |  |  |
| N |  | 5 |  | 2 |
| GMC, EU/mL (95% CI) |  | <LLOQ (<LLOQ; <LLOQ) |  | <LLOQ (<LLOQ; <LLOQ) |
| **Day 15** |  |  |  |  |
| N |  | 5 |  | 2 |
| GMC, EU/mL (95% CI) |  | <LLOQ (<LLOQ; <LLOQ) |  | <LLOQ (<LLOQ; <LLOQ) |
| Responder, n/N^a^ (%) |  | 1/5 (20.0) |  | 0/2 (0.0) |
| 95% CI |  | (0.5; 71.6) |  | (0.0; 84.2) |
| **Day 36** |  |  |  |  |
| N |  | 5 |  | 2 |
| GMC, EU/mL (95% CI) |  | 215 (52; 887) |  | <LLOQ (<LLOQ; <LLOQ) |
| Responder, n/N^a^ (%) |  | 5/5 (100.0) |  | 0/2 (0.0) |
| 95% CI |  | (47.8; 100.0) |  | (0.0; 84.2) |
| **Day 50** |  |  |  |  |
| N |  | 5 |  | 2 |
| GMC, EU/mL (95% CI) |  | 174 (40; 747) |  | <LLOQ (<LLOQ; <LLOQ) |
| Responder, n/N^a^ (%) |  | 5/5 (100.0) |  | 0/2 (0.0) |
| 95% CI |  | (47.8; 100.0) |  | (0.0; 84.2) |
| **Day 92** |  |  |  |  |
| N |  | 5 |  | 2 |
| GMC, EU/mL (95% CI) |  | 155 (36; 665) |  | <LLOQ (<LLOQ; <LLOQ) |
| Responder, n/N^a^ (%) |  | 5/5 (100.0) |  | 0/2 (0.0) |
| 95% CI |  | (47.8; 100.0) |  | (0.0; 84.2) |
| **Day 99** |  |  |  |  |
| N |  | 5 |  | 2 |
| GMC, EU/mL (95% CI) |  | 510 (92; 2827) |  | <LLOQ (<LLOQ; <LLOQ) |
| Responder, n/N^a^ (%) |  | 5/5 (100.0) |  | 0/2 (0.0) |
| 95% CI |  | (47.8; 100.0) |  | (0.0; 84.2) |
| **Day 113** |  |  |  |  |
| N |  | 5 |  | 2 |
| GMC, EU/mL (95% CI) |  | 825 (230; 2959) |  | <LLOQ (<LLOQ; <LLOQ) |
| Responder, n/N^a^ (%) |  | 5/5 (100.0) |  | 0/2 (0.0) |
| 95% CI |  | (47.8; 100.0) |  | (0.0; 84.2) |
| **Day 180** |  |  |  |  |
| N |  | 5 |  | 2 |
| GMC, EU/mL (95% CI) |  | 236 (54; 1024) |  | <LLOQ (<LLOQ; <LLOQ) |
| Responder, n/N^a^ (%) |  | 5/5 (100.0) |  | 0/2 (0.0) |
| 95% CI |  | (47.8; 100.0) |  | (0.0; 84.2) |
| **Day 360** |  |  |  |  |
| N |  | 4 |  | 1 |
| GMC, EU/mL (95% CI) |  | 133 (27; 663) |  | <LLOQ |
| Responder, n/N^a^ (%) |  | 4/4 (100.0) |  | 0/1 (0.0) |
| 95% CI |  | (39.8; 100.0) |  | (0.0; 97.5) |

CI = confidence interval; ELISA = enzyme-linked immunosorbent assay; GMC = geometric mean concentration; GP = glycoprotein; LLOQ = lower limit of quantification (19.19 EU/mL); MARV = *Marburg* virus; MVA = MVA-BN-Filo; N = number of participants with data at that time point.

^a^Number of participants with data at baseline and at that time point.

All time points include only Group 3 participants who receive a booster vaccination on Day 92.

**Table 9. EBOV GP-specific neutralizing antibody responses (psVNA): descriptive statistics and responder rates; immunogenicity analysis set.**

|  | **56-day interval** | | | | **14-day interval** | |
| --- | --- | --- | --- | --- | --- | --- |
|  | Group 1 | Group 2 | Group 4 | Pooled placebo (Groups 1, 2 and 4) | Group 3 | |
|  | Ad26.Filo, MVA | MVA, Ad26.Filo | Ad26.ZEBOV, MVA^a^ | Placebo, placebo | MVA, Ad26.Filo^b^ | Placebo, placebo^b^ |
| **Baseline** |  |  |  |  |  |  |
| N | 15 | 15 | 12 | 7 | 14 | 2 |
| GMT, IC_50_ (95% CI) | <LLOQ (<LLOQ; <LLOQ) | <LLOQ (<LLOQ; <LLOQ) | <LLOQ (<LLOQ; <LLOQ) | <LLOQ (<LLOQ; <LLOQ) | <LLOQ (<LLOQ; <LLOQ) | <LLOQ (<LLOQ; <LLOQ) |
| **Day 15** |  |  |  |  |  |  |
| N | 0 | 0 | 0 | 0 | 14 | 2 |
| GMT, IC_50_ (95% CI) | - | - | - | - | <LLOQ (<LLOQ; <LLOQ) | <LLOQ (<LLOQ; <LLOQ) |
| Responder, n/N^c^ (%) | - | - | - | - | 1/14 (7.1) | 0/2 (0.0) |
| 95% CI | - | - | - | - | (0.2; 33.9) | (0.0; 84.2) |
| **Day 29** |  |  |  |  |  |  |
| N | 0 | 15 | 0 | 3 | 0 | 0 |
| GMT, IC_50_ (95% CI) | - | <LLOQ (<LLOQ; <LLOQ) | - | <LLOQ (<LLOQ; <LLOQ) | - | - |
| Responder, n/N^c^ (%) | - | 0/15 (0.0) | - | 0/3 (0.0) | - | - |
| 95% CI | - | (0.0; 21.8) | - | (0.0; 70.8) | - | - |
| **Day 36** |  |  |  |  |  |  |
| N | 0 | 0 | 0 | 0 | 14 | 2 |
| GMT, IC_50_ (95% CI) | - | - | - | - | 388 (201; 749) | <LLOQ (<LLOQ; <LLOQ) |
| Responder, n/N^c^ (%) | - | - | - | - | 12/14 (85.7) | 0/2 (0.0) |
| 95% CI | - | - | - | - | (57.2; 98.2) | (0.0; 84.2) |
| **Day 50** |  |  |  |  |  |  |
| N | 0 | 0 | 0 | 0 | 13 | 2 |
| GMT, IC_50_ (95% CI) | - | - | - | - | 708 (474; 1057) | <LLOQ (<LLOQ; <LLOQ) |
| Responder, n/N^c^ (%) | - | - | - | - | 13/13 (100.0) | 0/2 (0.0) |
| 95% CI | - | - | - | - | (75.3; 100.0) | (0.0; 84.2) |
| **Day 57** |  |  |  |  |  |  |
| N | 15 | 14 | 12 | 7 | 0 | 0 |
| GMT, IC_50_ (95% CI) | <LLOQ (<LLOQ; 167) | <LLOQ (<LLOQ; <LLOQ) | 427 (234; 779) | <LLOQ (<LLOQ; <LLOQ) | - | - |
| Responder, n/N^c^ (%) | 7/15 (46.7) | 1/14 (7.1) | 12/12 (100.0) | 0/7 (0.0) | - | - |
| 95% CI | (21.3; 73.4) | (0.2; 33.9) | (73.5; 100.0) | (0.0; 41.0) | - | - |
| **Day 71** |  |  |  |  |  |  |
| N | 15 | 14 | 11 | 7 | 0 | 0 |
| GMT, IC_50_ (95% CI) | 5820 (3467; 9772) | 10154 (5487; 18791) | 5695 (2803; 11573) | <LLOQ (<LLOQ; <LLOQ) | - | - |
| Responder, n/N^c^ (%) | 15/15 (100.0) | 14/14 (100.0) | 11/11 (100.0) | 0/7 (0.0) | - | - |
| 95% CI | (78.2; 100.0) | (76.8; 100.0) | (71.5; 100.0) | (0.0; 41.0) | - | - |
| **Day 78** |  |  |  |  |  |  |
| N | 14 | 14 | 11 | 7 | 0 | 0 |
| GMT, IC_50_ (95% CI) | 3614 (2012; 6492) | 6343 (3538; 11371) | 6192 (3275; 11707) | <LLOQ (<LLOQ; <LLOQ) | - | - |
| Responder, n/N^c^ (%) | 14/14 (100.0) | 14/14 (100.0) | 11/11 (100.0) | 0/7 (0.0) | - | - |
| 95% CI | (76.8; 100.0) | (76.8; 100.0) | (71.5; 100.0) | (0.0; 41.0) | - | - |
| **Day 92** |  |  |  |  |  |  |
| N | 15 | 14 | 11 | 7 | 0 | 0 |
| GMT, IC_50_ (95% CI) | 3031 (1881; 4884) | 4403 (2609; 7432) | 3736 (1838; 7593) | <LLOQ (<LLOQ; <LLOQ) | - | - |
| Responder, n/N^c^ (%) | 15/15 (100.0) | 14/14 (100.0) | 11/11 (100.0) | 0/7 (0.0) | - | - |
| 95% CI | (78.2; 100.0) | (76.8; 100.0) | (71.5; 100.0) | (0.0; 41.0) | - | - |
| **Day 180** |  |  |  |  |  |  |
| N | 14 | 14 | 10 | 7 | 8 | 0 |
| GMT, IC_50_ (95% CI) | 519 (315; 855) | 1385 (951; 2017) | 1148 (408; 3227) | <LLOQ (<LLOQ; <LLOQ) | 878 (512; 1508) | - |
| Responder, n/N^c^ (%) | 14/14 (100.0) | 14/14 (100.0) | 10/10 (100.0) | 0/7 (0.0) | 8/8 (100.0) | - |
| 95% CI | (76.8; 100.0) | (76.8; 100.0) | (69.2; 100.0) | (0.0; 41.0) | (63.1; 100.0) | - |
| **Day 360** |  |  |  |  |  |  |
| N | 14 | 13 | 9 | 0 | 8 | 0 |
| GMT, IC_50_ (95% CI) | 175 (<LLOQ; 306) | 449 (267; 754) | 656 (272; 1582) | - | 734 (412; 1305) | - |
| Responder, n/N^c^ (%) | 9/14 (64.3) | 12/13 (92.3) | 8/9 (88.9) | - | 8/8 (100.0) | - |
| 95% CI | (35.1; 87.2) | (64.0; 99.8) | (51.8; 99.7) | - | (63.1; 100.0) | - |

CI = confidence interval; EBOV = *Zaire ebolavirus;* ELISA = enzyme-linked immunosorbent assay; GMT = geometric mean titer; GP = glycoprotein; IC_50_ = 50% inhibitory concentration; Inf U = infectious units; LLOQ = lower limit of quantification (120 IC_50_); MVA = MVA-BN-Filo; N = number of participants with data at that time point; psVNA = pseudovirion neutralization assay.

^a^MVA-BN-Filo at a dose of 1x10^8^ Inf U; ^b^Includes all Group 3 participants up to and including the Day 50 time point, after which point only Group 3 participants who did not receive a booster vaccination with vaccine/placebo on Day 92 are included; ^c^Number of participants with data at baseline and at that time point.

Responder rates are shown with corresponding Exact Clopper-Pearson 95% CIs.

**Table 10. EBOV GP-specific neutralizing antibody responses (psVNA): descriptive statistics and responder rates; immunogenicity analysis set; Group 3 booster subset.**

|  |  | **14-day interval** | | |
| --- | --- | --- | --- | --- |
|  |  | MVA, Ad26.Filo, Ad26.Filo |  | Placebo, placebo, placebo |
| **Baseline** |  |  |  |  |
| N |  | 5 |  | 2 |
| GMT, IC_50_ (95% CI) |  | <LLOQ (<LLOQ; <LLOQ) |  | <LLOQ (<LLOQ; <LLOQ) |
| **Day 15** |  |  |  |  |
| N |  | 5 |  | 2 |
| GMT, IC_50_ (95% CI) |  | <LLOQ (<LLOQ; <LLOQ) |  | <LLOQ (<LLOQ; <LLOQ) |
| Responder, n/N^a^ (%) |  | 0/5 (0.0) |  | 0/2 (0.0) |
| 95% CI |  | (0.0; 52.2) |  | (0.0; 84.2) |
| **Day 36** |  |  |  |  |
| N |  | 5 |  | 2 |
| GMT, IC_50_ (95% CI) |  | 200 (<LLOQ; 802) |  | <LLOQ (<LLOQ; <LLOQ) |
| Responder, n/N^a^ (%) |  | 3/5 (60.0) |  | 0/2 (0.0) |
| 95% CI |  | (14.7; 94.7) |  | (0.0; 84.2) |
| **Day 50** |  |  |  |  |
| N |  | 5 |  | 2 |
| GMT, IC_50_ (95% CI) |  | 443 (188; 1046) |  | <LLOQ (<LLOQ; <LLOQ) |
| Responder, n/N^a^ (%) |  | 5/5 (100.0) |  | 0/2 (0.0) |
| 95% CI |  | (47.8; 100.0) |  | (0.0; 84.2) |
| **Day 92** |  |  |  |  |
| N |  | 5 |  | 2 |
| GMT, IC_50_ (95% CI) |  | 520 (168; 1612) |  | <LLOQ (<LLOQ; <LLOQ) |
| Responder, n/N^a^ (%) |  | 5/5 (100.0) |  | 0/2 (0.0) |
| 95% CI |  | (47.8; 100.0) |  | (0.0; 84.2) |
| **Day 99** |  |  |  |  |
| N |  | 5 |  | 2 |
| GMT, IC_50_ (95% CI) |  | 5589 (1155; 27051) |  | <LLOQ (<LLOQ; <LLOQ) |
| Responder, n/N^a^ (%) |  | 5/5 (100.0) |  | 0/2 (0.0) |
| 95% CI |  | (47.8; 100.0) |  | (0.0; 84.2) |
| **Day 113** |  |  |  |  |
| N |  | 5 |  | 2 |
| GMT, IC_50_ (95% CI) |  | 7779 (2604; 23239) |  | <LLOQ (<LLOQ; <LLOQ) |
| Responder, n/N^a^ (%) |  | 5/5 (100.0) |  | 0/2 (0.0) |
| 95% CI |  | (47.8; 100.0) |  | (0.0; 84.2) |
| **Day 180** |  |  |  |  |
| N |  | 5 |  | 2 |
| GMT, IC_50_ (95% CI) |  | 1823 (703; 4729) |  | <LLOQ (<LLOQ; <LLOQ) |
| Responder, n/N^a^ (%) |  | 5/5 (100.0) |  | 0/2 (0.0) |
| 95% CI |  | (47.8; 100.0) |  | (0.0; 84.2) |
| **Day 360** |  |  |  |  |
| N |  | 5 |  | 0 |
| GMT, IC_50_ (95% CI) |  | 453 (248; 829) |  | - |
| Responder, n/N^a^ (%) |  | 5/5 (100.0) |  | - |
| 95% CI |  | (47.8; 100.0) |  | - |

CI = confidence interval; EBOV = *Zaire* ebolavirus; GMT = geometric mean titer; GP = glycoprotein; IC_50_ = 50% inhibitory concentration; LLOQ = lower limit of quantification (120 IC_50_); MVA = MVA-BN-Filo; N = number of participants with data at that time point; psVNA = pseudovirion neutralization assay.

^a^Number of participants with data at baseline and at that time point.

All time points include only Group 3 participants who receive a booster vaccination on Day 92.

**Table 11. SUDV GP-specific neutralizing antibody responses (psVNA): descriptive statistics and responder rates; immunogenicity analysis set.**

|  | **56-day interval** | | | | **14-day interval** | |
| --- | --- | --- | --- | --- | --- | --- |
|  | Ad26.Filo, MVA | MVA, Ad26.Filo | Ad26.ZEBOV, MVA^a^ | Placebo, placebo | MVA, Ad26.Filo^b^ | Placebo, placebo^b^ |
| **Baseline** |  |  |  |  |  |  |
| N | 15 | 15 | 0 | 5 | 14 | 2 |
| GMT, IC_50_ (95% CI) | <LLOQ (<LLOQ; <LLOQ) | <LLOQ (<LLOQ; <LLOQ) | - | <LLOQ (<LLOQ; <LLOQ) | <LLOQ (<LLOQ; <LLOQ) | <LLOQ (<LLOQ; <LLOQ) |
| **Day 15** |  |  |  |  |  |  |
| N | 0 | 0 | 0 | 0 | 14 | 2 |
| GMT, IC_50_ (95% CI) | - | - | - | - | <LLOQ (<LLOQ; <LLOQ) | <LLOQ (<LLOQ; <LLOQ) |
| Responder, n/N^c^ (%) | - | - | - | - | 0/14 (0.0) | 0/2 (0.0) |
| 95% CI | - | - | - | - | (0.0; 23.2) | (0.0; 84.2) |
| **Day 29** |  |  |  |  |  |  |
| N | 0 | 15 | 0 | 3 | 0 | 0 |
| GMT, IC_50_ (95% CI) | - | <LLOQ (<LLOQ; <LLOQ) | - | <LLOQ (<LLOQ; <LLOQ) | - | - |
| Responder, n/N^c^ (%) | - | 0/15 (0.0) | - | 0/3 (0.0) | - | - |
| 95% CI | - | (0.0; 21.8) | - | (0.0; 70.8) | - | - |
| **Day 36** |  |  |  |  |  |  |
| N | 0 | 0 | 0 | 0 | 14 | 2 |
| GMT, IC_50_ (95% CI) | - | - | - | - | 45 (<LLOQ; 89) | <LLOQ (<LLOQ; <LLOQ) |
| Responder, n/N^c^ (%) | - | - | - | - | 5/14 (35.7) | 0/2 (0.0) |
| 95% CI | - | - | - | - | (12.8; 64.9) | (0.0; 84.2) |
| **Day 50** |  |  |  |  |  |  |
| N | 0 | 0 | 0 | 0 | 13 | 2 |
| GMT, IC_50_ (95% CI) | - | - | - | - | 72 (<LLOQ; 155) | <LLOQ (<LLOQ; <LLOQ) |
| Responder, n/N^c^ (%) | - | - | - | - | 7/13 (53.8) | 0/2 (0.0) |
| 95% CI | - | - | - | - | (25.1; 80.8) | (0.0; 84.2) |
| **Day 57** |  |  |  |  |  |  |
| N | 15 | 14 | 0 | 5 | 0 | 0 |
| GMT, IC_50_ (95% CI) | <LLOQ (<LLOQ; <LLOQ) | <LLOQ (<LLOQ; <LLOQ) | - | <LLOQ (<LLOQ; <LLOQ) | - | - |
| Responder, n/N^c^ (%) | 1/15 (6.7) | 0/14 (0.0) | - | 0/5 (0.0) | - | - |
| 95% CI | (0.2; 31.9) | (0.0; 23.2) | - | (0.0; 52.2) | - | - |
| **Day 71** |  |  |  |  |  |  |
| N | 15 | 14 | 0 | 5 | 0 | 0 |
| GMT, IC_50_ (95% CI) | 992 (526; 1870) | 2232 (1254; 3972) | - | <LLOQ (<LLOQ; <LLOQ) | - | - |
| Responder, n/N^c^ (%) | 15/15 (100.0) | 14/14 (100.0) | - | 0/5 (0.0) | - | - |
| 95% CI | (78.2; 100.0) | (76.8; 100.0) | - | (0.0; 52.2) | - | - |
| **Day 78** |  |  |  |  |  |  |
| N | 14 | 14 | 0 | 5 | 0 | 0 |
| GMT, IC_50_ (95% CI) | 526 (233; 1187) | 1483 (859; 2561) | - | <LLOQ (<LLOQ; <LLOQ) | - | - |
| Responder, n/N^c^ (%) | 13/14 (92.9) | 14/14 (100.0) | - | 0/5 (0.0) | - | - |
| 95% CI | (66.1; 99.8) | (76.8; 100.0) | - | (0.0; 52.2) | - | - |
| **Day 92** |  |  |  |  |  |  |
| N | 15 | 14 | 0 | 5 | 0 | 0 |
| GMT, IC_50_ (95% CI) | 401 (200; 805) | 952 (585; 1549) | - | <LLOQ (<LLOQ; <LLOQ) | - | - |
| Responder, n/N^c^ (%) | 14/15 (93.3) | 14/14 (100.0) | - | 0/5 (0.0) | - | - |
| 95% CI | (68.1; 99.8) | (76.8; 100.0) | - | (0.0; 52.2) | - | - |
| **Day 180** |  |  |  |  |  |  |
| N | 14 | 14 | 0 | 5 | 8 | 0 |
| GMT, IC_50_ (95% CI) | 72 (<LLOQ; 144) | 228 (115; 452) | - | <LLOQ (<LLOQ; <LLOQ) | 99 (40; 244) | - |
| Responder, n/N^c^ (%) | 8/14 (57.1) | 12/14 (85.7) | - | 0/5 (0.0) | 6/8 (75.0) | - |
| 95% CI | (28.9; 82.3) | (57.2; 98.2) | - | (0.0; 52.2) | (34.9; 96.8) | - |
| **Day 360** |  |  |  |  |  |  |
| N | 14 | 13 | 0 | 0 | 8 | 0 |
| GMT, IC_50_ (95% CI) | 46 (<LLOQ; 92) | 147 (72; 301) | - | - | 95 (<LLOQ; 288) | - |
| Responder, n/N^c^ (%) | 5/14 (35.7) | 10/13 (76.9) | - | - | 5/8 (62.5) | - |
| 95% CI | (12.8; 64.9) | (46.2; 95.0) | - | - | (24.5; 91.5) | - |

CI = confidence iinterval; GMT = geometric mean titer; GP = glycoprotein; IC_50_ = 50% inhibitory concentration; Inf U = infectious units; LLOQ = lower limit of quantification (40 IC_50_); MVA = MVA-BN-Filo; N = number of participants with data at that time point; psVNA = pseudovirion neutralization assay; SUDV = *Sudan ebolavirus*.

^a^MVA-BN-Filo at a dose of 1x10^8^ Inf U; ^b^Includes all Group 3 participants up to and including the Day 50 time point, after which point only Group 3 participants who did not receive a booster vaccination with vaccine/placebo on Day 92 are included; ^c^Number of participants with data at baseline and at that time point.

Responder rates are shown with corresponding Exact Clopper-Pearson 95% CIs.

**Table 12. SUDV GP-specific neutralizing antibody responses (psVNA): descriptive statistics and responder rates; immunogenicity analysis set; Group 3 booster subset.**

|  |  | **14-day interval** | | |
| --- | --- | --- | --- | --- |
|  |  | MVA, Ad26.Filo, Ad26.Filo |  | Placebo, placebo, placebo |
| **Baseline** |  |  |  |  |
| N |  | 5 |  | 2 |
| GMT, IC_50_ (95% CI) |  | <LLOQ (<LLOQ; <LLOQ) |  | <LLOQ (<LLOQ; <LLOQ) |
| **Day 15** |  |  |  |  |
| N |  | 5 |  | 2 |
| GMT, IC_50_ (95% CI) |  | <LLOQ (<LLOQ; <LLOQ) |  | <LLOQ (<LLOQ; <LLOQ) |
| Responder, n/N^a^ (%) |  | 0/5 (0.0) |  | 0/2 (0.0) |
| 95% CI |  | (0.0; 52.2) |  | (0.0; 84.2) |
| **Day 36** |  |  |  |  |
| N |  | 5 |  | 2 |
| GMT, IC_50_ (95% CI) |  | <LLOQ (<LLOQ; 79) |  | <LLOQ (<LLOQ; <LLOQ) |
| Responder, n/N^a^ (%) |  | 1/5 (20.0) |  | 0/2 (0.0) |
| 95% CI |  | (0.5; 71.6) |  | (0.0; 84.2) |
| **Day 50** |  |  |  |  |
| N |  | 5 |  | 2 |
| GMT, IC_50_ (95% CI) |  | 75 (<LLOQ; 337) |  | <LLOQ (<LLOQ; <LLOQ) |
| Responder, n/N^a^ (%) |  | 3/5 (60.0) |  | 0/2 (0.0) |
| 95% CI |  | (14.7; 94.7) |  | (0.0; 84.2) |
| **Day 92** |  |  |  |  |
| N |  | 5 |  | 2 |
| GMT, IC_50_ (95% CI) |  | 63 (<LLOQ; 236) |  | <LLOQ (<LLOQ; <LLOQ) |
| Responder, n/N^a^ (%) |  | 3/5 (60.0) |  | 0/2 (0.0) |
| 95% CI |  | (14.7; 94.7) |  | (0.0; 84.2) |
| **Day 99** |  |  |  |  |
| N |  | 5 |  | 2 |
| GMT, IC_50_ (95% CI) |  | 601 (52; 6995) |  | <LLOQ (<LLOQ; <LLOQ) |
| Responder, n/N^a^ (%) |  | 4/5 (80.0) |  | 0/2 (0.0) |
| 95% CI |  | (28.4; 99.5) |  | (0.0; 84.2) |
| **Day 113** |  |  |  |  |
| N |  | 5 |  | 2 |
| GMT, IC_50_ (95% CI) |  | 884 (280; 2788) |  | <LLOQ (<LLOQ; <LLOQ) |
| Responder, n/N^a^ (%) |  | 5/5 (100.0) |  | 0/2 (0.0) |
| 95% CI |  | (47.8; 100.0) |  | (0.0; 84.2) |
| **Day 180** |  |  |  |  |
| N |  | 5 |  | 2 |
| GMT, IC_50_ (95% CI) |  | 346 (155; 774) |  | <LLOQ (<LLOQ; <LLOQ) |
| Responder, n/N^a^ (%) |  | 5/5 (100.0) |  | 0/2 (0.0) |
| 95% CI |  | (47.8; 100.0) |  | (0.0; 84.2) |
| **Day 360** |  |  |  |  |
| N |  | 5 |  | 0 |
| GMT, IC_50_ (95% CI) |  | 178 (<LLOQ; 917) |  | - |
| Responder, n/N^a^ (%) |  | 4/5 (80.0) |  | - |
| 95% CI |  | (28.4; 99.5) |  | - |

CI = confidence interval; GMT = geometric mean titer; GP = glycoprotein; IC_50_ = 50% inhibitory concentration; LLOQ = lower limit of quantification (40 IC_50_); MVA = MVA-BN-Filo; N = number of participants with data at that time point; psVNA = pseudovirion neutralization assay; SUDV = *Sudan ebolavirus*.

^a^Number of participants with data at baseline and at that time point.

All time points include only Group 3 participants who receive a booster vaccination with vaccine/placebo on Day 92.

**Table 13. MARV GP-specific neutralizing antibody responses (psVNA): descriptive statistics and responder rates; immunogenicity analysis set.**

|  | **56-day interval** | | | | **14-day interval** | |
| --- | --- | --- | --- | --- | --- | --- |
|  | Ad26.Filo, MVA | MVA, Ad26.Filo | Ad26.ZEBOV, MVA^a^ | Placebo, placebo | MVA, Ad26.Filo^b^ | Placebo, placebo^b^ |
| **Baseline** |  |  |  |  |  |  |
| N | 15 | 15 | 0 | 5 | 14 | 2 |
| GMT, IC_50_ (95% CI) | <LLOQ (<LLOQ; <LLOQ) | <LLOQ (<LLOQ; <LLOQ) | - | <LLOQ (<LLOQ; <LLOQ) | <LLOQ (<LLOQ; <LLOQ) | <LLOQ (<LLOQ; <LLOQ) |
| **Day 15** |  |  |  |  |  |  |
| N | 0 | 0 | 0 | 0 | 14 | 2 |
| GMT, IC_50_ (95% CI) | - | - | - | - | <LLOQ (<LLOQ; <LLOQ) | <LLOQ (<LLOQ; <LLOQ) |
| Responder, n/N^c^ (%) | - | - | - | - | 0/14 (0.0) | 0/2 (0.0) |
| 95% CI | - | - | - | - | (0.0; 23.2) | (0.0; 84.2) |
| **Day 29** |  |  |  |  |  |  |
| N | 0 | 15 | 0 | 3 | 0 | 0 |
| GMT, IC_50_ (95% CI) | - | <LLOQ (<LLOQ; <LLOQ) | - | <LLOQ (<LLOQ; <LLOQ) | - | - |
| Responder, n/N^c^ (%) | - | 0/15 (0.0) | - | 0/3 (0.0) | - | - |
| 95% CI | - | (0.0; 21.8) | - | (0.0; 70.8) | - | - |
| **Day 36** |  |  |  |  |  |  |
| N | 0 | 0 | 0 | 0 | 14 | 2 |
| GMT, IC_50_ (95% CI) | - | - | - | - | <LLOQ (<LLOQ; <LLOQ) | <LLOQ (<LLOQ; <LLOQ) |
| Responder, n/N^c^ (%) | - | - | - | - | 0/14 (0.0) | 0/2 (0.0) |
| 95% CI | - | - | - | - | (0.0; 23.2) | (0.0; 84.2) |
| **Day 50** |  |  |  |  |  |  |
| N | 0 | 0 | 0 | 0 | 13 | 2 |
| GMT, IC_50_ (95% CI) | - | - | - | - | <LLOQ (<LLOQ; <LLOQ) | <LLOQ (<LLOQ; <LLOQ) |
| Responder, n/N^c^ (%) | - | - | - | - | 0/13 (0.0) | 0/2 (0.0) |
| 95% CI | - | - | - | - | (0.0; 24.7) | (0.0; 84.2) |
| **Day 57** |  |  |  |  |  |  |
| N | 15 | 14 | 0 | 5 | 0 | 0 |
| GMT, IC_50_ (95% CI) | <LLOQ (<LLOQ; <LLOQ) | <LLOQ (<LLOQ; <LLOQ) | - | <LLOQ (<LLOQ; <LLOQ) | - | - |
| Responder, n/N^c^ (%) | 0/15 (0.0) | 0/14 (0.0) | - | 0/5 (0.0) | - | - |
| 95% CI | (0.0; 21.8) | (0.0; 23.2) | - | (0.0; 52.2) | - | - |
| **Day 71** |  |  |  |  |  |  |
| N | 15 | 14 | 0 | 5 | 0 | 0 |
| GMT, IC_50_ (95% CI) | 71 (<LLOQ; 163) | <LLOQ (<LLOQ; <LLOQ) | - | <LLOQ (<LLOQ; <LLOQ) | - | - |
| Responder, n/N^c^ (%) | 7/15 (46.7) | 0/14 (0.0) | - | 0/5 (0.0) | - | - |
| 95% CI | (21.3; 73.4) | (0.0; 23.2) | - | (0.0; 52.2) | - | - |
| **Day 78** |  |  |  |  |  |  |
| N | 14 | 14 | 0 | 5 | 0 | 0 |
| GMT, IC_50_ (95% CI) | 72 (<LLOQ; 143) | <LLOQ (<LLOQ; <LLOQ) | - | <LLOQ (<LLOQ; <LLOQ) | - | - |
| Responder, n/N^c^ (%) | 8/14 (57.1) | 1/14 (7.1) | - | 0/5 (0.0) | - | - |
| 95% CI | (28.9; 82.3) | (0.2; 33.9) | - | (0.0; 52.2) | - | - |
| **Day 92** |  |  |  |  |  |  |
| N | 15 | 14 | 0 | 5 | 0 | 0 |
| GMT, IC_50_ (95% CI) | <LLOQ (<LLOQ; 63) | <LLOQ (<LLOQ; <LLOQ) | - | <LLOQ (<LLOQ; <LLOQ) | - | - |
| Responder, n/N^c^ (%) | 3/15 (20.0) | 0/14 (0.0) | - | 0/5 (0.0) | - | - |
| 95% CI | (4.3; 48.1) | (0.0; 23.2) | - | (0.0; 52.2) | - | - |
| **Day 180** |  |  |  |  |  |  |
| N | 14 | 14 | 0 | 5 | 8 | 0 |
| GMT, IC_50_ (95% CI) | <LLOQ (<LLOQ; <LLOQ) | <LLOQ (<LLOQ; <LLOQ) | - | <LLOQ (<LLOQ; <LLOQ) | <LLOQ (<LLOQ; <LLOQ) | - |
| Responder, n/N^c^ (%) | 0/14 (0.0) | 0/14 (0.0) | - | 0/5 (0.0) | 0/8 (0.0) | - |
| 95% CI | (0.0; 23.2) | (0.0; 23.2) | - | (0.0; 52.2) | (0.0; 36.9) | - |
| **Day 360** |  |  |  |  |  |  |
| N | 14 | 13 | 0 | 0 | 8 | 0 |
| GMT, IC_50_ (95% CI) | <LLOQ (<LLOQ; <LLOQ) | <LLOQ (<LLOQ; <LLOQ) | - | - | <LLOQ (<LLOQ; <LLOQ) | - |
| Responder, n/N^c^ (%) | 0/14 (0.0) | 0/13 (0.0) | - | - | 0/8 (0.0) | - |
| 95% CI | (0.0; 23.2) | (0.0; 24.7) | - | - | (0.0; 36.9) | - |

CI = confidence interval; GMT = geometric mean titer; GP = glycoprotein; IC_50_ = 50% inhibitory concentration; Inf U = infectious units; LLOQ = lower limit of quantification (40 IC_50_); MARV = *Marburg virus*; MVA = MVA-BN-Filo; N = number of participants with data at that time point; psVNA = pseudovirion neutralization assay.

^a^MVA-BN-Filo at a dose of 1x10^8^ Inf U; ^b^Includes all Group 3 participants up to and including the Day 50 time point, after which point only Group 3 participants who did not receive a booster vaccination with vaccine/placebo on Day 92 are included; ^c^Number of participants with data at baseline and at that time point.

Responder rates are shown with corresponding Exact Clopper-Pearson 95% CIs.

**Table 14. MARV GP-specific neutralizing antibody responses (psVNA): descriptive statistics and responder rates; immunogenicity analysis set; Group 3 booster subset.**

|  | | **14-day interval** | | |
| --- | --- | --- | --- | --- |
|  | | MVA, Ad26.Filo, Ad26.Filo |  | Placebo, placebo, placebo |
| **Baseline** |  |  |  |  |
| N |  | 5 |  | 2 |
| GMT, IC_50_ (95% CI) |  | <LLOQ (<LLOQ; <LLOQ) |  | <LLOQ (<LLOQ; <LLOQ) |
| **Day 15** |  |  |  |  |
| N |  | 5 |  | 2 |
| GMT, IC_50_ (95% CI) |  | <LLOQ (<LLOQ; <LLOQ) |  | <LLOQ (<LLOQ; <LLOQ) |
| Responder, n/N^a^ (%) |  | 0/5 (0.0) |  | 0/2 (0.0) |
| 95% CI |  | (0.0; 52.2) |  | (0.0; 84.2) |
| **Day 36** |  |  |  |  |
| N |  | 5 |  | 2 |
| GMT, IC_50_ (95% CI) |  | <LLOQ (<LLOQ; <LLOQ) |  | <LLOQ (<LLOQ; <LLOQ) |
| Responder, n/N^a^ (%) |  | 0/5 (0.0) |  | 0/2 (0.0) |
| 95% CI |  | (0.0; 52.2) |  | (0.0; 84.2) |
| **Day 50** |  |  |  |  |
| N |  | 5 |  | 2 |
| GMT, IC_50_ (95% CI) |  | <LLOQ (<LLOQ; <LLOQ) |  | <LLOQ (<LLOQ; <LLOQ) |
| Responder, n/N^a^ (%) |  | 0/5 (0.0) |  | 0/2 (0.0) |
| 95% CI |  | (0.0; 52.2) |  | (0.0; 84.2) |
| **Day 92** |  |  |  |  |
| N |  | 5 |  | 2 |
| GMT, IC_50_ (95% CI) |  | <LLOQ (<LLOQ; 91) |  | <LLOQ (<LLOQ; <LLOQ) |
| Responder, n/N^a^ (%) |  | 1/5 (20.0) |  | 0/2 (0.0) |
| 95% CI |  | (0.5; 71.6) |  | (0.0; 84.2) |
| **Day 99** |  |  |  |  |
| N |  | 5 |  | 2 |
| GMT, IC_50_ (95% CI) |  | 51 (<LLOQ; 275) |  | <LLOQ (<LLOQ; <LLOQ) |
| Responder, n/N^a^ (%) |  | 2/5 (40.0) |  | 0/2 (0.0) |
| 95% CI |  | (5.3; 85.3) |  | (0.0; 84.2) |
| **Day 113** |  |  |  |  |
| N |  | 5 |  | 2 |
| GMT, IC_50_ (95% CI) |  | 55 (<LLOQ; 313) |  | <LLOQ (<LLOQ; <LLOQ) |
| Responder, n/N^a^ (%) |  | 2/5 (40.0) |  | 0/2 (0.0) |
| 95% CI |  | (5.3; 85.3) |  | (0.0; 84.2) |
| **Day 180** |  |  |  |  |
| N |  | 5 |  | 2 |
| GMT, IC_50_ (95% CI) |  | <LLOQ (<LLOQ; 78) |  | <LLOQ (<LLOQ; <LLOQ) |
| Responder, n/N^a^ (%) |  | 1/5 (20.0) |  | 0/2 (0.0) |
| 95% CI |  | (0.5; 71.6) |  | (0.0; 84.2) |
| **Day 360** |  |  |  |  |
| N |  | 5 |  | 0 |
| GMT, IC_50_ (95% CI) |  | <LLOQ (<LLOQ; <LLOQ) |  | - |
| Responder, n/N^a^ (%) |  | 0/5 (0.0) |  | - |
| 95% CI |  | (0.0; 52.2) |  | - |

CI = confidence interval; GMT = geometric mean titer; GP = glycoprotein; IC_50_ = 50% inhibitory concentration; LLOQ = lower limit of quantification (40 IC_50_); MARV = *Marburg virus*; MVA = MVA-BN-Filo; N = number of participants with data at that time point; psVNA = pseudovirion neutralization assay.

^a^Number of participants with data at baseline and at that time point.

All time points include only Group 3 participants who receive a booster vaccination on Day 92.

**Fig 1. Study design.**

MVA = MVA-BN-Filo; vp = viral particles; Inf U = infectious units; N = number of participants in a group; n = number of participants to receive study vaccine (active or placebo).

^a^Only a subset of participants (5:2, active:placebo) received a booster vaccination on Day 92.

^b^MVA-BN-Filo at a dose of 1x10^8^ Inf U.

^c^A subset of participants in Group 3 was enrolled in a substudy with a third dose on Day 92. It was planned that the first 8 participants who were willing to participate in the substudy would receive a third dose on Day 92 based on the previous vaccination. After the enrollment of 8 participants, the unblinded monitor and unblinded pharmacist would assess whether 7 participants who had received MVA-BN-Filo 5x10^8^ Inf U prime/Ad26.Filo boost were enrolled. If less than 7 participants of the active regimen were enrolled, 2 additional participants were enrolled. If at least 7 participants of the active regimen were enrolled, no additional participants were enrolled. Participants who had received placebo received placebo for a third time.


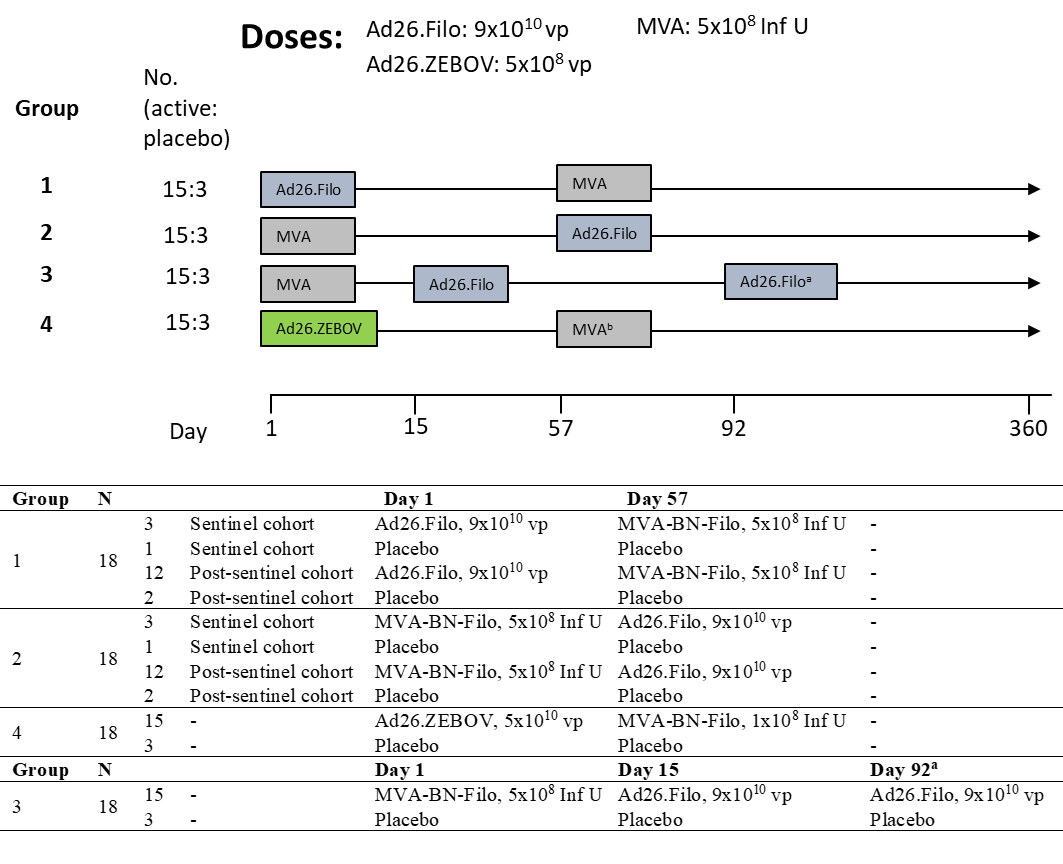


**Fig 2.** **CD4+ T cell cytokine responses (ICS): EBOV GP.**

Blue closed circles represent measurements from individual participants. Median values per time point are indicated by thick horizontal lines, and whiskers denote the IQR.

EBOV = *Zaire ebolavirus*; GP = glycoprotein; ICS = intracellular cytokine staining; Inf U = infectious units; IQR = interquartile range; Ad26.F = Ad26.Filo; Ad26.Z = Ad26.ZEBOV; MVA = MVA-BN-Filo; NA = not applicable.

^a^Includes all Group 3 participants up to and including the Day 50 time point, after which point only Group 3 participants who did not receive a third dose of vaccine/placebo on Day 92 are included.

^b^MVA-BN-Filo at a dose 1x10^8^ Inf U.

^c^All time points include only Group 3 participants who receive a booster vaccination with vaccine/placebo on Day 92.





**Fig 3.** **CD8+ T cell cytokine responses (ICS): EBOV GP.**

Blue closed circles represent measurements from individual participants. Median values per time point are indicated by thick horizonal lines, and whiskers denote the IQR.

EBOV = *Zaire ebolavirus*; GP = glycoprotein; ICS = intracellular cytokine staining; Inf U = infectious units; IQR = interquartile range; Ad26.F = Ad26.Filo; Ad26.Z = Ad26.ZEBOV; MVA = MVA-BN-Filo; NA = not applicable.

^a^Includes all Group 3 participants up to and including the Day 50 time point, after which point only Group 3 participants who did not receive a third dose of vaccine/placebo on Day 92 are included.

^b^MVA-BN-Filo at a dose 1x10^8^ Inf U.

^c^All time points include only Group 3 participants who receive a booster vaccination with vaccine/placebo on Day 92.





**Fig 4.** **CD4+ T cell cytokine responses (ICS): SUDV GP.**

Blue closed circles represent measurements from individual participants. Median values per time point are indicated by thick horizonal lines and whiskers denote the IQR.

GP = glycoprotein; ICS = intracellular cytokine staining; Inf U = infectious units; IQR = interquartile range; Ad26.F = Ad26.Filo; Ad26.Z = Ad26.ZEBOV; MVA = MVA-BN-Filo; NA = not applicable; SUDV = *Sudan ebolavirus*.

^a^Includes all Group 3 participants up to and including the Day 50 time point, after which point only Group 3 participants who did not receive a third dose of vaccine/placebo on Day 92 are included.

^b^MVA-BN-Filo at a dose 1x10^8^ Inf U.

^c^All time points include only Group 3 participants who receive a booster vaccination with vaccine/placebo on Day 92.





**Fig 5.** **CD8+ T cell cytokine responses (ICS): SUDV GP.**

Blue closed circles represent measurements from individual participants. Median values per time point are indicated by thick horizonal lines and whiskers denote the IQR.

GP = glycoprotein; ICS = intracellular cytokine staining; Inf U = infectious units; IQR = interquartile range; Ad26.F = Ad26.Filo; Ad26.Z = Ad26.ZEBOV; MVA = MVA-BN-Filo; NA = not applicable; SUDV = *Sudan ebolavirus*.

^a^Includes all Group 3 participants up to and including day 50; all time points after this exclude participants who received the Ad26.F/placebo booster dose on Day 92.

^b^MVA-BN-Filo at a dose 1x10^8^ Inf U.

^c^All time points include only Group 3 participants who receive a booster vaccination with vaccine/placebo on Day 92.





**Fig 6.** **CD4+ T cell cytokine responses (ICS): MARV GP.**

Blue closed circles represent measurements from individual participants. Median values per time point are indicated by thick horizonal lines and whiskers denote the IQR.

GP = glycoprotein; ICS = intracellular cytokine staining; Inf U = infectious units; IQR = interquartile range; MARV = *Marburg virus*; Ad26.F = Ad26.Filo; Ad26.Z = Ad26.ZEBOV; MVA = MVA-BN-Filo; NA = not applicable.

^a^Includes all Group 3 participants up to and including the Day 50 time point, after which point only Group 3 participants who did not receive a third dose of vaccine/placebo on Day 92 are included.

^b^MVA-BN-Filo at a dose 1x10^8^ Inf U.

^c^All time points include only Group 3 participants who receive a booster vaccination with vaccine/placebo on Day 92.





**Fig 7.** **CD8+ T cell cytokine responses (ICS): MARV GP.**

Blue closed circles represent measurements from individual participants. Median values per time point are indicated by thick horizonal lines and whiskers denote the IQR.

GP = glycoprotein; ICS = intracellular cytokine staining; Inf U = infectious units; IQR = interquartile range; MARV = *Marburg virus*; Ad26.F = Ad26.Filo; Ad26.Z = Ad26.ZEBOV; MVA = MVA-BN-Filo; NA = not applicable.

^a^Includes all Group 3 participants up to and including the Day 50 time point, after which point only Group 3 participants who did not receive a third dose of vaccine/placebo on Day 92 are included.

^b^MVA-BN-Filo at a dose 1x10^8^ Inf U.

^c^All time points include only Group 3 participants who receive a booster vaccination with vaccine/placebo on Day 92.
