# Supplementary material for: First-in-human study to evaluate safety, tolerability, and immunogenicity of heterologous regimens using the multivalent filovirus vaccines Ad26.Filo and MVA-BN-Filo administered in different sequences and schedules: A randomized, controlled study
Source: PLoS One. 2022 Oct 5;17(10):e0274906. doi: 10.1371/journal.pone.0274906 (PMC9534391; doi:10.1371/journal.pone.0274906)
Supplement: S1 Protocol — (PDF) [file pone.0274906.s002.pdf]

Janssen Vaccines & Prevention B.V.*

Clinical Protocol

A Phase 1, First-in-human Study to Evaluate Safety, Tolerability, and Immunogenicity of Heterologous Prime-boost Regimens Using the Multivalent Filovirus Vaccines Ad26.Filo and MVA-BN-Filo Administered in Different Sequences and Schedules in Healthy Adults

Protocol VAC69120FLV1001; Phase 1
AMENDMENT 4

DMID Protocol Number: 15-0056 (Version 6.0)

DMID Funding Mechanism: Contract HHSN272200800056C

VAC69120 (Ad26.Filo/MVA-BN-Filo)

* Janssen Vaccines & Prevention B.V. (formerly known as Crucell Holland B.V.) is a Janssen pharmaceutical company of Johnson & Johnson and is hereafter referred to as the sponsor of the study.

This study will be conducted under
US Food & Drug Administration IND regulations (21 CFR Part 312).

Status: Approved
Date: 12 December 2017
EDMS number: EDMS-ERI-108202139, 15.0

GCP Compliance: This study will be conducted in compliance with Good Clinical Practice, and applicable regulatory requirements.

**Protocol History<br>VAC69120FLV1001_Protocol**		
Document Type and<br>*File Name*	Issued Date	Comments
Initial Clinical Protocol<br>*VAC69120FLV1001_Protocol*	13 July 2016	-
Protocol Amendment 1<br>*VAC69120FLV1001_Protocol_Amend_1*	31 August 2016	For details, see Section **Amendment 1**
Protocol Amendment 2<br>*VAC69120FLV1001 Protocol_Amend_2*	10 January 2017	For details, see Section **Amendment 2**
Protocol Amendment 3<br>*VAC69120FLV1001 Protocol_Amend_3*	24 February 2017	For details, see Section **Amendment 3**
Protocol Amendment 4<br>*VAC69120FLV1001 Protocol_Amend_4*	This document	For details, see Section **Amendment 4**

TABLE OF CONTENTS

TABLE OF CONTENTS 3

LIST OF IN-TEXT TABLES 5

PROTOCOL AMENDMENTS 6

SYNOPSIS 11

TIME AND EVENTS SCHEDULE 18

ABBREVIATIONS 24

DEFINITION OF TERMS 25

1. INTRODUCTION 26
1.1. Background 26
1.1.1. Nonclinical Studies 27
1.1.1.1. Immunogenicity and Efficacy 27
1.1.1.2. Toxicology 29
1.1.1.3. Biodistribution 30
1.1.2. Clinical Studies 30
1.1.2.1. Safety Profile of Ad26-based Vaccines 30
1.1.2.1.1. Pooled Safety Data From Studies VAC52150EBL1001 and VAC52150EBL1002 30
1.1.2.1.2. Relevant Safety Information from Ongoing VAC52150 Studies 35
1.1.2.1.3. Safety Data From Other Ad26-based Vaccines. 35
1.1.2.2. Safety Profile of MVA-BN-based Vaccines 36
1.1.2.3. Viral Shedding 36
1.1.2.4. Immunogenicity 37
1.2. Overall Rationale for the Study 38

2. OBJECTIVES, ENDPOINTS, AND HYPOTHESIS 39
2.1. Objectives and Endpoints 39
2.1.1. Objectives 39
2.1.2. Endpoints 40
2.2. Hypothesis 41

3. STUDY DESIGN AND RATIONALE 41
3.1. Overview of Study Design 41
3.2. Study Design Rationale 44

4. SUBJECT POPULATION 46
4.1. Inclusion Criteria 46
4.2. Exclusion Criteria 49
4.3. Prohibitions and Restrictions 52

5. TREATMENT ALLOCATION AND BLINDING 53

6. DOSAGE AND ADMINISTRATION 54

7. TREATMENT COMPLIANCE 55

8. PRESTUDY AND CONCOMITANT THERAPY 55

9. STUDY EVALUATIONS 55
9.1. Study Procedures 55
9.1.1. Overview 55
9.1.2. Visit Windows 56
9.1.3. Screening 57

9.1.4. Vaccination 59
9.1.5. Post-vaccination 61
9.1.6. Early Withdrawal 61
9.2. Immunogenicity Evaluations 61
9.3. Safety Evaluations 62
9.4. Vaccine-induced Seropositivity 67
9.5. Sample Collection and Handling 67

10. SUBJECT COMPLETION/DISCONTINUATION OF STUDY TREATMENT/ WITHDRAWAL FROM THE STUDY 68
10.1. Completion 68
10.2. Discontinuation of Study Vaccine/Withdrawal From the Study 68
10.3. Contraindications to Vaccination 69
10.4. Withdrawal From the Use of Research Samples 70

11. STATISTICAL METHODS 70
11.1. Analysis Sets 70
11.2. Sample Size Determination 70
11.3. Subject Information 71
11.4. Immunogenicity Analyses 71
11.5. Safety Analyses 72
11.6. SMC 73
11.7. Pausing Rules 74
11.7.1. Pausing Rules for Prime Dose in Sentinel Cohorts of Groups 1 and 2 74
11.7.2. Pausing Rules for Prime Dose in Post-sentinel Cohorts of Groups 1 and 2 74
11.7.3. Pausing Rules for Boost Dose in Sentinel Cohorts of Groups 1 and 2 76
11.7.4. Pausing Rules for Boost Dose in Post-sentinel Cohorts of Groups 1 and 2 76
11.7.5. Pausing Rules for Prime and Boost Dose in Group 3 77
11.7.6. Criteria for Discontinuation From Boost Administration to an Individual Subject 78

12. ADVERSE EVENT REPORTING 79
12.1. Definitions 79
12.1.1. AE Definitions and Classifications 79
12.1.2. Attribution Definitions 82
12.1.3. Severity Criteria 82
12.2. Special Reporting Situations 82
12.3. Procedures 83
12.3.1. All AEs 83
12.3.2. SAEs 84
12.3.3. Immediate Reportable Events 85
12.3.4. Pregnancy 85
12.4. Contacting Sponsor Regarding Safety 86

13. PRODUCT QUALITY COMPLAINT HANDLING 86
13.1. Procedures 86
13.2. Contacting Sponsor Regarding Product Quality 86

14. STUDY VACCINE INFORMATION 87
14.1. Physical Description of Study Vaccines 87
14.2. Packaging and Labeling 89
14.3. Preparation, Handling, and Storage 89
14.4. Study Vaccine Accountability 90

15. STUDY-SPECIFIC MATERIALS 90

16. ETHICAL ASPECTS 91
16.1. Study-specific Design Considerations 91
16.2. Regulatory Ethics Compliance 93
16.2.1. Investigator Responsibilities 93

16.2.2. Independent Ethics Committee and Institutional Review Board 93
16.2.3. Informed Consent 95
16.2.4. Privacy of Personal Data 95
16.2.5. Long-term Retention of Samples for Additional Future Research 96
16.2.6. Country Selection 96
16.3. Public Readiness and Emergency Preparedness Act 96

17. ADMINISTRATIVE REQUIREMENTS 97
17.1. Protocol Amendments 97
17.2. Regulatory Documentation 97
17.2.1. Regulatory Approval/Notification 97
17.2.2. Required Prestudy Documentation 97
17.3. Subject Identification, Enrollment, and Screening Logs 98
17.4. Source Documentation 99
17.5. Case Report Form Completion and Data Handling 99
17.6. Data Quality Assurance/Quality Control 100
17.7. Record Retention 101
17.8. Protocol Deviations 101
17.9. Monitoring 102
17.10. Study Completion/Termination 102
17.10.1. Study Completion/End of Study 102
17.10.2. Study Termination 103
17.11. On-site Audits 103
17.12. Use of Information and Publication 103

REFERENCES 105

ATTACHMENTS 107

INVESTIGATOR AGREEMENT 112

LAST PAGE 113

LIST OF IN-TEXT TABLES

TABLES

Table 1: Vaccination Schedules 43
Table 2: Visit Windows 57
Table 3: Summary of Immunogenicity Assays (Humoral [Serology]) 62
Table 4: Summary of Immunologic Assays (Cellular) 62
Table 5: Probability of Observing ≥1 AE Given a True AE Incidence 71

PROTOCOL AMENDMENTS

Amendments below are listed beginning with the most recent amendment.

Amendment 4 (12 December 2017)

The overall reason for the amendment: This amendment is developed in response to changes to the global clinical development plan. As a result, as of this amendment, subjects in this study will no longer be approached to roll-over to the VAC52150EBL4001 study.

The changes made to the clinical protocol VAC69120FLV1001 Amendment 3 (dated 24 February 2017) are listed below, including the rationale of each change and a list of all applicable sections.

Rationale: The original development plan (at the time of the ongoing Ebola epidemic in Africa) was an accelerated plan with the anticipation of conducting Phase 3 efficacy studies (with limited safety data collection) shortly after Phase 1 and in parallel with Phase 2. The sponsor designed the VAC52150EBL4001 study for the extended follow-up of serious adverse events to enhance the ability for signal detection of rare events. Since there is no longer an ongoing Ebola epidemic, it is currently not possible to conduct a parallel Phase 3 efficacy study as part of an accelerated development plan. The ongoing Phase 2 and 3 studies will continue long-term follow-up in a placebo-controlled manner until agreement to unblind has been achieved. Placebo-controlled blinded data during long-term follow-up will provide high quality data on long-term safety and will supplement the open-label registry data. As a result, as of this amendment, subjects in this study will no longer be approached to roll-over to the VAC52150EBL4001 study.

SYNOPSIS
3.1 Overview of Study Design
9.1.6 Rollover Study

Amendment 3 (24 February 2017)

The overall reason for the amendment: This amendment is developed in response to emerging clinical data of the monovalent vaccine program.
The changes made to the clinical protocol VAC69120FLV1001 Amendment 2 (dated 10 January 2017) are listed below, including the rationale of each change and a list of all applicable sections.

Rationale: Immunogenicity data from a Phase 1 study of the monovalent vaccine program (VAC52150EBL1002) demonstrated excellent stability of the binding antibody response out to one year following prime-boost regimens with Ad26.ZEBOV/MVA-BN-Filo (or the reverse). A third vaccination using Ad26.ZEBOV at $5x10^{10}$ vp was given at the one year time point leading to a marked and rapid increase in the binding antibody responses within 7 days (at least 10-fold). The marked and rapid rise was generally independent of the antecedent prime-boost regimen. The profile of the antibody response strongly suggest that robust anamnestic responses can be induced after re-exposure to an EBOV antigen, in this case mimicked by a third vaccination.
This amendment proposes administration of a third vaccination using Ad26.Filo at Day 92 post-prime. The aim is to enroll 7 or 8 subjects who will receive Ad26.Filo as the third vaccination in order to explore whether this anamnestic response might also be induced by the multivalent vaccine program.

SYNOPSIS
Time and Events Schedule
Definition of Terms
1.1 Background
1.2 Overall Rationale for the Study
2.1.1 Objectives
2.1.2 Endpoints
3.1 Overview of Study Design
3.2 Study Design Rationale
4.2 Exclusion Criteria
5 TREATMENT ALLOCATION AND BLINDING
6 DOSAGE AND ADMINISTRATION
8 PRESTUDY AND CONCOMITANT THERAPY
9.1.1 Overview
9.1.2 Visit Windows
9.1.4 Vaccination
9.1.5 Post-vaccination
9.3 Safety Evaluations
10.2 Discontinuation of Study Vaccine/Withdrawal From the Study
10.3 Contraindications to Vaccination
11 STATISTICAL METHODS
11.4 Immunogenicity Analyses
11.5 Safety Analyses
11.7.5 Pausing Rules for Prime and Boost Dose in Group 3
11.7.6 Criteria for Discontinuation From Boost Administration to an Individual Subject
12.3.1 All AEs
16.1 Study-specific Design Considerations

Rationale: The possibility to perform additional interim analyses at the sponsor's discretion was added.

SYNOPSIS
3.1 Overview of Study Design
11 STATISTICAL METHODS

Rationale: Changes were made to follow-up the unsolicited adverse events (AEs) until 28 days post last dose to be consistent with the AE follow-up period post-prime and to align with the standard follow-up periods for vaccines.

SYNOPSIS
Time and Events Schedule
2.1.2 Endpoints
3.1 Overview of Study Design
9.3 Safety Evaluations
12.3.1 All AEs

Rationale: Minor editorial changes have been made.

Time and Events Schedule
SYNOPSIS
2.1.2 Endpoints
3.1 Overview of Study Design
11.5 Safety Analyses
11.6 SMC
12.1.1 AE Definitions and Classifications

Amendment 2 (10 January 2017)

The overall reason for the amendment: The changes in this amendment are being made to clarify that when a laboratory value falls between the local laboratory normal reference range and a grade 1 FDA toxicity category, the subject will remain eligible for inclusion in the study if the laboratory value is within the normal range, while having a grade 1 toxicity.
In this amendment one secondary objective has been revised and shifted to exploratory objective.

The changes made to the clinical protocol VAC69120FLV1001 Amendment 1 (dated 31 August 2016) are listed below, including the rationale of each change and a list of all applicable sections.

Rationale: This amendment resolves a potential misunderstanding about subject's eligibility for inclusion in the study when screening laboratory values are within the local laboratory reference ranges but overlap with the FDA toxicity table. Subjects are eligible for inclusion even if their screening laboratory values overlap with the FDA toxicity table as long as the value is within the laboratory's normal reference range.

4.1 Inclusion Criteria
Attachment 1: Toxicity Grading Scale Adapted from the FDA Toxicity Grading Scale for Healthy Adult and Adolescent Volunteers Enrolled in Preventive Vaccine Clinical Trials

Rationale: The secondary objective to compare humoral immune responses of the monovalent Ad26.ZEBOV regimen (group 4) to the response levels in the multivalent program has been revised and shifted to exploratory objective.

SYNOPSIS
2.1.1 Objectives

Rationale: The concept of “overdose” was erased across the Janssen vaccines development platform for products only under development (ie, no Company Core Data Sheet/Label).

12.2 Special Reporting Situations

Rationale: The protocol has been updated in line with the current protocol template (version 1 November 2016).

4.1 Inclusion Criteria
10.2 Discontinuation of Study Vaccine/Withdrawal From the Study
17.5 Case Report Form Completion and Data Handling

Rationale: Minor textual changes have been made, in addition to modifications for clarity and updates to be in line with other current protocols.

SYNOPSIS
ABBREVIATIONS
2.1.1 Objectives
2.1.2 Endpoints
4.2 Exclusion Criteria
12.1.1 AE Definitions and Classifications
14.1 Physical Description of Study Vaccines
16.2.6 Country Selection

Amendment 1 (31 August 2016)

The overall reason for the amendment: The changes in this amendment are being made in response to feedback received from the US Food and Drug Administration (FDA) and in response to the evolving safety profile of Ad26.ZEBOV. In addition, some changes have been added for clarification.

The changes made to the clinical protocol VAC69120FLV1001 (dated 13 July 2016) are listed below, including the rationale of each change and a list of all applicable sections.

**Rationale:** As requested by the FDA, oropharyngeal pain has been added as unsolicited AE (post-prime event) reported with a frequency of ≥5% in the MVA-BN-Filo $1x10^8$ Inf U group of the pooled safety data from studies VAC52150EBL1001 and VAC52150EBL1002.
1.1.2.1.1 Pooled Safety Data From Studies VAC52150EBL1001 and VAC52150EBL1002
**Rationale:** Relevant safety information from ongoing VAC52150 studies has been updated.
1.1.2.1.2 Relevant Safety Information from Ongoing VAC52150 Studies
**Rationale:** It has been clarified that only 1 FSH test is required at screening for women of any age with amenorrhea for >6 months but <12 months as this is deemed sufficient to determine a post menopausal state.
Time and Events Schedule<br>4.1 Inclusion Criteria
**Rationale:** In the inclusion criteria, "Fibrinogen: within laboratory normal range" has been removed from the list of acceptable laboratory parameters. Fibrinogen is an acute phase reactant and a random, single screening assessment in an otherwise healthy subject would only increase the screen failure rate without excluding subjects at an elevated risk. This parameter was deleted by amendment from a prior Ebola vaccine study using the MVA-BN-Filo component without jeopardizing the subjects' safety.
4.1 Inclusion Criteria<br>9.3 Safety Evaluations
**Rationale:** The inclusion criteria related to total white blood cell count has been updated to allow enrollment of subjects with ethnic neutropenia, which is not a pathophysiologic process.
4.1 Inclusion Criteria
**Rationale:** The text has been updated to reflect that the Phase 3 clinical study NCT1144637 has been completed.
1.1.2.2 Safety Profile of MVA-BN-based Vaccines
**Rationale:** As requested by the FDA, the pausing rules for boost dose in post-sentinel cohorts of Groups 1 and 2 have been clarified. Administration of the boost dose will be halted if one or more subjects experience anaphylaxis within 24 hours, or generalized urticaria within 72 hours after the boost vaccination.
11.7.4 Pausing Rules for Boost Dose in Post-sentinel Cohorts of Groups 1 and 2
**Rationale:** The list of events of neuroimmunologic significance in Section 12.3.3 has been replaced by a reference to the relevant section where this list is also presented.
12.3.3 Immediate Reportable Events

SYNOPSIS

A Phase 1, First-in-human Study to Evaluate Safety, Tolerability, and Immunogenicity of Heterologous Prime-boost Regimens Using the Multivalent Filovirus Vaccines Ad26.Filo and MVA-BN-Filo Administered in Different Sequences and Schedules in Healthy Adults

OVERALL RATIONALE

In this first-in-human study, a multivalent vaccine comprising 3 adenovirus serotype 26 (Ad26) vectors expressing the glycoproteins (GPs) of the Ebola virus (EBOV) Mayinga variant (Ad26.ZEBOV), the Marburg virus (MARV) Angola variant (Ad26.MARVA), and the Sudan virus (SUDV) Gulu variant (Ad26.SUDV) (together known as **Ad26.Filo**) and the Modified Vaccinia Ankara (MVA) - Bavarian Nordic (BN) vector expressing the GPs of EBOV, MARV, and SUDV and the nucleoprotein of Taï Forest virus (TAFV) (**MVA-BN-Filo**) will be evaluated as a heterologous prime-boost regimen, in which one study vaccine is used to prime a filovirus-specific immune response and the other study vaccine is used to boost the immune response. MVA-BN-Filo prime will be boosted by Ad26.Filo 14 or 56 days later; Ad26.Filo prime will be boosted by MVA-BN-Filo 56 days later. In addition, a group assessing the monovalent Ad26.ZEBOV prime at Day 1/MVA-BN-Filo boost at Day 57 will be included in the study to serve as a tolerability and immunogenicity control arm, as this heterologous regimen has already been assessed in clinical studies.

Recent clinical data from a Phase 1 study of the monovalent vaccine program (VAC52150EBL1002) exploring the safety, tolerability, and immunogenicity of a third vaccination (Ad26.ZEBOV) given at one year post-prime vaccination demonstrated a marked induction of binding antibody responses to the EBOV glycoprotein. A log increase in antibody titers within 7 days of administration of the third vaccination was observed, suggesting that the first 2 vaccinations induced a strong memory response that gave rise to a robust anamnestic response upon re-exposure to antigen mimicked through the third vaccination. It is unknown whether a similar effect would be observed in this study for subjects of Group 3 given Ad26.Filo as a third vaccination at Day 92 post-prime, approximately 2.5 months (77 days post-boost) after the boost vaccination of Ad26.Filo. To explore whether a similar anamnestic antibody response to the 3 glycoproteins, namely Ebola, Sudan, and Marburg virus GP would be generated in the multivalent vaccine program, a third vaccination (Ad26.Filo) will be given at Day 92 post-prime to a subset of subjects in Group 3.

OBJECTIVES, ENDPOINTS, AND HYPOTHESIS

OBJECTIVES

Primary Objectives

To assess the safety and tolerability of:

- A prime vaccination with Ad26.Filo at the dose of $9x10^{10}$ viral particles (vp) boosted by MVA-BN-Filo at the dose of $5x10^{8}$ infectious units (Inf U) 56 days later.
- A prime dose of MVA-BN-Filo at the dose of $5x10^{8}$ Inf U boosted by Ad26.Filo at the dose of $9x10^{10}$ vp either 14 or 56 days later.
- A third vaccination using Ad26.Filo at the dose of $9x10^{10}$ vp at Day 92.

Secondary Objectives

- To assess humoral immune responses of the 3 multivalent filovirus regimens tested, as measured by enzyme-linked immunosorbent assay (ELISA) against each GP insert.

Exploratory Objectives

- To assess humoral and cellular immune responses of the regimens tested, including but not limited to, virus neutralization assay, functional and/or biophysical characterization of antibody responses, enzyme-linked immunospot assay (ELISpot), and intracellular cytokine staining (ICS).
- To assess humoral immune responses, as measured by ELISA to EBOV GP, of the monovalent Ad26.ZEBOV regimen (group 4) to evaluate if response levels of the multivalent program are consistent with the ones observed in the monovalent Ad26.ZEBOV clinical studies.

ENDPOINTS

Primary Endpoints

The safety and tolerability endpoints are:

- Unsolicited adverse events (AEs) from signing of the Informed Consent Form (ICF) onwards until 28 days post-boost, and again from the third vaccination until 28 days thereafter (for a subset of subjects in Group 3) (note: events that started before the third vaccination but are still present at the time of third vaccination should also be recorded).
- Solicited local and systemic AEs (reactogenicity) until 7 days after each study vaccine administration.
- Serious AEs from signing of the ICF onwards until the end of the study.

Secondary Endpoints

Humoral Immune Responses

- Binding antibody responses against EBOV, MARV, and SUDV GPs.

Exploratory Endpoints

Additional exploratory analyses may be performed to further investigate study vaccine-elicited immune responses. Exploratory endpoints may include, but might not be limited to, the following:

Humoral Immune Responses

- Neutralizing antibody responses against EBOV, MARV, and/or SUDV GPs defined as the serum titer that is able to inhibit viral infection (50%, 80%, or 90% inhibitory concentration).
- Binding and/or neutralizing antibody responses against adenoviral vector (Ad) and/or MVA vector.
- Characterization of study vaccine-elicited antibodies.

Cell-mediated Immune Responses

- Presence and functional capacity of T cells after pathogen-specific stimulation of peripheral blood mononuclear cells with EBOV, MARV, and/or SUDV GP-specific peptides (ELISpot and ICS).
- Transcriptome analysis of immune cell populations.

Hypothesis

The study is exploratory, and no formal statistical hypothesis testing is planned.

OVERVIEW OF STUDY DESIGN

This is a Phase 1, randomized, double-blind, placebo-controlled, first-in-human study exploring the safety, tolerability, and immunogenicity of 3 heterologous prime-boost regimens compared with placebo using MVA-BN-Filo at a dose of $5x10^8$ Inf U and Ad26.Filo at a dose of $9x10^{10}$ vp (Groups 1, 2, and 3). In addition, a group assessing a monovalent Ad26.ZEBOV prime at a dose of $5x10^{10}$ vp at Day 1 and

MVA-BN-Filo boost at the dose of $1x10^8$ Inf U at Day 57 (Group 4) will be included in the study to serve as a tolerability and immunogenicity control arm. Placebo recipients are included for blinding purposes and safety analyses, and will provide control specimens for immunogenicity assays. The study will be conducted in healthy adult subjects aged ≥18 to ≤50 years not previously vaccinated with a filovirus candidate vaccine and with no known previous exposure to EBOV, MARV, SUDV, or TAFV. A total of 72 healthy adult male and female subjects are planned to be enrolled.

Additionally, this study will explore whether a third vaccination (Ad26.Filo) given at Day 92 post-prime to a subset of subjects in Group 3 would induce a similar anamnestic antibody response to the 3 glycoproteins, namely Ebola, Sudan and Marburg. The first 8 subjects in Group 3 who are willing to enroll for the third vaccination, will receive a third vaccination at Day 92. In an observer-blind manner, subjects who previously received placebo will receive placebo a third time and subjects who previously received MVA-BN-Filo/Ad26.Filo vaccination will receive Ad26.Filo at the dose of $9x10^{10}$ vp as third vaccination. After enrollment of the 8 subjects, the unblinded monitor and unblinded pharmacist will assess whether 7 subjects who previously received MVA-BN-Filo/Ad26.Filo vaccination have been enrolled. If less than 7 subjects of the active vaccine regimen have been enrolled, 2 additional subjects will be enrolled. If at least 7 subjects of the active vaccine regimen have been enrolled, no further subjects will be enrolled. The aim is to enroll 7 or 8 subjects who will receive Ad26.Filo as third vaccination. Subjects who receive the third vaccination will follow a separate time and events schedule as of Day 92 while the remainder of Group 3 who did not receive the third vaccination would continue in the study according to the original time and event schedule for Group 3.

The study will last approximately 395 days per subject and will consist of a screening period of up to 35 days, a vaccination period in which the subjects will be vaccinated at baseline (Day 1) followed by a boost on Day 15 or 57, and a third vaccination at Day 92 (subset of subjects in Group 3), and a post last dose follow-up until all subjects have had their Day 360 (359 days post-prime) visit or withdrew earlier.

Subjects will be enrolled into 4 different groups of 18 healthy subjects each to receive 1 of the 4 following treatment regimens:

- Ad26.Filo prime at Day 1/MVA-BN-Filo boost at Day 57 (Group 1).
- MVA-BN-Filo prime at Day 1/Ad26.Filo boost at Day 57 (Group 2).
- MVA-BN-Filo prime at Day 1/Ad26.Filo boost at Day 15/a subset of subjects will receive a third vaccination (Ad26.Filo) at Day 92 (Group 3).
- Ad26.ZEBOV prime at Day 1/MVA-BN-Filo boost at Day 57 (Group 4).

Subjects will be randomly assigned within groups to active vaccine or placebo (0.9% normal saline) in a 5:1 ratio (ie, 15 active/3 placebo).

Groups 1 and 2 will start with 4 sentinel subjects in each group (3 active/1 placebo per group). If no pausing rule is met by Day 4 (ie, at least 72 hours after prime vaccination) in the sentinel cohorts, recruitment will open to the remaining subjects in Groups 1 and 2 (12 active/2 placebo per group). The randomization of Group 4 does not depend on the outcome of the sentinel cohorts because the safety profile of this regimen has been assessed in other studies; therefore, Group 4 randomization will start immediately after assignment of the sentinel subjects of Groups 1 and 2 and will be randomized as a whole (15 active/3 placebo). After randomization of Groups 1, 2, and 4, Group 3 will be randomized as a whole (15 active/3 placebo).

Predefined pausing rules will guide study progression under the supervision of an independent Data Monitoring Committee, which will review and evaluate only safety-related data in this study. Therefore, the term Safety Monitoring Committee (SMC) will be used throughout this document.

The different study vaccination schedules are summarized in the table below.

Group	N			Day 1	Day 57	
1	18	3	Sentinel cohort	Ad26.Filo, 9x10^{10} vp	MVA-BN-Filo, 5x10^{8} Inf U	-
		1	Sentinel cohort	Placebo	Placebo	-
		12	Post-sentinel cohort	Ad26.Filo, 9x10^{10} vp	MVA-BN-Filo, 5x10^{8} Inf U	-
		2	Post-sentinel cohort	Placebo	Placebo	-
2	18	3	Sentinel cohort	MVA-BN-Filo, 5x10^{8} Inf U	Ad26.Filo, 9x10^{10} vp	-
		1	Sentinel cohort	Placebo	Placebo	-
		12	Post-sentinel cohort	MVA-BN-Filo, 5x10^{8} Inf U	Ad26.Filo, 9x10^{10} vp	-
		2	Post-sentinel cohort	Placebo	Placebo	-
4	18	15	-	Ad26.ZEBOV, 5x10^{10} vp	MVA-BN-Filo, 1x10^{8} Inf U	-
		3	-	Placebo	Placebo	-
**Group**	**N**			**Day 1**	**Day 15**	**Day 92[a]**
3	18	15	-	MVA-BN-Filo, 5x10^{8} Inf U	Ad26.Filo, 9x10^{10} vp	Ad26.Filo, 9x10^{10} vp
		3	-	Placebo	Placebo	Placebo

Inf U = infectious units; N = number of subjects to receive study vaccine (active vaccine or placebo [0.9% saline]); vp = viral particles.

a. A subset of subjects in Group 3 will be enrolled in a third vaccination substudy at Day 92. The first 8 subjects who are willing to participate will receive a third vaccination at Day 92 based on their previous vaccination. After enrollment of the 8 subjects, the unblinded monitor and unblinded pharmacist will assess whether 7 subjects who previously received MVA-BN-Filo/Ad26.Filo vaccination have been enrolled. If less than 7 subjects of the active vaccine regimen have been enrolled, 2 additional subjects will be enrolled. If at least 7 subjects of the active vaccine regimen have been enrolled, no further subjects will be enrolled.

When all subjects have completed their 21-day post last dose visit (Day 78 in Groups 1, 2, and 4, Day 36 in Group 3, and Day 113 for a subset of Group 3), or withdrew earlier, a primary analysis of all data collected up to that point will be performed. The final analysis will be performed when all subjects in Groups 1 to 4 have completed the last study visit or withdrew earlier. Additional interim analyses may be performed at the sponsor's discretion.

SUBJECT POPULATION

Screening for eligible subjects will be performed within 35 days before administration of the study vaccine (Day 1). Subjects must be healthy (on the basis of physical examination, medical history, and clinical judgment) men and women, aged ≥18 to ≤50 years.

DOSAGE AND ADMINISTRATION

Study vaccines (MVA-BN-Filo, Ad26.Filo, Ad26.ZEBOV) or placebo will be administered as intramuscular injections in either deltoid in the upper arm. For each subject, the boost vaccination should be administered in the opposite deltoid from the prime vaccination unless local site reaction cannot be assessed reliably in the opposite arm, and it should be recorded in the Case Report Form in which arm the vaccination has been administered. The third vaccination can be administered in either deltoid.

Subjects will receive active vaccine or placebo (0.9% normal saline), according to randomization, on Days 1 and 57 (Groups 1, 2, and 4) or on Days 1, 15, and 92 (Group 3, Day 92 only in a subset of subjects) at the following dose levels:

- Ad26.Filo 9x10^{10} vp or placebo on Day 1 (Group 1), Day 57 (Group 2), Day 15 (Group 3) , or Day 92 (subset of Group 3).
- MVA-BN-Filo at 5x10^{8} Inf U or placebo on Day 1 (Groups 2 and 3) or Day 57 (Group 1).
- Ad26.ZEBOV 5x10^{10} vp or placebo on Day 1 (Group 4).
- MVA-BN-Filo 1x10^{8} Inf U or placebo on Day 57 (Group 4).

The Ad26.Filo dose of 9x10^{10} vp was selected for Groups 1, 2, and 3 to be within the range of, but not higher than, the highest dose of Ad26.ZEBOV assessed in humans (1x10^{11} vp), where only mild and

transient AEs were observed. In addition, this Ad26.Filo dose is below the dose of $1.2x10^{11}$ vp tested in non-human primates, where heterologous prime-boost regimens of Ad26- and MVA-based vaccines were well tolerated at doses of up to $1.2x10^{11}$ vp and $5x10^8$ 50% tissue culture infective dose ($TCID_{50}$), respectively. The MVA-BN-Filo dose of $5x10^8$ Inf U was selected for Groups 1, 2, and 3, as this was the dose tested in nonclinical studies in non-human primates, where it was well tolerated. The MVA-BN-Filo dose of $1x10^8$ Inf U was selected for Group 4 (Ad26.ZEBOV prime/MVA-BN-Filo boost) to permit comparison with ongoing clinical studies evaluating heterologous prime-boost regimens of MVA-BN-Filo at the dose of $1x10^8$ Inf U and Ad26.ZEBOV at the dose of $5x10^{10}$ vp.

A 1, 57 day vaccination schedule was selected for Groups 1, 2, and 4 because humoral immune responses were the highest with this schedule in ongoing clinical studies evaluating Ad26.ZEBOV/MVA-BN-Filo prime-boost regimens (with either vaccine as prime or boost). A 1, 15 day vaccination schedule with MVA-BN-Filo as prime and Ad26.Filo as boost was selected for Group 3 because the highest CD8+ T cell responses were achieved in an ongoing clinical study with the MVA-BN-Filo/Ad26.ZEBOV schedule.

IMMUNOGENICITY EVALUATIONS

Immunologic assessments and their purposes are summarized in the tables below. The exploratory assay package may include, but might not be limited to, the listed assays. Sample collection and processing will be performed according to current versions of approved standard operating procedures.

Summary of Immunogenicity Assays (Humoral [Serology])

Assay	Purpose
Secondary endpoint assays	
ELISA	Analysis of antibodies binding to EBOV, MARV, and SUDV GPs
Exploratory endpoint assays	
Virus neutralization assays	Analysis of neutralizing antibodies against EBOV, MARV, and SUDV GPs
Ad and/or MVA ELISA and/or neutralization assays	Binding and/or neutralizing antibodies against Ad and/or MVA
Molecular antibody characterization	Analysis of anti-EBOV GP, anti-MARV GP, and anti-SUDV GP antibody characteristics, including IgG subtyping

Ad = adenoviral vector; EBOV = Ebola virus; ELISA = enzyme-linked immunosorbent assay; GP = glycoprotein; IgG = immunoglobulin G; MARV = Marburg virus; MVA = Modified Vaccinia Ankara; SUDV = Sudan virus.

Summary of Immunologic Assays (Cellular)

Assay	Purpose
Exploratory endpoint assays	
ELISpot	T cell IFN-γ responses to EBOV, MARV, and SUDV GPs
Intracellular cytokine staining	Analysis of T cell responses to EBOV, MARV, and SUDV GPs (including CD4/8+, IL-2, IFN-γ, TNF-α, and/or activation markers)
Transcriptomics	Transcriptome analysis of immune cell populations

CD = cluster of differentiation; EBOV = Ebola virus; ELISpot = enzyme-linked immunospot; GP = glycoprotein; IFN = interferon; IL = interleukin; MARV = Marburg virus; SUDV = Sudan virus; TNF = tumor necrosis factor.

Future scientific research may be conducted using samples obtained in this study to further investigate the study vaccines- and disease-related questions. This may include the development of new or the improvement of existing techniques to characterize EBOV-, MARV- and/or SUDV-directed immune responses or diagnostic tests.

SAFETY EVALUATIONS

After vaccination, subjects will remain at the site for observation and vital sign assessments for at least 60 minutes (or 30 minutes for subjects who received a third vaccination), or longer if deemed necessary by the investigator. Reactogenicity and vital signs will be assessed at 30 (±5) minutes, and vital signs will be checked again at 60 (±5) minutes except for subjects who received a third vaccination. If an event occurs during the 60-minute observation period (or 30-minute observation period for subjects who received a third vaccination), the highest overall grade of the event, across the entire 60 minutes (or 30 minutes for subset of Group 3), will be reported. Subjects will be instructed to contact the investigator immediately if they experience any AE about which they are concerned. Subjects in the sentinel cohorts will be contacted approximately 24 hours after study vaccination to confirm that no pre-specified pausing rules have been met. Subjects will use a diary to record oral body temperature and to document symptoms of unsolicited and solicited local and systemic AEs in the evening after each vaccination and then daily for the next 7 days. If more than 1 measurement is made or if an AE is reported more than once on any given day, the highest value or highest severity of that day will be recorded. The investigator or designee will document unsolicited AEs from signing of the ICF onwards until 28 days post last dose, and serious AEs from signing of the ICF onwards until the end of the study.

Standard chemistry, hematologic, and urinalysis parameters will be assessed before each study vaccine administration and 7 days after each study vaccine administration. Vital signs (including heart rate, blood pressure, and oral body temperature) will be assessed before and 30 (±5) and 60 (±5) minutes (60 minutes not for subjects who received a third vaccination) after each study vaccine administration. A single, 12-lead electrocardiogram will be performed at screening and will only be repeated during the study if deemed necessary by the investigator.

STATISTICAL METHODS

A primary analysis will be performed when all subjects have completed their 21-day post last dose visit (Day 78 in Groups 1, 2, and 4, Day 36 in Group 3, and Day 113 for a subset of Group 3) or withdrew earlier. This analysis will include all available data up to this point.

The final analysis will be performed when all subjects from Groups 1 to 4 have completed the last study visit or withdrew earlier.

Additional interim analyses may be performed at the sponsor's discretion.

The sample size for this study is not based on formal hypothesis testing considerations, but is within the range of subjects recommended in the Code of Federal Regulations (CFR 312.21) for the first-in-human products in this investigation. In each of the 4 groups, 15 subjects will receive active vaccine and 3 subjects will receive placebo. Placebo recipients are included for blinding purposes and safety analyses, and will provide control specimens for immunologic assays. Group 4 was included to serve as a tolerability and immunogenicity control arm.

The sample size for this study will provide a preliminary safety and immunogenicity assessment. While mild to moderate vaccine reactions (local and systemic responses) are expected, AEs that preclude further study vaccine administration or more serious events that would limit product development are not anticipated. With 15 subjects receiving active vaccines per group, the observation of 0 such reactions would be associated with a 97.5% 1-sided confidence upper limit that the true rate is <22%.

The table below shows the probabilities of observing ≥1 AE at given true AE rates.

Probability of Observing ≥1 AE Given a True AE Incidence

	Probability of Observing ≥1 AE (%)		
**True AE Incidence**	**n_1=15**	**n_2=30**	**N=45**
**1%**	14	26	36
**2.5%**	32	53	68
**5%**	54	79	90
**10%**	79	96	99
**20%**	96	100	100

AE = adverse event.
For Ad26.Filo: n_1: number of subjects receiving Ad26.Filo as prime; n_2: number of subjects receiving Ad26.Filo as boost; N: number of subjects receiving Ad26.Filo as either prime or boost.
For MVA-BN-Filo: n_1: number of subjects receiving MVA-BN-Filo as boost; n_2: number of subjects receiving MVA-BN-Filo as prime; N: number of subjects receiving MVA-BN-Filo as either prime or boost.

TIME AND EVENTS SCHEDULE

GROUPS 1, 2, AND 4

	Screening	Study Period[a]													
		Vacc.	Post-vaccination				Vacc.	Post-vaccination							
		Day 1	Day 4	Day 8	Day 15	Day 29	Day 57	Day 60	Day 64	Day 71	Day 78	Day 92	Day 180	Day 240	Day 360
**Visit N**		1	2	3	4	5	6	7	8	9	10	11	12	13	14
**Study Procedures**															
Screening/Administrative[b]															
Informed consent[c]	X														
Inclusion/exclusion criteria[d]	X	X													
Medical history	X														
Review of prestudy therapies	X														
Serology (HIV-1/2, hepatitis B/C)	X														
Serum pregnancy test[e]	X														
Follicle-stimulating hormone (FSH)[f]	X														
Urine drug screen	X														
Study Vaccine Administration															
Randomization		X													
Administer study vaccine/placebo		X[g]					X[h]								
Safety Evaluations[i]															
Urine pregnancy test		X[j]					X[j]								
Physical examination[k]	X	X	X	X	X	X	X	X	X	X	X	X	X	X	X
Vital signs (heart rate, blood pressure, oral body temperature)	X	X[l]					X[l]								
12-lead ECG	X[m]														
Distribution of subjects' diaries (solicited AEs)[n]		X					X								
Collection of subjects' diaries and review by staff			X[o]	X				X[o]	X						
Solicited AEs		X	X	X			X	X	X						
Unsolicited AEs	Continuous until 28 days after the boost vaccination														
SAEs and IREs	X	X	X	X	X	X	X	X	X	X	X	X	X	X	X
Concomitant medications		X	X	X	X	X	X	X	X	X	X	X[p]	X[p]	X[p]	X[p]
Clinical Laboratory Assessments															
Hematology, chemistry	X[q,r]	X[j]		X			X[j]		X						

	Screening	Study Period[a]													
		Vacc.	Post-vaccination				Vacc.	Post-vaccination							
Urinalysis	X[q,r]	X[j]		X			X[j]		X						
Immunogenicity															
Blood samples for humoral immunity		X[j]			X	X	X[j]		X	X	X	X	X	X	X
Blood samples for cellular immunity		X[j]	X	X	X	X	X[j]	X	X	X	X	X	X	X	X
Approximate blood draws															
Daily blood draws (mL)	30	110	10	20	50	60	100	10	70	70	130	50	60	60	60
Cumulative blood draws (mL)	30	140	150	170	220	280	380	390	460	530	660	710	770	830	890

AE = adverse event; ECG = electrocardiogram; FSH = follicle-stimulating hormone; HIV-1/2 = human immunodeficiency virus types 1/2; Inf U = infectious units; IRE: Immediate Reportable Event; SAE = serious adverse event; Vacc. = vaccination day; vp = viral particles.

Footnotes:

a. Refer to Section 9.1.2 for visit windows.

b. Screening may be split into multiple days or visits. Retesting of values (eg, safety laboratory) that lead to exclusion is allowed once using an unscheduled visit during the screening period.

c. Must be signed before first study-related activity.

d. Minimum criteria for the availability of documentation supporting the eligibility criteria are described in Section 17.4. Check clinical status again before first dose of study vaccine.

e. For women of childbearing potential.

f. An FSH test is required for women of any age with amenorrhea for >6 months but <12 months. A woman will then be considered post-menopausal if the measurement of FSH is >40 IU/L or mIU/mL.

g. Ad26.Filo $9x10^{10}$ vp (Group 1), or MVA-BN-Filo at $5x10^{8}$ Inf U (Group 2), or Ad26.ZEBOV $5x10^{10}$ vp (Group 4), or placebo.

h. Heterologous boost with MVA-BN-Filo at $5x10^{8}$ Inf U (Group 1), or Ad26.Filo $9x10^{10}$ vp (Group 2), or MVA-BN-Filo $1x10^{8}$ Inf U (Group 4), or placebo.

i. To be performed in the following order: vital signs, ECG, and blood draw.

j. Prior to vaccine administration.

k. An abbreviated physical examination, including height and body weight but excluding genito-urinary examination, will be carried out at screening. At all post-vaccination visits, an abbreviated, symptom-directed physical examination will be performed as indicated by the investigator based on any clinically relevant issues, clinically relevant symptoms, and medical history. The symptom-directed physical examination may be repeated if deemed necessary by the investigator.

l. Before vaccine administration, and 30 (±5) and 60 (±5) minutes after vaccine administration.

m. A single, 12-lead ECG (supine) after at least 5 minutes rest will be performed at screening. ECGs may be repeated at other time points during the study if clinically indicated based on signs and symptoms.

n. Subjects will use the subject diary to document symptoms of unsolicited and solicited local and systemic adverse events in the evening after each vaccination and then daily for the next 7 days.

o. The investigator or the designee will review subject's diary for local and systemic tolerability, and the investigator will discuss diary entry with the subject and clarify possible misinterpretations on how to fill in the diary. If observations of solicited AEs are made during a visit, the investigator or the designee will ensure that they are reflected in the diary.

p. Only in conjunction with SAEs.

q. Samples at screening may be collected under fed or fasting conditions.

r. Laboratory screening assessments must be performed within 28 days before Day 1.

GROUP 3

Note: Subjects participating at the third vaccination should follow this Time and Event schedule until Day 50 and should then switch to the Time and Events Schedule for GROUP 3: Subjects Participating in Third Vaccination.

	Screening	Study Period[a]												
		Vacc.	Post-vaccination		Vacc.	Post-vaccination								
		Day 1	Day 4	Day 8	Day 15	Day 18	Day 22	Day 29	Day 36	Day 50	Day 92[s]	Day 180[s]	Day 240[s]	Day 360[s]
**Visit N**		1	2	3	4	5	6	7	8	9	10	11	12	13
**Study Procedures**														
Screening/Administrative[b]														
Informed consent[c]	X													
Inclusion/exclusion criteria[d]	X	X												
Medical history	X													
Review of prestudy therapies	X													
Serology (HIV-1/2, hepatitis B/C)	X													
Serum pregnancy test[e]	X													
FSH[f]	X													
Urine drug screen	X													
Study Vaccine Administration														
Randomization		X												
Administer study vaccine/placebo		X[g]			X[h]									
Safety Evaluations[i]														
Urine pregnancy test		X[j]			X[j]									
Physical examination[k]	X	X	X	X	X	X	X	X	X	X	X	X	X	X
Vital signs (heart rate, blood pressure, oral body temperature)	X	X[l]			X[l]									
12-lead ECG	X[m]													
Distribution of subjects' diaries (solicited AEs)[n]		X			X									
Collection of subjects' diaries and review by staff			X[o]	X		X[o]	X							
Solicited AEs		X	X	X	X	X	X							
Unsolicited AEs	Continuous until 28 days after the boost vaccination													
SAEs and IREs	X	X	X	X	X	X	X	X	X	X	X	X	X	X
Concomitant medications		X	X	X	X	X	X	X	X	X[p]	X[p]	X[p]	X[p]	X[p]
Clinical Laboratory Assessments														
Hematology, chemistry	X[q,r]	X[j]		X	X[j]		X							

Urinalysis	X[q,r]	X[j]		X	X[j]		X							
Immunogenicity														
Blood samples for humoral immunity		X[j]			X[j]		X	X	X	X	X	X	X	X
Blood samples for cellular immunity		X[j]	X	X	X[j]	X	X	X	X	X	X	X	X	X
Approximate blood draws														
Daily blood draws (mL)	30	100	10	20	80	10	60	50	100	60	70	60	60	60
Cumulative blood draws (mL)	30	130	140	160	240	250	310	360	460	520	590	650	710	770

AE = adverse event; ECG = electrocardiogram; FSH = follicle-stimulating hormone; HIV = human immunodeficiency virus types 1/2; ICF = informed consent; Inf U = infectious units; IRE: Immediate Reportable Event; SAE = serious adverse event; Vacc. = vaccination day; vp = viral particles.

Footnotes:

a. Refer to Section 9.1.2 for visit windows.
b. Screening may be split into multiple days or visits. Retesting of values (eg, safety laboratory) that lead to exclusion is allowed once using an unscheduled visit during the screening period.
c. Must be signed before first study-related activity.
d. Minimum criteria for the availability of documentation supporting the eligibility criteria are described in Section 17.4. Check clinical status again before first dose of study vaccine.
e. For women of childbearing potential.
f. An FSH test is required for women of any age with amenorrhea for >6 months but <12 months. A woman will then be considered post-menopausal if the measurement of FSH is >40 IU/L or mIU/mL.
g. MVA-BN-Filo at $5x10^8$ Inf U or placebo.
h. Heterologous boost with Ad26.Filo $9x10^{10}$ vp or placebo.
i. To be performed in the following order: vital signs, ECG, and blood draw.
j. Prior to vaccine administration.
k. An abbreviated physical examination, including height and body weight but excluding genito-urinary examination, will be carried out at screening. At all post-vaccination visits, an abbreviated, symptom-directed physical examination will be performed as indicated by the investigator based on any clinically relevant issues, clinically relevant symptoms, and medical history. The symptom-directed physical examination may be repeated if deemed necessary by the investigator.
l. Before vaccine administration, and 30 (±5) and 60 (±5) minutes after vaccine administration.
m. A single, 12-lead ECG (supine) after at least 5 minutes rest will be performed at screening. ECGs may be repeated at other time points during the study if clinically indicated based on signs and symptoms.
n. Subjects will use the subject diary to document symptoms of unsolicited and solicited local and systemic adverse events in the evening after each vaccination and then daily for the next 7 days.
o. The investigator or the designee will review subject's diary for local and systemic tolerability, and the investigator will discuss diary entry with the subject and clarify possible misinterpretations on how to fill in the diary. If observations of solicited AEs are made during a visit, the investigator or the designee will ensure that they are reflected in the diary.
p. Only in conjunction with SAEs.
q. Samples at screening may be collected under fed or fasting conditions.
r. Laboratory screening assessments must be performed within 28 days before Day 1.
s. Subjects who enroll for the third vaccination, see Time and Events Schedule for GROUP 3: Subjects Participating in Third Vaccination.

GROUP 3: Subjects Participating in Third Vaccination

	Study Period[a]					
	Vaccination	Post-vaccination				
	Day 92[b]	Day 99	Day 113	Day 180	Day 240	Day 360
**Visit N**	10	11	12	13	14	15
**Study Procedures**						
Pre-vaccination						
Informed consent	X[c]					
Study Vaccine Administration						
Administer study vaccine/placebo	X[d]					
Safety Evaluations[e]						
Urine pregnancy test	X[f]					
Physical examination[g]	X	X	X	X	X	X
Vital signs (heart rate, blood pressure, oral body temperature)	X[h]					
Distribution of subjects' diaries (solicited AEs)[i]	X					
Collection of subjects' diaries and review by staff		X				
Solicited AEs	X	X				
Unsolicited AEs	Continuous until 28 days after the third vaccination					
SAEs and IREs	X	X	X	X	X	X
Concomitant medications	X	X	X	X[j]	X[j]	X[j]
Clinical Laboratory Assessments						
Hematology, chemistry	X[f]	X				
Urinalysis	X[f]	X				
Immunogenicity						
Blood samples for humoral immunity	X[f]	X	X	X	X	X
Blood samples for cellular immunity	X[f]	X	X	X	X	X
Approximate blood draws						
Daily blood draws (mL)	80	100	60	60	60	60
Cumulative blood draws (mL)	600	700	760	820	880	940

AE: adverse event; IRE: immediate reportable event; SAE: serious adverse event

Footnotes:

a. Refer to Section 9.1.2 for visit windows.
b. Subjects eligible for the third vaccination will follow this schedule.
c. Subjects who are willing to receive a third vaccination must sign the ICF before the first study-related activity.
d. Ad26.Filo $9x10^{10}$ vp or placebo.
e. To be performed in the following order: vital signs and blood draw.
f. Prior to vaccine administration.

g. At all post-vaccination visits, an abbreviated, symptom-directed physical examination will be performed as indicated by the investigator based on any clinically relevant issues, clinically relevant symptoms, and medical history. The symptom-directed physical examination may be repeated if deemed necessary by the investigator.
h. Before vaccine administration and 30 (±5) minutes after vaccine administration.
i. Subjects will use the subject diary to document symptoms of unsolicited and solicited local and systemic adverse events in the evening after each vaccination and then daily for the next 7 days.
j. Only in conjunction with SAEs.

ABBREVIATIONS

Adxx	adenoviral vector xx (vector)
Ad26.Filo	trivalent vaccine comprising 3 monovalent Ad26 vectors expressing the glycoprotein of the Ebola virus Mayinga variant (Ad26.ZEBOV), Marburg virus Angola variant (Ad26.MARVA), and Sudan virus Gulu variant (Ad26.SUDV)
Ad26.MARVA	monovalent Ad26 vector expressing the glycoprotein of the Marburg virus Angola variant (*Lake Victoria marburgvirus*)
Ad26.SUDV	monovalent Ad26 vector expressing the glycoprotein of the Sudan virus Gulu variant (*Sudan ebolavirus*)
Ad26.ZEBOV	monovalent Ad26 vector expressing the glycoprotein of the Ebola virus Mayinga variant (*Zaire ebolavirus*)
Ad35.Filo	trivalent vaccine comprising 3 Ad35 vectors expressing the glycoprotein of the Ebola virus Mayinga variant (Ad35.ZEBOV), Marburg virus Angola variant (Ad35.MARVA), and Sudan virus Gulu variant (Ad35.SUDV)
Ad35.ZEBOV	monovalent Ad35 vector expressing the glycoprotein of the Ebola virus Mayinga variant (*Zaire ebolavirus*)
AE	adverse event
ALT	alanine aminotransferase
AST	aspartate aminotransferase
β-hCG	β-human chorionic gonadotropin
BMI	body mass index
CD	cluster of differentiation
CFR	Code of Federal Regulations
CICP	Countermeasures Injury Compensation Program
CRF	Case Report Form
DMID	Division of Microbiology and Infectious Diseases
DNA	deoxyribonucleic acid
EBOV	Ebola virus
ECG	electrocardiogram
eDC	electronic data capture
ELISA	enzyme-linked immunosorbent assay
FA	Full Analysis
FANG	Filovirus Animal Nonclinical Group
FDA	Food and Drug Administration
FSH	follicle-stimulating hormone
GCP	Good Clinical Practice
GLP	Good Laboratory Practice
GMC	geometric mean concentration
GP	glycoprotein
HIV-1/2	human immunodeficiency virus type 1/type 2
ICF	Informed Consent Form
ICH	International Council for Harmonisation
IEC	Independent Ethics Committee
IM	intramuscular
Inf U	infectious units
IR	Immunogenicity Response (analysis set)
IRB	Institutional Review Board
IRE	Immediate Reportable Event
MARV	Marburg virus
MVA	Modified Vaccinia Ankara
MVA-BN-Filo	Modified Vaccinia Ankara - Bavarian Nordic vector expressing the glycoproteins of Ebola virus, Marburg virus Musoke variant, Sudan virus, and the nucleoprotein of Taï Forest virus
NHP	non-human primate
NIAID	National Institute of Allergy and Infectious Diseases
NIH	National Institutes of Health
NP	nucleoprotein
PQC	product quality complaint

PREP	Public Readiness and Emergency Preparedness
RBC	red blood cell
RNA	ribonucleic acid
SAE	serious adverse event
SMC	Safety Monitoring Committee
SUDV	Sudan virus
SUSAR	serious and unexpected suspected adverse reaction
TAFV	Taï Forest virus
$TCID_{50}$	50% tissue culture infective dose
Tris	Tris hydroxymethyl-aminomethane
ULN	upper limit of normal
vp	viral particle(s)
WBC	white blood cell
ZEBOV	Ebola virus Zaire

DEFINITION OF TERMS

Prime vaccination visit	Day 1 for all groups
- 3 days post-prime	- Day 4 for all groups
- 7 days post-prime	- Day 8 for all groups
- 14 days post-prime	- Day 15 (Groups 1, 2, and 4)
- 28 days post-prime	- Day 29 (Groups 1, 2, and 4)
Boost vaccination visit	Day 15 for Group 3; Day 57 for Groups 1, 2, and 4
- 3 days post-boost	- Day 18 for Group 3; Day 60 for Groups 1, 2, and 4
- 7 days post-boost	- Day 22 for Group 3; Day 64 for Groups 1, 2, and 4
- 14 days post-boost	- Day 29 for Group 3; Day 71 for Groups 1, 2, and 4
- 21 days post-boost	- Day 36 for Group 3; Day 78 for Groups 1, 2, and 4
- 35 days post-boost	- Day 50 for Group 3; Day 92 for Groups 1, 2, and 4
- 77 days post-boost	- Day 92 for Group 3 (subjects not participating in the third vaccination)
- 123 days post-boost	- Day 180 for Groups 1, 2, and 4
- 165 days post-boost	- Day 180 for Group 3 (subjects not participating in the third vaccination)
- 183 days post-boost	- Day 240 for Groups 1, 2, and 4
- 225 days post-boost	- Day 240 for Group 3 (subjects not participating in the third vaccination)
- 303 days post-boost	- Day 360 for Groups 1, 2, and 4
- 345 days post-boost	- Day 360 for Group 3 (subjects not participating in the third vaccination)
Third vaccination visit	Day 92 for a subset of subjects in Group 3 (subjects participating in the third vaccination)
- 7 days post third vaccination	- Day 99
- 21 days post third vaccination	- Day 113
- 88 days post third vaccination	- Day 180
- 148 days post third vaccination	- Day 240
- 268 days post third vaccination	- Day 360
Active vaccine	Ad26.Filo, MVA-BN-Filo, or Ad26.ZEBOV
Study vaccine	Ad26.Filo, MVA-BN-Filo, Ad26.ZEBOV, or placebo
Placebo vaccine	0.9% normal saline
Independent study vaccine monitor	An unblinded study vaccine monitor assigned to the study who is responsible for the unblinded interface between the sponsor and the investigational site pharmacy
Blinded study vaccine administrator	A blinded trained study nurse, medical doctor, or otherwise qualified healthcare provider

1. INTRODUCTION

Janssen Vaccines & Prevention B.V. (formerly known as Crucell Holland B.V.) is investigating the potential of a prophylactic filovirus vaccine regimen comprising the following 2 candidate filovirus vaccines:

Ad26.Filo is a multivalent vaccine comprising 3 adenovirus serotype 26 (Ad26) vectors expressing the glycoprotein (GP) of the Ebola virus (EBOV) Mayinga variant (Ad26.ZEBOV), the GP of the Marburg virus (MARV) Angola variant (Ad26.MARVA), and the GP of the Sudan virus (SUDV) Gulu variant (Ad26.SUDV). All 3 are produced in the human PER.C6® cell line.

MVA-mBN226B (hereafter referred to as MVA-BN-Filo) is a multivalent vaccine using the Modified Vaccinia Ankara - Bavarian Nordic (MVA-BN®) vector expressing the GP of the EBOV Mayinga variant, the GP of the MARV Musoke variant, the GP of the SUDV Gulu variant, and the nucleoprotein (NP) of the Taï Forest virus (TAFV, formerly known as *Côte d'Ivoire ebolavirus*). It is produced in chicken embryo fibroblast cells. The EBOV GP and SUDV GP expressed by Ad26.Filo have 100% similarity with the GPs expressed by the MVA-BN-Filo vaccine. The MARV Angola GP expressed by Ad26.Filo has 93% similarity with the MARV Musoke GP expressed by MVA-BN-Filo.

For the most comprehensive nonclinical and clinical information regarding Ad26.Filo and MVA-BN-Filo, refer to the latest version of the Investigator's Brochure and Addenda for Ad26.Filo and MVA-BN-Filo.[14,16]

The term "sponsor" used throughout this document refers to the entities listed in the Contact Information page(s), which will be provided as a separate document.

The Division of Microbiology and Infectious Diseases (DMID) of the National Institute of Allergy and Infectious Diseases (NIAID) of the United States National Institutes of Health (NIH) is funding this study under contract HHSN272200800056C.

1.1. Background

Filoviruses can induce severe hemorrhagic fever in humans and non-human primates (NHPs), for which there is currently no licensed vaccine, treatment, or cure. These viruses are highly prioritized by the United States Government, who has defined them as "Category A" agents because of the high mortality rate of infected individuals.

In the past 40 years, a total of 24 *Ebolavirus* and 11 *Marburgvirus* natural outbreaks involving fatal cases have occurred in or originated in Africa,[3,26] with approximately 50% of these outbreaks occurring in the last decade. Case fatality rates in Ebola virus disease range from 25% to 90%, and case fatality rates in Marburg hemorrhagic fever range from 24% to 88%.[25,26] In 2014, an *Ebolavirus* outbreak, initiating in Guinea in December 2013, and spreading primarily to Sierra Leone and Liberia, resulted in more cases (more than 27,000) and deaths (more than 11,000) than all previous outbreaks combined. This *Ebolavirus* outbreak is by far the largest and most complex ever recorded, highlighting the epidemic potential of filoviruses and the need for a prophylactic vaccine.

Filoviruses are named for their long, filamentous shape. Within this filamentous virus, a single 19-kilobase negative-sense ribonucleic acid (RNA) genome encodes 7 proteins: the GP, the polymerase, the NP, the secondary matrix protein, the transcriptional activator, the polymerase cofactor, and the matrix protein. The virion surface is covered by homotrimers of the viral GP, which is believed to be the sole host attachment factor for filoviruses. Following cell entry, the viruses replicate their genomes and viral proteins in the cytoplasm using an RNA-dependent RNA polymerase, which is carried into the cell together with the virus.[10]

Janssen Vaccines & Prevention B.V., the Netherlands, in collaboration with Bavarian Nordic A/S, Denmark, and in partnership with the NIAID of the NIH, are currently developing a monovalent vaccine regimen (VAC52150), for prophylaxis against the EBOV species, which is responsible for the latest *Ebolavirus* outbreak in West Africa. This regimen consists of the monovalent vector Ad26.ZEBOV, which targets the EBOV Mayinga variant GP, in conjunction with the multivalent vaccine MVA-BN-Filo. In addition, the development of the monovalent vector Ad26.ZEBOV is part of a larger ongoing development program for a multivalent vaccine against multiple filoviruses that cause disease in humans, including EBOV, MARV, and SUDV. Three Ad26 vector-based monovalent vaccines, ie, Ad26.ZEBOV, Ad26.MARVA, and Ad26.SUDV, and the multivalent MVA-BN-Filo vaccine expressing the GPs of EBOV, MARV, and SUDV plus the NP of TAFV, are planned to be administered in a heterologous prime-boost regimen.

In this first-in-human study, the multivalent vaccines Ad26.Filo and MVA-BN-Filo will be evaluated as heterologous prime-boost regimens, in which each vaccine is used to prime a filovirus-specific immune response followed by a boost immunization with the other vaccine. MVA-BN-Filo prime will be boosted by Ad26.Filo 14 or 56 days later; Ad26.Filo prime will be boosted by MVA-BN-Filo 56 days later. Additionally, in a subset of subjects, a third vaccination will be administered at Day 92 to evaluate whether an anamnestic response could be observed after this third vaccination.

1.1.1. Nonclinical Studies

1.1.1.1. Immunogenicity and Efficacy

Immunogenicity and efficacy of the heterologous regimen of Ad26.Filo (a trivalent vaccine comprising Ad26.ZEBOV, Ad26.MARVA, and Ad26.SUDV) and MVA-BN-Filo (a multivalent vaccine comprising the EBOV, MARV, and SUDV GPs plus the TAFV NP) have been assessed for 1, 29 and 1, 57 day prime-boost intervals in NHPs.

In a proof-of-concept study, heterologous regimens of Ad26.Filo at a dose of $1.2x10^{11}$ vp and MVA-BN-Filo at a dose of $5x10^{8}$ 50% tissue culture infective dose ($TCID_{50}$) were evaluated in an NHP model (Cynomolgus macaques, *Macaca fascicularis*). Heterologous 1, 57 day prime-boost regimens were assessed in a small number of animals (2 per regimen). Complete survival after intramuscular (IM) challenge with EBOV Kikwit 1995 challenge (Filovirus Animal Nonclinical Group [FANG]-recommended challenge material) was observed for regimens consisting of Ad26.Filo/MVA-BN-Filo, MVA-BN-Filo/Ad26.Filo, and Ad26.Filo/Ad35.Filo (trivalent vaccine comprising the monovalent adenovirus serotype 35

[Ad35] vectors: Ad35.ZEBOV, Ad35.MARVA, Ad35.SUDV) prime-boost immunizations. Additionally, 100% survival (2/2 NHPs) following EBOV Kikwit 1995 challenge was observed with a 1, 29 day monovalent regimen consisting of Ad26.ZEBOV/Ad35.ZEBOV (monovalent Ad35 vector expressing the EBOV GP) prime-boost immunizations. Very limited signs of filovirus-induced disease were observed after challenge in all protected animals. A specific humoral immune response against all 3 viruses was observed after prime with Ad26.Filo or MVA-BN-Filo. Post-prime immune responses tended to be higher after Ad26.Filo priming than after MVA-BN-Filo priming. A filovirus-specific cellular immune response was elicited in 5 of 10 animals at Week 4 post-prime immunization. A strong boost response was observed in all animals receiving the boost immunization 8 weeks (Day 57) after the prime immunization, independent of the vaccination regimen.

In a second study performed to confirm the preclinical proof-of-concept data, 1, 29 and 1, 57 day prime-boost intervals were assessed in a larger number of NHPs, with either Ad26.Filo ($1.2x10^{11}$ vp) or MVA-BN-Filo ($5x10^8$ $TCID_{50}$) given as prime followed by the other vaccine as boost. For the 1, 57 day prime-boost interval, both prime-boost regimens tested resulted in 75% survival (3/4 NHPs) following EBOV Kikwit challenge, as did the 1, 29 day prime-boost interval with Ad26.Filo as prime; however, the 1, 29 day prime-boost interval with MVA-BN-Filo as prime resulted in only 25% survival. Very limited signs of filovirus-induced disease were observed after challenge in surviving animals.

NHP studies with the monovalent Ad26.ZEBOV ($5x10^{10}$ vp) candidate vaccine combined with MVA-BN-Filo ($1x10^8$ $TCID_{50}$ or $5x10^8$ $TCID_{50}$), again using either vector as prime or boost in a 1, 57 day schedule, are supportive of the findings with Ad26.Filo and MVA-BN-Filo: 100% survival (8/8 NHPs) was recorded in EBOV Kikwit challenge studies with Ad26.ZEBOV and MVA-BN-Filo.[15] Slightly lower survival rates were achieved with shorter vaccination schedules of 1, 29 days (40% [2/5 NHPs] and 100% [2/2 NHPs] in 2 studies with Ad26.ZEBOV as prime and 50% [4/8 NHPs] with MVA-BN-Filo as prime) or 1, 43 days (80% [4/5 NHPs] with Ad26.ZEBOV as prime), with a trend favoring regimens using Ad26.ZEBOV as prime.[15]

Ad26.Filo/MVA-BN-Filo, MVA-BN-Filo/Ad26.Filo, and Ad26.Filo/Ad35.Filo prime-boost regimens were also assessed in a proof-of-concept study that evaluated survival following MARV Angola challenge (FANG-recommended challenge material). Using 1, 57 day prime-boost intervals for Ad26.Filo ($1.2x10^{11}$ vp) and MVA-BN-Filo ($5x10^8$ $TCID_{50}$) regimens or Ad26.Filo ($1.2x10^{11}$ vp) and Ad35.Filo ($1.2x10^{11}$ vp) regimens, both prime-boost regimens resulted in 100% survival in NHPs (5/5 and 4/4 animals, respectively) after IM challenge with MARV Angola. Ad26.Filo/MVA-BN-Filo 1, 57 day prime-boost regimens appeared to be superior to MVA-BN-Filo/Ad26.Filo prime-boost regimens, as only 3/5 (60%) animals survived in the MVA-BN-Filo/Ad26.Filo group. Very limited signs of filovirus-induced disease were observed after challenge in the Ad26.Filo/Ad35.Filo group, whereas 1 animal in the Ad26.Filo/MVA-BN-Filo group had substantial morbidity between Days 8 and 13 after challenge, which had resolved by Day 14 after challenge.

A proof-of-concept study to evaluate survival following SUDV Gulu challenge (FANG-recommended challenge material) in NHPs, using a study design analogous to the

MARV proof-of-concept study described above, has recently been completed, with comparable results to the MARV study. Using a 1, 57 day prime-boost schedule, both Ad26.Filo/MVA-BN-Filo and Ad26.Filo/Ad35.Filo prime-boost regimens resulted in 100% survival in NHPs (5/5 animals in both groups) after IM challenge with SUDV Gulu, while the MVA-BN-Filo/Ad26.Filo prime-boost regimen resulted in 60% survival (3/5 animals). Surviving animals had limited signs of filovirus-induced disease after challenge.

1.1.1.2. Toxicology

There is a substantial amount of animal and human data available supporting the safety of Ad26- and MVA-based vaccines. The nonclinical safety profiles of either MVA-BN-Filo alone or heterologous prime-boost regimens of MVA-BN-Filo and the monovalent Ad26.ZEBOV vaccine were evaluated in separate Good Laboratory Practice (GLP) repeated-dose toxicology studies in rabbits. Overall, the available nonclinical studies indicated that heterologous prime-boost regimens of Ad26- and MVA-based vaccines were well tolerated, not showing any adverse effects of the vaccines when given at full human doses to the animals.

Because no specific toxicity data were available for Ad26.Filo, and because the proposed dose of Ad26.Filo ($9x10^{10}$ vp) is higher than the dose previously tested in most studies with Ad26.ZEBOV ($5x10^{10}$ vp), a GLP repeated-dose toxicology study to evaluate the safety profile of heterologous prime-boost regimens of Ad26.Filo and MVA-BN-Filo was conducted in rabbits. Prime-boost combinations of Ad26.Filo and MVA-BN-Filo (or Ad26.Filo and Ad35.ZEBOV) were well tolerated when administered to rabbits IM once every 2 weeks for up to 4 weeks (ie, total of 3 injections).[14] The observed transient changes in body weight, food consumption, body temperature, and clinical pathology parameters (including C-reactive protein) were considered to reflect a normal, non-adverse response to the vaccine administration. All vaccination regimens elicited EBOV-, MARV-, and SUDV-specific immune responses. Non-adverse organ weight/microscopic changes related to an immunogenic response were observed in iliac lymph nodes and spleen at the end of treatment. In addition, local inflammatory reactions at the administration sites were observed. The microscopic findings showed ongoing recovery after a 3-week treatment-free period.

Data on reproductive toxicology are available for the monovalent Ad26.ZEBOV vaccine and MVA-BN-Filo vaccine. A GLP-compliant, combined embryo-fetal and pre- and postnatal development study with heterologous prime-boost regimens of Ad26.ZEBOV ($1x10^{11}$ vp) and MVA-BN-Filo ($3.61x10^{8}$ infectious units [Inf U]; note that these units can be used interchangeably with $TCID_{50}$) was conducted in New Zealand White rabbits to determine maternal and developmental toxicity following maternal exposure to the vaccines. This study was performed with IM injections 8 days prior to gestation (prime) and on gestation day 6 (boost). The Ad26.ZEBOV and MVA-BN-Filo prime-boost regimens did not induce maternal or developmental toxicity following maternal exposure during the premating and gestation period. All vaccine regimens elicited detectable EBOV GP-specific maternal antibody titers that were transferred to the fetuses. An inflammatory/immune response observed following IM immunization with Ad26.ZEBOV or MVA-BN-Filo was associated with increases in monocyte and lymphocyte counts, and increases in fibrinogen, globulin, and C-reactive protein

concentrations. The observed changes reflect a typical response commonly seen with vaccine administration and were not considered adverse.

1.1.1.3. Biodistribution

The proposed vaccination regimen makes use of 2 different vectors: Ad26 and MVA-BN. Although the biodistribution of Ad26.Filo and MVA-BN-Filo have not specifically been studied, the biodistribution potential of the Ad26 vector (in combination with another insert) and the MVA-BN vector alone have been evaluated in rabbits. The Ad26 vector had a limited biodistribution after IM injection in rabbits and showed clearance from the muscle at the site of injection, draining (iliac) lymph nodes, and spleen within 3 months for most of the inoculated animals. Compared with Ad26, MVA-BN was more rapidly cleared (within 48 hours post-vaccination). As biodistribution is dependent on the viral vector platform and not on the insert, a further specific study with Ad26.Filo or MVA-BN-Filo is considered redundant.

1.1.2. Clinical Studies

There is no previous clinical experience with Ad26.Filo and limited clinical experience with heterologous prime-boost regimens using MVA-BN-Filo and Ad26.ZEBOV. Available safety and immunogenicity data generated with heterologous prime-boost regimens of Ad26.ZEBOV and MVA-BN-Filo in several ongoing clinical studies are discussed below.

1.1.2.1. Safety Profile of Ad26-based Vaccines

As of 10 December 2015 (ie, the cut-off date of the Ad26.Filo Investigator's Brochure[14]), the safety, tolerability, and immunogenicity of the Ad26.ZEBOV vaccine were being assessed in ongoing Phase 1 (VAC52150EBL1001, VAC52150EBL1002, VAC52150EBL1003, VAC52150EBL1004, and VAC52150EBL1005), Phase 2 (VAC52150EBL2001 and VAC52150EBL2002), and Phase 3 clinical studies (VAC52150EBL3001, VAC52150EBL3002, and VAC52150EBL3003). In these ongoing studies, the monovalent Ad26.ZEBOV vaccine and the multivalent MVA-BN-Filo vaccine are combined in homologous or heterologous prime-boost regimens in which each vector is used to prime a filovirus-specific immune response followed by a boost immunization with the same or the other vector 2 to 12 weeks later.[15]

As of 10 December 2015, approximately 1,000 healthy adult subjects in these ongoing studies had received Ad26.ZEBOV at dose levels ranging from 0.8×10^{10} vp to 1×10^{11} vp. Safety and immunogenicity results from the primary analysis, performed when all subjects had completed their 21-day post-boost visit or withdrew earlier, were available only for Studies VAC52150EBL1001[6] and VAC52150EBL1002[8].

1.1.2.1.1. Pooled Safety Data From Studies VAC52150EBL1001 and VAC52150EBL1002

The pooled safety data from Studies VAC52150EBL1001 and VAC52150EBL1002, based on the primary analyses of Groups 1 to 5 of study VAC52150EBL1001 and Groups 1 to 8 of study VAC52150EBL1002, are reported in this protocol. These primary analyses were performed when all subjects in the above-mentioned groups had completed their 21-day post-boost visit or discontinued earlier. It should be noted that, at the database cut-off date for primary analysis of

Groups 1 to 8 of study VAC52150EBL1002 (2 July 2015), the third part of this study (Groups 9 and 10), as well as the third open-label vaccination (Day 360), had not yet been included in the study design (added per protocol amendment 5; approved 26 October 2015).[4]

Hence, the 2 combined studies provided the following study population for prime and boost vaccination:

Prime Vaccination (Post-prime)[a]	Boost Vaccination (Post-boost)[a]
32 subjects primed with placebo	32 subjects boosted with placebo
84 subjects primed with MVA-BN-Filo $1x10^8$ Inf U	65 subjects boosted with MVA-BN-Filo $1x10^8$ Inf U
83 subjects primed with Ad26.ZEBOV $5x10^{10}$ vp	28 subjects boosted with MVA-BN-Filo $4.4x10^8$ Inf U
15 subjects primed with Ad26.ZEBOV $1x10^{11}$ vp	84 subjects boosted with Ad26.ZEBOV $5x10^{10}$ vp

Inf U = infectious units; vp = viral particles
a See Ad26.Filo Investigator's Brochure for detailed description of the population.[14]

Of note, as all subjects primed with Ad26.ZEBOV $1x10^{11}$ vp belong to a single group within Study VAC52150EBL1002, and the number of subjects is limited, the data from this group should be interpreted with caution.

Solicited Adverse Events

Summaries of all solicited adverse events (AEs) reported after prime vaccination and after boost vaccination are provided in Appendix 3 and Appendix 4, respectively, of the Ad26.Filo Investigator's Brochure.[14] The events are tabulated by worst severity grade.

Solicited local AEs were more frequently reported after prime or boost vaccination with MVA-BN-Filo and Ad26.ZEBOV than with placebo.[14] In addition, there was a trend towards higher frequencies of solicited local AEs after prime (74.7%) or boost (84.5%) vaccination with Ad26.ZEBOV $5x10^{10}$ vp than after prime (57.1% with $1x10^8$ Inf U) or boost (58.5% and 64.3% with $1x10^8$ Inf U and $4.4x10^8$ Inf U, respectively) vaccination with MVA-BN-Filo. From the limited data available, it appears that increasing the Ad26.ZEBOV dose for prime vaccination to $1x10^{11}$ vp did not increase the reported frequency of solicited local AEs (46.7%) compared with the standard dose of $5x10^{10}$ vp (74.7%). The most frequently reported solicited local AE was injection site pain, both after prime and after boost vaccination.

Grade 3 solicited local AEs were reported by 1 subject after prime with Ad26.ZEBOV $5x10^{10}$ vp (injection site pain) and by 2 subjects after boost with Ad26.ZEBOV $5x10^{10}$ vp (injection site erythema, in 1 case associated with injection site swelling). All solicited local AEs were transient in nature and resolved without sequelae.

Solicited systemic AEs were more frequently reported after prime or boost vaccination with Ad26.ZEBOV $5x10^{10}$ vp (post-prime and post-boost: 77.1% and 64.3%) than with MVA-BN-Filo $1x10^8$ Inf U (post-prime and post-boost: 51.2% and 36.9%) or placebo (post-prime and post-boost: 59.4% and 31.3%). From the limited data available, it appears that increasing the Ad26.ZEBOV dose for prime vaccination to $1x10^{11}$ vp did not increase the reported frequency of solicited systemic AEs (66.7%) compared with the dose of $5x10^{10}$ vp (77.1%). Similarly, increasing the MVA-BN-Filo dose for boost vaccination to $4.4x10^8$ Inf U did

not result in a higher frequency of solicited systemic AEs (28.6%) compared with the $1x10^8$ Inf U dose (36.9%). Overall, the most frequently reported solicited systemic AEs were fatigue, headache, myalgia, and chills, both after prime and after boost vaccination.

In the Ad26.ZEBOV $5x10^{10}$ vp group, Grade 3 solicited systemic AEs were reported more frequently, both after prime (10.8% [9 subjects]) and after boost (8.3% [7 subjects]), than in the MVA-BN-Filo $1x10^8$ Inf U group (only in 1 subject [1.2%] after prime). In the Ad26.ZEBOV $5x10^{10}$ vp group, this difference was mostly due to chills, fatigue, and headache (reported by 6, 5, and 4 subjects, respectively) and to a minor extent to myalgia and nausea (each reported by 2 subjects) and pyrexia and arthralgia (each reported by 1 subject) after prime vaccination. In the MVA-BN-Filo $1x10^8$ Inf U group, the subject experiencing Grade 3 solicited systemic AEs complained of myalgia, nausea, and vomiting. The same pattern of solicited systemic AEs was reported after boost with Ad26.ZEBOV $5x10^{10}$ vp, with the following events being reported: chills, headache, and fatigue (reported by 5, 4, and 2 subjects, respectively) and nausea, myalgia and pyrexia (each reported by 1 subject). Grade 3 solicited systemic AEs were not reported in the limited number of subjects who received Ad26.ZEBOV $1x10^{11}$ vp as prime. After boost with MVA-BN-Filo $4.4x10^8$ Inf U, 1 subject reported Grade 3 pyrexia. Grade 3 fatigue was reported by 1 subject after boost with placebo. All solicited systemic AEs were transient in nature and resolved without sequelae.

Unsolicited AEs

Summaries of all unsolicited AEs reported after prime vaccination and after boost vaccination are provided in Appendix 5 and Appendix 6, respectively, of the Ad26.Filo Investigator's Brochure.[14] The events are tabulated by system organ class and preferred term.

The overall frequency of unsolicited AEs was generally comparable between Ad26.ZEBOV $5x10^{10}$ vp (post-prime and post-boost: 75.9% and 51.2%), MVA-BN-Filo $1x10^8$ Inf U (post-prime and post-boost: 72.6% and 56.9%), and placebo (post-prime and post-boost: 65.6% and 68.8%).[14] From the limited data available, it appears that increasing the Ad26.ZEBOV dose for prime vaccination to $1x10^{11}$ vp did not increase the reported frequency of unsolicited AEs (53.3%) compared with the standard dose of $5x10^{10}$ vp (75.9%). There was a trend towards lower frequencies of unsolicited AEs post-boost for the higher MVA-BN-Filo dose of $4.4x10^8$ Inf U (39.3%) compared with the standard dose of MVA-BN-Filo $1x10^8$ Inf U (56.9%).

Ad26.ZEBOV

Post-prime Events

Unsolicited AEs reported with an overall frequency of ≥5% in the Ad26.ZEBOV $5x10^{10}$ vp group were leukopenia (10.8% versus 9.4% in the placebo group), neutropenia (25.3% versus 18.8% in the placebo group), and hypokalemia (12% versus 12.5% in the placebo group). Of these unsolicited AEs, only neutropenia was reported with a frequency at least 1.25 times higher than in the placebo group.

Among the limited number of subjects receiving the Ad26.ZEBOV $1x10^{11}$ vp dose, unsolicited AEs reported in more than 1 subject were diarrhea, increased alanine aminotransferase (ALT), and increased blood pressure (each reported in 2 subjects, ie, 13.3% of subjects receiving this dose).

Post-boost Events

Unsolicited AEs reported with an overall frequency of ≥5% in the Ad26.ZEBOV $5x10^{10}$ vp group were neutropenia (11.9% of subjects versus 6.3% in the placebo group) and hypokalemia (9.5% of subjects versus 9.4% in the placebo group). Of these unsolicited AEs, only neutropenia was reported with a frequency at least 1.25 times higher than in the placebo group.

<u>MVA-BN-Filo</u>

Post-prime Events

Unsolicited AEs reported with a frequency of ≥5% in the MVA-BN-Filo $1x10^8$ Inf U group were neutropenia (13.1% of subjects versus 18.8% in the placebo group), upper respiratory tract infection (7.1% of subjects versus 6.3% in the placebo group), hypokalemia (11.9% of subjects versus 12.5% in the placebo group), and oropharyngeal pain (6% of subjects versus 0% in the placebo group).

Post-boost Events

Unsolicited AEs reported with a frequency of ≥5% in the MVA-BN-Filo $1x10^8$ Inf U group were neutropenia (6.2% of subjects versus 6.3% in the placebo group) and hypokalemia (15.4% of subjects versus 9.4% in the placebo group). Of these unsolicited AEs, only hypokalemia was reported with a frequency at least 1.25 times higher than in the placebo group.

Among the limited number of subjects receiving the MVA-BN-Filo $4.4x10^8$ Inf U dose, unsolicited AEs reported in more than 1 subject were increased blood pressure (10.7% of subjects versus 0 in the placebo group) and proteinuria (7.1% of subjects versus 0 in the placebo group), both being reported with a frequency at least 1.25 times higher than in the placebo group.

Laboratory Abnormalities

Summaries of emerging worst laboratory toxicity grades after prime vaccination and after boost vaccination, expressed as the percentage of the total number of subjects undergoing each lab test, are provided in Appendix 7 and Appendix 8, respectively, of the Ad26.Filo Investigator's Brochure.[14]

No consistent post-prime and post-boost modification of chemistry safety parameters was apparent. Grade 3 hypokalemia was reported across all groups, except after prime with Ad26.ZEBOV $1x10^{11}$ vp and after boost with MVA-BN-Filo $4.4x10^8$ Inf U. Grade 3 hyponatremia was reported post-boost in 1 subject who received Ad26.ZEBOV $5x10^{10}$ vp.

The only consistent hematologic finding was a trend towards decreased neutrophil or segmented neutrophil count after prime vaccination with Ad26.ZEBOV $5x10^{10}$ vp: a total of 10 subjects (12.0%) had either Grade 2 (8 subjects) or Grade 3 (2 subjects) decreased (segmented) neutrophil count in the Ad26.ZEBOV $5x10^{10}$ vp group compared with 5 subjects (6.0%) (4 Grade 2 and 1 Grade 3) in the MVA-BN-Filo $1x10^{8}$ Inf U group. After boost vaccination, 6 subjects (7.1%) had either Grade 2 (5 subjects) or Grade 3 (1 subject) decreased (segmented) neutrophil count in the Ad26.ZEBOV $5x10^{10}$ vp group, compared with only 1 subject (1.5%) with a Grade 2 decrease in the MVA-BN-Filo $1x10^{8}$ Inf U group. In the placebo group, only 1 subject (5.0%) had Grade 2 decreased (segmented) neutrophil count, after either prime or boost. No abnormalities in neutrophil count were reported in the high dose groups (either Ad26.ZEBOV $1x10^{11}$ vp prime or MVA-BN-Filo $4.4x10^{8}$ Inf U boost); however, because of the limited number of subjects in these groups, these findings should be interpreted with caution. As for other hematology parameters, Grade 3 abnormalities were reported in a few cases in all groups either after prime vaccination (1 subject with hemoglobin decrease after Ad26.ZEBOV $5x10^{10}$ vp vaccination and 1 subject with prolonged activated partial thromboplastin time after placebo vaccination) or after boost vaccination (2 and 1 subjects with hemoglobin decrease after MVA-BN-Filo $1x10^{8}$ Inf U and placebo vaccinations, respectively).

Deaths, Serious AEs, and Other Significant AEs

No deaths and no AEs of special interest (any cardiac sign or symptom that developed since the prime vaccination, electrocardiogram [ECG] changes determined to be clinically significant) were reported during the primary analysis period of Studies VAC52150EBL1001 and VAC52150EBL1002.

Two AEs leading to permanent discontinuation of vaccination were reported during the primary analysis period in Study VAC52150EBL1001: 2 subjects in the substudy who experienced Grade 3 neutropenia did not receive the boost vaccination, as they met criteria for contraindications to boost (specified in the protocol), but continued scheduled assessments as planned. No AEs leading to permanent discontinuation were reported in study VAC52150EBL1002.

Two serious AEs (SAEs) were reported in Study VAC52150EBL1001 (main study) during the primary analysis period, both of which were not considered related to the study vaccine by the investigator:

- Left forearm fracture sustained in an ice-skating accident that required hospitalization in a subject primed with MVA-BN-Filo $1x10^{8}$ Inf U.
- Gastritis with hematemesis due to excessive alcohol intake that required an overnight stay in a hospital in a placebo-primed subject.

No SAEs were reported during the primary analysis period in the VAC52150EBL1001 substudy or during the primary analysis period in study VAC52150EBL1002.

Conclusions from Studies VAC52150EBL1001 and VAC52150EBL1002

The combined analysis of the primary analysis safety data from Studies VAC52150EBL1001 and VAC52150EBL1002 suggests that both the Ad26.ZEBOV and the MVA-BN-Filo vaccines are well tolerated, with no clinically relevant safety/tolerability concerns. The majority of the AEs were mild, transient in nature, and resolved without sequelae. The findings appear to be consistent with the safety profiles observed with similar vaccines.[2,12] From the limited data available, it appears that increasing the Ad26.ZEBOV dose for prime vaccination from $5x10^{10}$ vp to $1x10^{11}$ vp or increasing the MVA-BN-Filo dose for boost vaccination from $1x10^8$ Inf U to $4.4x10^8$ Inf U is not associated with a higher frequency of AEs or laboratory abnormalities.

1.1.2.1.2. Relevant Safety Information from Ongoing VAC52150 Studies

One subject in the study VAC52150EBL2001 experienced a serious and very rare condition called “Miller Fisher syndrome”. This condition consists of double vision, pain on moving the eye, and difficulty with balance while walking. Miller Fisher syndrome most commonly occurs following a recent infection. The subject experienced these symptoms about a week after suffering from a common cold and fever. The event happened about a month after boost vaccination with either MVA-BN-Filo or placebo. This subject had to go to the hospital for treatment and has recovered. After an extensive investigation, the event has been considered to be doubtfully related to vaccine and most likely related to the previous common cold.

In the ongoing clinical studies with more than 2,000 participants, there have been a few reports of mild to moderate tingling especially in the hands and feet or a sensation of mild to moderate muscle weakness in subjects vaccinated with Ad26.ZEBOV or placebo. These symptoms have been observed to last no more than 24-48 hours in the majority of cases but can last for several weeks before going away on their own. These types of symptoms have also been reported following administration of other licensed vaccines and following acute viral infections of various types. One serious case of probable peripheral sensory neuropathy of moderate severity has occurred and has been ongoing for several months, interfering with some of the subject's daily activities.

1.1.2.1.3. Safety Data From Other Ad26-based Vaccines.

In addition to completed studies with Ad26.ZEBOV, the safety of other Ad26-based vaccines has been assessed in 3 randomized, placebo-controlled, Phase 1 studies (IPCAVD-001, IPCAVD-003, and IPCAVD-004) with the prototype vaccine Ad26.ENVA.01. This vaccine was administered to 206 healthy HIV-uninfected subjects between the ages of 18 and 50 in the United States and Africa. No deaths or vaccine-related SAEs were reported for any study.[1,13] In the largest study (IPCAVD-004), the proportion of subjects with moderate or severe symptoms was not statistically significantly different between vaccine and placebo groups.[17]

In addition, a completed, Phase 1/2a, double-blind, randomized, placebo-controlled dose escalation study of a malaria vaccine, sponsored by Crucell Holland B.V. (MAL-V-A001), investigated the safety, tolerability, and immunogenicity of 2 dose levels of the Ad35.CS.01/Ad26.CS.01 prime-boost regimens in healthy subjects. The dose escalation phase was followed by an evaluation of efficacy of the higher dose level in an experimental malaria

challenge. In total, 28 subjects received ≥1 dose of the Ad26.CS.01 vaccine, while 6 subjects received placebo. The analysis of AEs did not show any consistent pattern suggestive of an association of Ad35.CS.01 or Ad26.CS.01 with specific AEs.[1]

Additionally, there is 1 ongoing Phase 1/2a study with Ad26.Mos.HIV (Study HIV-V-A004), a trivalent recombinant vaccine encoding Mosaic human immunodeficiency virus type 1 (HIV-1) group-specific antigen (Gag), polymerase (Pol), and envelope (Env) proteins, in 394 healthy HIV-uninfected subjects (Ad26.Mos.HIV/placebo ratio: 7/1). As of 13 October 2015 (ie, the cut-off date of the latest version of the Investigator's Brochure of Ad26.Mos.HIV and Ad26.Mos4.HIV), 1 SAE assessed as related to the study products by the investigator had been reported: severe allergic reaction and chest pain. The reaction was resolved within 1 day of onset. The subject was withdrawn from the study because of noncompliance (history of drug abuse and non-reported psychiatric issues). No deaths had been reported.[13]

1.1.2.2. Safety Profile of MVA-BN-based Vaccines

MVA-BN is a further attenuated version of the MVA virus, which itself is a highly attenuated strain of the poxvirus Chorioallantois Vaccinia Virus Ankara. MVA-BN induces strong cellular activity as well as humoral (antibody) immune responses and has demonstrated an ability to stimulate a response even in individuals with preexisting immunity against vaccinia. One of the advantages of MVA-BN is the virus' inability to replicate in a vaccinated individual. The replication cycle is blocked at a very late stage, which ensures that new viruses are not generated and released. This means that the virus cannot spread in the vaccinated person, and none of the serious side effects normally associated with replicating vaccinia viruses have been seen with MVA-BN.

MVA-BN (IMVAMUNE® outside the European Union, IMVANEX® in the European Union) has received marketing authorization in the European Union for active immunization against smallpox in adults and in Canada for persons 18 years of age and older who have a contraindication to the first or second generation smallpox vaccines including individuals with immune deficiencies and skin disorders.[12] Results from completed and ongoing clinical studies of MVA-BN-based vaccines (at doses of up to $5x10^8$ $TCID_{50}$) in more than 8,100 individuals, including elderly, children, and immunocompromised subjects in whom replicating vaccines are contraindicated, have shown that the platform displays high immunogenicity and a favorable safety profile.[16] Across all clinical studies, no trends for unexpected and/or serious adverse reactions due to the product were detected. MVA-BN and MVA-BN-based recombinant vaccines were shown to be safe in healthy subjects as well as in immunocompromised populations with a contraindication against conventional smallpox vaccines, eg, individuals with HIV infection or diagnosed with atopic dermatitis.

Data from extensive nonclinical studies support the safety profile of the MVA-BN strain.[22,23,24]

1.1.2.3. Viral Shedding

Viral shedding information is available from 6 clinical studies with adenovector-based vaccines against HIV-1 (using Ad26 and Ad35: Ad26.ENVA.01 and Ad35.ENV) and *Mycobacterium*

tuberculosis (using Ad35.TB-S [AERAS-402]). In none of these clinical studies was viral shedding observed. In a clinical study evaluating viral shedding of Ad26.ENVA.01 and Ad35.ENV (Study IPCAVD-004), all cultures from oropharyngeal swabs and urine were negative for adenovirus; in 5 clinical studies evaluating viral shedding of Ad35-TB-S (Studies C-001-402, C-003-402, C-008-402, C-009-402, and C-017-402), no shedding of adenovirus was observed in any of the urine or throat cultures.[15] Viral shedding of Ad26 vector in urine samples and mid-turbinate swabs after single dose vaccination with Ad26.ZEBOV ($5x10^{10}$ vp) is being investigated in study VAC52150EBL2001 (France-specific amendment to global protocol amendment 3)[5]; results are pending.

MVA-BN-Filo is an attenuated recombinant MVA incapable of replication in human cells. In human cells, upon infection, viral genes are expressed, but no infectious progeny virus is produced. Given the block in virus assembly and the very limited host range of the vector, no viral shedding studies were performed.

1.1.2.4. Immunogenicity

Immunogenicity data are available for the heterologous Ad26.ZEBOV and MVA-BN-Filo prime-boost regimens from the primary analyses of Phase 1 Studies VAC52150EBL1001 and VAC52150EBL1002. In Studies VAC52150EBL1001 and VAC52150EBL1002, both Ad26.ZEBOV/MVA-BN-Filo prime-boost and MVA-BN-Filo/Ad26.ZEBOV prime-boost regimens proved to be highly immunogenic and induced considerable humoral as well as cellular immune responses. In response to the prime dose of Ad26.ZEBOV, but not in response to the prime dose of MVA-BN-Filo, robust antibody and T cell responses were detected. In both regimens, response levels were boosted extensively.

Extending the interval between prime and boost led to increased antibody responses, with higher responses being observed in the longest vaccination schedule tested (1, 57 days). Geometric mean concentrations (GMC) (enzyme-linked immunosorbent assay [ELISA] units/mL) were approximately 1.7- to 2-fold higher with the 1, 57 day schedule than with the 1, 29 day schedule with either prime-boost regimen. In both studies, the MVA-BN-Filo/Ad26.ZEBOV regimens induced a 2.3- to 2.5-fold higher 21-day post-boost GMC than Ad26.ZEBOV/MVA-BN-Filo regimens for both the 1, 29 and 1, 57 day schedules. The increased antibody response after MVA-BN-Filo/Ad26.ZEBOV regimens was also confirmed for the 1, 15 day schedule. Here, the induced antibody response at 21 days post-boost reached a level that was comparable with or higher than with the Ad26.ZEBOV/MVA-BN-Filo 1, 29 schedule. For the MVA-BN-Filo/Ad26.ZEBOV schedules, GMC ELISA units for the 1, 15 day schedule corresponded to approximately 60% and 30% of 1, 29 and 1, 57 day schedules, respectively.

The effect on T cell responses was regimen- and schedule-specific. Extending the interval between prime and boost led to no, or only a slight, increase of cellular responses with the Ad26.Filo/MVA-BN-Filo regimen, while cellular responses decreased with increasing prime-boost intervals with the MVA-BN-Filo/Ad26.Filo regimen. Indeed, in Study VAC52150EBL1002, the highest cluster of differentiation (CD8+) T cell responses were achieved with the MVA-BN-Filo/Ad26.ZEBOV 1, 15 day prime-boost schedule, a schedule for

which data from NHP studies are not available. This regimen and schedule also yielded CD4+ T cell responses of the same or higher magnitude than all other regimens at 21-days post-boost. However, given the limited number of subjects receiving the MVA-BN-Filo/Ad26.ZEBOV 1, 15 day prime-boost schedule, data should be interpreted with caution.

The effect of higher doses of either Ad26.ZEBOV ($1x10^{11}$ vp) or MVA-BN-Filo ($4.4x10^{8}$ Inf U) provided limited or no advantages, both with respect to humoral and cellular immunity. Note, however, that the number of subjects who received higher doses of vaccine is limited, and these data should be interpreted with caution.

Induced immune responses, evaluated in Study VAC52150EBL1001, were functional; neutralizing activity of the antibody responses was detected in all subjects. The composition of the vaccine-induced T cell response was very favorable, with a high percentage of polyfunctional T cells, which are generally thought to play a role in immunologic memory and effector functions.

1.2. Overall Rationale for the Study

Protective efficacy against filovirus infections has been evaluated in several preclinical studies in NHPs, where monovalent or multivalent adenovector-based candidate vaccines were assessed in heterologous regimens of different adenovectors (Ad26 and Ad35) or the Ad26 vector and MVA-BN-Filo (see Section 1.1.1). Doses and schedules of administration varied across studies. Because of the difficulties in carrying out challenge studies that require biosafety level 4 conditions, and the large number of possible permutations to be investigated (regimen, time schedule, doses), the total number of NHPs per individual combination was relatively limited.

Nonetheless, NHP studies strongly suggested that protection against challenge with the EBOV Kikwit variant, the MARV Angola variant, and the SUDV Gulu variant was conferred with a 1, 57 day vaccination schedule using Ad26.Filo and MVA-BN-Filo (see Section 1.1.1.1 for details).

Immunogenicity data in humans for Ad26.ZEBOV (candidate monovalent vaccine) and MVA-BN-Filo regimens suggest that 1, 57 day (with either vector as prime or boost) and 1, 15 day (with MVA-BN-Filo prime followed by Ad26.Filo boost) schedules are worth evaluating (see Section 1.1.2.4 for details).

Overall, the promising preclinical data for heterologous regimens of Ad26.Filo and MVA-BN-Filo, as well as the clinical immunogenicity and safety profile of the Ad26.ZEBOV and MVA-BN-Filo regimens, justify testing a vaccination regimen based on Ad26.Filo and MVA-BN-Filo in clinical studies.

Recent clinical data from a Phase 1 study of the monovalent vaccine program (VAC52150EBL1002) exploring the safety, tolerability, and immunogenicity of a third vaccination (Ad26.ZEBOV) given at one year post-prime vaccination demonstrated a marked induction of binding antibody responses to the EBOV glycoprotein. A log increase in antibody titers within 7 days of administration of the third vaccination was observed, suggesting that the

first 2 vaccinations induced a strong memory response that gave rise to a robust anamnestic response upon re-exposure to antigen mimicked through the third vaccination. It is unknown whether a similar effect would be observed in this study for subjects of Group 3 given Ad26.Filo as a third vaccination at Day 92 post-prime, approximately 2.5 months (77 days post-boost) after the boost vaccination of Ad26.Filo. To explore whether a similar anamnestic antibody response to the 3 glycoproteins, namely Ebola, Sudan, and Marburg virus GP would be generated in the multivalent vaccine program, a third vaccination (Ad26.Filo) will be given at Day 92 post-prime to a subset of subjects in Group 3.

2. OBJECTIVES, ENDPOINTS, AND HYPOTHESIS

2.1. Objectives and Endpoints

2.1.1. Objectives

This study aims to evaluate the safety, tolerability, and immunogenicity of different MVA-BN-Filo and Ad26.Filo vaccine regimens.

Primary Objectives

To assess the safety and tolerability of:

- A prime vaccination with Ad26.Filo at the dose of $9x10^{10}$ vp boosted by MVA-BN-Filo at the dose of $5x10^{8}$ Inf U 56 days later.
- A prime vaccination with MVA-BN-Filo at the dose of $5x10^{8}$ Inf U boosted by Ad26.Filo at the dose of $9x10^{10}$ vp either 14 or 56 days later.
- A third vaccination using Ad26.Filo at the dose of $9x10^{10}$ vp at Day 92.

Secondary Objectives

- To assess humoral immune responses of the 3 multivalent filovirus regimens tested, as measured by ELISA against each GP.

Exploratory Objectives

- To assess humoral and cellular immune responses of the regimens tested, including but not limited to, virus neutralization assay, functional and/or biophysical characterization of antibody responses, enzyme-linked immunospot assay (ELISpot), and intracellular cytokine staining (ICS).
- To assess humoral immune responses, as measured by ELISA to EBOV GP, of the monovalent Ad26.ZEBOV regimen (group 4) to evaluate if response levels of the multivalent program are consistent with the ones observed in the monovalent Ad26.ZEBOV clinical studies.

2.1.2. Endpoints

Primary Endpoints

The safety and tolerability endpoints are:

- Unsolicited AEs from signing of the ICF onwards until 28 days post-boost, and again from the third vaccination until 28 days thereafter (for a subset of subjects in Group 3) (note: events that started before the third vaccination but are still present at the time of third vaccination should also be recorded).
- Solicited local and systemic AEs (reactogenicity) until 7 days after each study vaccine administration.
- SAEs from signing of the ICF onwards until the end of the study.

Secondary Endpoints

Humoral Immune Responses (Serology)

- Binding antibody responses against EBOV, MARV, and SUDV GPs.

Exploratory Endpoints

Additional exploratory analyses may be performed to further investigate study vaccine-elicited immune responses. Exploratory endpoints may include, but might not be limited to, the following:

Humoral Immune Responses (Serology)

- Neutralizing antibody responses against EBOV, MARV, and/or SUDV GPs, defined as the serum titer that is able to inhibit viral infection (50%, 80%, or 90% inhibitory concentration).
- Binding and/or neutralizing antibody responses against adenoviral vector (Ad) and/or MVA vector.
- Characterization of study vaccine-elicited antibodies.

Cell-mediated Immune Responses

- Presence and functional capacity of T cells after pathogen-specific stimulation of peripheral blood mononuclear cells with EBOV, MARV, and/or SUDV GP-specific peptides (ELISpot and ICS).
- Transcriptome analysis of immune cell populations.

Refer to Section 9 for related evaluations.

2.2. Hypothesis

The study is exploratory, and no formal statistical hypothesis testing is planned.

3. STUDY DESIGN AND RATIONALE

3.1. Overview of Study Design

This is a Phase 1, randomized, double-blind, placebo-controlled, first-in-human study exploring the safety, tolerability, and immunogenicity of 3 heterologous prime-boost regimens compared with placebo using MVA-BN-Filo at a dose of $5x10^8$ Inf U and Ad26.Filo at a dose of $9x10^{10}$ vp (Groups 1, 2, and 3). In addition, a group assessing a monovalent Ad26.ZEBOV prime at a dose of $5x10^{10}$ vp at Day 1 and MVA-BN-Filo boost at a dose of $1x10^8$ Inf U at Day 57 (Group 4) will be included in the study to serve as a tolerability and immunogenicity control arm. Placebo recipients are included for blinding purposes and safety analyses, and will provide control specimens for immunogenicity assays. The study will be conducted in healthy adult subjects aged ≥18 to ≤50 years not previously vaccinated with a filovirus candidate vaccine and with no known previous exposure to EBOV, MARV, SUDV, or TAFV. A total of 72 healthy adult male and female subjects are planned to be enrolled.

Additionally, this study will explore whether a third vaccination (Ad26.Filo) given at Day 92 post-prime to a subset of subjects in Group 3 would induce a similar anamnestic antibody response to the 3 glycoproteins, namely Ebola, Sudan and Marburg. The first 8 subjects in Group 3 who are willing to enroll for the third vaccination, will receive a third vaccination at Day 92. In an observer-blind manner, subjects who previously received placebo will receive placebo a third time and subjects who previously received MVA-BN-Filo/Ad26.Filo vaccination will receive Ad26.Filo at the dose of $9x10^{10}$ vp as third vaccination. After enrollment of the 8 subjects, the unblinded monitor and unblinded pharmacist will assess whether 7 subjects who previously received MVA-BN-Filo/Ad26.Filo vaccination have been enrolled. If less than 7 subjects of the active vaccine regimen have been enrolled, 2 additional subjects will be enrolled. If at least 7 subjects of the active vaccine regimen have been enrolled, no further subjects will be enrolled. The aim is to enroll 7 or 8 subjects who will receive Ad26.Filo as third vaccination. Subjects who receive the third vaccination will follow a separate time and events schedule as of Day 92 while the remainder of Group 3 who did not receive the third vaccination would continue in the study according to the original time and event schedule for Group 3.

The study will last approximately 395 days per subject and will consist of a screening period of up to 35 days, a vaccination period in which the subjects will be vaccinated at baseline (Day 1) followed by a boost on Day 15 or 57, and a third vaccination at Day 92 (subset of subjects in Group 3), and a post last dose follow-up until all subjects have had their Day 360 (359 days post-prime) visit or withdrew earlier.

Subjects will be enrolled into 4 different groups of 18 healthy subjects each to receive 1 of the 4 following treatment regimens:

- Ad26.Filo prime at Day 1/MVA-BN-Filo boost at Day 57 (Group 1).

- MVA-BN-Filo prime at Day 1/Ad26.Filo boost at Day 57 (Group 2).
- MVA-BN-Filo prime at Day 1/Ad26.Filo boost at Day 15/a subset of subjects will receive a third vaccination (Ad26.Filo) at Day 92 (Group 3).
- Ad26.ZEBOV prime at Day 1/MVA-BN-Filo boost at Day 57 (Group 4).

Subjects will be randomly assigned within groups to active vaccine or placebo (0.9% normal saline) in a 5:1 ratio (ie, 15 active: 3 placebo).

Groups 1 and 2 will start with 4 sentinel subjects in each group (3 active/1 placebo per group). If no pausing rule is met by Day 4 (ie, at least 72 hours after prime vaccination) in the sentinel cohorts (see Section 11.7), recruitment will open to the remaining subjects in Groups 1 and 2 (12 active/2 placebo per group). The randomization of Group 4 does not depend on the outcome of the sentinel cohorts because the safety profile of this regimen has been assessed in other studies; therefore, Group 4 randomization will start immediately after assignment of the sentinel subjects of Groups 1 and 2 and will be randomized as a whole (15 active/3 placebo). After randomization of Groups 1, 2, and 4, Group 3 will be randomized as a whole (15 active/3 placebo).

Predefined pausing rules will guide study progression under the supervision of an independent Data Monitoring Committee, which will review and evaluate only safety-related data in this study (see Section 11.7). Therefore, the term Safety Monitoring Committee (SMC) will be used throughout this document.

The blinded principal investigator and sponsor's medical monitor will be responsible for the safety monitoring of the study. If at least 1 pre-specified pausing rule is met, the principal investigator will install a pause during which vaccinations will be withheld and will request an independent SMC meeting. If no pausing rule is met in the sentinel cohort of a given group by Day 4, the decision to open recruitment to the remaining subjects of that group will be made by the investigator, without SMC review. Similarly, if no pausing rule is met, the decision to start randomization of Group 3 will be made by the investigator, without SMC review. For details on the SMC, see Section 11.6. For details on the pausing rules, see Section 11.7.

The different study vaccination schedules are summarized in Table 1.

Table 1: Vaccination Schedules

Group	N			Day 1	Day 57	
1	18	3	Sentinel cohort	Ad26.Filo, 9x10^{10} vp	MVA-BN-Filo, 5x10^{8} Inf U	-
		1	Sentinel cohort	Placebo	Placebo	-
		12	Post-sentinel cohort	Ad26.Filo, 9x10^{10} vp	MVA-BN-Filo, 5x10^{8} Inf U	-
		2	Post-sentinel cohort	Placebo	Placebo	-
2	18	3	Sentinel cohort	MVA-BN-Filo, 5x10^{8} Inf U	Ad26.Filo, 9x10^{10} vp	-
		1	Sentinel cohort	Placebo	Placebo	-
		12	Post-sentinel cohort	MVA-BN-Filo, 5x10^{8} Inf U	Ad26.Filo, 9x10^{10} vp	-
		2	Post-sentinel cohort	Placebo	Placebo	-
4	18	15	-	Ad26.ZEBOV, 5x10^{10} vp	MVA-BN-Filo, 1x10^{8} Inf U	-
		3	-	Placebo	Placebo	-
**Group**	**N**			**Day 1**	**Day 15**	**Day 92[a]**
3	18	15	-	MVA-BN-Filo, 5x10^{8} Inf U	Ad26.Filo, 9x10^{10} vp	Ad26.Filo, 9x10^{10} vp
		3	-	Placebo	Placebo	Placebo

Inf U = infectious units; N = number of subjects to receive study vaccine (active vaccine or placebo [0.9% saline]);vp = viral particles.

a. A subset of subjects in Group 3 will be enrolled in a third vaccination substudy at Day 92. The first 8 subjects who are willing to participate will receive a third vaccination at Day 92 based on their previous vaccination. After enrollment of the 8 subjects, the unblinded monitor and unblinded pharmacist will assess whether 7 subjects who previously received MVA-BN-Filo/Ad26.Filo vaccination have been enrolled. If less than 7 subjects of the active vaccine regimen have been enrolled, 2 additional subjects will be enrolled. If at least 7 subjects of the active vaccine regimen have been enrolled, no further subjects will be enrolled.

After each vaccination, subjects will remain at the site for observation and vital sign assessments for at least 60 minutes (or 30 minutes for subjects who received a third vaccination), or longer if deemed necessary by the investigator. Reactogenicity and vital signs will be assessed at 30 (±5) minutes, and vital signs will be checked again at 60 (±5) minutes except for subjects who received a third vaccination. If an event occurs during the 60-minute observation period (or 30-minute observation period for subjects who received a third vaccination), the highest overall grade of the event, across the entire 60 minutes (or 30 minutes for subset of Group 3), will be reported. Subjects will be instructed to contact the investigator immediately if they experience any AE about which they are concerned. Subjects in the sentinel cohorts will be contacted approximately 24 hours after study vaccination to confirm that no pre-specified pausing rules have been met. Subjects will use a diary to record oral body temperature and to document symptoms of unsolicited and solicited local and systemic AEs in the evening after each vaccination and then daily for the next 7 days (see Section 9.1.1). If more than 1 measurement is made or if an AE is reported more than once on any given day, the highest value or highest severity of that day will be recorded. Refer to Section 12.1.3 for grading of severity of solicited AEs. The investigator or designee will document unsolicited AEs from signing of the ICF

onwards until 28 days post-boost, and again from the third vaccination until 28 days thereafter (for a subset of Group 3) (note: events that started before the third vaccination but are still present at the time of third vaccination should also be recorded), and SAEs and IREs from signing of the ICF onwards until the end of the study. In addition, the investigator or the designee will collect samples for safety assessments (hematology, chemistry, and urinalysis) and assessments of immune responses, at the time points indicated in the Time and Events Schedule.

When all subjects have completed their 21-day post last dose visit (Day 78 in Groups 1, 2, and 4, Day 36 in Group 3, and Day 113 for a subset of Group 3), or withdrew earlier, a primary analysis of all data collected up to that point will be performed. The final analysis will be performed when all subjects in Groups 1 to 4 have completed the last study visit or withdrew earlier. Additional interim analyses may be performed at the sponsor's discretion.

3.2. Study Design Rationale

Vaccine Regimens and Schedules

The regimens and schedules evaluated in Groups 1, 2, and 3 of this study are aimed at providing information on the safety and immunogenicity of 3 different prime-boost schedules with Ad26.Filo and MVA-BN-Filo. Additionally, in a subset of subjects in Group 3, the safety and immunogenicity of a third vaccination using Ad26.Filo at the dose of 9×10^{10} vp at Day 92 will be evaluated. A regimen using the candidate monovalent vaccine Ad26.ZEBOV as prime and MVA-BN-Filo as boost will be assessed in Group 4 and is included in the study to serve as a tolerability and immunogenicity control arm.

Extrapolation of NHP data to humans should be made with caution; nonetheless, NHP challenge data as well as immunogenicity data in humans (using Ad26.ZEBOV and MVA-BN-Filo regimens) (see Section 1.1 for details) suggest that a 1, 57 day vaccination schedule is worth evaluating, with either vaccine (ie, MVA-BN-Filo or Ad26.Filo) as prime or boost, because humoral immune responses were highest with this schedule in ongoing clinical studies evaluating Ad26.ZEBOV and MVA-BN-Filo prime-boost regimens. Immunogenicity data in humans for Ad26.ZEBOV and MVA-BN-Filo regimens further suggest that the 1, 15 day vaccination schedule with MVA-BN-Filo prime/Ad26.Filo boost is worth evaluating, because the highest CD8+ T cell responses were achieved in an ongoing clinical study with the MVA-BN-Filo/Ad26.ZEBOV schedule (see Section 1.1.2.4 for details).

Overall, the proposed study design allows assessment in humans of the most promising regimens and schedules identified in NHP studies with Ad26.Filo and MVA-BN-Filo and in human studies with the monovalent candidate vaccine Ad26.ZEBOV and MVA-BN-Filo (in terms of immune responses). The staggered recruitment will permit careful evaluation of safety while avoiding undue exposure of subjects to unexpected toxicity. Finally, the availability of a control arm will allow useful data to be generated for the assessment of possible immunologic interference among the 3 different antigens included in the Ad26.Filo formulation, albeit only for the GP of the EBOV Mayinga variant.

Dose Selection

This study plans to assess Ad26.Filo at a dose of $9x10^{10}$ vp ($3x10^{10}$ vp for each of the 3 monovalent vaccine components) and MVA-BN-Filo at a dose of $5x10^{8}$ Inf U.

The Ad26.Filo dose of $9x10^{10}$ vp was selected for Groups 1, 2, and 3 to be within the range of, but not higher than, the highest dose of Ad26.ZEBOV assessed in humans ($1x10^{11}$ vp, assessed in Study VAC52150EBL1002), where only mild and transient AEs were observed. In addition, this Ad26.Filo dose is below the dose of $1.2x10^{11}$ vp ($4x10^{10}$ vp for each of the 3 monovalent vaccine components) tested in NHPs, where heterologous prime-boost regimens of Ad26- and MVA-based vaccines were well tolerated at doses of up to $1.2x10^{11}$ vp and $5x10^{8}$ $TCID_{50}$, respectively. These data suggest that a dose of $9x10^{10}$ vp for Ad26.Filo will not lead to significant safety or tolerability issues.

The MVA-BN-Filo dose of $5x10^{8}$ Inf U was selected for Groups 1, 2, and 3, as this was the dose tested in nonclinical studies in NHPs, where it was well tolerated (see Section 1.1.1). Further, in humans (Study VAC52150EBL1002), MVA-BN-Filo doses of up to $4.4x10^{8}$ $TCID_{50}$ were evaluated, and only mild and transient AEs were observed in the majority of subjects.

Note that in the present study, the dose of MVA-BN-Filo is expressed as Inf U, whereas in nonclinical studies and the initial clinical studies with the prime-boost vaccine regimens of Ad26.ZEBOV and MVA-BN-Filo, the dose of MVA-BN-Filo was also described in units of $TCID_{50}$. These units can be used interchangeably: $1.0x10^{8}$ Inf U/mL corresponds to $1.0x10^{8}$ $TCID_{50}$.

The MVA-BN-Filo dose of $1x10^{8}$ Inf U was selected for Group 4 (Ad26.ZEBOV prime/MVA-BN-Filo boost) to permit comparison with ongoing clinical studies evaluating heterologous prime-boost regimens of MVA-BN-Filo at a dose of $1x10^{8}$ Inf U and Ad26.ZEBOV at a dose of $5x10^{10}$ vp.

Findings for both Ad26.ZEBOV and MVA-BN-Filo are consistent with safety profiles observed for similar vaccines.

Control and Blinding

Placebo recipients are included for blinding purposes and safety analyses, and will provide control specimens for immunogenicity assays. In addition to placebo control, Ad26.ZEBOV prime/MVA-BN-Filo boost will be assessed in Group 4 to serve as a tolerability and immunogenicity control arm.

Randomization will be used to minimize bias in the assignment of subjects to vaccine regimens, to increase the likelihood that known and unknown subject attributes (eg, demographic and baseline characteristics) are evenly balanced across treatment groups, and to enhance the validity of statistical comparisons across treatment groups.

Blinding will be guaranteed by preparation of study vaccine by an independent unblinded site research pharmacist (not involved in any other study activities) and by the administration of

vaccine in a masked syringe by a blinded study vaccine administrator (see Definition of Terms, and see Section 5 for details of blinding).

4. SUBJECT POPULATION

Screening for eligible subjects will be performed within 35 days before administration of the study vaccine. Laboratory screening assessments must be performed within 28 days before Day 1.

Enrollment will be based solely on the eligibility criteria and will be open to both sexes and all races/ethnicities. Sex/minority breakdown cannot be predicted for this study but should follow the general population distribution. Pregnant women and women who are breastfeeding will not be eligible for inclusion, as limited data are available for the use of Ad26.ZEBOV and MVA-BN-Filo in humans. In addition, Ad26.Filo has not been studied in humans.

The inclusion and exclusion criteria for enrolling subjects in this study are described in the following 2 subsections. If there is a question about the inclusion or exclusion criteria below, the investigator should consult with the appropriate sponsor representative before enrolling a subject in the study. Waivers are not allowed.

For a discussion of the statistical considerations of subject selection, refer to Section 11.2.

4.1. Inclusion Criteria

Each potential subject must satisfy all of the following criteria to be enrolled in the study:

1. Able to read and provide consent after completing the informed consent process.

2. Man or woman aged ≥18 to ≤50 years.

3. Body mass index (BMI) of ≥18.5 and <35.0 kg/m^2.

4. Healthy on the basis of physical examination, medical history, and the investigator's clinical judgment.

5. Criterion modified per Amendment 1

5.1 Must have acceptable laboratory parameters* within 28 days before Day 1. Acceptable laboratory parameters include the following:

   a. Hemoglobin: within laboratory normal sex-specific range.
   b. Total white blood cell (WBC) count not <2500 cells/mm^3 and differential as follows: absolute neutrophil count not <1500 cells/mm^3 and absolute lymphocyte count within laboratory normal range; eosinophil count ≤Grade 1 (based on Food and Drug Administration [FDA] toxicity tables; see Attachment 1); basophil and monocyte counts each <2x upper limit of normal (ULN) range.
   c. Platelets: within laboratory normal range.

d. Urinalysis (clean urine sample): protein and blood <1+, glucose negative.

Note: For women: in case of menstruation, urinalysis must be postponed, but a result should be available before randomization.

e. ALT/aspartate aminotransferase (AST): ≤1.1x institutional ULN.

f. Serum creatinine: ≤1.1x institutional ULN.

g. Prothrombin time: ≤1.1x institutional ULN.

h. Activated partial thromboplastin time: ≤1.1x institutional ULN.

Note: If the acceptable laboratory screening parameters listed above are out-of-range, repeat of screening tests is permitted once, provided there is an alternative explanation for the out-of-range value. When reference is made to the within laboratory normal range, FDA toxicity grades should not be considered for subject's inclusion. Due to an overlap of the local laboratory normal ranges and FDA toxicity grade for some parameters, a subject remains eligible if the lab value is within the normal range, while being a grade 1 toxicity.*

***Note: Grade 1 abnormalities for laboratory tests other than those covered in the list above are considered acceptable if not clinically significant. Toxicity grade for laboratory values will be determined according to a toxicity grading scale adapted from the FDA Toxicity Grading Scale for Healthy Adult and Adolescent Volunteers Enrolled in Preventive Vaccine Clinical Trials included in* Attachment 1.

6. All women of childbearing potential must:

   a. Have a negative serum (β-human chorionic gonadotropin [β-hCG]) pregnancy test at screening.

   b. Have a negative urine β-hCG pregnancy test immediately prior to each study vaccine administration.

7. Criterion modified per Amendment 1, 2, and 3

7.1 Contraceptive use by women should be consistent with local regulations regarding the use of contraceptive methods for subjects participating in clinical studies.

Before randomization, a woman must be either:

a. Of childbearing potential and

- Practicing an effective method of birth control (failure rate of <1% per year when used consistently and correctly) from 28 days before prime vaccination until at least 3 months after the last vaccination, consistent with local regulations regarding the use of birth control methods for subjects participating in clinical studies: Examples of effective contraceptives include:
  - User-independent methods:
    Injectable or implantable progestogen-only hormone contraception associated with inhibition of ovulation; intrauterine device; intrauterine hormone-releasing

system; vasectomized partner; and sexual abstinence (sexual abstinence is considered an effective method **only** if defined as refraining from heterosexual intercourse from 28 days before the prime vaccination until at least 3 months after the last vaccination; the reliability of sexual abstinence needs to be evaluated in relation to the duration of the study and the preferred and usual lifestyle of the subject).

- User-dependent methods:
  Combined (estrogen- and progestogen-containing) hormonal contraception associated with inhibition of ovulation: oral, intravaginal, and transdermal; vaginal ring; progestogen-only hormone contraception associated with inhibition of ovulation: oral and injectable.

<u>Note</u>: Typical use failure rates may differ from those when used consistently and correctly.

<u>Note</u>: If the childbearing potential changes after start of the study (eg, woman who is not heterosexually active becomes active), a woman must begin an effective method of birth control, as described above.

b. Not of childbearing potential defined as:

- Post-menopausal:
  A post-menopausal state is defined as amenorrhea for ≥12 months without an alternative medical cause in women >45 years of age, or amenorrhea for >6 months but <12 months and 1 measurement of serum follicle-stimulating hormone (FSH) level >40 IU/L or mIU/mL in women of any age.
- Permanently sterile:
  Permanent sterilization methods include hysterectomy, bilateral salpingectomy, bilateral tubal occlusion/ligation procedures, and bilateral oophorectomy.

Women not of childbearing potential are not required to use the birth control methods recommended above.

8. Criterion modified per Amendment 3

8.1 A woman must agree not to donate eggs (ova, oocytes) for the purposes of assisted reproduction from the start of screening onwards until at least 3 months after the last vaccination.

9. Criterion modified per Amendment 3

9.1 During the study and for a minimum of 3 months after receiving the last vaccination, a man who has not had a vasectomy:

- who is sexually active with a woman of childbearing potential must agree to consistently use a barrier method of contraception (eg, condom with spermicidal foam/gel/film/cream/suppository)

- who is sexually active with a woman who is pregnant must use a condom
- must agree not to donate sperm.

Note: Spermicides should only be used if considered appropriate by the subject and his physician, as they may increase the risk of HIV transmission.

10. Subject must be available and willing to participate for the duration of the study visits and follow-up.

11. Subject must be willing to provide verifiable identification.

12. Subject must have a means to be contacted.

4.2. Exclusion Criteria

Any potential subject who meets any of the following criteria will be excluded from participating in the study.

1. Has been vaccinated with a candidate filovirus vaccine.

2. Has been diagnosed with disease caused by EBOV, MARV, SUDV, or TAFV or exposed to EBOV, MARV, SUDV, or TAFV, including subjects who traveled to epidemic filovirus areas in West Africa during the last 2 years (ie, since the start of the last *Ebolavirus* outbreak) should be excluded from the study.

3. Criterion modified per Amendment 3

3.1 Subjects who anticipate traveling to epidemic filovirus areas before the primary analysis (21-day post last dose visit) will be excluded from enrollment into the study.

4. Has received any Ad26- or MVA-based candidate vaccines in the past.

5. Known allergy or history of anaphylaxis or other serious adverse reactions to vaccines or vaccine products (including any of the constituents of the study vaccines, eg, polysorbate 80, ethylenediaminetetraacetic acid, or L-histidine for the Ad26.Filo and Ad26.ZEBOV vaccines; and Tris-hydroxymethyl-aminomethane (Tris) for the MVA-BN-Filo vaccine), including known allergy to egg, egg products, and aminoglycosides.

6. Acute illness (this does not include minor illnesses such as diarrhea or mild upper respiratory tract infection) or body temperature ≥38.0°C on Day 1.

7. Chronic active hepatitis B or hepatitis C infection, documented by hepatitis B surface antigen and hepatitis C antibody, respectively.

8. HIV type 1 or type 2 infection.

9. A woman who is pregnant or breastfeeding, or planning to become pregnant while enrolled in the study or within 3 months after the boost vaccination.

10. Bleeding or clotting disorders.

11. Any clinically significant acute or chronic medical condition that, in the opinion of the investigator, would preclude participation (eg, history of seizure disorders, autoimmune diseases, any spleen disease, active malignancy, active tuberculosis, asthma, other systemic infections).

12. History of malignancy other than squamous cell or basal cell skin cancer, unless there has been surgical excision that is considered by the investigator to have achieved cure. Subjects with a history of skin cancer must not be vaccinated at the previous tumor site.

13. Post-organ and/or stem cell transplant whether or not with chronic immunosuppressive therapy.

14. Major surgery (per the investigator's judgment) within 4 weeks prior to study entry or planned major surgery through the course of the study.

15. History of myocarditis, pericarditis, cardiomyopathy, transient ischemic attack or stroke, myocardial infarction, angina, coronary artery disease, congestive heart failure, clinically significant arrhythmia (including any arrhythmia requiring medication, treatment, or clinical follow-up).

16. Electrocardiograms with clinically significant findings (per the investigator's judgment) or features that would interfere with the assessment of myocarditis/pericarditis, including any of the following:

    a. Conduction disturbance (complete left or complete right bundle branch block or nonspecific intraventricular conduction disturbance with prolongation of any of the following intervals: QRS ≥120 ms, PR interval ≥210 ms, any second- or third-degree atrioventricular block, or prolongation of the QT interval corrected according to Bazett's formula [QTcB] [>450 ms]).
    b. Significant repolarization (ST-segment or T-wave) abnormality.
    c. Significant atrial or ventricular arrhythmia; frequent atrial or ventricular ectopy (eg, frequent premature atrial contractions, 2 premature ventricular contractions in a row).
    d. ST-elevation consistent with ischemia or evidence of past or evolving myocardial infarction.

17. History of diabetes mellitus type 1 or type 2, including cases controlled with diet alone.

    Note: History of isolated gestational diabetes is not an exclusion criterion.

18. Thyroidectomy, or thyroid disease requiring medication during the last 12 months.

19. Uncontrolled hypertension, defined as systolic blood pressure ≥140 mm Hg at enrollment or diastolic blood pressure ≥90 mm Hg at enrollment.

    Note: In the case of diastolic values ≥90 mm Hg or systolic values ≥140 mm Hg, the measurement can be repeated after 30 minutes rest, and the subject can be enrolled in the case of 2 consecutive measurements fulfilling the inclusion criterion. Subjects who have a diastolic blood pressure value ≥90 mm Hg or a systolic blood pressure value ≥140 mm Hg prior to the prime vaccination can receive the vaccination if the elevated blood pressure is considered to be not clinically significant and the subject had normal blood pressure at screening.

20. Major psychiatric illness and/or substance abuse problems during the past 12 months that in the opinion of the investigator would preclude participation.

21. Receipt or planned administration of licensed live attenuated vaccines from 30 days before Day 1 (until 30 days after the last study vaccine administration), or receipt or planned administration of any other licensed vaccine from 15 days before Day 1 (until 15 days after the last study vaccine administration).

22. Use of investigational therapeutic agents within 3 months from the start of screening.

23. Current or planned participation in another clinical study during the study period.

    Note: Participation in an observational clinical study is allowed.

24. Receipt of blood products or immunoglobulin in the past 3 months.

25. Donation of a unit of blood within 8 weeks before Day 1 or planning to donate blood from the start of screening onwards until at least 3 months after the last administration of study vaccine and within 1 month of planned immunogenicity visits (ie, 1 month prior to 6-month and 12-month visits).

26. Current or past abuse of recreational or narcotic drugs.

    Note: Urine will be tested to check for current use of amphetamines, benzodiazepines, cocaine, cannabinoids, and opioids.

27. Current alcohol use judged by the investigator to potentially interfere with subject study adherence.

28. History of chronic urticaria (recurrent hives).

29. Chronic or recurrent use of medications that modify host immune response, eg, cancer chemotherapeutic agents, systemic corticosteroids.

30. Inability to communicate reliably with the investigator.

31. Unlikeliness to adhere to the requirements of the study, in the opinion of the investigator.

32. Study-site employees and family members of the investigator.

33. Employees of the DMID and the Center for Biologics Evaluation & Research.

NOTE: The investigator should ensure that all study enrollment criteria have been met at the end of the screening period. If a subject's clinical status changes (including any available laboratory results or the receipt of additional medical records) after screening but before Day 1 such that the subject no longer meets all eligibility criteria, then the subject should be excluded from participation in the study. Section 17.4 describes the required documentation to support meeting the enrollment criteria.

4.3. Prohibitions and Restrictions

Potential subjects must be willing and able to adhere to the following prohibitions and restrictions during the course of the study to be eligible for participation:

1. Traveling to epidemic filovirus areas is prohibited from screening onwards until the primary analysis.

   Note: Subjects who travel to epidemic filovirus areas (as noted in Exclusion Criterion #3) after the primary analysis must return at least 1 month before any blood sample collection is performed. Any travel to epidemic filovirus areas should be documented in the Case Report Form (CRF). Subjects travelling to epidemic filovirus areas will be excluded from follow-up collection of blood for immunogenicity assessments if they contract disease caused by a filovirus.

2. Agree to follow the contraceptive requirements as noted in the inclusion criteria (see Section 4.1).

3. Women must agree not to donate eggs (ova, oocytes) for the purposes of assisted reproduction during study participation (from screening onwards until at least 3 months after the last administration of study vaccine) (see Section 4.1).

4. Men must agree not to donate sperm during study participation (from screening onwards until at least 3 months after the last administration of study vaccine) (see Section 4.1).

5. Subjects must agree not to donate blood or blood products during study participation (from screening onwards until at least 3 months after the last administration of study vaccine).

6. Refer to Section 8 for details regarding prohibited and restricted therapy during the

study.

5. TREATMENT ALLOCATION AND BLINDING

Vaccine Schedule Allocation

Subjects will be randomly assigned within groups to active vaccine or placebo based on a computer-generated randomization schedule prepared before the study by or under the supervision of the sponsor. Randomization will start with 4 sentinel subjects per group in Groups 1 and 2 (3 active/1 placebo). If no pausing rule is met by Day 4 (ie, at least 72 hours after prime vaccination) in the sentinel cohorts (see Section 11.7), recruitment will open to the remaining subjects in Groups 1 and 2 (12 active/2 placebo per group). The randomization of Group 4 (15 active/3 placebo) does not depend on the outcome of the sentinel cohorts because the safety profile of this regimen has been assessed in other studies; therefore, Group 4 randomization will start immediately after assignment of the sentinel subjects of Groups 1 and 2 and will be randomized as a whole (15 active/3 placebo). After randomization of Groups 1, 2, and 4, Group 3 will be randomized as a whole (15 active/3 placebo). The randomization within each group will be balanced by using randomly permuted blocks.

A subset of subjects in Group 3 will receive a third vaccination at Day 92. In this substudy, the first 8 subjects who are willing to participate will receive either placebo or Ad26.Filo as third vaccination based on their previous vaccinations. The aim is to enroll 7 to 8 subjects who will receive Ad26.Filo as the third vaccination.

Blinding

Subjects, clinical staff, and site personnel will be blinded to the study vaccine allocation until the end of study (Day 360), except for the unblinded site research pharmacist, who has primary responsibility for study vaccine preparation and dispensing. To preserve blinding, the unblinded site research pharmacist will place a blinding tape or overlay on the syringe to mask its content. The vaccine will then be sent to a blinded study vaccine administrator (see Definition of Terms) for administration to the subject.

When all subjects have had their 21-day post last dose visit or withdrew earlier, a primary analysis on all data collected up to that point will be conducted. The database will be locked, and the study will be unblinded for the primary analysis. Subjects, clinical staff, and site personnel will remain blind to the study vaccine allocation until the end of study (Day 360) (except for programming, statistics, clinical and clinical immunology personnel, the sponsor committee involved in making future decisions for the program, and those with primary responsibility for study vaccine preparation and dispensing). All subjects will be followed up until Day 360.

The administration of the third vaccination to a subset of subjects in Group 3 will be observer-blind. The site, subjects, and sponsor will remain blinded to the study vaccine allocation until the end of study (Day 360).

The investigator will be provided with a sealed randomization code for each subject, containing coded details of the treatment in the double-blind phase. All randomization codes, whether opened or sealed, will be collected after the end of the subject's participation in the study.

Under normal circumstances, the blind must not be broken until the end of study (Day 360). Otherwise, the blind should be broken only if a specific emergency treatment/course of action would be dictated by knowing the treatment status of the subject. In such cases, the investigator may, in an emergency, determine the identity of the study vaccine. It is recommended that the investigator contact the sponsor or its designee if possible to discuss the particular situation before breaking the blind. Telephone contact with the sponsor or its designee will be available 24 hours per day, 7 days per week. In the event the blind is broken, the sponsor must be informed as soon as possible. The date and reason for the unblinding must be documented in the appropriate section of the CRF, and in the source document.

If the randomization code is broken by the investigator or the study-site personnel, the subject must be withdrawn from the study and must be followed as appropriate. If the code is broken by the sponsor for safety reporting purposes, the subject may remain in the study.

6. DOSAGE AND ADMINISTRATION

An overview of the vaccination schedules is provided in Table 1. All subjects will receive active vaccine (MVA-BN-Filo, Ad26.Filo, or Ad26.ZEBOV) or placebo (0.9% normal saline), according to randomization, on Days 1 and 57 (Groups 1, 2, and 4) or on Days 1, 15, and 92 (Group 3, Day 92 only in subset of subjects) at the following dose levels:

- Ad26.Filo $9x10^{10}$ vp or placebo on Day 1 (Group 1), Day 57 (Group 2), Day 15 (Group 3), or Day 92 (subset of Group 3).
- MVA-BN-Filo at $5x10^{8}$ Inf U or placebo on Day 1 (Groups 2 and 3) or Day 57 (Group 1).
- Ad26.ZEBOV $5x10^{10}$ vp or placebo on Day 1 (Group 4).
- MVA-BN-Filo $1x10^{8}$ Inf U or placebo on Day 57 (Group 4).

Study vaccines or placebo will be administered as IM injections as described in Section 14.1 in the upper arm. When choosing an arm for the injection, the blinded study vaccine administrator should consider whether there is an arm injury, local skin problem, and/or significant tattoo that precludes administering the injection or will interfere with evaluating the arm after injection and should not administer the vaccine at such a location. For each subject, the boost vaccination should be administered in the opposite deltoid from the prime vaccination unless local site reaction cannot be assessed reliably in the opposite arm, and it should be recorded in the CRF in which arm the vaccination has been administered. The third vaccination can be administered in either deltoid. No local or topical anesthetic will be used prior to the injection.

The Site Investigational Product Procedures Manual specifies the maximum time that will be allowed between preparation and administration of the study vaccine.

7. TREATMENT COMPLIANCE

All study vaccines will be administered by a blinded study vaccine administrator (see Definition of Terms). The date and time of each study vaccine administration will be recorded in the CRF.

8. PRESTUDY AND CONCOMITANT THERAPY

Prestudy therapies up to 30 days prior to the start of screening must be recorded in the CRF.

Concomitant therapies must be recorded from screening onwards until the 21-day post last dose visit. Thereafter, concomitant therapies should be recorded only if given in conjunction with SAEs that meet the criteria outlined in Section 12.3.2.

Vaccination with a licensed live attenuated vaccine within 30 days before Day 1 and within 30 days after the last study vaccine administration, and vaccination with any other licensed vaccine within 15 days before Day 1 or within 15 days after the last study vaccine administration, are prohibited (per Exclusion Criterion #21). Medically indicated vaccines (eg, influenza [except live attenuated influenza vaccine], tetanus, hepatitis A, hepatitis B, or rabies) are not prohibited, but should be given at least 15 days before (or at least 15 days after) administration of study vaccine to avoid potential confusion of adverse reactions. However, if a vaccine is indicated in the post-exposure setting (eg, rabies or tetanus), it must take priority over the study vaccine.

Study subjects can receive medications, such as acetaminophen, non-steroidal anti-inflammatory drugs, or antihistamines as needed. Use of these medications as routine prophylaxis within 24 hours prior to study vaccine administration is discouraged.

Chronic or recurrent use of medications that modify host immune response (such as systemic corticosteroids or cancer chemotherapeutic agents) are prohibited (per Exclusion Criterion #29).

Subjects must use adequate birth control measures (see Section 4.1).

The sponsor must be notified in advance (or as soon as possible thereafter) of any instances in which prohibited therapies are administered. Prohibited therapies will be captured as protocol deviations.

9. STUDY EVALUATIONS

9.1. Study Procedures

9.1.1. Overview

The Time and Events Schedule summarizes the frequency and timing of safety, tolerability, and immunogenicity assessments applicable to this study. Additional study visits may be required if, in the investigator's opinion, further clinical or laboratory evaluation is needed.

Additional serum or urine pregnancy tests may be performed, as determined necessary by the investigator or required by local regulation, to establish the absence of pregnancy at any time during the subject's participation in the study.

Subjects will be provided with a thermometer, ruler, and subject diary to measure and record oral body temperature, and unsolicited and solicited local (at injection site) and systemic events. The diary will include instructions how to capture the data and grading scales to assess severity of the symptoms. The study staff is responsible for providing appropriate training to the subject to avoid missing or incorrect data (refer to Study Training Manual). The diary card will be reviewed by the study personnel at visits indicated on the Time and Events Schedule.

From screening onwards until the end of the study, the total blood volume to be collected from each subject will be approximately 890 mL for Groups 1, 2, and 4 and 770 mL for Group 3 (940 mL for the subset of subjects in Group 3 receiving a third vaccination). The maximal amount of blood that will be drawn over a period of approximately 50 to 60 days will be 490 mL in all groups (Groups 1, 2, and 4: between Days 15 and 78, and between Days 29 and 92; Group 3: between Days 1 and 50).

Repeat or unscheduled samples may be taken for safety reasons or for technical issues with the samples.

9.1.2. Visit Windows

Visit windows that will be allowed are summarized in Table 2.

If a subject did not receive study vaccine on the planned day of vaccination, the timings of the next visits post-vaccination (see Time and Events Schedule) will be determined relative to the actual day of vaccination.

Table 2: Visit Windows

Visit Description	Day	Window
Boost Vaccination[a]	Day 57 for Groups 1, 2, and 4; Day 15 for Group 3	±1 day
Three days post-vaccination (prime and boost)	Days 4 and 60 for Groups 1, 2, and 4; Days 4 and 18 for Group 3	±1 day
Seven days post-vaccination (prime and boost)	Days 8 and 64 for Groups 1, 2, and 4; Days 8 and 22 for Group 3	±1 day
Fourteen days post-vaccination (prime)	Day 15 for Groups 1, 2, and 4	±2 days
Twenty-eight days post-vaccination (prime)	Day 29 for Groups 1, 2, and 4	±2 days
Fourteen days post-vaccination (boost)	Day 71 for Groups 1, 2, and 4; Day 29 for Group 3	±2 days
Twenty-one days post-vaccination (boost)	Day 78 for Groups 1, 2, and 4; Day 36 for Group 3	±2 days
Thirty-five days post-vaccination (boost)	Day 92 for Groups 1, 2, and 4; Day 50 for Group 3	±2 days
Seventy-seven days post-vaccination (boost)	Day 92 for Group 3	+10 days
Seven days post-vaccination (third vaccination)	Day 99 for subjects in Group 3 participating in the third vaccination	±1 day
Twenty-one days post-vaccination (third vaccination)	Day 113 for subjects in Group 3 participating in the third vaccination	±2 days
One hundred seventy-nine days post-vaccination (prime)	Day 180 for all Groups	±10 days
Two hundred thirty-nine days post-vaccination (prime)	Day 240 for all Groups	±15 days
Three hundred fifty-nine days post-vaccination (prime)	Day 360 for all Groups	±15 days

a See Section 10.3 for timing in case of events constituting a contraindication to vaccination.

9.1.3. Screening

Up to 35 days before the baseline visit (Day 1; day of prime vaccination), screening assessments, with the exception of laboratory screening assessments, will occur (see Time and Events Schedule); laboratory screening assessments must be performed within 28 days before Day 1. Screening may be split into multiple days or visits.

The ICF will be signed before any study-specific procedures at the start of the screening period (see Section 16.2.3). For men and for women of non-childbearing potential (defined in Inclusion Criterion #7), there will be no minimum duration of the screening period, and it will last only for the time required to verify eligibility criteria. For women of childbearing potential, a negative serum β-hCG pregnancy test before Day 1 and a negative urine test immediately prior to each study vaccination should be obtained to confirm that the subjects are not pregnant.

Only healthy subjects complying with the criteria specified in Section 4 will be included in the study. The investigator will provide detailed information on the study to the subject and will

obtain written informed consent prior to each subject's study participation. The procedures indicated in the Time and Events Schedule will only be performed after the subject's written informed consent has been obtained.

The following is performed to determine the eligibility requirements, as specified in the inclusion and exclusion criteria:

- Review of all inclusion and exclusion criteria.
- Review of medical history and demographics.
- Review of prestudy therapies.
- Serum pregnancy test (for women of childbearing potential).
- Blood sampling for hematology and chemistry (fasting or non-fasting state).
- Urine sampling for urinalysis.
- Serology testing (HIV type 1 or type 2, hepatitis B virus, hepatitis C virus).
- FSH assessment (if necessary for confirmation of post-menopausal state; see Inclusion Criterion #7).
- Urine drug screen.
- Abbreviated physical examination, excluding genito-urinary examination.
- Measurement of vital signs (heart rate and blood pressure, oral body temperature).
- Twelve-lead ECG.

The overall eligibility of the subject to participate in the study will be assessed once all screening values and results of any other required evaluations are available. Retesting of values (eg, safety laboratory) that lead to exclusion is allowed once using an unscheduled visit during the screening period to assess eligibility, provided there is an alternative explanation for the out-of-range value. Study subjects who qualify for inclusion will be contacted and scheduled for enrollment and prime vaccination within 28 days of collection of screening blood safety samples. Women of childbearing potential qualifying for inclusion will be contacted and scheduled for enrollment and prime vaccination at least 28 days after confirmation of practicing an effective method of birth control and within 28 days of collection of screening blood safety samples.

If the screening blood safety sample was taken more than 28 days prior to the scheduled day of prime immunization, it should be repeated prior to Day 1, and the subject can be included only after availability of results and confirmation that the subject fulfills Inclusion Criterion #5 and not Exclusion Criterion #11.

9.1.4. Vaccination

Prime Vaccination – Day 1

The investigator should ensure that all enrollment criteria have been met during screening. If a subject's status (including any unscheduled laboratory results or the receipt of additional medical records) after screening, but before the prime vaccination, changes such that the subject no longer meets all enrollment criteria, then the subject should be excluded from participation in the study.

Following a re-check of the inclusion and exclusion criteria, a urine pregnancy test (for women of childbearing potential), a physical examination, and measurements of vital signs, eligible subjects will be allocated to a vaccination schedule (see Section 5), and receive treatment as described in Section 6 unless any of the pre-specified criteria not to proceed with vaccination are met (for details, see Sections 10.2, 10.3, and 11.7).

Subjects who have a diastolic blood pressure value ≥90 mm Hg or a systolic blood pressure value ≥140 mm Hg prior to prime vaccination can receive the vaccination if they meet the conditions specified in Exclusion Criterion #19.

Before the prime vaccination, samples for hematology, chemistry, and urinalysis will be collected (see Section 9.3 for details). All subjects will also have blood drawn for baseline safety samples and immunologic assays.

Study vaccine will be administered by the blinded study vaccine administrator (see Definition of Terms, and see Section 5 for details of blinding). After vaccination, subjects will remain at the site for observation and vital sign assessments for at least 60 minutes, or longer if deemed necessary by the investigator. Reactogenicity and vital signs will be assessed at 30 (±5) minutes, and vital signs will be checked again at 60 (±5) minutes. If an event occurs during the 60-minute observation period, the highest overall grade of the event across the entire 60 minutes will be reported.

Upon discharge from the site, subjects will be provided with a diary, a ruler, and a thermometer to measure and record unsolicited and solicited local AEs, systemic events, and oral body temperature. Subjects will also record symptoms of unsolicited and solicited local and systemic AEs in the diary in the evening after each vaccination and then daily for the next 7 days.

Subjects will be instructed to contact the investigator immediately if they experience any AE about which they are concerned. Subjects in the sentinel cohorts will be contacted approximately 24 hours after study vaccination to confirm that no pre-specified pausing rules have been met. All AEs and SAEs will be collected and documented on the CRFs, together with the information on any concomitant medications.

Boost Vaccination – Day 57 in Groups 1, 2, and 4; Day 15 in Group 3

Subjects will receive the boost vaccination, unless any of the pre-specified criteria not to proceed with vaccination are met (see Sections 10.2, 10.3, and 11.7).

A urine pregnancy test (women of childbearing potential), a physical examination, and vital signs measurements will be performed before study vaccine administration. Pre-vaccination samples for hematology, chemistry, and urinalysis will also be collected (see Section 9.3 for details). In menstruating women, urinalysis will be postponed to the next visit. All subjects will also have blood drawn for immunologic assays.

Study vaccine will be administered by the blinded study vaccine administrator (see Definition of Terms, and see Section 5 for details of blinding). After vaccination, subjects will remain at the site for observation and vital sign assessments for at least 60 minutes, or longer if deemed necessary by the investigator. Reactogenicity and vital signs will be assessed at 30 (±5) minutes, and vital signs will be checked again at 60 (±5) minutes. If an event occurs during the 60-minute observation period, the highest overall grade of the event across the entire 60 minutes will be reported.

Upon discharge from the site, procedures will be identical to those following discharge after prime vaccination.

Boost Vaccination (Third Vaccination) - Day 92 in Subset of Group 3

The first 8 subjects in Group 3 who are willing to enroll for the third vaccination, will receive a third vaccination at Day 92. In an observer-blind manner, subjects who previously received placebo will receive placebo a third time and subjects who previously received MVA-BN-Filo/Ad26.Filo vaccination will receive Ad26.Filo at the dose of $9x10^{10}$ vp as third vaccination. After enrollment of the 8 subjects, the unblinded monitor and unblinded pharmacist will assess whether 7 subjects who previously received MVA-BN-Filo/Ad26.Filo vaccination have been enrolled. If less than 7 subjects of the active vaccine regimen have been enrolled, 2 additional subjects will be enrolled. If at least 7 subjects of the active vaccine regimen have been enrolled, no further subjects will be enrolled. The aim is to enroll 7 or 8 subjects who will receive Ad26.Filo as third vaccination.

Subjects will receive the third vaccination, unless any of the pre-specified criteria not to proceed with vaccination are met (see Sections 10.2 and 10.3).

A urine pregnancy test (women of childbearing potential), a physical examination, and vital signs measurements will be performed before study vaccine administration. Pre-vaccination samples for hematology, chemistry, and urinalysis will also be collected (see Section 9.3 for details). In menstruating women, urinalysis will be postponed to the next visit. All subjects will also have blood drawn for immunologic assays.

Study vaccine will be administered by the blinded study vaccine administrator (see Definition of Terms, and see Section 5 for details of blinding). After vaccination, subjects will remain at the site for observation and vital sign assessments for at least 30 minutes, or longer if deemed necessary by the investigator. Reactogenicity and vital signs will be assessed at 30 (±5) minutes. If an event occurs during the 30-minute observation period, the highest overall grade of the event across the entire 30 minutes will be reported.

Upon discharge from the site, procedures will be identical to those following discharge after prime and boost vaccination.

9.1.5. Post-vaccination

In Groups 1, 2, and 4, subjects will come to the clinic at 3, 7, 14, and 28 days after prime vaccination and 3, 7, 14, 21, and 35 days after boost vaccination, as described in the Time and Events Schedule. All subjects in Group 3 will come to the clinic at 3 and 7 days after prime vaccination and at 3, 7, 14, 21, 35, and 77 days after boost vaccination. Subjects in Group 3 who are participating in the third vaccination will also come to the clinic at 7 and 21 days after the third vaccination. All subjects will also come to the clinic at 179, 239, and 359 days after prime vaccination.

End-of-study assessments will be performed on the Day 360 visit for all subjects except those who withdrew earlier (see Section 10.2).

For details of the specific assessments performed at each post-vaccination visit, refer to the Time and Events Schedule.

At all visits, when leaving the clinic, subjects will be instructed to contact the investigator immediately if they experience any AE about which they are concerned.

9.1.6. Early Withdrawal

In case of early withdrawal due to an AE, the investigator will collect all information relevant to the AE and safety of the subject, and will follow the subject until resolution of the AE or until reaching a clinically stable endpoint. If feasible, blood will be drawn for immunologic assays. Subjects who wish to withdraw consent will be offered an optional visit for safety follow-up (before the formal withdrawal of consent). The subject has the right to refuse.

See Section 10.4 for details.

9.2. Immunogenicity Evaluations

Venous blood samples will be collected for the determination of immune responses at the time points and in volumes as indicated in the Time and Events Schedule.

Immunologic assessments and their purposes are summarized in Table 3 and Table 4. The exploratory assay package may include, but might not be limited to, the listed assays. Sample collection and processing will be performed according to current versions of approved standard operating procedures.

Table 3: Summary of Immunogenicity Assays (Humoral [Serology])

Assay	Purpose
Secondary endpoint assays	
ELISA	Analysis of antibodies binding to EBOV, MARV, and SUDV GPs
Exploratory endpoint assays	
Virus neutralization assays	Analysis of neutralizing antibodies against EBOV, MARV, and SUDV GP
Ad and/or MVA ELISA and/or neutralization assays	Binding and/or neutralizing antibodies against Ad and/or MVA
Molecular antibody characterization	Analysis of anti-EBOV GP, anti-MARV GP, and anti-SUDV GP antibody characteristics, including IgG subtyping

Ad = adenoviral vector; EBOV = Ebola virus; ELISA = enzyme-linked immunosorbent assay; GP = glycoprotein; IgG = immunoglobulin G; MARV = Marburg virus; MVA = Modified Vaccinia Ankara; SUDV = Sudan virus.

Table 4: Summary of Immunologic Assays (Cellular)

Assay	Purpose
Exploratory endpoint assays	
ELISpot	T cell IFN-γ responses to EBOV, MARV, and SUDV GPs
Intracellular cytokine staining	Analysis of T cell responses to EBOV, MARV, and SUDV GPs (including CD4/8+, IL-2, IFN-γ, TNF-α, and/or activation markers)
Transcriptomics	Transcriptome analysis of immune cell populations

CD = cluster of differentiation; EBOV = Ebola virus; ELISpot = enzyme-linked immunospot; GP = glycoprotein; IFN = interferon; IL = interleukin; MARV = Marburg virus; SUDV = Sudan virus; TNF = tumor necrosis factor.

Future scientific research may be conducted using samples obtained in this study to further investigate study vaccines- and disease-related questions. This may include the development of new or the improvement of existing techniques to characterize EBOV-, MARV-, and/or SUDV-directed immune responses or diagnostic tests.

9.3. Safety Evaluations

See Section 11.6 for details regarding the SMC.

Any clinically relevant AEs that occur post-vaccination until 28 days post last dose must be recorded on the CRF (reactogenicity will be reported by the subject until 7 days after each study vaccine administration). Thereafter, recording will be limited to SAEs.

All Grade 1, 2, and 3 changes, regardless of clinical significance, must be assessed for relatedness to the study vaccination. The assessment of causality must be done by a licensed study physician (the investigator or designee).

Any clinically significant abnormalities persisting at the end of the study or upon early withdrawal will be followed by the investigator until resolution or until a clinically stable endpoint is reached.

The study will include the following evaluations of safety and tolerability according to the time points provided in the Time and Events Schedule:

AEs

Adverse events will be collected as described in Section 12.3.1 and will be followed by the investigator as described in Section 12.

For solicited AEs, the following applies:

Solicited AEs

Information related to solicited AEs, as defined in Section 12.1.1, will be recorded by subjects in a diary. All subjects will be provided with a diary and instructions on how to complete the diary (Section 9.1.1). The investigator or the designee should discuss the information from the diary with the subject and document the relevant information in the clinic chart. There will be a 30-minute post-vaccination assessment of solicited AEs at the site. Solicited AEs will be captured on a separate CRF page as described in the CRF Completion Guidelines.

Injection Site (Local) AEs

Subjects will be asked to note in the diary occurrences of pain/tenderness, erythema/redness, induration/swelling, and itching, and/or warmth at the injection site daily until 7 days after each administration of study vaccine. These occurrences will be recorded in the diary provided to serve as a reminder to the subject for the next visit. The extent (largest diameter) of any erythema/redness and induration/swelling should be measured (using the ruler supplied), and the highest severity of any solicited AE should be recorded daily.

- **Injection Site Pain/Tenderness**

  Injection site pain (eg, stinging, burning) is an unpleasant sensory and emotional experience associated with actual or potential tissue damage and occurring at the immunization site (with or without involvement of surrounding tissue). Injection site tenderness is a painful sensation localized at the injection site upon palpation and/or movement of the limb. Because of the subjective nature of the reaction, the severity assessment of pain/tenderness is self-reported (if a subject is unable to provide self-report, other reporters include parent/caregiver or healthcare provider).[11]

- **Injection Site Erythema/Redness**

  Injection site erythema is a redness of the skin caused by dilatation and congestion of the capillaries localized at the injection site. It can best be described by looking and measuring. It will be evaluated through self-assessment by the subject. As the subject may not understand the term 'erythema', the synonymous term 'redness' is being used in the diary.

- **Injection Site Swelling/Induration**

  Injection site swelling is a visible, localized enlargement of an injected limb. It may be either soft (typically) or firm (less typical). Injection site induration is a palpable thickening, firmness, or hardening of soft tissue, usually has well-demarcated palpable borders, can be visible (raised or sunken compared with surrounding skin), is often ‘woody’ to touch and

has a flat shape. As differentiation between swelling and induration may be difficult without healthcare professional's assessment, both symptoms have been combined to allow self-assessment by the subjects. Both swelling and induration can best be described by looking and measuring.

Note: any other injection site events not meeting the above case definitions should be reported separately as unsolicited AEs.[18,19]

Systemic AEs

Subjects will be instructed on how to record daily oral body temperature using a thermometer provided for home use. Oral body temperature should be measured at approximately the same time each day. Subjects should record the oral body temperature in the evening post-vaccination, and then daily for the next 7 days in the diary, preferably in the evening. Additionally, a temperature assessment taken in the morning, either at home or in the clinic, on the day 7 post-vaccination visit may be recorded in the diary. If more than 1 measurement is made on any given day, the highest temperature of that day should be recorded in the CRF.

Fever is defined as endogenous elevation of body temperature ≥38°C, as recorded in at least 1 measurement.[20]

If a solicited local or systemic AE is not resolved at Day 8, the follow-up will be captured in the diary. The subject will be instructed to record the date of last symptoms and maximum severity in the diary after resolution.

Subjects will also be instructed on how to note daily in the diary for 7 days post-vaccination the following events:

- fatigue
- headache
- myalgia
- arthralgia
- chills
- nausea
- vomiting
- rash
- general itching

If a solicited systemic AE is reported more than once on any given day, the highest severity of that day should be recorded.

The severity of these solicited systemic AEs will be graded according to the criteria presented in Section 12.1.3.

Clinical Laboratory Tests

Samples will be collected for hematology, serum chemistry, and urinalysis.

The investigator must review the laboratory results, document this review, and record any laboratory abnormalities that represent an increase in toxicity grade (Grade 1, 2, or 3) after study vaccination as an AE and a causality must be assigned. The laboratory reports must be filed with the source documents.

All AEs should be followed closely to resolution, or until reaching a clinically stable endpoint, according to a sampling schedule that is medically appropriate for the individual parameters.

The following tests will be performed by the central laboratory at the time points indicated in the Time and Events Schedule, unless otherwise specified. Parameters marked with an asterisk (*) will only be measured at screening.

- Hematology and coagulation panel:

-hemoglobin	-prothrombin time
-WBC count with differential	-activated partial thromboplastin time
-red blood cell (RBC) count, parameters, and morphology	
-platelet count	

A WBC evaluation may include any abnormal cells, which will then be reported by the laboratory. An RBC evaluation may include abnormalities in the RBC count and/or RBC parameters and/or RBC morphology, which will then be reported by the laboratory. In addition, any other abnormal cells in a blood smear will also be reported. Only clinically significant abnormal WBCs, abnormal RBCs, or any other abnormal cells in a blood smear will be reported as an AE.

- Serum chemistry panel:

-sodium*	-albumin*
-potassium*	-total protein*
-blood urea nitrogen	-total bilirubin*
-AST	-creatinine
-ALT	-FSH* (if necessary for confirmation of post-menopausal state; see Inclusion Criterion #7)
-glucose (fasting or non-fasting)*	

- Urinalysis - dipstick:

-glucose	-protein
	-blood

Microscopic examination will be performed in the event of positive urinalysis dipstick tests. In the microscopic examination, observations other than the presence of WBCs, RBCs, and casts may also be reported by the laboratory and may need to be reported as AEs if considered clinically significant.

Laboratory values will be determined according to a toxicity grading scale adapted from the FDA Toxicity Grading Scale for Healthy Adult and Adolescent Volunteers Enrolled in Preventive Vaccine Clinical Trials included in Attachment 1. Grading will be adjusted to laboratory normal range if needed.

Additional clinical laboratory assessments to be performed are as follows:

- Serum pregnancy test for women of childbearing potential at screening.
- Urine pregnancy test for women of childbearing potential before each study vaccination.
- Serology (HIV type 1 and type 2, hepatitis B virus, hepatitis C virus) at screening.
- Urine drug screen at screening.

ECG

A single, 12-lead ECG will be performed at screening and will be read locally.

Electrocardiograms, with or without troponin I assessment, will only be repeated during the study if clinically indicated based on signs and symptoms (per the investigator's judgment).

During the collection of ECGs, subjects should be in supine position in a quiet setting without distractions (eg, television, cell phones). Subjects should rest (while sitting comfortably) for at least 5 minutes before ECG collection and should refrain from talking or moving arms or legs. If blood sampling or vital signs measurement is scheduled for the same time point as ECG recording, the procedures should be performed in the following order: vital signs, ECG(s), blood draw.

Vital Signs

Vital signs measurements (heart rate, blood pressure, oral body temperature) will be performed in supine position at screening and before and at 30 (±5) and 60 (±5) minutes after each study vaccine administration, except for the subset of Group 3 where vital signs measurements will be performed before the third vaccination and at 30 (±5) minutes after the third vaccination, as indicated in the Time and Events Schedule. Blood pressure and heart rate measurements should be preceded by at least 5 minutes of rest in supine position and in a quiet setting without distractions (eg, television, cell phones). Confirmatory vital signs measurements can be performed if inconsistent with a prior measurement.

Vital signs toxicity grading will be done according to a toxicity grading scale adapted from the FDA Toxicity Grading Scale for Healthy Adult and Adolescent Volunteers Enrolled in Preventive Vaccine Clinical Trials included in Attachment 1. Any abnormal finding that represents an increase in toxicity grade (from baseline to Grade 1, 2, or 3) after study vaccination must be recorded. All events should be followed to resolution, or until reaching a clinically stable endpoint.

Physical Examination

An abbreviated physical examination, including height and body weight but excluding genito-urinary examination, will be performed at screening. At other visits, an abbreviated, symptom-directed physical examination will be performed as indicated by the investigator based on any clinically relevant issues, clinically relevant symptoms, and medical history. The symptom-directed physical examination may be repeated if deemed necessary by the investigator.

Physical examinations will be performed by the investigator or by the designated medically trained clinician.

All events should be followed to resolution, or until reaching a clinically stable endpoint.

9.4. Vaccine-induced Seropositivity

In general, uninfected subjects who participate in filovirus vaccine studies may develop filovirus-specific antibodies as a result of an immune response to the candidate filovirus vaccine, referred to as vaccine-induced seropositivity. These antibodies may be detected in filovirus serologic tests, causing the test to appear positive even in the absence of actual filovirus infection. Vaccine-induced seropositivity may become evident during the study, or after the study has been completed.

Subjects should not donate a unit of blood within 8 weeks before Day 1 or plan to donate blood during participation in the study (from the start of screening onwards; Exclusion Criterion #25).

Consent will be obtained to contact the doctors that are regularly consulted by the subject, to let them know that the subject is taking part in this study. It is important for all of the subject's doctors to know that the subject may be taking investigational vaccines. When the study is unblinded, the subject will be contacted and informed about which vaccine (active or placebo) the subject had received.

9.5. Sample Collection and Handling

The actual dates and times of sample collection must be recorded in the CRF or laboratory requisition form. Refer to the Time and Events Schedule for the timing and frequency of all sample collections.

Instructions for the collection, handling, storage, and shipment of immunogenicity samples will be provided in the Laboratory Manual. Collection, handling, storage, and shipment of samples must be under the specified, and where applicable, controlled temperature conditions as indicated in the Laboratory Manual.

10. SUBJECT COMPLETION/DISCONTINUATION OF STUDY TREATMENT/ WITHDRAWAL FROM THE STUDY

10.1. Completion

A subject will be considered to have completed the study if he or she has completed all end-of-study assessments on Day 360.

10.2. Discontinuation of Study Vaccine/Withdrawal From the Study

Discontinuation of Study Vaccine

A subject will not be automatically withdrawn from the study if he or she has to discontinue study vaccine administration.

Subjects will be discontinued from study vaccine administration for the reasons listed below. These subjects must not receive any further study vaccine, but should continue to be monitored for safety and for immunogenicity if this does not result in a safety risk for the subject.

- Any reason listed in Section 11.7 for preventing a subject from receiving boost vaccination or third vaccination.
- Pregnancy.
- Any AE considered at least possibly related to study vaccine, worsening of health status, or intercurrent illnesses that, in the opinion of the investigator, requires discontinuation from study vaccine.
- Confirmed filovirus disease.
- Intake of disallowed medications (see Section 8).

Withdrawal From the Study

Each subject has the right to withdraw at any time for whatever reason without affecting the right to treatment by the investigator. The investigator should make an attempt to contact subjects who did not return for scheduled visits or follow-up. Although the subject is not obliged to give reason(s) for withdrawing early, the investigator should make a reasonable effort to ascertain the reason(s) while fully respecting the subject's rights.

A subject will be withdrawn from the study for any of the following reasons:

- Repeated failure to comply with protocol requirements.
- Decision by the sponsor to stop or cancel the study.
- Decision by the investigator to withdraw subjects.
- Decision by local regulatory authorities and Institutional Review Board (IRB)/Independent Ethics Committee (IEC) to stop or cancel the study.
- Lost to follow-up.
- Withdrawal of consent.

- Death.

If a subject is lost to follow-up, every reasonable effort must be made by the study-site personnel to contact the subject and determine the reason for discontinuation/withdrawal. The measures taken to follow up must be documented.

Subjects who withdraw after randomization but before the prime vaccination will be replaced. Enrollment will be stopped when 18 subjects each in Groups 1 to 4 have received at least 1 study vaccination.

Subjects who withdraw after receiving the prime vaccination will not be replaced. If a subject withdraws early from the study, early withdrawal assessments should be obtained (see Section 9.1.6).

When a subject withdraws before completing the study, the reason for withdrawal is to be documented in the CRF and in the source document. Study vaccine assigned to the withdrawn subject may not be assigned to another subject.

A subject who wishes to withdraw consent from participation in the study will be offered an optional visit for safety follow-up (before formal withdrawal of consent). The subject has the right to refuse.

10.3. Contraindications to Vaccination

The following events constitute a contraindication to vaccination at that point in time.

- Acute illness at the time of vaccination (this does not include minor illnesses such as diarrhea or mild upper respiratory tract infection).
- Fever (oral temperature ≥38.0°C) at the time of vaccination.

If any of these events occur at the scheduled time for vaccination:

- For the prime vaccination: The subject can be rescheduled for any later date. If the rescheduling results in prime dose being administered beyond the timing of screening procedures (28 days for laboratory screening assessments and 35 days for all other screening procedures), screening tests must be repeated as appropriate.

- For any boost vaccination, including the third vaccination: The subject may be vaccinated up to 10 days beyond the window allowed for the scheduled vaccination, or will be discontinued from that vaccination at the discretion of the investigator and after consultation with the sponsor:

Note: In case the boost vaccination or the third vaccination is postponed, the timing of the safety/immunogenicity visits post-boost will be planned relative to the actual vaccination day.

Note that medically indicated vaccines should be administered at least 15 days before or 15 days after study vaccine administration (see Section 8).

Other contraindications to vaccination are provided in Section 11.7.

10.4. Withdrawal From the Use of Research Samples

A subject who withdraws from the study will have the following options for storage of samples for future use:

- The collected samples will be retained and used in accordance with the subject's original informed consent for storage of samples for future use.
- The subject may withdraw consent for storage of samples for future use, in which case the samples will be destroyed, and no further testing will take place. To initiate the sample destruction process, the investigator must notify the sponsor study-site contact about withdrawal of consent for the storage of samples for future use and request sample destruction. If requested, the investigator will receive written confirmation from the sponsor that the samples have been destroyed.

Withdrawal From Storage of Samples for Future Use While Remaining in the Study

The subject may withdraw consent for storage of samples for future use (see Section 16.2.5) while remaining in the study. In such a case, the samples will be destroyed as described above. Details of the sample retention for research are presented in the main ICF.

11. STATISTICAL METHODS

Statistical analysis will be done by the sponsor or under the authority of the sponsor. A general description of the statistical methods to be used to analyze the immunogenicity and safety data is outlined below. Specific details will be provided in the Statistical Analysis Plan.

A primary analysis will be performed when all subjects have completed the 21-day post last dose visit (Day 78 in Groups 1, 2, and 4, Day 36 in Group 3, and Day 113 for a subset of Group 3) or withdrew earlier. This analysis will include all available data up to this point.

The final analysis will be performed when all subjects from Groups 1 to 4 have completed the last study visit or withdrew earlier.

Additional interim analyses may be performed at the sponsor's discretion.

11.1. Analysis Sets

The <u>Full Analysis (FA)</u> set includes all subjects who were randomized and received at least 1 dose of study vaccine, regardless of the occurrence of protocol deviations. Safety data will be analyzed based on the FA set.

The <u>Immunogenicity Response (IR)</u> analysis set includes all randomized and vaccinated subjects, who have data from baseline and at least 1 post-vaccination immunogenicity blood draw.

11.2. Sample Size Determination

The primary outcome of the study will be safety. The sample size for this study is not based on formal hypothesis testing considerations, but is within the range of subjects recommended in the

Code of Federal Regulations (CFR) 312.21 for the first-in-human products in this investigation. In each of the 4 groups, 15 subjects will receive active vaccine and 3 subjects will receive placebo. Placebo recipients are included for blinding purposes and safety analyses, and will provide control specimens for immunologic assays. Group 4 was included to serve as a tolerability and immunogenicity control arm.

The sample size for this study will provide a preliminary safety and immunogenicity assessment. While mild to moderate vaccine reactions (local and systemic responses) are expected, AEs that preclude further study vaccine administration or more serious events that would limit product development are not anticipated. With 15 subjects receiving active vaccines per group, the observation of 0 such reactions would be associated with a 97.5% 1-sided confidence upper limit that the true rate is <22%.

Table 5 shows the probabilities of observing ≥1 AE at given true AE rates.

Table 5: Probability of Observing ≥1 AE Given a True AE Incidence

	Probability of Observing ≥1 AE (%)		
**True AE Incidence**	**n_1=15**	**n_2=30**	**N=45**
**1%**	14	26	36
**2.5%**	32	53	68
**5%**	54	79	90
**10%**	79	96	99
**20%**	96	100	100

AE = adverse event.

For Ad26.Filo: n_1: number of subjects receiving Ad26.Filo as prime; n_2: number of subjects receiving Ad26.Filo as boost; N: number of subjects receiving Ad26.Filo as either prime or boost.

For MVA-BN-Filo: n_1: number of subjects receiving MVA-BN-Filo as boost; n_2: number of subjects receiving MVA-BN-Filo as prime; N: number of subjects receiving MVA-BN-Filo as either prime or boost.

11.3. Subject Information

For all subjects, demographic characteristics (eg, age, height, weight, BMI, race, and sex) and screening/baseline characteristics (eg, physical examination and medical history) will be tabulated and summarized with descriptive statistics.

11.4. Immunogenicity Analyses

No formal hypothesis on immunogenicity will be tested. The analysis of immunogenicity will be done on the IR analysis set. See Section 11.1 for definitions of the analysis sets.

Descriptive statistics (actual values and changes from references) will be calculated for continuous immunologic parameters at all time points. Graphical representations of changes in immunologic parameters will be prepared, as applicable. Frequency tabulations will be calculated for discrete (qualitative) immunologic parameters at all time points.

In addition, for the immunologic parameters, the response patterns over time will be analyzed, taking into account within-subject correlations. More detailed descriptions will be provided in the Statistical Analysis Plan.

Similar summaries will be presented for the subjects in Group 3 who will receive a third vaccination for the time points after this last vaccination.

11.5. Safety Analyses

No formal statistical testing of safety data is planned. Safety data will be analyzed descriptively (including 95% confidence intervals, if applicable).

Baseline for all safety parameters will be defined as the last value before the prime vaccination.

For the subjects in Group 3 who will receive a third vaccination, similar safety summaries will be provided. Details will be provided in the statistical analysis plan.

AEs (Including Reactogenicity)

The verbatim terms used in the CRF by investigators to identify AEs will be coded using the Medical Dictionary for Regulatory Activities. All reported AEs (solicited local at injection site, solicited systemic, and unsolicited) with onset after each vaccination (ie, treatment-emergent AEs and AEs that have worsened since baseline) will be included in the analysis. For each AE, the number of subjects who experience at least 1 occurrence of the given event will be summarized by vaccine group and presented separately for solicited and unsolicited AEs. Summaries, listings, datasets, and/or subject narratives may be provided as appropriate for those subjects who die, discontinue study vaccinations due to an AE, or experience a severe AE, SAE, or IREs. The analysis of solicited AEs will be performed on those subjects in the FA set for whom reactogenicity assessments are available in the database. The analysis of unsolicited AEs will be done based on the FA set.

Solicited local (at injection site) and systemic AEs will be summarized descriptively. The overall frequencies per vaccine group as well as frequencies according to severity and duration will be calculated for solicited AEs. In addition, the number and percentages of subjects with at least 1 solicited local (at injection site) or systemic AE will be presented. Frequencies of solicited and unsolicited AEs, separately for all and vaccination-related only, will be presented by system organ class and preferred term.

Summaries and listings may be provided separately for AEs that were reported pre-dose at the moment of subsequent vaccinations.

Additional safety analyses other than those listed above may be provided depending on the review of the clinical database.

Clinical Laboratory Tests

Laboratory data will be summarized by type of laboratory test. Graphical presentation of changes in laboratory tests will be made, as applicable. If the baseline value is not available, the value at screening will be used as baseline value. Toxicity grade for laboratory abnormalities will be determined according to a toxicity grading scale adapted from the FDA Toxicity Grading Scale for Healthy Adult and Adolescent Volunteers Enrolled in Preventive Vaccine Clinical Trials

included in Attachment 1. Grading will be adjusted to laboratory normal range if needed. Laboratory abnormalities will be tabulated per treatment group and scheduled time point.

Vital Signs

For heart rate, blood pressure (systolic, diastolic) values, and oral body temperature, the percentage of subjects with values beyond pre-specified limits will be summarized.

Physical Examination

Physical examination findings and changes from baseline will be summarized at each scheduled time point. Abnormalities will be listed. BMI will be calculated using the recording of height at screening. Body weight and BMI results will be tabulated and summarized descriptively.

11.6. SMC

An independent SMC will be appointed by the DMID before the start of the study. The SMC will consist of at least 3 members, of which at least 1 is a medical expert in the relevant therapeutic area. The SMC responsibilities, authorities, and procedures will be documented in its charter. An SMC meeting will be called by the DMID if any pausing rule(s) is/are met (see Section 11.7 for details).

The SMC will receive unblinded data and will review all data that are deemed necessary to evaluate the AE(s), SAE(s), and/or laboratory abnormality/ies that triggered the meeting(s) and will make a recommendation to the DMID if the study should proceed as planned, be modified, or be terminated. The DMID clinical project manager and medical monitor will review the SMC recommendation(s) and report them to the sponsor. The sponsor will be responsible for responding to the SMC and DMID regarding any actions that were recommended but that will not be implemented.

If a pausing rule is met, further vaccinations within the same vaccine group will be halted and further vaccinations may be halted in the other groups (except for Group 4) until SMC review is complete. Resumption of vaccination may be recommended by the SMC following review of the available cumulative safety data as outlined in the charter. The site will be allowed to resume activities upon receipt of a written notification from the sponsor. As applicable, the appropriate regulatory authorities will be informed in writing of the recommendation(s) by the SMC and the decision by the sponsor to resume, modify, or discontinue study activities. The site is responsible for notifying their IEC/IRB according to local standards and regulations.

An ad hoc SMC meeting may be called for reasons other than meeting pausing rules, including decision to proceed or not to proceed with boost dose administration in individual subjects (see Section 11.7.6).

The SMC will have a final review meeting 6 to 8 months after clinical database lock to review the cumulative unblinded safety data for the study. The data will be provided in a standard summary format. The SMC may be asked to provide recommendations in response to questions posed by the DMID.

11.7. Pausing Rules

The principal investigator and the designated co-investigators will review the safety of enrolled subjects on an ongoing basis. The sponsor's medical monitor will be involved in all discussions and decisions. In Groups 1 and 2, there is a different set of pausing rules for the sentinel cohorts and for the post-sentinel cohorts. If no pausing rule is met in the sentinel cohort of a given group by Day 4, recruitment will open to the remaining subjects of that group.

11.7.1. Pausing Rules for Prime Dose in Sentinel Cohorts of Groups 1 and 2

Administration of Ad26.Filo $9x10^{10}$ vp in Group 1 and of MVA-BN-Filo $5x10^{8}$ Inf U in Group 2 will start with the 4 subjects (3 on active/1 on placebo) in the sentinel cohorts. Prior to opening recruitment to the remaining subjects in these 2 groups, further vaccinations will be withheld, and an SMC meeting will be called if, in the sentinel cohort, within the first 72 hours post-vaccination:

1. One or more subjects experience any SAE other than the result from trauma or accident (clearly not attributable to study vaccine), regardless of relatedness to study product; *OR*
2. One or more subjects experience anaphylaxis or generalized urticaria within 24 hours after the prime vaccination; *OR*
3. One or more subjects experience a severe (≥Grade 3) unsolicited AE that is considered to be related to the study vaccine; *OR*
4. One or more subjects experience a severe (≥Grade 3) solicited injection site reaction lasting for ≥72 hours. (The size [measured in mm] of erythema/redness and the occurrence of induration/swelling will not be used as a pausing criterion.) *Note: If a severe (≥Grade 3) solicited injection site reaction is ongoing at Visit 2 for <72 hours, the investigator will schedule telephone calls or extra visits on the following day(s) to assess the evolution of the event and its duration*; *OR*
5. One or more subjects experience a severe (≥Grade 3) solicited systemic AE lasting for ≥72 hours (subjective systemic reaction corroborated by study personnel). *Note: If a severe (≥Grade 3) solicited systemic AE is ongoing at Visit 2 for <72 hours, the investigator will schedule telephone calls or extra visits on the following day(s) to assess the evolution of the event and its duration*; *OR*
6. Death of a subject.

The above pausing rules are intended to be group-specific, ie, occurrence of a pausing rule in Group 1 will trigger withholding of vaccination in Group 1 only and not in Group 2, and vice versa.

11.7.2. Pausing Rules for Prime Dose in Post-sentinel Cohorts of Groups 1 and 2

Occurrence of any of the following events will lead to pause of further study vaccination, and will trigger a meeting of the SMC to discuss study pause or discontinuation of further vaccination:

1. One or more subjects experience any SAE other than the result from trauma or accident (clearly not attributable to study vaccine), regardless of relatedness to study product; *OR*
2. One or more subjects experience anaphylaxis within 24 hours, or generalized urticaria within 72 hours after the prime vaccination; *OR*
3. Two or more subjects (including case[s] occurring in the sentinel cohorts) experience a severe (≥Grade 3) unsolicited AE of the same type that is considered to be related to any of the study vaccines. <u>*Note*</u>*: If SMC recommends to resume vaccination after pause for this provision, occurrence of the same severe (≥Grade 3) AE in additional subject(s) will not necessarily result in study pause, but the recommendation to withhold vaccination or not will be made by the SMC on a case-by-case basis*; *OR*
4. Two or more subjects (including case[s] occurring in the sentinel cohorts) experience a severe (≥Grade 3) laboratory abnormality within the same preferred term (including unexplained hematuria) considered to be related to any of the study vaccines. <u>*Note*</u>*: If SMC recommends to resume vaccination after pause for this provision, occurrence of the same severe (≥Grade 3) laboratory abnormality in additional subject(s) will not necessarily result in study pause, but the recommendation to withhold vaccination or not will be made by the SMC on a case-by-case basis*; *OR*
5. Three or more subjects (including case[s] occurring in the sentinel cohorts) who received the prime dose of study vaccine experience the same severe (≥Grade 3) solicited injection site reaction that persists for ≥72 hours. (The size [measured in mm] of erythema/redness and the occurrence of induration/swelling will not be used as a pausing criterion). If a severe (≥Grade 3) solicited injection site reaction is ongoing at Visit 2 for <72 hours, the investigator will schedule telephone calls or extra visits on the following day(s) to assess the evolution of the event and its duration. <u>*Note*</u>*: If SMC recommends to resume vaccination after pause for this provision, occurrence of the same severe (≥Grade 3) solicited injection site reaction in additional subject(s) will not necessarily result in study pause, but the recommendation to withhold vaccination or not will be made by the SMC on a case-by-case basis*; *OR*
6. Three or more subjects (including case[s] occurring in the sentinel cohorts) who received the prime dose of study vaccine experience the same severe (≥Grade 3) solicited systemic AE that persists for ≥72 hours (subjective systemic reaction corroborated by study personnel). If a severe (≥Grade 3) solicited systemic AE is ongoing at Visit 2 for <72 hours, the investigator will schedule telephone calls or extra visits on the following day(s) to assess the evolution of the event and its duration. <u>*Note*</u>*: If SMC recommends to resume vaccination after pause for this provision, occurrence of the same severe (≥Grade 3) solicited systemic AE in additional subject(s) will not necessarily result in study pause, but the recommendation to withhold vaccination or not will be made by the SMC on a case-by-case basis*; *OR*
7. Death of a subject.

The above pausing rules are intended to be group-specific, ie, occurrence of a pausing rule in Group 1 will trigger withholding of vaccination in Group 1 only and not in Group 2, and vice versa.

11.7.3. Pausing Rules for Boost Dose in Sentinel Cohorts of Groups 1 and 2

Administration of the boost dose of MVA-BN-Filo $5x10^8$ Inf U in Group 1 and of the boost dose of Ad26.Filo $9x10^{10}$ vp in Group 2 will start with the 4 subjects in the sentinel cohorts (3 on active/1 on placebo) 56 days after the prime dose. Prior to administration of boost dose to the remaining subjects in these 2 groups, further vaccinations will be withheld, and an SMC meeting will be called if, in the sentinel cohort, within the first 72 hours post-vaccination:

1. One or more subjects experience any SAE other than the result from trauma or accident (clearly not attributable to study vaccine), regardless of relatedness to study product; *OR*
2. One or more subjects experience anaphylaxis or generalized urticaria within 24 hours after the boost vaccination; *OR*
3. One or more subjects experience a severe (≥Grade 3) unsolicited AE that is considered to be related to any of the study vaccines; *OR*
4. One or more subjects experience a severe (≥Grade 3) solicited injection site reaction that persists for ≥72 hours. (The size [measured in mm] of erythema/redness and the occurrence of induration/swelling will not be used as a pausing criterion.) *Note: If a severe (≥Grade 3) solicited injection site reaction is ongoing at Visit 7 for <72 hours, the investigator will schedule telephone calls or extra visits on the following day(s) to assess the evolution of the event and its duration; OR*
5. One or more subjects experience a severe (≥Grade 3) solicited systemic AE that persists for ≥72 hours (subjective systemic reaction corroborated by study personnel). *Note: If a severe (≥Grade 3) solicited systemic AE is ongoing at Visit 7 for <72 hours, the investigator will schedule telephone calls or extra visits the following day(s) to assess the evolution of the event and its duration; OR*
6. Death of a subject.

The above pausing rules are intended to be group-specific, ie, occurrence of a pausing rule in Group 1 will trigger withholding of vaccination in Group 1 only and not in Group 2, and vice versa.

11.7.4. Pausing Rules for Boost Dose in Post-sentinel Cohorts of Groups 1 and 2

Administration of the boost dose will be halted if:

1. One or more subjects receiving the study vaccine experience any SAE other than the result from trauma or accident (clearly not attributable to study vaccine), regardless of relatedness to study product; *OR*
2. One or more subjects experience anaphylaxis within 24 hours, or generalized urticaria within 72 hours after the boost vaccination; *OR*
3. Two or more subjects (including case[s] occurring in the sentinel cohorts) receiving the study vaccine experience a severe (≥Grade 3) unsolicited AE of the same type that is considered to be related to the study vaccines. *Note: If SMC recommends to resume vaccination after pause for this provision, occurrence of the same severe (≥Grade 3) AE in*

additional subject(s) will not necessarily result in study pause, but the recommendation to withhold vaccination or not will be made by the SMC on a case-by-case basis; *OR*

4. Two or more subjects (including case[s] occurring in the sentinel cohorts) experience a severe (≥Grade 3) laboratory abnormality within the same preferred term (including unexplained hematuria) considered to be related to any of the study vaccines. Note: *If SMC recommends to resume vaccination after pause for this provision, occurrence of the same severe (≥Grade 3) AE in additional subject(s) will not necessarily result in study pause, but the recommendation to withhold vaccination or not will be made by the SMC on a case-by-case basis*; *OR*
5. Three or more subjects (including case[s] occurring in the sentinel cohorts) receiving the study vaccine experience a severe (≥Grade 3) solicited injection site reaction lasting for ≥72 hours. (The size [measured in mm] of erythema/redness and the occurrence of induration/swelling will not be used as a pausing criterion). If a severe (≥Grade 3) solicited injection site reaction is ongoing at Visit 7 for <72 hours, the investigator will schedule telephone calls or extra visits on the following day(s) to assess the evolution of the event and its duration. Note: *If SMC recommends to resume vaccination after pause for this provision, occurrence of the same severe (≥Grade 3) solicited injection site reaction in additional subject(s) will not necessarily result in study pause, but the recommendation to withhold vaccination or not will be made by the SMC on a case-by-case basis*; *OR*
6. Three or more subjects (including case[s] occurring in the sentinel cohorts) receiving the study vaccine experience a severe (≥Grade 3) solicited systemic AE lasting for ≥72 hours (subjective systemic reaction corroborated by study personnel). If a severe (≥Grade 3) solicited systemic AE is ongoing at Visit 7 for <72 hours, the investigator will schedule telephone calls or extra visits on the following day(s) to assess the evolution of the event and its duration. Note: *If SMC recommends to resume vaccination after pause for this provision, occurrence of the same severe (≥Grade 3) solicited systemic AE in additional subject(s) will not necessarily result in study pause, but the recommendation to withhold vaccination or not will be made by the SMC on a case-by-case basis*; *OR*
7. Death of a subject.

The above pausing rules are intended to be group-specific, ie, occurrence of a pausing rule in Group 1 will trigger withholding of vaccination in Group 1 only and not in Group 2, and vice versa.

11.7.5. Pausing Rules for Prime and Boost Dose in Group 3

Study vaccine administration will be halted if:

1. One or more subjects receiving the study vaccine experience any SAE other than the result from trauma or accident (clearly not attributable to study vaccine), regardless of relatedness to study product; *OR*
2. One or more subjects receiving the study vaccine experience anaphylaxis or generalized urticaria within 24 hours after the prime or boost vaccination; *OR*
3. Two or more subjects receiving the study vaccine experience a severe (≥Grade 3) unsolicited AE of the same type that is considered to be related to any of the study vaccines; *OR*

4. Two or more subjects experience a severe (≥Grade 3) laboratory abnormality within the same preferred term (including unexplained hematuria) considered to be related to any of the study vaccines. Note: *If SMC recommends to resume vaccination after pause for this provision, occurrence of the same severe (≥Grade 3) laboratory abnormality in additional subject(s) will not necessarily result in study pause, but the recommendation to withhold vaccination or not will be made by the SMC on a case-by-case basis*; *OR*
5. Three or more subjects receiving the study vaccine experience a severe (≥Grade 3) solicited injection site reaction lasting for ≥72 hours. (The size [measured in mm] of erythema/redness and the occurrence of induration/swelling will not be used as a pausing criterion). If a severe (≥Grade 3) solicited injection site reaction is ongoing at Visits 2 or 5 for <72 hours, the investigator will schedule telephone calls or extra visits on the following day(s) to assess the evolution of the event and its duration. Note: *If SMC recommends to resume vaccination after pause for this provision, occurrence of the same severe (≥Grade 3) solicited injection site reaction in additional subject(s) will not necessarily result in study pause but the recommendation to withhold vaccination or not will be made by the SMC on a case-by-case basis*; *OR*
6. Three or more subjects receiving the study vaccine experience a severe (≥Grade 3) solicited systemic AE lasting for ≥72 hours (subjective systemic reaction corroborated by study personnel). If a severe (≥Grade 3) solicited systemic AE is ongoing at Visits 2 or 5 for <72 hours, the investigator will schedule telephone calls or extra visits on the following day(s) to assess the evolution of the event and its duration. Note: *If SMC recommends to resume vaccination after pause for this provision, occurrence of the same severe (≥Grade 3) solicited systemic AE in additional subject(s) will not necessarily result in study pause, but the recommendation to withhold vaccination or not will be made by the SMC on a case-by-case basis*; *OR*
7. Death of a subject.

There will not be any formal pausing rules for the subset of subjects in Group 3 who will receive a third vaccination.

11.7.6. Criteria for Discontinuation From Boost Administration to an Individual Subject

A subject will not be given any boost vaccination, including the third vaccination, if he/she experiences:

1. Anaphylaxis within 24 hours after administration of the prime or boost dose of a study vaccine; *OR*
2. Generalized urticaria within 72 hours after administration of the prime or boost dose of a study vaccine; *OR*
3. An SAE that is considered to be related to a study vaccine after prime or boost; *OR*
4. A severe (≥Grade 3) laboratory abnormality (including unexplained hematuria) that is considered to be related to a study vaccine after prime or boost; *OR*
5. A severe (≥Grade 3) unsolicited AE that is considered to be related to a study vaccine after prime or boost; *OR*

6. A severe (≥Grade 3) solicited injection site reaction with a duration of ≥72 hours after prime or boost; *OR*
7. A severe (≥Grade 3) solicited systemic AE with a duration of ≥72 hours considered to be related to a study vaccine after prime or boost.

Vaccinations for an individual subject may be suspended for safety concerns other than those described above at the discretion of the investigator if he/she feels the subject's safety may be threatened. The investigator may ask for an ad hoc SMC meeting to be held for any single event or combination of multiple events that, in his/her professional opinion, jeopardize the safety of the subjects or the reliability of the data.

Vaccinations for the study may be suspended for safety concerns other than those described above or before pausing rules are met if, in the judgment of investigator, subject safety may be threatened. The sponsor should be notified that the SMC will need to be convened.

12. ADVERSE EVENT REPORTING

Timely, accurate, and complete reporting and analysis of safety information from clinical studies are crucial for the protection of subjects, investigators, and the sponsor, and are mandated by regulatory agencies worldwide. The sponsor has established Standard Operating Procedures in conformity with regulatory requirements worldwide to ensure appropriate reporting of safety information; all clinical studies conducted by the sponsor or its affiliates will be conducted in accordance with those procedures.

12.1. Definitions

12.1.1. AE Definitions and Classifications

Method of Detecting AEs and SAEs

Care will be taken not to introduce bias when detecting AEs or SAEs. Open-ended and non-leading verbal questioning of the subject is the preferred method to inquire about AE occurrence. For some studies, subjects are not always able to provide valid verbal responses to open-ended questions. In these circumstances, another method of detecting these events is specified.

Solicited AEs

Solicited AEs are predefined local and systemic events for which the subject is specifically questioned and which are noted by subjects in their diary (see Section 9.1.1).

Unsolicited AEs

Unsolicited AEs are all AEs for which the subject is NOT specifically questioned in the subject diary.

AEs

An AE is any untoward medical occurrence in a clinical study subject administered a medicinal (investigational or non-investigational) product. An AE does not necessarily have a causal

relationship with the treatment. An AE can therefore be any unfavorable and unintended sign (including an abnormal finding), symptom, or disease temporally associated with the use of a medicinal (investigational or non-investigational) product, whether or not related to that medicinal (investigational or non-investigational) product. (Definition per International Council for Harmonisation [ICH]).

This includes any occurrence that is new in onset or aggravated in severity, toxicity grade, or frequency from the baseline condition, or abnormal results of diagnostic procedures, including laboratory test abnormalities.

Note: The sponsor collects AEs starting with the signing of the ICF (refer to Section 12.3.1 for time of last AE recording).

SAEs

An SAE based on ICH and European Union Guidelines on Pharmacovigilance for Medicinal Products for Human Use is any untoward medical occurrence that at any dose:

- Results in death.
- Is life threatening.
  (The subject was at risk of death at the time of the event. It does not refer to an event that hypothetically might have caused death if it were more severe).
- Requires inpatient hospitalization or prolongation of existing hospitalization.
- Results in persistent or significant disability/incapacity.
- Is a congenital anomaly/birth defect.
- Is a suspected transmission of any infectious agent via a medicinal product.
- Is medically important.*

*Medical and scientific judgment should be exercised in deciding whether expedited reporting is also appropriate in other situations, such as important medical events that may not be immediately life threatening or result in death or hospitalization but may jeopardize the subject or may require intervention to prevent one of the other outcomes listed in the definition above. These should usually be considered serious.

If a serious and unexpected AE occurs for which there is evidence suggesting a causal relationship between the study vaccine and the event (eg, death from anaphylaxis), the event must be reported as a serious and unexpected suspected adverse reaction (SUSAR) (even after the study is over, if the sponsor, SMC or investigator becomes aware of them) by the sponsor to the health authorities and by the investigator to the IEC/IRB according to regulatory and local requirements.

Unlisted (Unexpected) AEs/Reference Safety Information

An AE is considered unlisted if the nature or severity is not consistent with the applicable product reference safety information. For Ad26.Filo, Ad26.ZEBOV, and MVA-BN-Filo, the

expectedness of an AE will be determined by whether or not it is listed in the Investigator's Brochures and Addenda, if applicable.[14,15,16]

AEs Associated With the Use of the Study Vaccines

An AE is considered associated with the use of the study vaccine if the attribution is **"related"** by the definitions listed in Section 12.1.2.

An AE is considered not associated with the use of the study vaccine if the attribution is **"unrelated"** by the definitions listed in Section 12.1.2.

Immediate Reportable Events

The following list of neuroinflammatory disorders are categorized as IREs, and should be reported to the sponsor within 24 hours of becoming aware of the event using the IRE Form. Relevant data from the IRE Form will be captured in the clinical database.

- Cranial nerve disorders, including paralyses/paresis (eg, Bell's palsy).
- Optic neuritis.
- Multiple sclerosis.
- Transverse myelitis.
- Guillain-Barré syndrome, including Miller Fisher syndrome, Bickerstaff's encephalitis and other variants.
- Acute disseminated encephalomyelitis, including site specific variants: eg, non-infectious encephalitis, encephalomyelitis, myelitis, myeloradiculomyelitis.
- Myasthenia gravis and Lambert-Eaton myasthenic syndrome.
- Immune-mediated peripheral neuropathies and plexopathies, (including chronic inflammatory demyelinating polyneuropathy, multifocal motor neuropathy and polyneuropathies associated with monoclonal gammopathy).
- Narcolepsy.
- Isolated paresthesia of >7 days duration.

Symptoms, signs or conditions which might (or might not) represent the above diagnoses, should be recorded and reported as IREs even if the final or definitive diagnosis has not yet been determined, and alternative diagnoses have not yet been eliminated or shown to be less likely. Follow up information and final diagnoses, if applicable, should be submitted as soon as they become available.

If the IRE is also a serious (SAE), it will be reported using the same process as for other SAEs.

12.1.2. Attribution Definitions

Every effort should be made by the investigator to explain any AE and to assess its potential causal relationship, ie, to administration of the study vaccine or to alternative causes (eg, natural history of an underlying disease, concomitant therapies). This applies to all AEs, whether serious or non-serious. Assessment of causality must be done by a licensed study physician (the investigator or designee).

Causality of AEs should be assessed by the investigator based on the following:

Related: there is suspicion that there is a relationship between the study vaccine and the AE (without determining the extent of probability); there is a reasonable possibility that the study vaccine contributed to the AE.

Unrelated: there is no suspicion that there is a relationship between the study vaccine and the AE; there are other more likely causes and administration of the study vaccine is not suspected to have contributed to the AE.

By definition, all solicited AEs at the injection site (local) will be considered related to the study vaccine administration.

12.1.3. Severity Criteria

Adverse events and laboratory data will be coded for severity using the toxicity grading tables in Attachment 1, adapted from the FDA Toxicity Grading Scale for Healthy Adult and Adolescent Volunteers Enrolled in Preventive Vaccine Clinical Trials. For AEs not identified in the table, the following guidelines will apply:

**Mild**	Grade 1	Symptoms causing no or minimal interference with usual social and functional activities.
**Moderate**	Grade 2	Symptoms causing greater than minimal interference with usual social and functional activities.
**Severe**	≥Grade 3	Symptoms causing inability to perform usual social and functional activities.

12.2. Special Reporting Situations

Safety events of interest on a sponsor study vaccine that may require expedited reporting and/or safety evaluation include, but are not limited to:

- Accidental or occupational exposure to a sponsor study vaccine.
- Medication error involving a sponsor product (with or without subject exposure to the sponsor study vaccine, eg, name confusion).
- IREs.

Special reporting situations should be recorded in the CRF. Any special reporting situation that meets the criteria of an SAE should also be recorded on the Serious Adverse Event Form.

12.3. Procedures

12.3.1. All AEs

All AEs and special reporting situations, whether serious or non-serious, will be reported from the time a signed and dated ICF is obtained until 28 days post last dose (Day 85 in Groups 1, 2, and 4, Day 43 in Group 3, and Day 120 in a subset of Group 3). Serious AEs will be collected from signing of the ICF onwards until the end of the study. Subjects will record symptoms of unsolicited AEs and of local or systemic solicited AEs in the diary in the evening after each vaccination and then daily for the next 7 days.

All AEs, including out-of-range values from laboratory tests, that have not resolved by the end of the study, or that have not resolved upon discontinuation of the subject's participation in the study, must be followed until resolution, or until reaching a clinically stable endpoint.

Serious AEs must be reported by the investigator using the Serious Adverse Event Form. SUSARs will be reported even after the study is completed if the sponsor, the SMC, or the investigator becomes aware of them. The sponsor will evaluate any safety information that is spontaneously reported by an investigator beyond the time frame specified in the protocol.

The investigator will monitor and analyze the study data including all AE and clinical laboratory data as they become available and will make determinations regarding the severity of the adverse experiences and their relation to study vaccine. All AEs will be deemed related to study vaccine or not related to study vaccine, according to the definitions in Section 12.1.2. To ensure that all AEs are captured in a timely manner, the CRFs will be entered in real-time and subjected to review to identify AEs which may invoke pausing rules.

The investigator or designee must review both post-injection reactogenicity and other AE CRFs to insure the prompt and complete identification of all events that require expedited reporting as SAEs, invoke pausing rules, or are other serious and unexpected events.

All AEs, regardless of seriousness, severity, or presumed relationship to study vaccine, must be recorded using medical terminology in the source document and the CRF. Whenever possible, diagnoses should be given when signs and symptoms are due to a common etiology (eg, cough, runny nose, sneezing, sore throat, and head congestion should be reported as "upper respiratory infection"). Investigators must record in the CRF their opinion concerning the relationship of the AE to study vaccine. For causality assessment, the investigator or designee needs to be a licensed study physician. All measures required for AE management must be recorded in the source document and reported according to sponsor instructions.

The sponsor assumes responsibility for appropriate reporting of AEs to the regulatory authorities. The sponsor will also report SUSARs to the investigator (and the head of the investigational institute where required). The investigator (or sponsor where required) must report SUSARs to

the appropriate IEC/IRB that approved the protocol unless otherwise required and documented by the IEC/IRB. A SUSAR will be reported to regulatory authorities unblinded. Participating investigators and the IEC/IRB will receive a blinded SUSAR summary, unless otherwise specified.

Subjects will be provided with a “wallet (study) card” and instructed to carry this card with them for the duration of the study indicating the following:

- Study number.
- Statement, in the local language(s), that the subject is participating in a clinical study.
- Investigator’s name and 24-hour contact telephone number.
- The sponsor’s name and 24-hour contact telephone number (for medical staff only).
- Site number.
- Subject number.
- Information about who should be contacted in case of emergency.

12.3.2. SAEs

All SAEs occurring during the study must be reported to the appropriate sponsor contact person by study-site personnel within 24 hours of their knowledge of the event.

The site, upon receipt, will immediately forward any SAE reports to the sponsor and, in parallel, to the DMID. Information regarding SAEs will be transmitted to the sponsor using the Serious Adverse Event Form, which must be completed and signed by a physician from the study site, and transmitted to the sponsor and DMID within 24 hours. The initial and follow-up reports of an SAE should be made by facsimile (fax).

All SAEs that have not resolved by the end of the study, or that have not resolved upon discontinuation of the subject’s participation in the study, must be followed until any of the following occurs:

- The event resolves.
- The event stabilizes.
- The event returns to baseline, if a baseline value/status is available.
- The event can be attributed to agents other than the study vaccine or to factors unrelated to study conduct.
- It becomes unlikely that any additional information can be obtained (subject or healthcare practitioner refusal to provide additional information, lost to follow-up after demonstration of due diligence with follow-up efforts).

Suspected transmission of an infectious agent by a medicinal product will be reported as an SAE. Any event requiring hospitalization (or prolongation of hospitalization) that occurs during the

course of a subject's participation in a study must be reported as an SAE, except hospitalizations for the following:

- Hospitalizations not intended to treat an acute illness or AE (eg, social reasons such as pending placement in long-term care facility).
- Surgery or procedure planned before entry into the study (must be documented in the CRF). *Note: Hospitalizations that were planned before the signing of the ICF, and where the underlying condition for which the hospitalization was planned has not worsened, will not be considered SAEs. Any AE that results in a prolongation of the originally planned hospitalization is to be reported as a new SAE.*

During the entire study, the cause of death of a subject, whether or not the event is expected or associated with the study vaccine, is considered an SAE.

12.3.3. Immediate Reportable Events

One subject in the study VAC52150EBL2001 experienced a serious and very rare condition called "Miller Fisher syndrome". This condition consists of double vision, pain on moving the eye, and difficulty with balance while walking. Miller Fisher syndrome most commonly occurs following a recent infection. The subject experienced these symptoms about a week after suffering from a common cold and fever. The event happened about a month after boost vaccination with either MVA-BN-Filo or placebo. This subject had to go to the hospital for treatment and has recovered. After an extensive investigation, the event has been considered to be doubtfully related to vaccine and most likely related to the previous common cold.

Any events of neuroimmunologic significance (see Section 12.1.1) should be categorized as Immediately Reportable Events and should be reported throughout the study using the form provided **within 24 hours to the sponsor**. Events suggestive of the disorders considered IREs should be reported even if the final diagnosis has not been yet determined, and follow-up information and final diagnosis should be submitted to the sponsor as soon as they become available.

If an event meets SAE criteria (Section 12.3.2), it should be documented as such using the SAE form, as well as the relevant CRF AE page and complete IRE form page 3 to be included as part of the SAE report.

12.3.4. Pregnancy

All initial reports of pregnancy in female subjects must be reported to the sponsor by the study-site personnel within 24 hours of their knowledge of the event using the appropriate pregnancy notification form. Abnormal pregnancy outcomes (eg, spontaneous abortion, fetal death, stillbirth, congenital anomalies, or ectopic pregnancy) are considered SAEs and must be reported using the Serious Adverse Event Form. Any subject who becomes pregnant during the study must be promptly discontinued from further study vaccination but should continue participation in the study for safety follow-up.

Because the effect of the study vaccine on sperm is unknown, pregnancies in partners of male subjects included in the study will be reported by the study-site personnel as noted above.

Follow-up information regarding the outcome of the pregnancy and any postnatal sequelae in the infant will be required to be sent to the sponsor.

12.4. Contacting Sponsor Regarding Safety

The sponsor's medical monitor should be contacted regarding safety issues or questions regarding the study. The sponsor's medical monitor will be the first point of contact to discuss questions relating to study safety, ≥Grade 3 or higher AEs, and SAEs, unblinding, pausing rules, and dosing continuation. The sponsor's medical monitor will provide guidance to the investigator regarding protocol-related issues, decisions, and reporting of any safety-related issues throughout the duration of the study. The role and responsibility of the sponsor's medical monitor are further detailed in the Medical Monitoring Plan for the study. The names (and corresponding telephone numbers) of the individuals who should be contacted regarding SAE reports in this study are listed in the Contact Information page(s), which will be provided as a separate document.

13. PRODUCT QUALITY COMPLAINT HANDLING

A product quality complaint (PQC) is defined as any suspicion of a product defect related to manufacturing, labeling, or packaging, ie, any dissatisfaction relative to the identity, quality, durability, or reliability of a product, including its labeling or package integrity. A PQC may have an impact on the safety and efficacy of the product. Timely, accurate, and complete reporting and analysis of PQC information from studies are crucial for the protection of subjects, investigators, and the sponsor, and are mandated by regulatory agencies worldwide. The sponsor has established procedures in conformity with regulatory requirements worldwide to ensure appropriate reporting of PQC information; all studies conducted by the sponsor or its affiliates will be conducted in accordance with those procedures.

13.1. Procedures

All initial PQCs must be reported to the sponsor by the study-site personnel within 24 hours after being made aware of the event.

If the defect is combined with an SAE, the study-site personnel must report the PQC to the sponsor according to the SAE reporting timelines (see Section 12.3.2). A sample of the suspected product should be maintained for further investigation if requested by the sponsor.

13.2. Contacting Sponsor Regarding Product Quality

The sites should contact their independent study vaccine monitor (see Definition of Terms) for product quality issues as detailed in the Site Investigational Product Procedures Manual.

14. STUDY VACCINE INFORMATION

14.1. Physical Description of Study Vaccines

Ad26.Filo

Ad26.Filo is a trivalent vaccine composed of 3 recombinant Ad26 vectors expressing the EBOV, MARV, and SUDV GPs: Ad26.ZEBOV, Ad26.MARVA, and Ad26.SUDV.

The following 3 active pharmaceutical ingredients are formulated in a target 1:1:1 vp ratio at a nominal titer of 8×10^{10} vp/mL per individual monovalent vaccine (total titer: 2.4×10^{11} vp/mL):

- Ad26.ZEBOV (JNJ-61210474): a monovalent, replication-incompetent Ad26 vaccine, expressing the full length of the EBOV (formerly known as *Zaire ebolavirus*) Mayinga GP, and produced in the human PER.C6 cell line.
- Ad26.MARVA (JNJ-54712138): a monovalent, replication-incompetent Ad26 vaccine, expressing the full length MARV (formerly known as *Lake Victoria marburgvirus*) Angola GP, and produced in the human PER.C6 cell line.
- Ad26.SUDV (JNJ-61210487): a monovalent, replication-incompetent Ad26 vaccine, expressing the full length SUDV (formerly known as *Sudan ebolavirus*) Gulu GP and produced in the human PER.C6 cell line.

The Ad26.Filo vaccine is a colorless to slightly yellowish or brownish, clear to slightly opalescent suspension, practically free from particles. The vaccine will be supplied at a nominal titer of 2.4×10^{11} vp/mL (range: 1.2 to 3.6×10^{11} vp/mL) in single-use vials as a frozen liquid to be thawed before use. Each stoppered and sealed 2-mL glass vial contains an extractable volume of at least 0.5 mL. The product will be diluted to achieve a dose of 9×10^{10} total vp, or 3×10^{10} vp per Ad26 vector. The 9×10^{10} vp dose will be administered in a volume of 1 mL.

The nominal titer in the Ad26.Filo Investigator’s Brochure[14] and on the label reflects the nominal titer in compliance with the relevant product specifications. It does not reflect the release titer, which is determined by the release and stability studies and which will be described in the relevant study reports. The selected batch for this study will have a release titer aimed to be as close as possible to the nominal titer.

Further preparation details are provided in the Site Investigational Product Procedures Manual. Refer to the Ad26.Filo Investigator’s Brochure for a list of excipients.[14]

A diluent consisting of the drug product formulation buffer will be supplied.

The Ad26.Filo vaccine is manufactured under the responsibility of Janssen Vaccines & Prevention B.V., the Netherlands.

MVA-BN-Filo

MVA-BN-Filo (JNJ-63839880) is a multivalent vaccine expressing the EBOV Mayinga GP, the MARV Musoke GP, the SUDV Gulu GP, and the TAFV NP. It is manufactured in chicken embryo fibroblast cells derived from specific pathogen-free eggs.

The EBOV and SUDV GPs expressed by Ad26.Filo have 100% similarity with the EBOV and SUDV GPs expressed by MVA-BN-Filo. The MARV Angola GP expressed by Ad26.Filo has 93% similarity with the MARV Musoke GP expressed by MVA-BN-Filo.

The MVA-BN-Filo vaccine is a clear to milky, light yellow-colored suspension with no visible extraneous particles, that will be supplied at a nominal titer of $2x10^8$ Inf U/mL in 2-mL glass vials as a frozen liquid suspension to be thawed before use. Each stoppered and sealed 2-mL glass vial contains an extractable volume of at least 0.5 mL.

The product will be diluted to achieve a dose of $1x10^8$ Inf U/mL. No dilution will be needed to achieve the dose of $5x10^8$ Inf U/mL. Both doses will be administered in a volume of 1 mL.

The nominal titer in the MVA-BN-Filo Investigator's Brochure[16] and on the label reflects the nominal titer in compliance with the relevant product specifications. It does not reflect the release titer, which is determined by the release and stability studies and which will be described in the relevant study reports. The selected batch for this study will have a much higher release titer formulated to be as close as possible to $5x10^8$ Inf U/mL.

Further preparation details are provided in the Site Investigational Product Procedures Manual. Refer to the MVA-BN-Filo Investigator's Brochure for a list of excipients.[16]

A diluent consisting of Tris-buffered saline (10 mM Tris, 140 mM NaCl, pH 7.7) will be supplied.

The MVA-BN-Filo vaccine is manufactured under the responsibility of Janssen Vaccines & Prevention B.V., the Netherlands.

Ad26.ZEBOV

Ad26.ZEBOV (JNJ-61210474) is a monovalent, replication-incompetent Ad26-based vector that expresses the full-length EBOV Mayinga GP and is produced in the human cell line PER.C6.

The Ad26.ZEBOV vaccine is a colorless to slightly yellowish or brownish, clear to slightly opalescent suspension, practically free from particles. The vaccine will be supplied at a nominal titer of $1x10^{11}$ vp/mL in single-use vials as a frozen liquid to be thawed before use. Each stoppered and sealed 2-mL glass vial contains an extractable volume of at least 0.5 mL.

The nominal titer will be used throughout the study duration for dosing and dilution calculation purposes. To ensure that all treatment arms receive a total volume of 1 mL for the IM injection, the product will be diluted to achieve a dose of $5x10^{10}$ total vp, administered in a volume of 1 mL.

Further preparation details are provided in the Site Investigational Product Procedures Manual. Refer to the Ad26.ZEBOV Investigator's Brochure for a list of excipients.[15]

A diluent consisting of the drug product formulation buffer will be supplied.

The Ad26.ZEBOV vaccine is manufactured under the responsibility of Janssen Vaccines & Prevention B.V., the Netherlands.

Placebo

The placebo supplied for this study will be commercially available sterile normal saline (0.9% sodium chloride for injection).

14.2. Packaging and Labeling

All study vaccines will be manufactured and packaged in accordance with Good Manufacturing Practice. All study vaccines will be packaged and labeled under the responsibility of the sponsor. No study vaccine can be repacked or relabeled without prior approval from the sponsor.

For each component the nominal titer will be reflected on the labels.

Further details for study vaccine packaging and labeling can be found in the Site Investigational Product Procedures Manual.

14.3. Preparation, Handling, and Storage

Study vaccine must be stored at controlled temperatures: Ad26.Filo vials must be stored at ≤–65°C, Ad26.ZEBOV vials must be stored at ≤–60°C, and MVA-BN-Filo vials must be stored at ≤–20°C.

Diluents for both Ad26-based vectors (Ad26.Filo and Ad26.ZEBOV) and MVA-BN-Filo are stored at 2°C to 8°C. Placebo will be stored at 20ºC to 25ºC (see USP Controlled Room Temperature).

Vials must be stored in a secured location with no access for unauthorized personnel. All study product storage equipment, including refrigerators and freezers, must be equipped with a continuous temperature monitor and alarm. All study product storage equipment, including refrigerators and freezers, should be equipped with back-up power systems. In the event that study vaccine is exposed to temperatures outside the specified temperature ranges, all relevant data will be sent to the sponsor to determine if the affected study vaccine can be used or will be replaced. The affected study vaccine must be quarantined and not used until further instruction from the sponsor is received.

An unblinded site research pharmacist will prepare all doses for vaccine administration and provide it to the clinic for administration to subjects by a blinded study vaccine administrator (see Definition of Terms, and see Section 5 for details of blinding).

Full details on the receipt and storage at the site, the preparation, the holding time, and storage conditions from the time of preparation to delivery of Ad26.Filo, Ad26.ZEBOV, and MVA-BN-Filo are provided in the Site Investigational Product Procedures Manual.

14.4. Study Vaccine Accountability

The investigator is responsible for ensuring that all study vaccine received at the site is inventoried and accounted for throughout the study. The investigator is responsible for the distribution and disposition of study vaccine, and has ultimate responsibility for accountability. To maintain the blind, the investigator will delegate to the unblinded site research pharmacist responsibility for study product accountability. The site research pharmacist will be responsible for maintaining complete records and documentation of study product receipt, accountability, dispensation, temperature, and storage conditions, and final disposition of the study product. All study products, whether administered or not, must be documented on the appropriate study product accountability record or dispensing log. The study vaccine administered to the subject must be documented on the study vaccine accountability form. All study vaccine will be stored and disposed of according to the sponsor's instructions. Study-site personnel must not combine contents of the study vaccine containers.

Study vaccine must be handled in strict accordance with the protocol and the container label, and must be stored at the study site in a limited-access area or in a locked cabinet under appropriate environmental conditions. Unused study vaccine must be available for verification by the sponsor's study-site monitor during on-site monitoring visits. The return to the sponsor of unused study vaccine will be documented on the study vaccine return form. When the study site is an authorized destruction unit and study vaccine supplies are destroyed on site, this must also be documented on the study vaccine return form.

Potentially hazardous materials such as used needles, syringes, and vials containing hazardous liquids (other than the vials that contained the vaccines or diluent) should be disposed of immediately in a safe manner and therefore will not be retained for study vaccine accountability purposes.

Study vaccine will be dispensed by a blinded study vaccine administrator (see Time and Events Schedule). Study vaccine will be administered only to subjects participating in the study. Returned study vaccine must not be dispensed again, even to the same subject. Study vaccine may not be relabeled or reassigned for use by other subjects. The investigator agrees neither to dispense the study vaccine from, nor store it at, any site other than the study sites agreed upon with the sponsor.

15. STUDY-SPECIFIC MATERIALS

The investigator will be provided with the following supplies:

- Investigator's Brochure and Addendum for Ad26.Filo, Ad26.ZEBOV, and MVA-BN-Filo.
- Site Investigational Product Procedures Manual.
- Laboratory Manual.
- Electronic Data Capture (eDC) Manual/electronic CRF Completion Guidelines and Randomization Instructions.
- Sample ICF.

- Subject diaries.
- Rulers (to measure diameter of any erythema/redness and induration/swelling.
- Thermometers.
- Subject wallet cards.
- Recruitment tools, as applicable.

16. ETHICAL ASPECTS

16.1. Study-specific Design Considerations

Potential subjects will be fully informed of the risks and requirements of the study and, during the study, subjects will be given any new information that may affect their decision to continue participation. They will be told that their consent to participate in the study is voluntary and may be withdrawn at any time with no reason given and without penalty or loss of benefits to which they would otherwise be entitled. Only subjects who are fully able to understand the risks, benefits, and potential AEs of the study, and provide their consent voluntarily, will be enrolled.

The total blood volume to be collected from each subject is expected to be approximately 890 mL for Groups 1, 2, and 4 and 770 mL for Group 3 (940 mL for the subset of subjects in Group 3 receiving a third vaccination). The maximal amount of blood that will be drawn over a period of approximately 50 to 60 days will be 490 mL in all groups (Groups 1, 2, and 4: between Days 15 and 78, and between Days 29 and 92; Group 3: between Days 1 and 50).

This will not exceed the United States Department of Health and Human Services Office for Human Research Protection, and FDA guidelines of 550 mL in any 60-day period.

The primary ethical concern of this study is that it will be performed in healthy subjects, who will be exposed to several risks while receiving relatively little benefit from participation in the study. Potential risks and benefits from study participation are detailed below and in the Investigator's Brochure.

Risks Related to Vaccines

Subjects may exhibit general signs and symptoms associated with administration of a vaccine, or a placebo vaccination, including fatigue, nausea, headache, vomiting, myalgia, rash, arthralgia, general itching, fever, and chills. In addition, subjects may experience local (injection site) reactions such as pain/tenderness, erythema/redness, induration/swelling, itching and/or warmth at the injection site. These events will be monitored, but are generally short-term and do not require treatment.

Subjects may have an allergic reaction to the vaccination. An allergic reaction may cause a rash, hives, or even difficulty breathing. Severe reactions (eg, anaphylaxis) are rare. Medications must be available in the clinic to treat serious allergic reactions.

Fear of injection might result in a vasovagal response, hyperventilation, and in children sometimes in vomiting, breath-holding, and rarely convulsions.

Risks related to vaccine-induced seropositivity are discussed in Section 9.4.

Risk of Myocarditis or Pericarditis

While replicating smallpox vaccines have been associated with an increased risk to develop myocarditis/pericarditis,[21] this has not been observed with MVA-BN and is not expected with this highly attenuated, non-replicating vaccine. Based on observations with first- and second-generation replication-competent smallpox vaccines, particular attention has been placed on the monitoring for cardiac signs and symptoms in all clinical studies using MVA-BN. Despite the close cardiac monitoring, no event indicating a case of myocarditis has been observed in any completed MVA-BN study. Only 1 possible, albeit doubtful, case of pericarditis (consisting of chest pain only, with no other cardiac findings suggestive of pericarditis) has been observed in the MVA-BN clinical trial program. In a review of prospective surveillances for cardiac AEs in 6 different clinical studies in 382 subjects receiving MVA vaccines, only 1 subject (0.3%) met the criteria for vaccine-induced myocarditis, and eventually, the subject was found to suffer from exercise-induced palpitations. Self-limited mild elevations in troponin I were recorded in 3 (0.8%) subjects without evidence of myocarditis/pericarditis.[7]

For the present study, subjects will be actively screened to exclude preexisting cardiac concerns.

Pregnancy and Birth Control

The effect of the study vaccines on a fetus or nursing baby is not known, nor is the effect on semen known, so women of childbearing potential are required to agree to practice effective birth control measures for sexual intercourse from 28 days before the prime vaccination until at least 3 months after the last vaccination (see Inclusion Criterion #7) and men who have not had a vasectomy and are having sexual intercourse with women of childbearing potential must agree to using a barrier method of contraception (see Inclusion Criterion #7). Women who are pregnant or breastfeeding, or are planning to become pregnant while enrolled in the study or within 3 months after the last vaccination, will be excluded from enrollment into the study.

Women of childbearing potential must also agree not to donate eggs (ova, oocytes) for the purposes of assisted reproduction from the start of screening onwards until at least 3 months after the last vaccination.

Men must also agree not to donate sperm from the start of screening onwards until at least 3 months after the last vaccination.

Risks From Blood Draws

Blood draws may cause pain, bruising, bleeding, and, rarely, infection at the site where the blood is taken.

Unknown Risks

There may be other serious risks that are not known.

Potential Benefits

Subjects may benefit from clinical testing and physical examination, and financial compensation for the time and inconveniences that may arise from participation in the study; others may benefit from knowledge gained in this study that may aid in the development of a filovirus vaccine.

16.2. Regulatory Ethics Compliance

16.2.1. Investigator Responsibilities

The investigator is responsible for ensuring that the study is performed in accordance with the protocol, with current ICH, FDA and United States federal guidelines (United States CFR applicable to clinical studies [45 CFR Part 46; 21 CFR Part 50, 21 CFR Part 56, and 21 CFR Part 312]; subject to FDA waiver, as applicable) on Good Clinical Practice (GCP), and with applicable regulatory and country-specific requirements.

Good Clinical Practice is an international ethical and scientific quality standard for designing, conducting, recording, and reporting studies that involve the participation of human subjects. Compliance with this standard provides public assurance that the rights, safety, and well-being of study subjects are protected, consistent with the principles that originated in the Declaration of Helsinki, and that the study data are credible.

16.2.2. Independent Ethics Committee and Institutional Review Board

Before the start of the study, the investigator (or sponsor where required) will provide the IEC/IRB with current and complete copies of the following documents (as required by local regulations):

- Final protocol and, if applicable, amendments.
- Sponsor-approved ICF (and any other written materials to be provided to the subjects).
- Investigator's Brochure (or equivalent information) and amendments/addenda.
- Sponsor-approved subject recruiting materials.
- Information on compensation for study-related injuries or payment to subjects for participation in the study, if applicable.
- Investigator's curriculum vitae or equivalent information (unless not required, as documented by the IEC/IRB).
- Information regarding funding, name of the sponsor, institutional affiliations, other potential conflicts of interest, and incentives for subjects.
- Any other documents that the IEC/IRB requests to fulfill its obligation.

This study will be undertaken only after the IEC/IRB has given full approval of the final protocol, amendments (if any, excluding the ones that are purely administrative, with no consequences for subjects, data, or study conduct, unless required locally), the ICF, applicable recruiting materials, and subject compensation programs, and the sponsor has received a copy of this approval. This approval letter must be dated and must clearly identify the IEC/IRB and the documents being approved.

During the study the investigator (or sponsor where required) will send the following documents and updates to the IEC/IRB for their review and approval, where appropriate:

- Protocol amendments (excluding the ones that are purely administrative, with no consequences for subjects, data, or study conduct).
- Revision(s) to ICF and any other written materials to be provided to subjects.
- If applicable, new or revised subject recruiting materials approved by the sponsor.
- Revisions to compensation for study-related injuries or payment to subjects for participation in the study, if applicable.
- New edition(s) of the Investigator's Brochure and amendments/addenda.
- Summaries of the status of the study at intervals stipulated in guidelines of the IEC/IRB (at least annually).
- Reports of AEs that are serious, unlisted/unexpected, and associated with the study vaccine.
- New information that may adversely affect the safety of the subjects or the conduct of the study.
- Deviations from or changes to the protocol to eliminate immediate hazards to the subjects.
- Report of deaths of subjects under the investigator's care.
- Notification if a new investigator is responsible for the study at the site.
- Development Safety Update Report and Line Listings, where applicable.
- Any other requirements of the IEC/IRB.

For all protocol amendments (excluding the ones that are purely administrative, with no consequences for subjects, data, or study conduct), the amendment and applicable ICF revisions must be submitted promptly to the IEC/IRB for review and approval before implementation of the change(s).

At least once a year, the IEC/IRB will be asked to review and reapprove this study, where required.

At the end of the study, the investigator (or sponsor where required) will notify the IEC/IRB about the study completion.

16.2.3. Informed Consent

Each subject must give written consent according to local requirements after the nature of the study has been fully explained. The ICF(s) must be signed before performance of any study-related activity. The ICF(s) that is/are used must be approved by both the sponsor and by the reviewing IEC/IRB and be in a language that the subject can read and understand. The informed consent should be in accordance with principles that originated in the Declaration of Helsinki, current ICH and GCP guidelines, applicable regulatory requirements, and sponsor policy.

Before enrollment in the study, the investigator or an authorized member of the study-site personnel must explain to potential subjects the aims, methods, reasonably anticipated benefits, and potential hazards of the study, and any discomfort participation in the study may entail. Subjects will be informed that their participation is voluntary and that they may withdraw consent to participate at any time. They will be informed that choosing not to participate will not affect the care the subject will receive. Finally, they will be told that the investigator will maintain a subject identification register for the purposes of long-term follow up if needed and that their records may be accessed by health authorities and authorized sponsor personnel without violating the confidentiality of the subject, to the extent permitted by the applicable law(s) or regulations. By signing the ICF, the subject is authorizing such access and agrees to allow his or her study physician to recontact the subject for the purpose of obtaining consent for additional safety evaluations, if needed.

The subject will be given sufficient time to read the ICF and the opportunity to ask questions. After this explanation and before entry into the study, consent should be appropriately recorded by means of the subject's personally dated signature. After having obtained the consent, a copy of the ICF must be given to the subject.

Subjects will be asked for consent to provide samples for future research (where local regulations permit). A copy of this signed ICF will be given to the subject.

16.2.4. Privacy of Personal Data

The collection and processing of personal data from subjects enrolled in this study will be limited to those data that are necessary to fulfill the objectives of the study.

These data must be collected and processed with adequate precautions to ensure confidentiality and compliance with applicable data privacy protection laws and regulations. Appropriate technical and organizational measures to protect the personal data against unauthorized disclosures or access, accidental or unlawful destruction, or accidental loss or alteration must be put in place. Sponsor personnel whose responsibilities require access to personal data agree to keep the identity of subjects confidential.

The informed consent obtained from the subject includes explicit consent for the processing of personal data and for the investigator/institution to allow direct access to his or her original medical records (source data/documents) and CRF data by the monitors, auditors, IEC/IRB,

DMID, and the regulatory authorities. This consent also addresses the transfer of the data to other entities and to other countries.

The subject has the right to request through the investigator access to his or her personal data and the right to request rectification of any data that are not correct or complete. Reasonable steps will be taken to respond to such a request, taking into consideration the nature of the request, the conditions of the study, and the applicable laws and regulations.

Exploratory research is not conducted under standards appropriate for the return of data to subjects. In addition, the sponsor cannot make decisions as to the significance of any findings resulting from exploratory research. Therefore, exploratory research data will not be returned to subjects or investigators, unless required by law or local regulations. Privacy and confidentiality of data generated in the future on stored samples will be protected by the same standards applicable to all other clinical data.

16.2.5. Long-term Retention of Samples for Additional Future Research

Each study subject will be asked to consent voluntarily for their blood samples to be stored for other research studies that may be done after this study is completed. Future testing may involve deoxyribonucleic acid (DNA)/RNA tests. Subjects unwilling to have their blood samples stored for future use can consent to participate in this study without having their blood samples stored for future testing (see Section 10.4). In such case, their blood samples will be destroyed after all the tests specified for this study have been concluded.

All samples, for which consent has been obtained and for which additional material is available after study-specified testing is complete, will be stored for future testing. Applicable approvals will be sought before any such samples are used for analyses that are not specified in the protocol or a protocol amendment approved by the IEC/IRB.

Stored samples will be coded throughout the sample storage and analysis process and will not be labeled with personal identifiers.

16.2.6. Country Selection

This study will only be conducted in those countries where the intent is to launch or otherwise help ensure access to the developed study vaccine if the need for the product persists, unless explicitly addressed as a specific ethical consideration in Section 16.1 Study-specific Design Considerations.

16.3. Public Readiness and Emergency Preparedness Act

This protocol and the vaccine tested are covered under the Public Readiness and Emergency Preparedness (PREP) Act. The PREP Act provides compensation to participants in the event of serious physical injury or death caused by covered drugs and vaccines, and liability protection for persons conducting the clinical study and the manufacturer of the drug or vaccine.

The vaccines used in this clinical study are covered countermeasures under the PREP Act. This coverage provides immunity for covered persons (including manufacturers, distributers, program

planners, and other qualified persons who prescribe, administer, or dispense the vaccine) from tort liability, unless the injury was caused by willful misconduct.

The Act authorizes an emergency fund administered by the Health Resources and Services Administration via the Countermeasures Injury Compensation Program (CICP). The CICP provides compensation to eligible individuals who suffer specified injuries from administration or use of a countermeasure pursuant to the declaration. Any requests for compensation must be filed with the CICP within 1 year of administration or use of the countermeasure. For more information please visit the CICP website at http://www.hrsa.gov/cicp/. It is also advised participants consult an attorney in the event they suffer a serious injury and wish to file a claim for compensation with the CICP.

17. ADMINISTRATIVE REQUIREMENTS

17.1. Protocol Amendments

Neither the investigator nor the sponsor will modify this protocol without a formal amendment by the sponsor. All protocol amendments must be issued by the sponsor, and signed and dated by the investigator. Protocol amendments must not be implemented without prior IEC/IRB approval, or when the relevant competent authority has raised any grounds for non-acceptance, except when necessary to eliminate immediate hazards to the subjects, in which case the amendment must be promptly submitted to the IEC/IRB and relevant competent authority. Documentation of amendment approval by the investigator and IEC/IRB must be provided to the sponsor. When the change(s) involves only logistic or administrative aspects of the study, the IEC/IRB (where required) only needs to be notified.

During the course of the study, in situations where a departure from the protocol is unavoidable, the investigator or other physician in attendance will contact the appropriate sponsor representative listed in the Contact Information page(s), which will be provided as a separate document. Except in emergency situations, this contact should be made before implementing any departure from the protocol. In all cases, contact with the sponsor must be made as soon as possible to discuss the situation and agree on an appropriate course of action. The data recorded in the CRF and source documents will reflect any departure from the protocol, and the source documents will describe this departure and the circumstances requiring it.

17.2. Regulatory Documentation

17.2.1. Regulatory Approval/Notification

This protocol and any amendment(s) must be submitted to the appropriate regulatory authorities in each respective country, if applicable. A study may not be initiated until all local regulatory requirements are met.

17.2.2. Required Prestudy Documentation

The following documents must be provided to the sponsor before shipment of study vaccine to a study site:

- Protocol and amendment(s), if any, signed and dated by the principal investigator.
- A copy of the dated and signed (or sealed, where appropriate per local regulations), written IEC/IRB approval of the protocol, amendments, ICF, any recruiting materials, and if applicable, subject compensation programs. This approval must clearly identify the specific protocol by title and number and must be signed (or sealed, where appropriate per local regulations) by the chairman or authorized designee.
- Name and address of the IEC/IRB, including a current list of the IEC/IRB members and their function, with a statement that it is organized and operates according to GCP and the applicable laws and regulations. If accompanied by a letter of explanation, or equivalent, from the IEC/IRB, a general statement may be substituted for this list. If an investigator or a member of the study-site personnel is a member of the IEC/IRB, documentation must be obtained to state that this person did not participate in the deliberations or in the vote/opinion of the study.
- Regulatory authority approval or notification, if applicable.
- Signed and dated statement of investigator (eg, Form FDA 1572).
- Documentation of investigator qualifications (eg, curriculum vitae).
- Completed investigator financial disclosure form from the principal investigator, where required.
- Signed and dated clinical trial agreement, which includes the financial agreement.
- Any other documentation required by local regulations.

The following documents must be provided to the sponsor before enrollment of the first subject:

- Completed investigator financial disclosure forms from all subinvestigators.
- Documentation of subinvestigator qualifications (eg, curriculum vitae).
- Name and address of any local laboratory conducting tests for the study.
- Local laboratory documentation demonstrating competence and test reliability (eg, accreditation/license).

17.3. Subject Identification, Enrollment, and Screening Logs

The investigator agrees to complete a subject identification and enrollment log to permit easy identification of each subject during and after the study. This document will be reviewed by the sponsor study-site contact for completeness.

The subject identification and enrollment log will be treated as confidential and will be filed by the investigator in the study file. To ensure subject confidentiality, no copy will be made. All reports and communications relating to the study will identify subjects by subject identification number. In cases where the subject is not randomized into the study, the screening number and the date seen will be used.

The investigator must also complete a subject screening log, which reports on all subjects who were seen to determine eligibility for inclusion in the study.

17.4. Source Documentation

At a minimum, source documents consistent in the type and level of detail with those commonly recorded at a study site as a basis for standard medical care must be available for the following: subject identification, eligibility, and study identification; study discussion and date of signed informed consent; dates of visits; results of safety and efficacy parameters as required by the protocol; record of all AEs and follow-up of AEs; concomitant medication; study vaccine receipt/dispensing/return records; study vaccine administration information; and date of study completion and reason for early discontinuation of study vaccine or withdrawal from the study, if applicable. This source documentation may be in the form of either paper or electronic medical records or a mixture of both, depending on the site's procedures. In rare cases, data may be entered directly into the CRF, and the CRF will be the source. The protocol-specific source documentation requirement should be defined and documented at the start of the study in the 'Source Document Identification Form'.

The author of an entry in the source documents should be identifiable.

Specific details required as source data for the study and source data collection methods will be reviewed with the investigator before the study.

The minimum source documentation requirements for Sections 4.1 and 4.2 that specify a need for documented medical history are a complete history of medical notes at the site.

Inclusion and exclusion criteria not requiring documented medical history must be verified at a minimum by subject interview or other protocol-required assessment (eg, physical examination, laboratory assessment) and documented in the source documents.

The subject diary will be considered a source document. Information from the diary provided to subjects to record symptoms of unsolicited and solicited local and systemic AEs until 7 days after each vaccination, will be reviewed by the investigator or designee to transcribe into the relevant parts of the CRF as described in the CRF Completion Guidelines.

17.5. Case Report Form Completion and Data Handling

Case Report Forms are prepared and provided by the sponsor for each subject in electronic format. All CRF entries, corrections, and alterations must be made by the investigator or authorized study-site personnel. The investigator must verify that all data entries in the CRF are accurate and correct.

Medidata Rave®, a validated 21 CFR Part 11 compliant eDC system, will be used for this study. The data system includes password protection and internal quality checks, such as automatic range checks, to identify data that appear inconsistent, incomplete, or inaccurate.

The study data will be transcribed by study-site personnel from the source documents onto an electronic CRF and transmitted in a secure manner to the sponsor within the time frame agreed upon between the sponsor and the study site. The electronic file will be considered to be the CRF.

Worksheets may be used for the capture of some data to facilitate completion of the CRF. Any such worksheets will become part of the subject's source documentation. All data relating to the study must be recorded in the CRFs prepared by the sponsor. Data must be entered into the CRFs in English. Study-site personnel must complete the CRF as soon as possible after a subject visit, and the forms should be available for review at the next scheduled monitoring visit. The investigator must verify that all data entries in the CRFs are accurate and correct.

If necessary, queries will be generated in the eDC tool. If corrections to a CRF are needed after the initial entry into the CRF, this can be done either of the following ways:

- Investigator and study-site personnel can make corrections in the eDC tool at their own initiative or as a response to an auto query (generated by the eDC tool).
- Sponsor or sponsor delegate can generate a query for resolution by the investigator and study-site personnel.

As with all changes to the eCRF, an audit trail will be available to document all corrections to data for which the eCRF was the source. In addition, authorized study-site personnel will be asked to document these changes in a paper/electronic medical record.

The data for this study will include safety and laboratory (clinical, immunology) data. Clinical data collected during the study will be reviewed and cleaned on an ongoing basis as part of iterative data management processes. Adverse events and concomitant therapies will be coded using standard dictionary terminology in the clinical database.

17.6. Data Quality Assurance/Quality Control

Steps to be taken to ensure the accuracy and reliability of data include the selection of qualified investigators and appropriate study sites, review of protocol procedures with the investigator and study-site personnel before the study, and periodic monitoring visits by the sponsor. Written instructions will be provided for collection, handling, storage, and shipment of samples.

Guidelines for CRF completion will be provided and reviewed with study-site personnel before data entry in the Medidata Rave eDC system.

The investigational site is responsible for conducting routine quality assurance and quality control activities to internally monitor study progress and protocol compliance, per the Clinical Quality Management Plan. The principal investigator will provide direct access to all study-related sites, source data/documents, and reports for the purpose of monitoring and auditing by the sponsor, and inspection by local/regulatory authorities. The principal investigator ensures that all study personnel are appropriately trained and current documentation of training or certification is maintained on site.

The sponsor will review CRFs for accuracy and completeness during on-site monitoring visits and after transmission to the sponsor; any discrepancies will be resolved with the investigator or designee, as appropriate. After upload of the data into the study database they will be verified for accuracy and consistency with the data sources.

Clinical site monitors will verify that the study is conducted and data are generated, documented (recorded), and reported in compliance with the protocol, GCP, and the applicable regulatory requirements. Clinical monitoring reports will be submitted to the sponsor and DMID.

SGS Data Management will implement quality control procedures beginning with the Medidata Rave eDC system and generate data quality control checks that will be run on the database. Any missing data or data anomalies will be communicated to the site(s) for clarification and resolution.

17.7. Record Retention

In compliance with the ICH/GCP guidelines, the investigator/institution will maintain all CRFs and all source documents that support the data collected from each subject, as well as all study documents as specified in ICH/GCP Section 8 and all study documents as specified by the applicable regulatory requirement(s). The investigator/institution will take measures to prevent accidental or premature destruction of these documents.

Essential documents (per ICH guidelines) must be retained until at least 2 years after the last approval of a marketing application in an ICH region and until there are no pending or contemplated marketing applications in an ICH region or until at least 2 years have elapsed since the formal discontinuation of clinical development of the investigational product. These documents will be retained for a longer period if required by the applicable regulatory requirements or by an agreement with the sponsor. It is the responsibility of the sponsor to inform the investigator/institution as to when these documents no longer need to be retained.

If the responsible investigator retires, relocates, or for other reasons withdraws from the responsibility of keeping the study records, custody must be transferred to a person who will accept the responsibility. The sponsor must be notified in writing of the name and address of the new custodian. Under no circumstance shall the investigator relocate or dispose of any study documents before having obtained written approval from the sponsor.

If it becomes necessary for the sponsor or the appropriate regulatory authority to review any documentation relating to this study, the investigator/institution must permit access to such reports.

17.8. Protocol Deviations

A protocol deviation is any noncompliance with the clinical study protocol, study-specific standard operating procedures or methods of procedure, or current GCP and ICH guidelines. All deviations from the protocol must be captured in the source documents and deviation log. Deviations will be included in the SMC reports. Deviations must also be sent to the IEC/IRB per their guidelines. Any major findings (major protocol deviations or other significant issues)

identified during sponsor audits or routine monitoring will have Corrective and Preventative Actions agreed upon with the sponsor and implemented by the site. All protocol deviations will be captured in the source documents and deviation log.

17.9. Monitoring

Site monitoring is conducted to ensure that the human subject protections, study and laboratory procedures, study intervention administration, and data collection processes are of high quality and meet sponsor and ICH/GCP guidelines and applicable regulations, and to ensure that this study is conducted in accordance with the protocol, protocol-specific methods of procedure, and applicable sponsor standard operating procedures.

The sponsor will perform on-site monitoring visits per their monitoring guidelines. The monitor will record dates of the visits in a study-site visit log that will be kept at the study site. The first post-initiation visit will be made as soon as possible after enrollment has begun. The nature and location of all source documents will be identified to ensure that all sources of original data required to complete the CRF are known to the sponsor and study-site personnel and are accessible for verification by the sponsor study-site contact. If electronic records are maintained at the study site, the method of verification must be discussed with the study-site personnel.

Direct access to source documents (medical records) by the sponsor and DMID must be allowed for the purpose of verifying that the recorded data are consistent with the original source data. Findings from this review will be discussed with the study-site personnel and documented in the monitoring reports. The sponsor expects that, during monitoring visits, the relevant study-site personnel will be available, the source documents will be accessible, and a suitable environment will be provided for review of study-related documents. The monitor will meet with the investigator on a regular basis during the study to provide feedback on the study conduct.

There will be independent monitoring of the pharmacy and preparation of study vaccines by an unblinded monitor (independent study vaccine monitor; see Definition of Terms); regular monitors will be blinded.

In addition to on-site monitoring visits, remote contacts can occur. These remote contacts will be documented in remote monitoring/telephone contact reports and email correspondence that will be filed in the Trial Master File. It is expected that during these remote contacts, study-site personnel will be available to provide an update on the progress of the study at the site.

17.10. Study Completion/Termination

17.10.1. Study Completion/End of Study

The study is considered completed with the last visit for the last subject participating in the study. The final data from the study site will be sent to the sponsor (or designee) after completion of the final subject visit at that study site, in the time frame specified in the Clinical Trial Agreement.

17.10.2. Study Termination

The sponsor reserves the right to close a study site or terminate the study at any time for any reason at the sole discretion of the sponsor. Study sites will be closed upon study completion. A study site is considered closed when all required documents and study supplies have been collected and a study-site closure visit has been performed.

The investigator may initiate study-site closure at any time, provided there is reasonable cause and sufficient notice is given in advance of the intended termination.

Reasons for the early closure of a study site by the sponsor or investigator may include but are not limited to:

- Failure of the investigator to comply with the protocol, the requirements of the IEC/IRB or local health authorities, the sponsor's procedures, or GCP guidelines.
- Inadequate recruitment of subjects by the investigator.
- Discontinuation of further study vaccine development.

17.11. On-site Audits

Representatives of the sponsor's clinical Quality Assurance department may visit a study site at any time during or after completion of the study to conduct an audit of the study in compliance with regulatory guidelines and company policy. These audits will require access to all study records, including source documents, for inspection. Subject privacy must, however, be respected. The investigator and study-site personnel are responsible for being present and available for consultation during routinely scheduled study-site audit visits conducted by the sponsor or its designees.

Similar auditing procedures may also be conducted by agents of any regulatory body, either as part of a national GCP compliance program or to review the results of this study in support of a regulatory submission. The investigator should immediately notify the sponsor if he or she has been contacted by a regulatory agency concerning an upcoming inspection.

17.12. Use of Information and Publication

All information, including but not limited to, information regarding the heterologous regimen of Ad26.Filo and MVA-BN-Filo or the heterologous regimen of Ad26.ZEBOV and MVA-BN-Filo or the sponsor's operations (eg, patent application, formulas, manufacturing processes, basic scientific data, prior clinical data, formulation information) supplied by the sponsor to the investigator and not previously published, and any data, including exploratory research data, generated as a result of this study, are considered confidential and remain the sole property of the sponsor. The investigator agrees to maintain this information in confidence and use this information only to accomplish this study, and will not use it for other purposes without the sponsor's prior written consent.

The investigator understands that the information developed in the study will be used by the sponsor in connection with the continued development of the heterologous regimen of Ad26.Filo

and MVA-BN-Filo, and thus may be disclosed as required to other clinical investigators or regulatory agencies. To permit the information derived from the clinical studies to be used, the investigator is obligated to provide the sponsor with all data obtained in the study.

The results of the study will be reported in a Clinical Study Report generated by the sponsor and will contain data from all study sites that participated in the study. Recruitment performance or specific expertise related to the nature and the key assessment parameters of the study will be used to determine a coordinating investigator. Results of exploratory analyses performed after the Clinical Study Report has been issued will be reported in a separate report and will not require a revision of the Clinical Study Report. Study subject identifiers will not be used in publication of results. Any work created in connection with performance of the study and contained in the data that can benefit from copyright protection (except any publication by the investigator as provided for below) shall be the property of the sponsor as author and owner of copyright in such work.

Consistent with Good Publication Practices and International Committee of Medical Journal Editors guidelines, the sponsor with its partners shall have the right to publish such primary (multicenter) data and information without approval from the investigator. The investigator has the right to publish study site-specific data after the primary data are published upon agreement with the sponsor. If an investigator wishes to publish information from the study, a copy of the manuscript must be provided to the sponsor for review at least 90 days before submission for publication or presentation. Expedited reviews will be arranged for abstracts, poster presentations, or other materials. If requested by the sponsor in writing, the investigator will withhold such publication for up to an additional 90 days to allow for filing of a patent application. In the event that issues arise regarding scientific integrity or regulatory compliance, the sponsor will review these issues with the investigator. The sponsor will not mandate modifications to scientific content and does not have the right to suppress information. For multicenter study designs and substudy approaches, secondary results generally should not be published before the primary endpoints of a study have been published. Similarly, investigators will recognize the integrity of a multicenter study by not submitting for publication data derived from the individual study site until the combined results from the completed study have been submitted for publication, within 12 months of the availability of the final data (tables, listings, graphs), or the sponsor confirms there will be no multicenter study publication. Authorship of publications resulting from this study will be based on the guidelines on authorship, such as those described in the Uniform Requirements for Manuscripts Submitted to Biomedical Journals, which state that the named authors must have made a significant contribution to the design of the study or analysis and interpretation of the data, provided critical review of the paper, and given final approval of the final version.

Registration of Clinical Studies and Disclosure of Results

The sponsor will register and disclose the existence of and the results of clinical studies as required by law. The study will be listed in clinicaltrials.gov at least 21 days prior to start of enrollment.

REFERENCES

1. Adenoviral Vaccine Safety Database. Crucell Holland B.V. (20 March 2015.)
2. Capeding MR, Tran NH, Hadinegoro SR, et al.; CYD14 Study Group. Clinical efficacy and safety of a novel tetravalent dengue vaccine in healthy children in Asia: a phase 3, randomised, observer-masked, placebo-controlled trial. Lancet. 2014;384:1358-1365.
3. Centers for Disease Control and Prevention. Marburg hemorrhagic fever (Marburg HF). Chronology of Marburg Hemorrhagic Fever Outbreaks. Updated October 2014. http://www.cdc.gov/vhf/marburg/resources/outbreak-table html. Accessed 28 December 2015.
4. Clinical Study Protocol VAC52150EBL1002 Amendment 5. A Phase 1, Randomized, Placebo-Controlled, Observer-Blind Study to Evaluate the Safety, Tolerability and Immunogenicity of Heterologous and Homologous Prime-Boost Regimens Using MVA-BN-Filo® and Ad26.ZEBOV Administered in Different Doses, Sequences and Schedules in Healthy Adult Subjects, Followed by Open-Label Administration of a Third Vaccination at One Year Post-Prime in Subjects Initially Randomized to Active Vaccine. Crucell Holland B.V. (October 2015).
5. Clinical Study Protocol VAC52150EBL2001 Amendment 3/Country-specific France-1. A Randomized, Observer-blind, Placebo-controlled, Phase 2 Study to Evaluate the Safety, Tolerability and Immunogenicity of Three Prime-boost Regimens of the Candidate Prophylactic Vaccines for Ebola Ad26.ZEBOV and MVA-BN-Filo in Healthy Adults in Europe. Crucell Holland B.V. (January 2016).
6. Clinical Study Report VAC52150EBL1001; Primary Analysis. A Phase 1, First-in-Human Study to Evaluate the Safety, Tolerability and Immunogenicity of Heterologous Prime-Boost Regimens Using MVA-BN®-Filo and Ad26.ZEBOV Administered in Different Sequences and Schedules in Healthy Adults. Crucell Holland B.V. (November 2015).
7. Elizaga ML, Vasan S, Marovich MA, et al. Prospective surveillance for cardiac adverse events in healthy adults receiving modified vaccinia Ankara: a systematic review. PLoS ONE. 2013;8:54407.
8. Extended Topline Results VAC52150EBL1002; Primary Analysis. A Phase 1, Randomized, Placebo-Controlled, Observer-Blind Study to Evaluate the Safety, Tolerability and Immunogenicity of Heterologous and Homologous Prime-Boost Regimens Using MVA-BN-Filo® and Ad26.ZEBOV Administered in Different Doses, Sequences and Schedules in Healthy Adult Subjects. Crucell Holland B.V. (November 2015).
9. Food and Drug Administration. Center for Biologics Evaluation and Research. Guidance for Industry. Toxicity Grading Scale for Healthy Adult and Adolescent Volunteers Enrolled in Preventive Vaccine Clinical Trials, September 2007.
10. Friedrich BM, Trefry JC, Biggins JE, et al. Potential vaccines and post-exposure treatments for filovirus infections. Viruses. 2012;4(9):1619-1650.
11. Gidudu JF, Walco GA, Taddio A, et al./ The Brighton Immunization Site Pain Working Group. Immunization site pain: Case definition and guidelines for collection, analysis, and presentation of immunization safety data. http://www.sciencedirect.com/science/article/pii/S0264410X12005063. Accessed 30 October 2015.
12. IMVANEX suspension for injection; smallpox vaccine (Live Modified Vaccinia Virus Ankara) [Summary of Product Characteristics]. http://www.ema.europa.eu/docs/en_GB/document_library/EPAR_-_Product_Information/human/002596/WC500147896.pdf. Accessed 27 July 2015.
13. Investigator's Brochure: JNJ-55471468-AAA, JNJ-55471494-AAA, JNJ-55471520-AAA, JNJ-64219324-AAA (Ad26.Mos.HIV and Ad26.Mos4.HIV), Edition 3. Crucell Holland B.V. (December 2015).
14. Investigator's Brochure: Ad26.Filo, Edition 1.0. Janssen Vaccines & Prevention B.V. (July 2016).
15. Investigator's Brochure: JNJ-61210474 (Ad26.ZEBOV), Edition 4. Janssen Vaccines & Prevention B.V. (June 2016).
16. Investigator's Brochure: MVA-BN-Filo (MVA-mBN226B), Edition 10. Bavarian Nordic A/S (July 2016).
17. Baden LR, Karita E, Mutua G, et al. Assessment of the safety and immunogenicity of 2 novel vaccine platforms for HIV-1 prevention: a randomized trial. Ann Intern Med. 2016;164(5):313-322.

18. Kohl KS, Walop W, Gidudu J, et al./The Brighton Collaboration Local Reaction Working Group for Swelling at or near Injection Site. Swelling at or near injection site: Case definition and guidelines for collection, analysis and presentation of immunization safety data. http://www.sciencedirect.com/science/article/pii/S0264410X07004835. Accessed 30 October 2015.

19. Kohl KS, Walop W, Gidudu J, et al./The Brighton Collaboration Local Reaction Working Group for Induration at or near Injection Site. Induration at or near injection site: Case definition and guidelines for collection, analysis and presentation of immunization safety data. http://www.sciencedirect.com/science/article/pii/S0264410X07005038. Accessed 30 October 2015.

20. Marcy SM, Kohl KS, Dagan R, et al./The Brighton Collaboration Fever Working Group. Fever as an adverse event following immunization: case definition and guidelines of data collection, analysis, and presentation. http://www.sciencedirect.com/science/article/pii/S0264410X03006601. Accessed 30 October 2015.

21. Neff J, Modlin J, Birkhead GS, et al. Monitoring the safety of a smallpox vaccination program in the United States: report of the joint Smallpox Vaccine Safety Working Group of the advisory committee on immunization practices and the Armed Forces Epidemiological Board. Clin Infect Dis. 2008;46 Suppl.3:S258-S270.

22. Stittelaar KJ, Kuiken T, de Swart RL, et al. Safety of modified vaccinia virus Ankara (MVA) in immune-suppressed macaques. Vaccine. 2001;19:3700-3709.

23. Verheust C, Goossens M, Pauwels K, Breyer D. Biosafety aspects of modified vaccinia virus Ankara (MVA)-based vectors used for gene therapy or vaccination. Vaccine. 2012;30:2623-2632.

24. Vollmar J, Arndtz N, Eckl KM, et al. Safety and immunogenicity of IMVAMUNE, a promising candidate as a third generation smallpox vaccine. Vaccine. 2006;24:2065-2070.

25. WHO Fact Sheet Marburg haemorrhagic fever. November 2012. http://www.who.int/mediacentre/factsheets/fs_marburg/en. Accessed 29 January 2016.

26. WHO Fact Sheet No103. Ebola Virus Disease 2015. Updated January 2016. http://www.who.int/mediacentre/factsheets/fs103/en/. Accessed 29 January 2016.

ATTACHMENTS

Attachment 1: Toxicity Grading Scale Adapted from the FDA Toxicity Grading Scale for Healthy Adult and Adolescent Volunteers Enrolled in Preventive Vaccine Clinical Trials

Adapted from the FDA Toxicity Grading Scale for Healthy Adult and Adolescent Volunteers Enrolled in Preventive Vaccine Clinical Trials.[9]

The abbreviations used in the following tables are:

AE: adverse event; ALT: alanine aminotransferase; AST: aspartate aminotransferase; CPK: creatine phosphokinase; ER: emergency room; F: female; hpf: high power field; IV: intravenous; LFT: liver function test; M: male; mEq: milliequivalent; mm Hg: millimeter of mercury; NA: not applicable; PT: prothrombin time; PTT: partial thromboplastin time; rbc/RBC: red blood cell; U: unit; ULN: upper limit of the normal range; WBC: white blood cell

CLINICAL ABNORMALITIES

**Local Reaction to Injectable Product**	**Mild (Grade 1)**	**Moderate (Grade 2)**	**Severe (Grade 3)**	**Potentially Life Threatening (Grade 4)**
Pain	Does not interfere with activity	Repeated use of non-narcotic pain reliever >24 hours or interferes with activity	Any use of narcotic pain reliever or prevents daily activity	ER visit or hospitalization
Tenderness	Mild discomfort to touch	Discomfort with movement	Significant discomfort at rest	ER visit or hospitalization
Erythema/Redness[a]	2.5 - 5 cm	5.1 - 10 cm	>10 cm	Necrosis or exfoliative dermatitis
Induration/Swelling[b]	2.5 - 5 cm and does not interfere with activity	5.1 - 10 cm or interferes with activity	>10 cm or prevents daily activity	Necrosis

a In addition to grading the measured local reaction at the greatest single diameter, the measurement should be recorded as a continuous variable.

b Induration/swelling should be evaluated and graded using the functional scale as well as the actual measurement.

VITAL SIGNS

**Vital Signs**[a]	**Mild (Grade 1)**	**Moderate (Grade 2)**	**Severe (Grade 3)**	**Potentially Life Threatening (Grade 4)**
Fever (°C)[b]<br>(°F)[b]	38.0 - 38.4<br>100.4 - 101.1	38.5 - 38.9<br>101.2 - 102.0	39.0 - 40<br>102.1 - 104	>40<br>>104
Tachycardia - beats per minute	101 - 115	116 - 130	>130	ER visit or hospitalization for arrhythmia
Bradycardia - beats per minute[c]	50 - 54	45 - 49	<45	ER visit or hospitalization for arrhythmia
Hypertension (systolic) - mm Hg	141 - 150	151 - 155	>155	ER visit or hospitalization for malignant hypertension
Hypertension (diastolic) - mm Hg	91 - 95	96 - 100	>100	ER visit or hospitalization for malignant hypertension
Hypotension (systolic) – mm Hg	85 - 89	80 - 84	<80	ER visit or hospitalization for hypotensive shock

a Subject should be at rest for all vital signs measurements.
b Oral temperature; no recent hot or cold beverages or smoking.
c When resting heart rate is 60 - 100 beats per minute. Use clinical judgment when characterizing bradycardia among some healthy subject populations, for example, conditioned athletes.

SYSTEMIC

Systemic (General)	Mild (Grade 1)	Moderate (Grade 2)	Severe (Grade 3)	Potentially Life Threatening (Grade 4)
Nausea/vomiting	No interference with activity or 1-2 episodes/24 hours	Some interference with activity or >2 episodes/24 hours	Prevents daily activity, requires outpatient IV hydration	ER visit or hospitalization for hypotensive shock
Diarrhea	2 - 3 loose stools or <400 g/24 hours	4 - 5 stools or 400 - 800 g/24 hours	6 or more watery stools or >800 g/24 hours or requires outpatient IV hydration	ER visit or hospitalization
Headache	No interference with activity	Repeated use of non-narcotic pain reliever >24 hours or some interference with activity	Significant; any use of narcotic pain reliever or prevents daily activity	ER visit or hospitalization
Fatigue	No interference with activity	Some interference with activity	Significant; prevents daily activity	ER visit or hospitalization
Myalgia	No interference with activity	Some interference with activity	Significant; prevents daily activity	ER visit or hospitalization
**Systemic Illness**	**Mild (Grade 1)**	**Moderate (Grade 2)**	**Severe (Grade 3)**	**Potentially Life Threatening (Grade 4)**
Illness or clinical AE (as defined according to applicable regulations)	No interference with activity	Some interference with activity not requiring medical intervention	Prevents daily activity and requires medical intervention	ER visit or hospitalization

LABORATORY ABNORMALITIES

When reference is made to the within laboratory normal range, FDA toxicity grades should not be considered for inclusion. Due to an overlap of the local laboratory normal ranges and FDA toxicity grade for some parameters, a subject remains eligible if the lab value is within the normal range, while having a grade 1 toxicity. The FDA table does not include toxicity grading for hematocrit, RBC count or INR.

	Laboratory Normal Ranges*	Mild (Grade 1)	Moderate (Grade 2)	Severe (Grade 3)	Potentially Life Threatening (Grade 4)[b]
**Serum**[a]					
Sodium – hyponatremia mEq/L	NA	132 - 134	130 - 131	125 - 129	<125
Sodium – hypernatremia mEq/L	NA	144 - 145	146 - 147	148 - 150	>150
Potassium – hyperkalemia mEq/L	NA	5.1 - 5.2	5.3 - 5.4	5.5 - 5.6	>5.6
Potassium – hypokalemia mEq/L	NA	3.5 - 3.6	3.3 - 3.4	3.1 - 3.2	<3.1
Glucose – hypoglycemia mg/dL	NA	65 - 69	55 - 64	45 - 54	<45
Glucose – hyperglycemia Fasting mg/dL	NA	100 - 110	111 - 125	>125	Insulin requirements or hyperosmolar coma
Glucose – hyperglycemia Random mg/dL	NA	110 - 125	126 - 200	>200	Insulin requirements or hyperosmolar coma
Blood nrea nitrogen mg/dL	NA	23 - 26	27 - 31	>31	Requires dialysis
Creatinine – mg/dL	0.4-1.1(F) 0.5-1.2 (M)	1.5 - 1.7	1.8 - 2.0	2.1 - 2.5	>2.5 or requires dialysis
Calcium – hypocalcemia mg/dL	NA	8.0 - 8.4	7.5 - 7.9	7.0 - 7.4	<7.0
Calcium – hypercalcemia mg/dL	NA	10.5 - 11.0	11.1 - 11.5	11.6 - 12.0	>12.0
Magnesium – hypomagnesemia mg/dL	NA	1.3 - 1.5	1.1 - 1.2	0.9 - 1.0	<0.9
Phosphorous – hypophosphatemia mg/dL	NA	2.3 - 2.5	2.0 - 2.2	1.6 - 1.9	<1.6
CPK – mg/dL	NA	1.25 - 1.5x ULN	1.6 - 3.0x ULN	3.1 - 10x ULN	>10x ULN
Albumin – Hypoalbuminemia g/dL	NA	2.8 - 3.1	2.5 - 2.7	<2.5	--
Total Protein – Hypoproteinemia g/dL	NA	5.5 - 6.0	5.0 - 5.4	<5.0	--
Alkaline phosphate – increase by factor	NA	1.1 - 2.0x ULN	2.1 - 3.0x ULN	3.1 - 10x ULN	>10x ULN
AST – increase by factor	9-34 (F) 11-36 (M)	1.1 - 2.5x ULN	2.6 - 5.0x ULN	5.1 - 10x ULN	>10x ULN
ALT – increase by factor	6-34 (F) 6-43 (M)	1.1 - 2.5x ULN	2.6 - 5.0x ULN	5.1 - 10x ULN	>10x ULN
Bilirubin – when accompanied by any increase in LFT (ALT or AST); increase by factor	NA	1.1 - 1.25x ULN	1.26 - 1.5x ULN	1.51 - 1.75x ULN	>1.75x ULN
Bilirubin – when LFT is normal; increase by factor	NA	1.1 - 1.5x ULN	1.6 - 2.0x ULN	2.0 - 3.0x ULN	>3.0x ULN

	Laboratory Normal Ranges*	Mild (Grade 1)	Moderate (Grade 2)	Severe (Grade 3)	Potentially Life Threatening (Grade 4)[b]
Cholesterol – mg/dL	NA	201 - 210	211 - 225	>226	--
Amylase – increase by factor	NA	1.1 - 1.5x ULN	1.6 - 2.0x ULN	2.1 - 5.0x ULN	>5.0x ULN
Lipase – increase by factor	NA	1.1 - 1.5x ULN	1.6 - 2.0x ULN	2.1 - 5.0x ULN	>5.0x ULN
**Hematology**[a]					
Hemoglobin (Female) – g/dL	11.6 – 16.4	11.0 - 12.0	9.5 - 10.9	8.0 - 9.4	<8.0
Hemoglobin (Female) – change from baseline; g/dL	NA	Any decrease - 1.5	1.6 - 2.0	2.1 - 5.0	>5.0
Hemoglobin (Male) – g/dL	12.7 – 18.1	12.5 - 13.5	10.5 - 12.4	8.5 - 10.4	<8.5
Hemoglobin (Male) – change from baseline; g/dL	NA	Any decrease - 1.5	1.6 - 2.0	2.1 - 5.0	>5.0
WBC Increase – cell/mm^3	3,800 – 10,700	10,800 - 15,000	15,001 - 20,000	20,001 - 25,000	>25,000
WBC Decrease – cell/mm^3	3,800 – 10,700	2,500 - 3,500	1,500 - 2,499	1,000 - 1,499	<1,000
Lymphocytes Decrease – cell/mm^3	910 – 4,280	750 - 1,000	500 - 749	250 - 499	<250
Neutrophils Decrease – cell/mm^3	1,960 – 7,230	1,500 - 2,000	1,000 - 1,499	500 - 999	<500
Eosinophils – cell/mm^3	0 - 570	650 - 1,500	1,501 - 1,500	>5,000	Hypereosino-philic
Platelets Decreased – cell/mm^3	140,000 – 400,000	125,000 - 140,000	100,000 - 124,000	25,000 - 99,000	<25,000
PT – increase by factor (prothrombin time)	9.7 - 12.3 sec	1.0 - 1.10x ULN	1.11 - 1.20x ULN	1.21 - 1.25x ULN	>1.25x ULN
PTT – increase by factor (partial thromboplastin time)	23.6 - 32.5 sec	1.0 - 1.2x ULN	1.21 - 1.4x ULN	1.41 - 1.5x ULN	>1.5x ULN
Fibrinogen increase – mg/dL	NA	400 - 500	501 - 600	>600	--
Fibrinogen decrease – mg/dL	NA	150 - 200	125 - 149	100 - 124	<100 or associated with gross bleeding or disseminated intravascular coagulation (DIC)
**Urine**[a]					
Protein	NA	Trace	1+	2+	Hospitalization or dialysis
Glucose	NA	Trace	1+	2+	Hospitalization for hyperglycemia
Blood (microscopic) - rbc/hpf	0 - 8 (F)<br>0 – 3 (M)	1 - 10	11 - 50	>50 and/or gross blood	Hospitalization or packed RBC (PRBC) transfusion

a The laboratory values provided in the tables serve as guidelines and are dependent upon institutional normal parameters. Institutional normal reference ranges should be provided to demonstrate that they are appropriate.

b The clinical signs or symptoms associated with laboratory abnormalities might result in characterization of the laboratory abnormalities as Potentially Life Threatening (Grade 4). For example, a low sodium value that falls within a Grade 3 parameter (125-129 mEq/L) should be recorded as a Grade 4 hyponatremia event if the subject had a new seizure associated with the low sodium value.

* Only normal ranges are added for these tests which are applicable to the trial

Note: normal ranges in overlap with grade 1 laboratory toxicities are indicated in grey.

INVESTIGATOR AGREEMENT

I have read this protocol and agree that it contains all necessary details for carrying out this study. I will conduct the study as outlined herein and will complete the study within the time designated.

The study will be carried out in accordance with GCP as required by the following:

United States (US) Code of Federal Regulations (CFR) applicable to clinical studies (45 CFR Part 46; 21 CFR Part 50, 21 CFR Part 56, and 21 CFR Part 312); subject to FDA waiver, as applicable.

ICH-GCP Guidance E6; 62 Federal Register 25691 (1997).

I will provide copies of the protocol and all pertinent information to all individuals responsible to me who assist in the conduct of this study. I will discuss this material with them to ensure that they are fully informed regarding the study vaccine, the conduct of the study, and the obligations of confidentiality.

All key personnel (all individuals responsible for the design and conduct of this study) will complete Human Subjects Protection Training such as ICH-GCP training or equivalent, prior to their involvement with this study.

Coordinating Investigator (where required):

Name (typed or printed): ______________________

Institution and Address: ______________________

____

____

____

Signature: ______________________ Date: ______________________

(Day Month Year)

Principal (Site) Investigator:

Name (typed or printed): ______________________

Institution and Address: ______________________

____

____

____

Telephone Number: ______________________

Signature: ______________________ Date: ______________________

(Day Month Year)

Sponsor's Responsible Medical Officer:

Name (typed or printed): PPD, MD

Institution: Janssen Vaccines & Prevention B.V.

Signature: electronic signature appended at the end of the protocol Date: ______________________

(Day Month Year)

Note: If the address or telephone number of the investigator changes during the course of the study, written notification will be provided by the investigator to the sponsor, and a protocol amendment will not be required.

LAST PAGE

SIGNATURES

Signed by	Date	Justification
PPD	12Dec2017, 12:48:15 PM, UTC	Document Approval
